# Supplementary material for: Maintenance of p-eIF2α levels by the eIF2B complex is vital for colorectal cancer
Source: EMBO J. 2025 Feb 27;44(7):2075–105. doi: 10.1038/s44318-025-00381-9 (PMC11962125; doi:10.1038/s44318-025-00381-9)
Supplement: Supplementary file 2 — Appendix [file 44318_2025_381_MOESM2_ESM.pdf]

Appendix

Maintenance of p-eIF2α levels by the eIF2B complex is vital for colorectal cancer

Ivana Paskov Škapik[1,2,14*], Chiara Giacomelli[3*], Sarah Hahn[1,2,14], Hanna Deinlein[1,2], Peter Gallant[1], Mathias Diebold[1,4], Josep Biayna[1,2,5], Anne Hendricks[1,2], Leon Olimski[1,2], Christoph Otto[2], Carolin Kastner[2], Elmar Wolf[1,6], Christina Schülein-Völk[1], Katja Maurus[7], Andreas Rosenwald[7], Nikolai Schleussner[8,9,10,11], Rene-Filip Jackstadt[8,10,11], Nicolas Schlegel[2], Christoph-Thomas Germer[2,12], Martin Bushell[3,13], Martin Eilers[1,12], Stefanie Schmidt[1,2§] and Armin Wiegering[1,2,12,14§]

1) Theodor Boveri Institute, Biocenter, University of Würzburg, 97074 Würzburg, Germany

2) Department of General, Visceral, Transplant, Vascular and Pediatric Surgery, University Hospital Würzburg, 97080 Würzburg, Germany

3) CRUK Scotland Institute, Garscube Estate, Switchback Road, Glasgow, G61 1BD, UK

4) Institute of Pharmacy and Food Chemistry, University of Würzburg, 97074 Würzburg, Germany

5) Institute of Cardiovascular Regeneration, Centre for Molecular Medicine, Goethe University Frankfurt, 60590 Frankfurt am Main, Germany

6) Institute of Biochemistry, CAU Kiel, 24118 Kiel, Germany

7) Institute of Pathology, University of Würzburg, 97074 Würzburg, Germany

8) Heidelberg Institute for Stem Cell Technology and Experimental Medicine (HI-STEM gGmbH), Heidelberg, Germany

9) Department of General, Visceral and Transplantation Surgery, University Hospital Heidelberg, University Heidelberg, 69120 Heidelberg, Germany

10) Cancer Progression and Metastasis Group, German Cancer Research Center (DKFZ) and DKFZ-ZMBH Alliance, Heidelberg, Germany

11) German Cancer Consortium (DKTK), DKFZ, Core Center Heidelberg, Heidelberg, Germany

12) Comprehensive Cancer Center Mainfranken, University Hospital Würzburg, 97080 Würzburg, Germany

13) School of Cancer Sciences, University of Glasgow, Garscube Estate, Switchback Road, Glasgow, G61 1QH, UK

14) Goethe University Frankfurt, University Hospital, Department of General, Visceral, Transplant and Thoracic Surgery, Frankfurt am Main, Germany.

§) Correspondence: wiegering_a@ukw.de; schmidt_s12@ukw.de

*) These authors contributed equally.

Table of Content:

Appendix Figure S1 page 3
Appendix Figure S2 page 4
Appendix Figure S3 pages 5-6
Appendix Figure S4 pages 7-8
Appendix Figure S5 page 9
Appendix Figure S6 pages 10-11
Appendix Figure S7 page 12
Appendix Figure S8 pages 13-14
Appendix Figure S9 page 15-16
Appendix Table S1 page 17

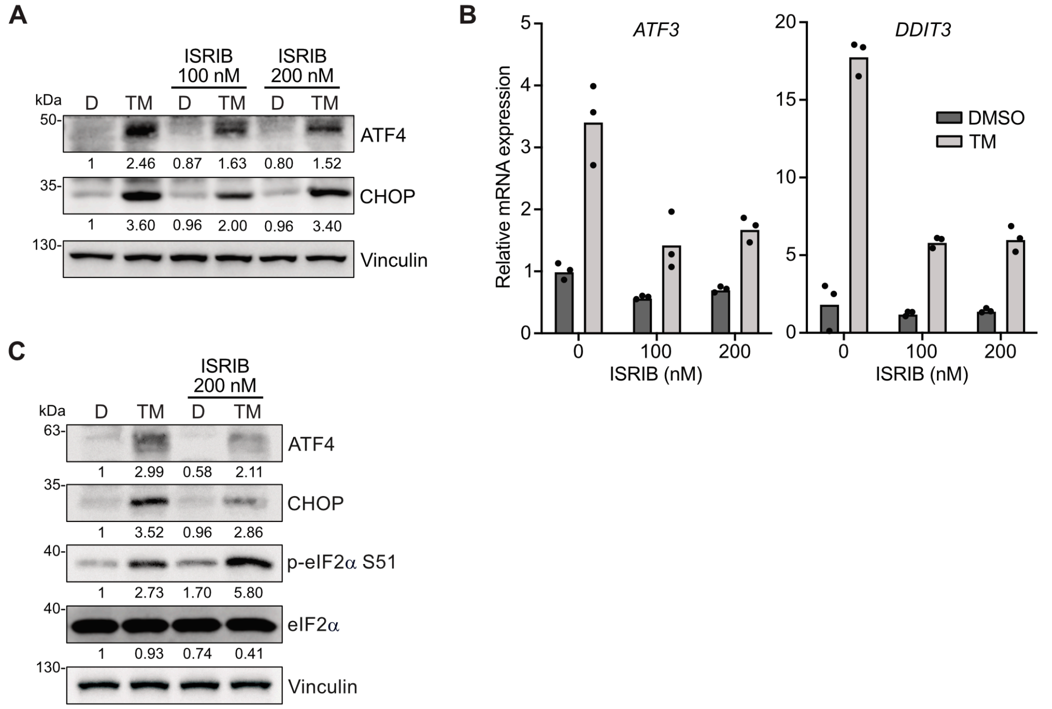

Appendix Figure S1: ISRIB reduces TM-induced ISR in CRC.
(A) Western blot of indicated proteins in SW480 cells treated with tunicamycin (TM, 1 µg/ml), ISRIB at indicated concentrations or DMSO as control for 3 hrs. The western blot is representative of two biological replicates with similar results. Levels of ATF4 and CHOP, normalized to vinculin, are given below each corresponding panel.
(B) mRNA expression of indicated genes in SW480 cells treated as described in (A). Data show mean of three technical triplicates of one experiment.
(C) Western blot of indicated proteins in LAKTP organoids treated with TM (1 µg/ml), ISRIB (200 nM) or DMSO as control for 3 hrs. The western blot is representative of two biological replicates with similar results. Levels of ATF4, CHOP, p-eIF2α S51 and total eIF2α, normalized to vinculin, are given below each corresponding panel.

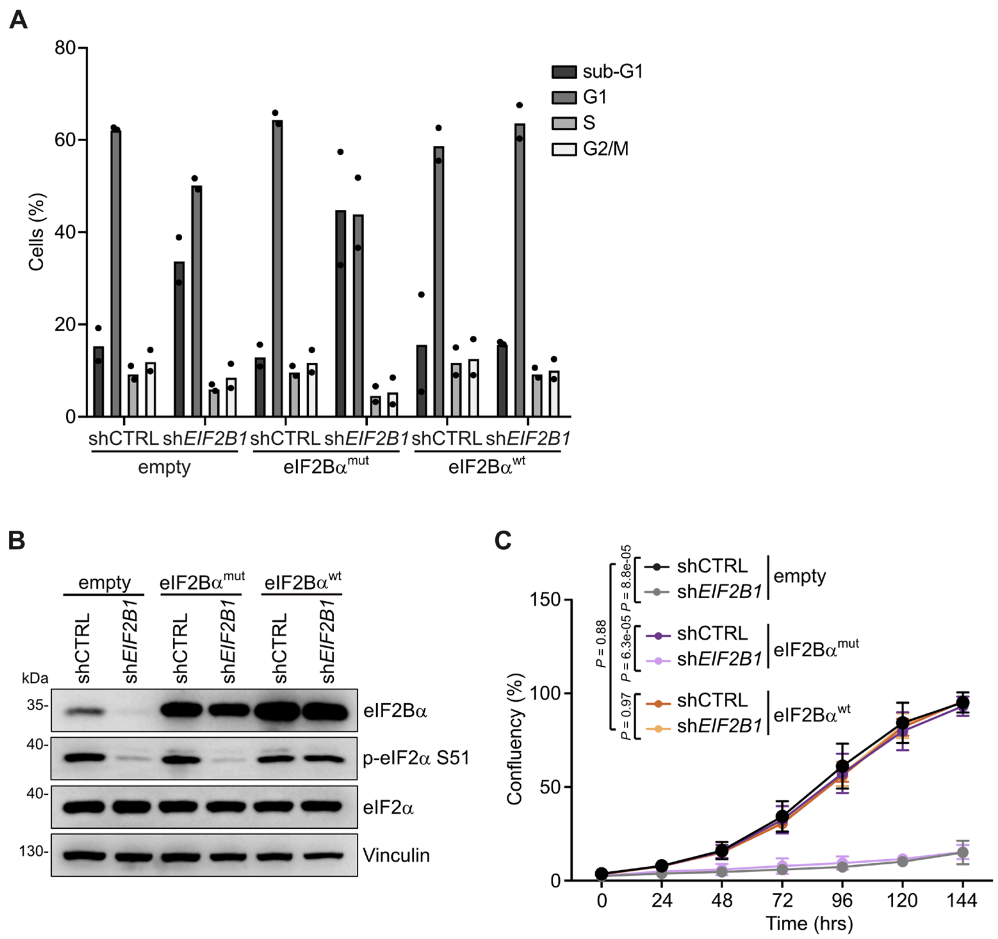

Appendix Figure S2: Disrupting the eIF2Bα homodimer is detrimental for PDAC cells.

(A) PI cell cycle FACS analysis of shCTRL- or sh*EIF2B1*-transduced SW480 cells stably overexpressing eIF2Bα mutant (eIF2Bα^{mut}), eIF2Bα WT (eIF2Bα^{wt}) construct, or without any overexpression (empty). Data show mean (*n* = 2 biological replicates).

(B) Western blot of indicated proteins in shCTRL- or sh*EIF2B1*-transduced PaTu8988T cells stably overexpressing eIF2Bα mutant (eIF2Bα^{mut}), eIF2Bα WT (eIF2Bα^{wt}) construct, or without any overexpression (empty). The western blot is representative of three biological replicates with similar results.

(C) Growth curve of PaTu8988T cells transduced as described in (B), measured with Incucyte® live cell imaging system. Data show mean ± s.d. (*n* = 3 biological replicates); Student's *t*-test.

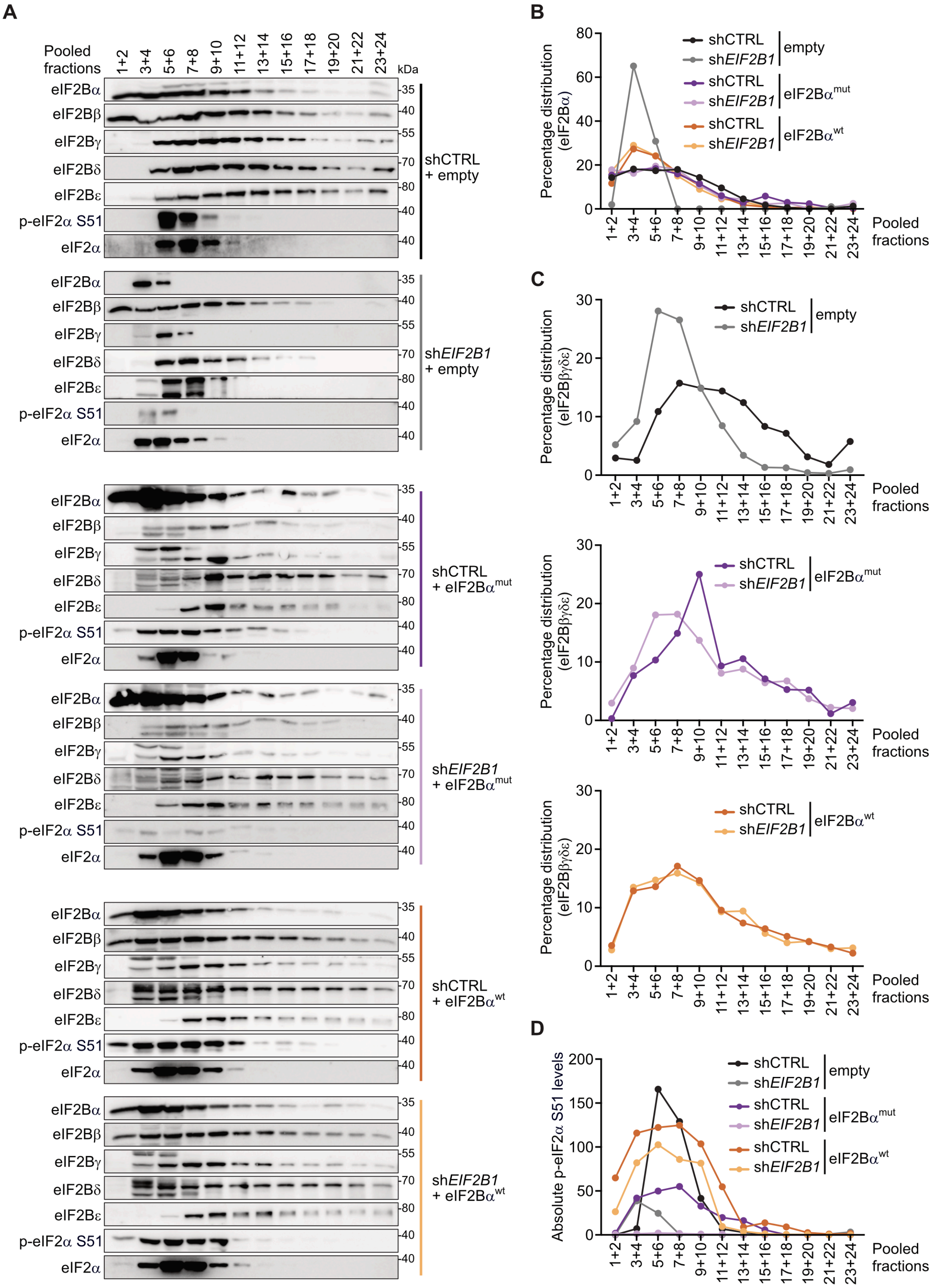
A
Pooled fractions
1+2
3+4
5+6
7+8
9+10
11+12
13+14
15+16
17+18
19+20
21+22
23+24
kDa
eIF2Bα
eIF2Bβ
eIF2Bγ
eIF2Bδ
eIF2Bε
p-eIF2α S51
eIF2α
35
40
55
70
80
shCTRL + empty
shEIF2B1 + empty
shCTRL + eIF2Bα^mut
shEIF2B1 + eIF2Bα^mut
shCTRL + eIF2Bα^wt
shEIF2B1 + eIF2Bα^wt
B
Percentage distribution (eIF2Bα)
shCTRL
shEIF2B1
empty
eIF2Bα^mut
eIF2Bα^wt
C
Percentage distribution (eIF2Bβγδε)
D
Absolute p-eIF2α S51 levels
Pooled fractions

Appendix Figure S3: Depletion of eIF2Bα and disruption of its homodimer dissociates the eIF2B decamer.

(A) Western blot of indicated proteins in shCTRL- or sh*EIF2B1*-transduced SW480 cells stably overexpressing eIF2Bα mutant (eIF2Bα^{mut}), eIF2Bα WT (eIF2Bα^{wt}) construct, or without any overexpression (empty). Each lane corresponds to two pooled fractions of sucrose gradients. Western blots of eIF2Bαβγδε subunits are representative of two biological replicates with similar results, western blots of total eIF2α and p-eIF2α S51 were performed once. Total eIF2α and p-eIF2α S51 western blots of samples “shCTRL+empty” are the same as shown in Appendix Fig. S4A. The experiments for shCTRL, sh*EIF2B1* and sh*EIF2B4* (Appendix Fig. S3 and Appendix Fig. S4) were done in parallel and "shCTRL+empty" served as control for both knockdown conditions.

(B) Quantification of eIF2Bα western blots from (A). Percentage distribution over all fractions is shown.

(C) Quantification of eIF2Bβγδε western blots from (A). Percentage distribution of the sum of eIF2Bβγδε over all fractions is shown.

(D) Quantification of p-eIF2α S51 western blots from (A). Absolute levels of p-eIF2α S51 over all fractions are shown. Quantification for p-eIF2α S51 in "shCTRL+empty" condition is the same as in Appendix Fig. S4D. The experiments for shCTRL, sh*EIF2B1* and sh*EIF2B4* (Appendix Fig. S3 and Appendix Fig. S4) were done in parallel and "shCTRL+empty" served as control for both knockdown conditions.

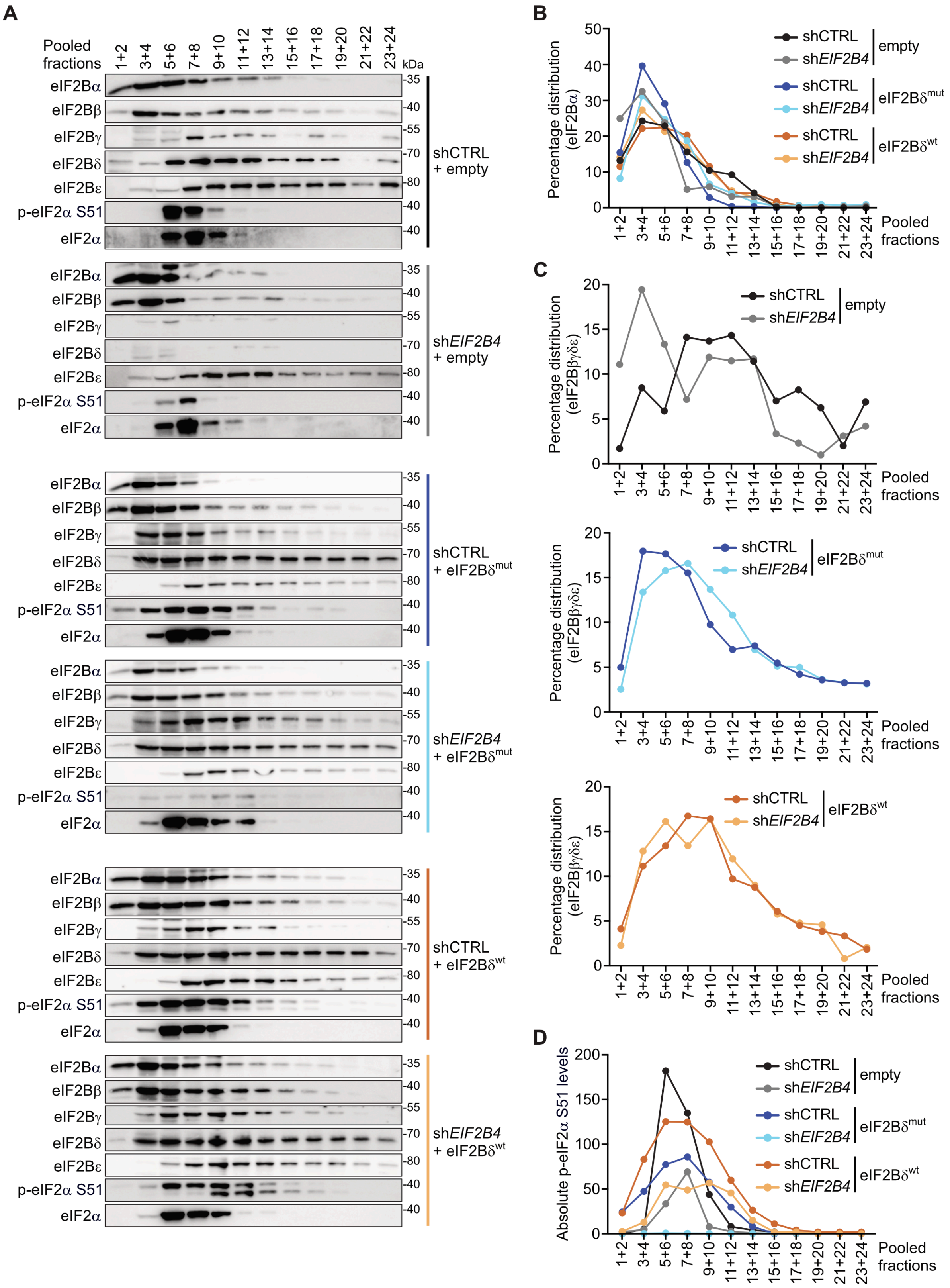
A
B
C
D
Pooled fractions
1+2
3+4
5+6
7+8
9+10
11+12
13+14
15+16
17+18
19+20
21+22
23+24
kDa
eIF2Bα
eIF2Bβ
eIF2Bγ
eIF2Bδ
eIF2Bε
p-eIF2α S51
eIF2α
shCTRL + empty
shEIF2B4 + empty
shCTRL + eIF2Bδmut
shEIF2B4 + eIF2Bδmut
shCTRL + eIF2Bδwt
shEIF2B4 + eIF2Bδwt
Percentage distribution (eIF2Bα)
Percentage distribution (eIF2Bβγδε)
Absolute p-eIF2α S51 levels
shCTRL
shEIF2B4
empty
eIF2Bδmut
eIF2Bδwt

Appendix Figure S4: Depletion of eIF2Bδ disrupts the eIF2B decamer.

(A) Western blot of indicated proteins in shCTRL- or sh*EIF2B4*-transduced SW480 cells stably overexpressing eIF2Bδ mutant (eIF2Bδ^{mut}), eIF2Bδ WT (eIF2Bδ^{wt}) construct, or without any overexpression (empty). Each lane corresponds to two pooled fractions of sucrose gradients. The western blots were performed once. Total eIF2α and p-eIF2α S51 western blots of samples “shCTRL+empty” are the same as shown in Appendix Fig. S3A. The experiments for shCTRL, sh*EIF2B1* and sh*EIF2B4* (Appendix Fig. S3 and Appendix Fig. S4) were done in parallel and "shCTRL+empty" served as control for both knockdown conditions.

(B) Quantification of eIF2Bα western blots from (A). Percentage distribution over all fractions is shown.

(C) Quantification of eIF2Bβγδε western blots from (A). Percentage distribution of the sum of eIF2Bβγδε over all fractions is shown.

(D) Quantification of p-eIF2α S51 western blots from (A). Absolute levels of p-eIF2α S51 over all fractions are shown. Quantification for p-eIF2α S51 in "shCTRL+empty" condition is the same as in Appendix Fig. S3D. The experiments for shCTRL, sh*EIF2B1* and sh*EIF2B4* (Appendix Fig. S3 and Appendix Fig. S4) were done in parallel and "shCTRL+empty" served as control for both knockdown conditions.

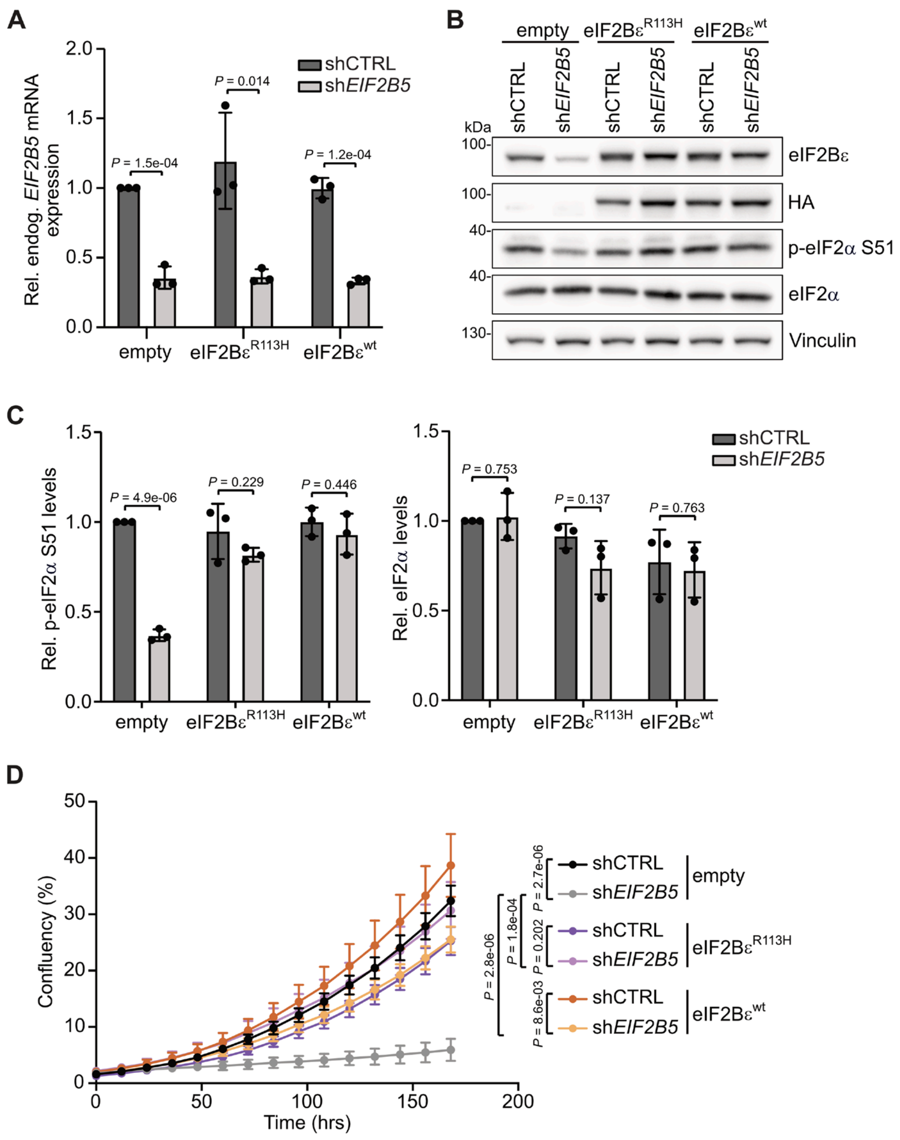

Appendix Figure S5: The decrease of p-eIF2α by eIF2B complex disruption is independent of eIF2B's GEF activity.

(A) mRNA expression of endogenous *EIF2B5* in shCTRL- or sh*EIF2B5*-transduced SW480 cells stably overexpressing eIF2Bε R113H mutant (eIF2Bε^{R113H}), eIF2Bε WT (eIF2Bε^{wt}) construct, or without any overexpression (empty). Data show mean ± s.d. (*n* = 3 biological replicates); Student's *t*-test.

(B) Western blot of indicated proteins in SW480 cells transduced as described in (A), representative of three biological replicates.

(C) Quantification of p-eIF2α S51 levels (left) and total eIF2α levels (right), normalized to vinculin, of western blots described in (B). Data show mean ± s.d. (*n* = 3 biological replicates); Student's *t*-test.

(D) Growth curve of SW480 cells transduced as described in (A), measured with Incucyte® live-cell imaging system. Data show mean ± s.d. (*n* = 4 biological replicates); Student's *t*-test.

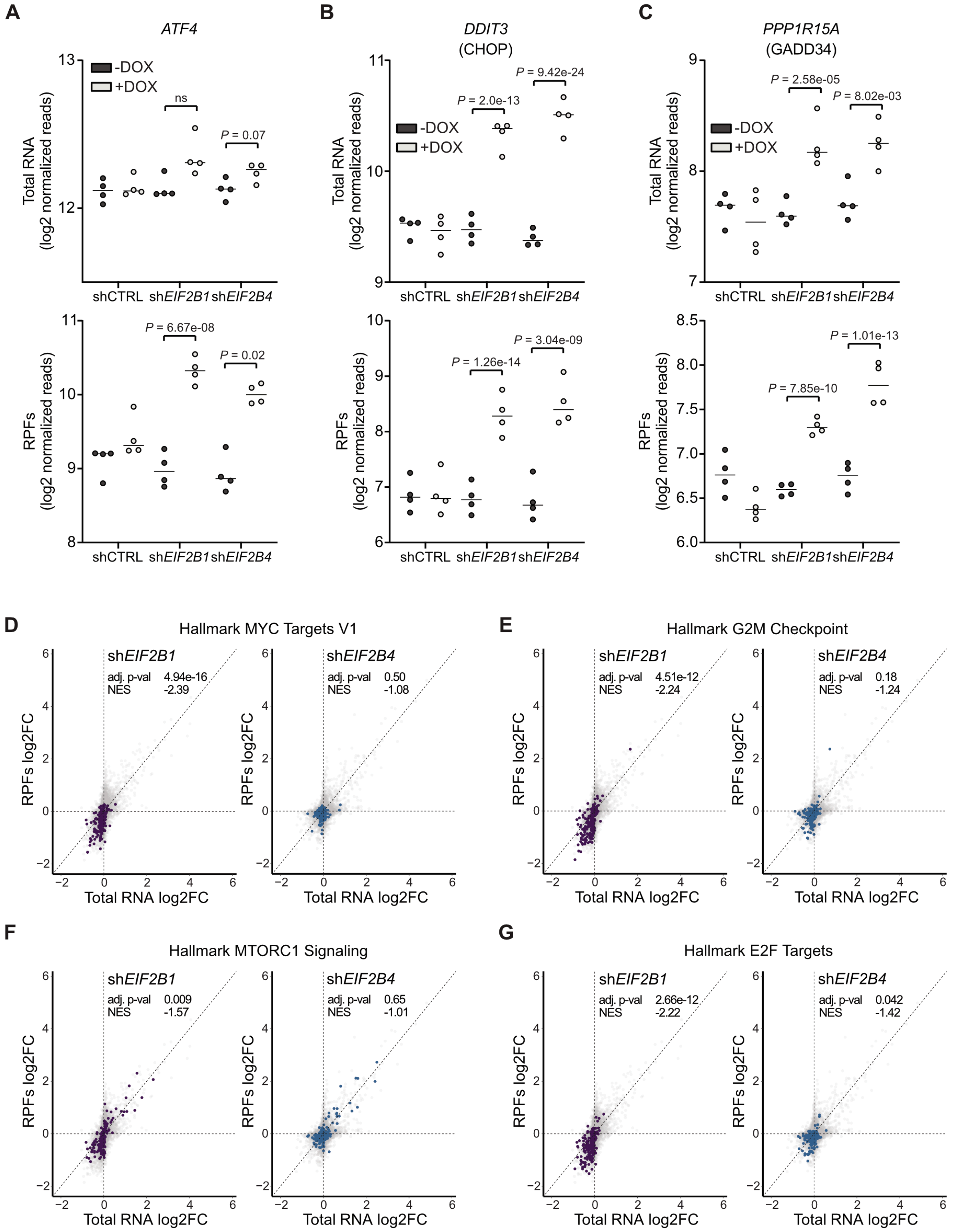

Appendix Figure S6: Translational consequences to eIF2Bα and eIF2Bδ knockdown. (A-C) Log2 read counts for total cytoplasmic RNA (top panels) and RPFs (bottom panels) normalized by regularized logarithm used in the DEseq2 package. All four independent data points are shown. Please note the graphs have different y-axis values. (A) *ATF4*; (B) *DDIT3*, encoding CHOP; (C) *PPP1R15A*, encoding GADD34. Adjusted p-values computed by DESeq2, corrected for shCTRL effects, are displayed in each graph. ns = not significant, adj. p-value >0.1.

(D-G) Pathway-specific scatter plots of log2FC of total cytoplasmic RNA (x-axes) and log2FC of RPFs (y-axes) (as in Fig. 5B). In each panel, colored dots represent the genes belonging to the Hallmarks MYC Targets V1 (D), G2M Checkpoint (E), MTORC1 Signaling (F), or E2F Targets (G). Each plot displays adj. p-values and NES calculated using fGSEA package. Color coding of the dots depends on the subunit being depleted (purple = sh*EIF2B1* scatters, blue = sh*EIF2B4* scatters).

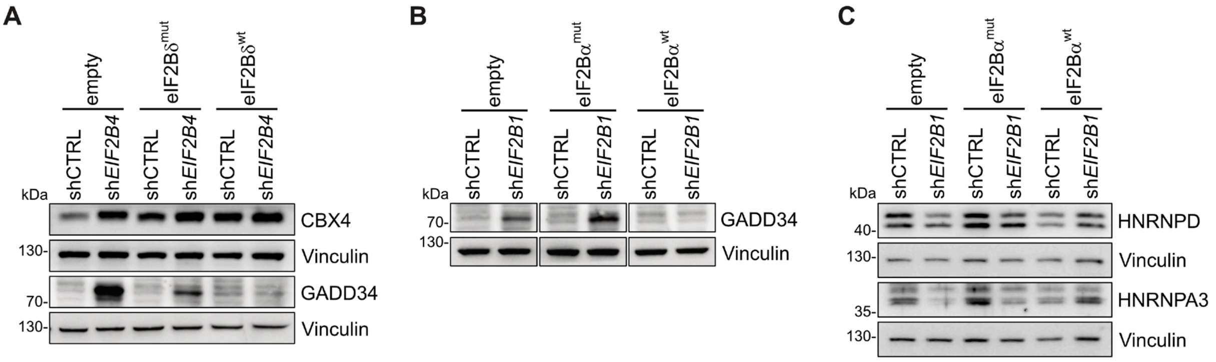

Appendix Figure S7: ISR proteins and HNRNPs are similarly regulated upon eIF2Bα/ eIF2Bδ depletion or mutation.

(A) Western blot of indicated proteins in shCTRL- or sh*EIF2B4*-transduced SW480 cells stably overexpressing eIF2Bδ mutant (eIF2Bδ^{mut}), eIF2Bδ WT (eIF2Bδ^{wt}) construct, or without any overexpression (empty). The western blot is representative of two biological replicates with similar results.

(B) Western blot of indicated proteins in shCTRL- or sh*EIF2B1*-transduced SW480 cells stably overexpressing eIF2Bα mutant (eIF2Bα^{mut}), eIF2Bα WT (eIF2Bα^{wt}) construct, or without any overexpression (empty). The western blot is representative of two biological replicates with similar results.

(C) Western blot of indicated proteins in SW480 cells transduced as described in (B). The western blot is representative of two biological replicates with similar results.

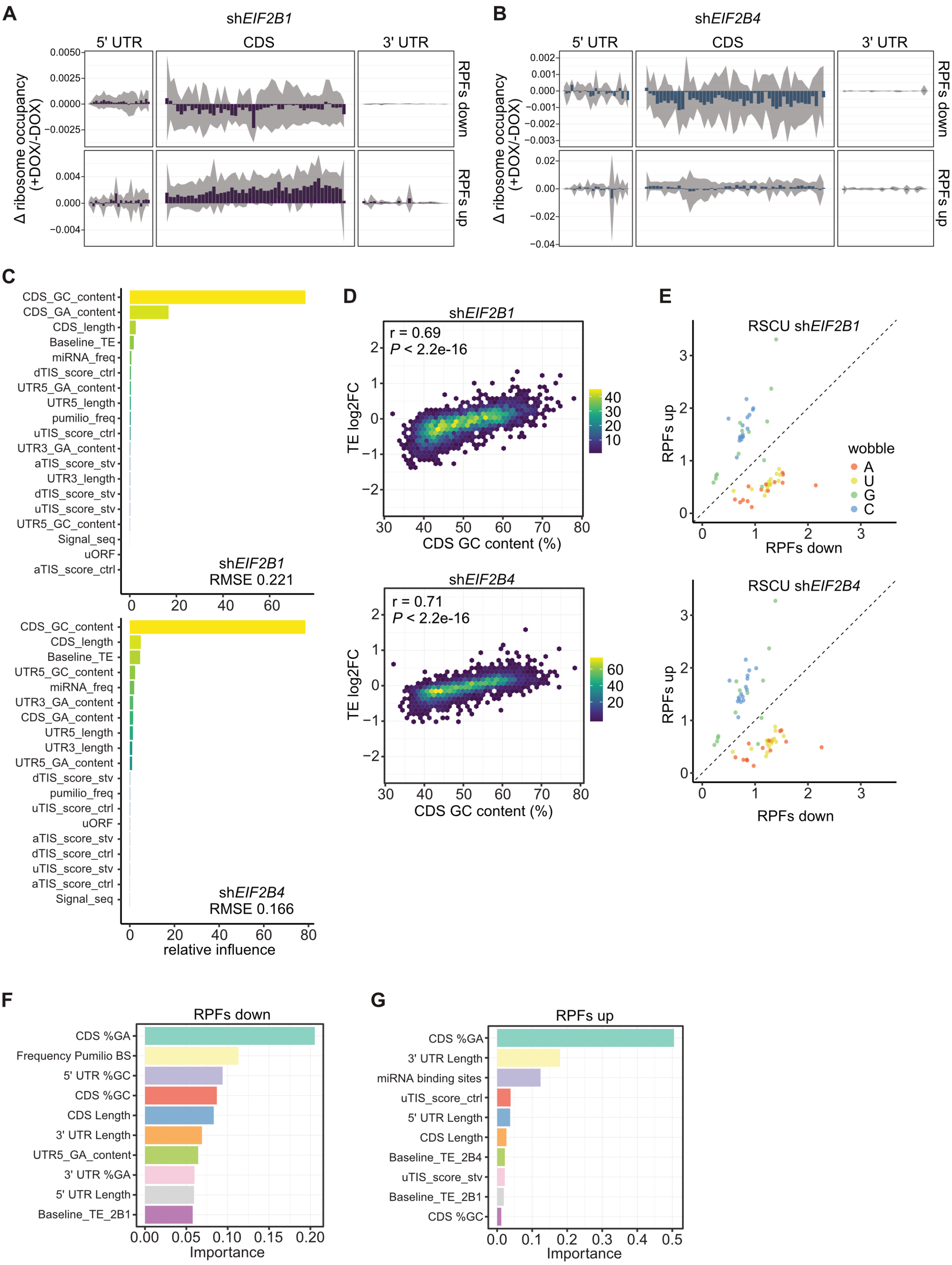

A
shEIF2B1
5' UTR
CDS
3' UTR
RPFs down
RPFs up
Δ ribosome occupancy (+DOX/-DOX)
B
shEIF2B4
C
CDS_GC_content
CDS_GA_content
CDS_length
Baseline_TE
miRNA_freq
dTIS_score_ctrl
UTR5_GA_content
UTR5_length
pumilio_freq
uTIS_score_ctrl
UTR3_GA_content
aTIS_score_stv
UTR3_length
dTIS_score_stv
uTIS_score_stv
UTR5_GC_content
Signal_seq
uORF
aTIS_score_ctrl
shEIF2B1
RMSE 0.221
CDS_GC_content
CDS_length
Baseline_TE
UTR5_GC_content
miRNA_freq
UTR3_GA_content
CDS_GA_content
UTR5_length
UTR3_length
UTR5_GA_content
dTIS_score_stv
pumilio_freq
uTIS_score_ctrl
uORF
aTIS_score_stv
dTIS_score_ctrl
uTIS_score_stv
aTIS_score_ctrl
Signal_seq
shEIF2B4
RMSE 0.166
relative influence
D
shEIF2B1
r = 0.69
P < 2.2e-16
TE log2FC
CDS GC content (%)
shEIF2B4
r = 0.71
P < 2.2e-16
E
RSCU shEIF2B1
RPFs up
RPFs down
wobble
A
U
G
C
RSCU shEIF2B4
F
RPFs down
CDS %GA
Frequency Pumilio BS
5' UTR %GC
CDS %GC
CDS Length
3' UTR Length
UTR5_GA_content
3' UTR %GA
5' UTR Length
Baseline_TE_2B1
Importance
G
RPFs up
CDS %GA
3' UTR Length
miRNA binding sites
uTIS_score_ctrl
5' UTR Length
CDS Length
Baseline_TE_2B4
uTIS_score_stv
Baseline_TE_2B1
CDS %GC
Importance

Appendix Figure S8: Translation regulation by knockdown of eIF2Bα and eIF2Bδ depends on GA content of the coding sequences and codon usage.

(A) Metagene plots representing the variation in ribosomal density for transcripts changing at the RPF levels upon induction of sh*EIF2B1*. Groups are defined as in Fig. 5B. Each portion of transcripts is binned for uniform representation (5’ and 3’ untranslated regions – 5’ UTR/3’ UTR = 25 bins; coding sequence – CDS = 50 bins).

(B) Metagene plots as described in (A) for sh*EIF2B4*.

(C) Relative influence on TE log2FC of selected molecular features of transcripts upon sh*EIF2B1* (top) or sh*EIF2B4* (bottom) induction (shCTRL corrected) as determined by gradient boosting analysis. Root Mean Square Errors (RMSE) are indicated, estimating how much data remains unexplained by the respective models.

(D) Scatter plots of percentage of GC content of CDSs (x-axis) and TE log2FC (y-axis) upon sh*EIF2B1* (top) or sh*EIF2B4* (bottom) induction. Correlation r value and p-value are indicated. Data points are binned and density per bin is represented (40 bins per axis), color represents the number of transcripts per bin as per legend.

(E) Relative Synonymous Codon Usage (RSCU) of transcripts changing at the RPF level upon induction of sh*EIF2B1* (top) or sh*EIF2B4* (bottom). Groups are defined as in (A). The third nucleotide (wobble) of each codon is color coded as per legend.

(F,G) Relative importance (determined by gradient boosting analysis) of molecular features in classifying transcripts that are downregulated (F) or upregulated (G) by sh*EIF2B1* compared to sh*EIF2B4*.

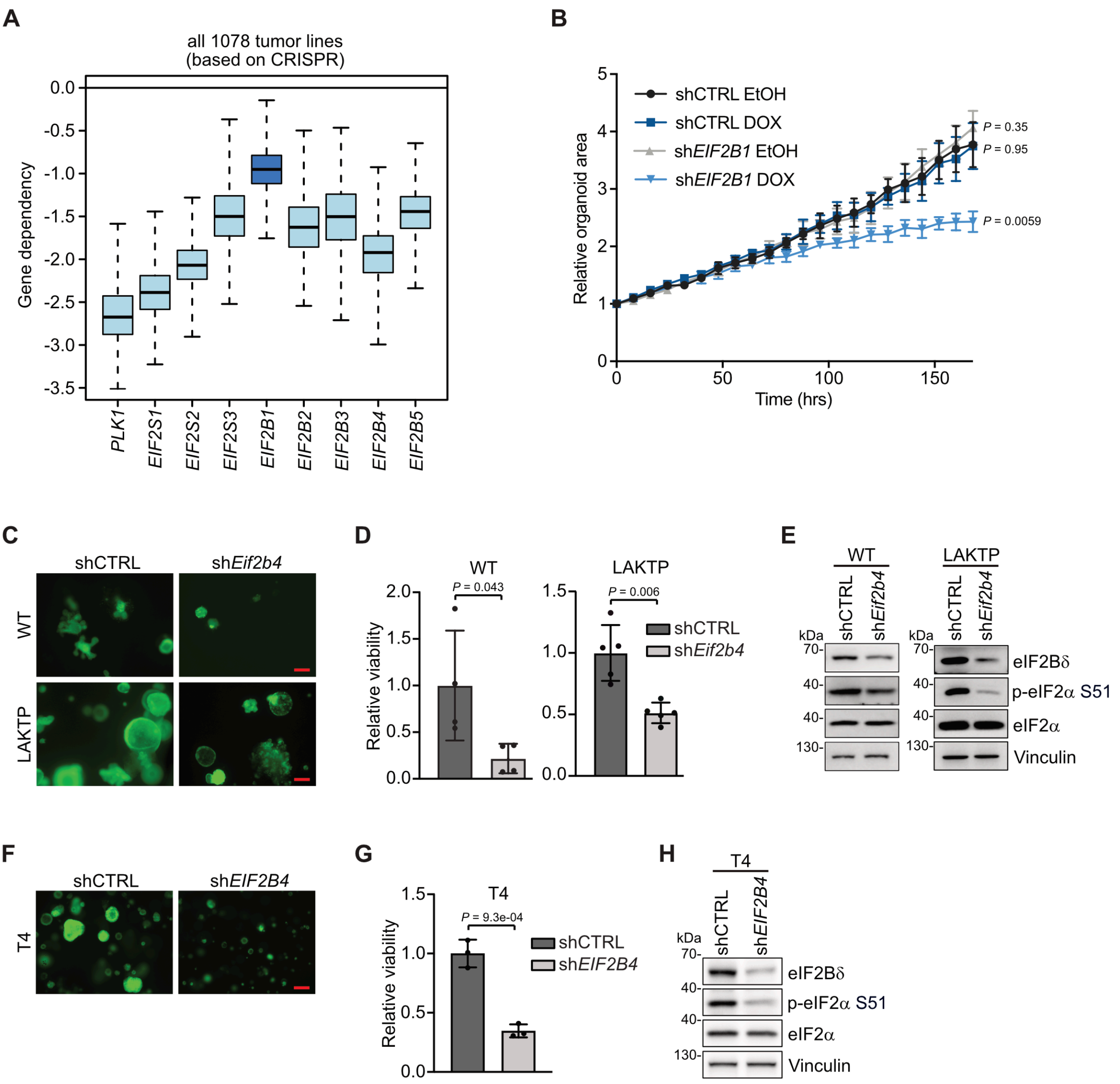

Appendix Figure S9: eIF2Bα is the least essential subunit compared to other eIF2B and eIF2 subunits.

(A) Box plot showing gene dependency scores from the DepMap database for *PLK1*, eIF2 subunits (*EIF2S1-3*) and eIF2B subunits (*EIF2B1-5*).

(B) Growth of PDO HD-3 transduced with doxycycline-inducible shCTRL or sh*EIF2B1-1* analyzed via Incucyte® live imaging over seven days of DOX treatment (EtOH as control). Data show mean ± s.d. (*n* = 3 biological replicates); Student's *t*-test.

(C) Pictures of murine WT and LAKTP intestinal organoids transduced with doxycycline-inducible shCTRL or murine sh*Eif2b4* (seven days of doxycycline treatment), representative of four or five biological replicates with similar results. Green signal (GFP) indicates shRNA induction. Scale bar = 200 µm. Picture of LAKTP shCTRL is the same as shown in Fig. EV5A. Experiments in LAKTP organoids were done in parallel and shCTRL served as control condition for both sh*Eif2b1-2* and sh*Eif2b4*.

(D) Relative viability of WT and LAKTP organoids transduced and treated as described in (C). Quantification of LAKTP shCTRL is the same as shown in Fig. EV5B. Experiments in LAKTP organoids were done in parallel and shCTRL served as control condition for both sh*Eif2b1-2* and sh*Eif2b4*. Data show mean ± s.d. (*n* = 4 or 5 biological replicates); Student's *t*-test.

(E) Western blot of indicated proteins in WT and LAKTP organoids transduced as described in (C), representative of three biological replicates with similar results (96 hrs of doxycycline treatment).
(F) Pictures of T4 PDOs transduced with doxycycline-inducible shCTRL or human sh*EIF2B4* (seven days of doxycycline treatment), representative of three biological replicates with similar results. Green signal (GFP) indicates shRNA induction. Scale bar = 200 µm.
(G) Relative viability of T4 PDOs transduced and treated as described in (F). Data show mean ± s.d. (n = 3 biological replicates); Student's t-test.
(H) Western blot of indicated proteins in T4 PDOs transduced as described in (F), representative of three biological replicates with similar results (96 hrs of doxycycline treatment).

Appendix Table S1: Results of panelseq for PDO HD-3.

PDO	Gene	Coding	Amino acid change	Frequency (%)
HD-3	*PIK3CA*	c.971C>T	p.Thr324Ile	32.98
	APC	c.3944C>A	p.Ser1315Ter	70.68
	TP53	c.473G>T	p.Arg158Leu	100.00
