# Supplementary figures and images for: Maintenance of p-eIF2α levels by the eIF2B complex is vital for colorectal cancer (part 1 of 2)
Source: EMBO J. 2025 Feb 27;44(7):2075–105. doi: 10.1038/s44318-025-00381-9 (PMC11962125; doi:10.1038/s44318-025-00381-9)

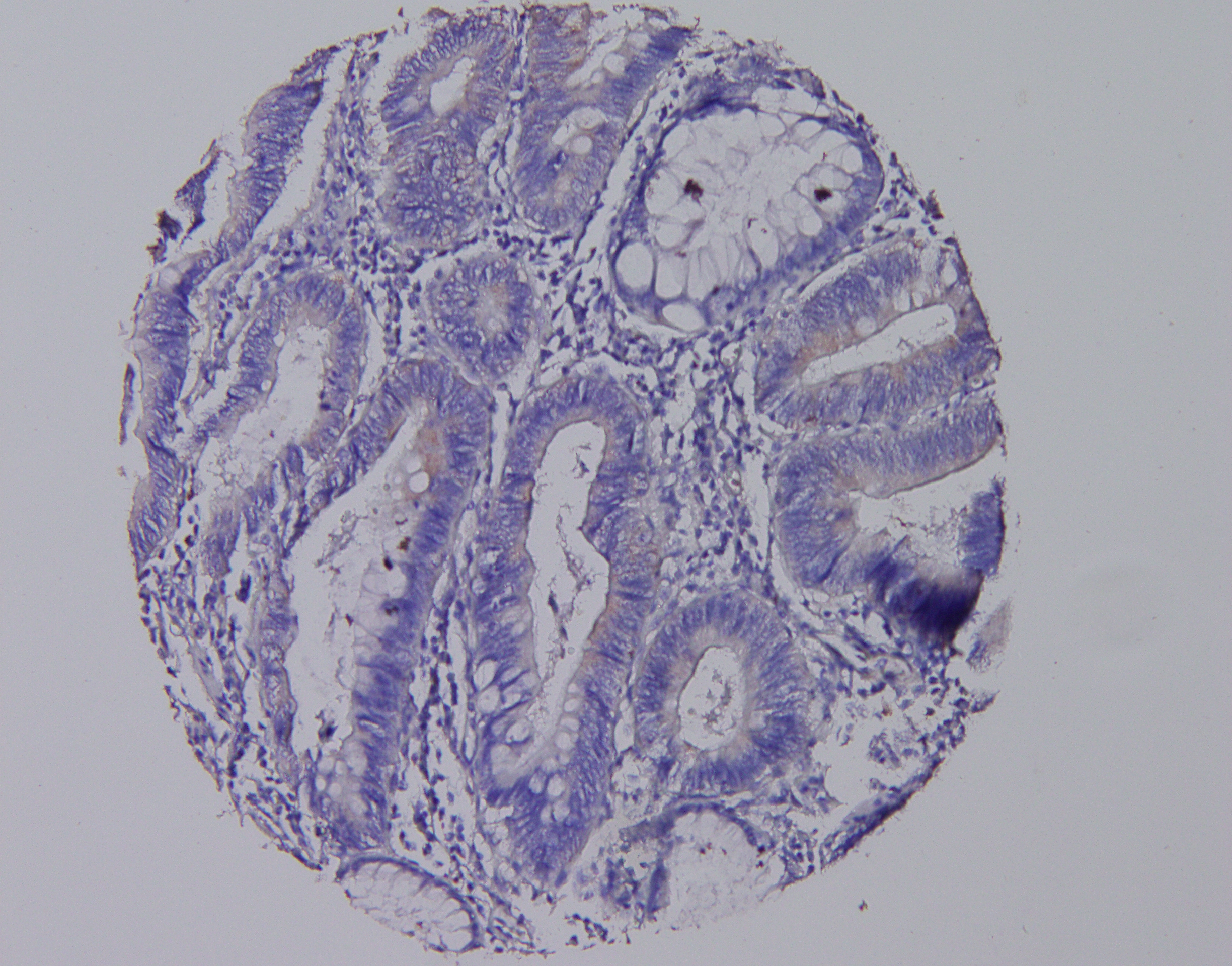

Supplement: Supplementary file 7 — Source data Fig. 1 [file 44318_2025_381_MOESM7_ESM.zip › Figure 1/1A/Adenoma.jpg]

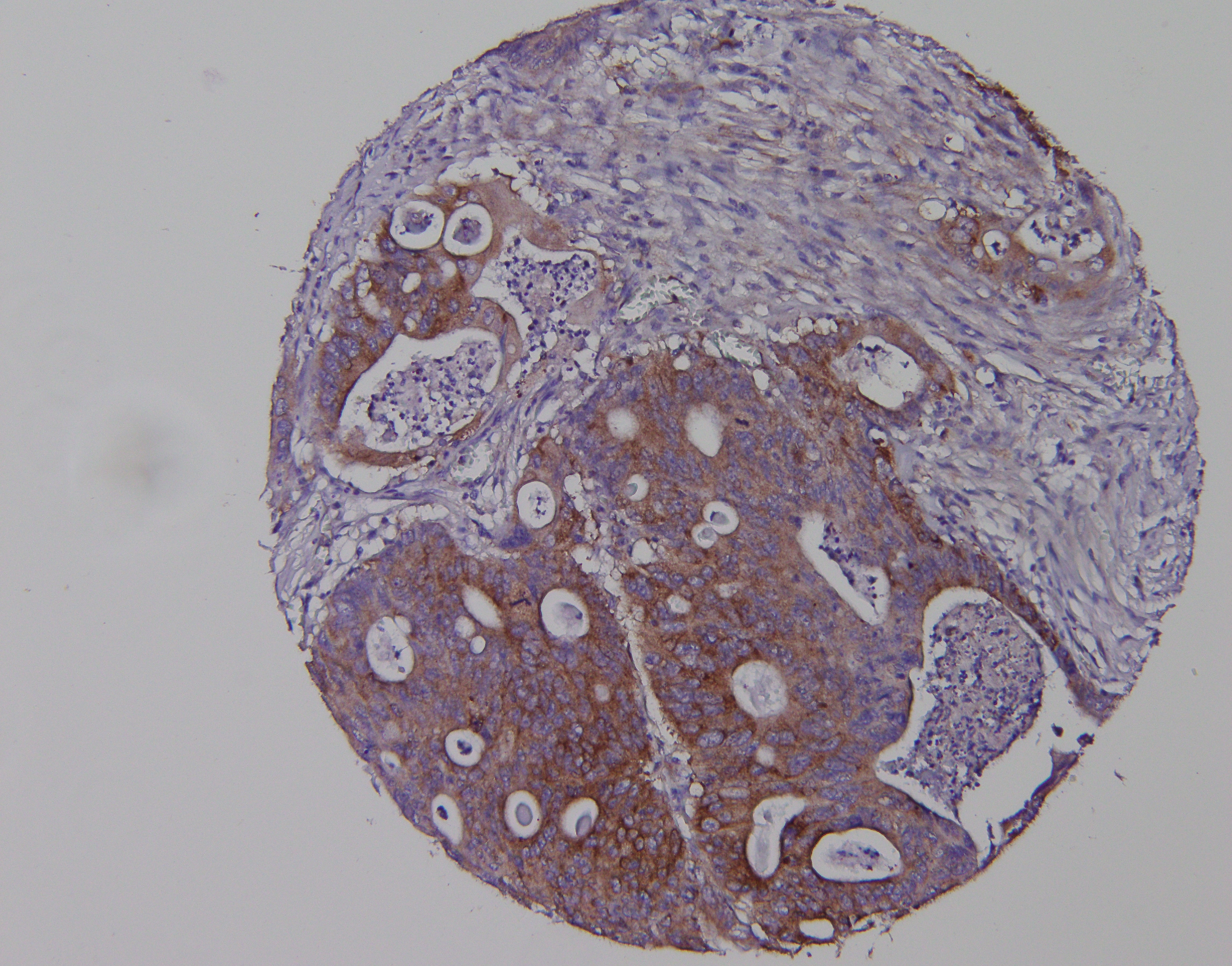

Supplement: Supplementary file 7 — Source data Fig. 1 [file 44318_2025_381_MOESM7_ESM.zip › Figure 1/1A/Colon carcinoma2.jpg]

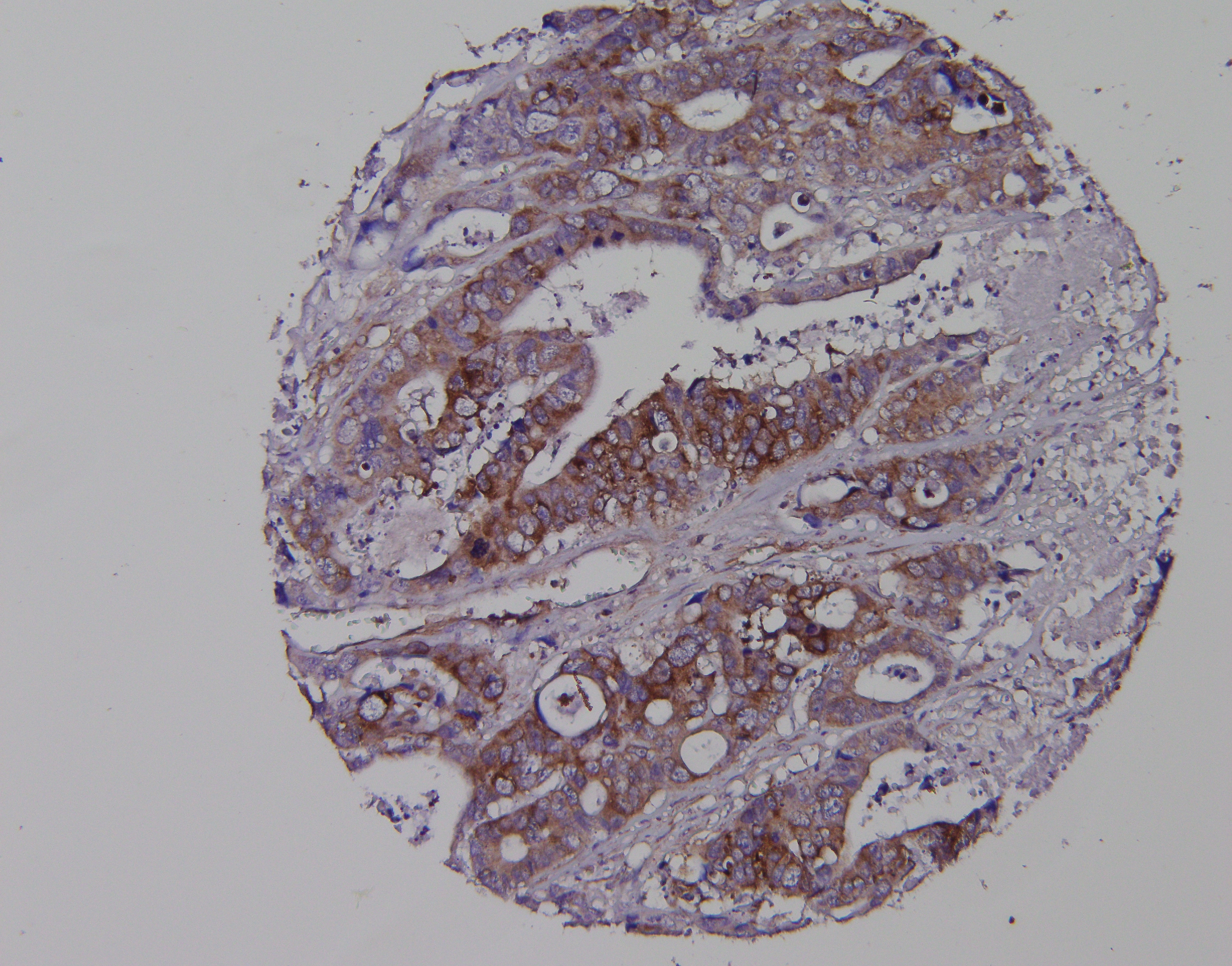

Supplement: Supplementary file 7 — Source data Fig. 1 [file 44318_2025_381_MOESM7_ESM.zip › Figure 1/1A/Colon carcinoma1.jpg]

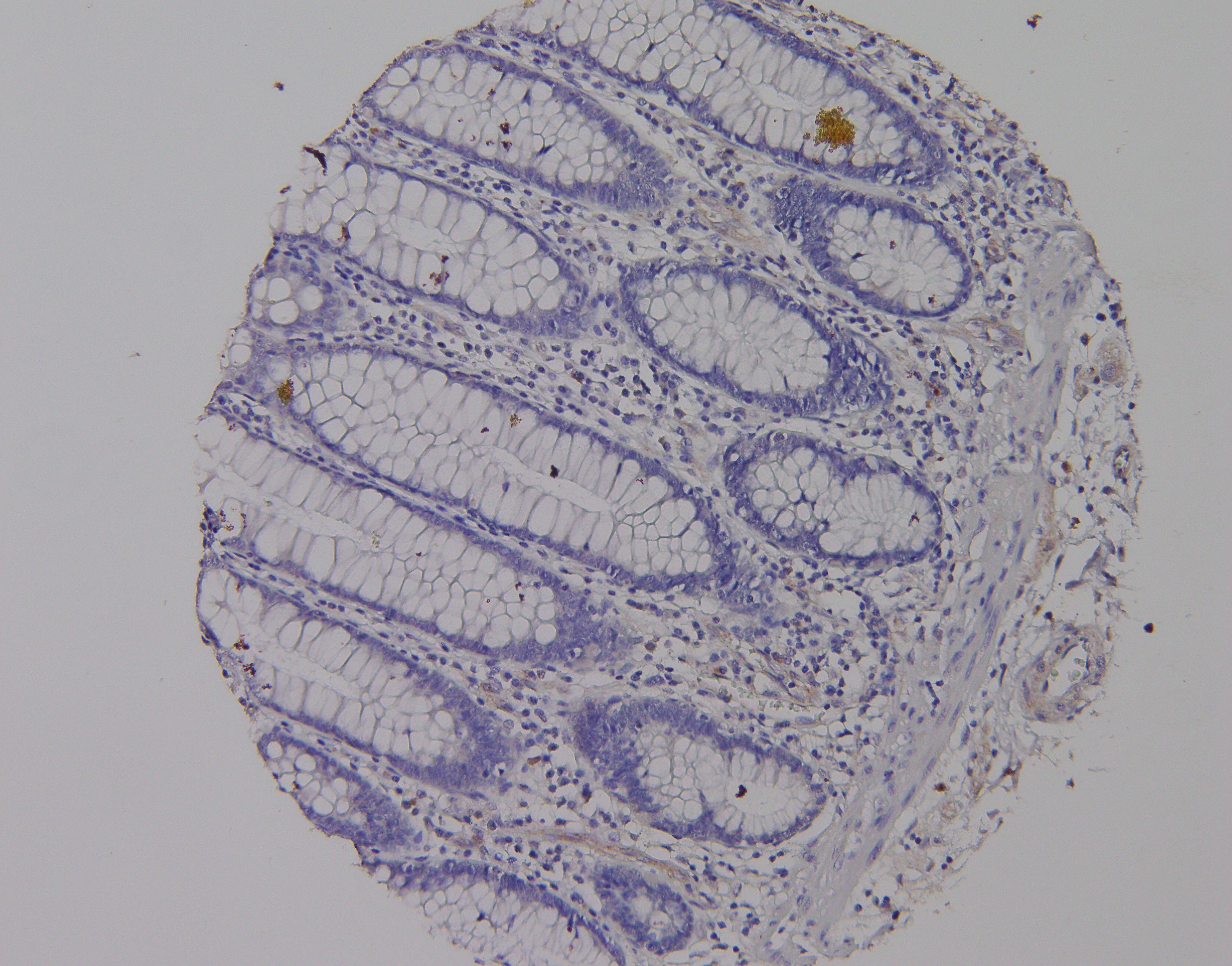

Supplement: Supplementary file 7 — Source data Fig. 1 [file 44318_2025_381_MOESM7_ESM.zip › Figure 1/1A/Colon mucosa.jpg]

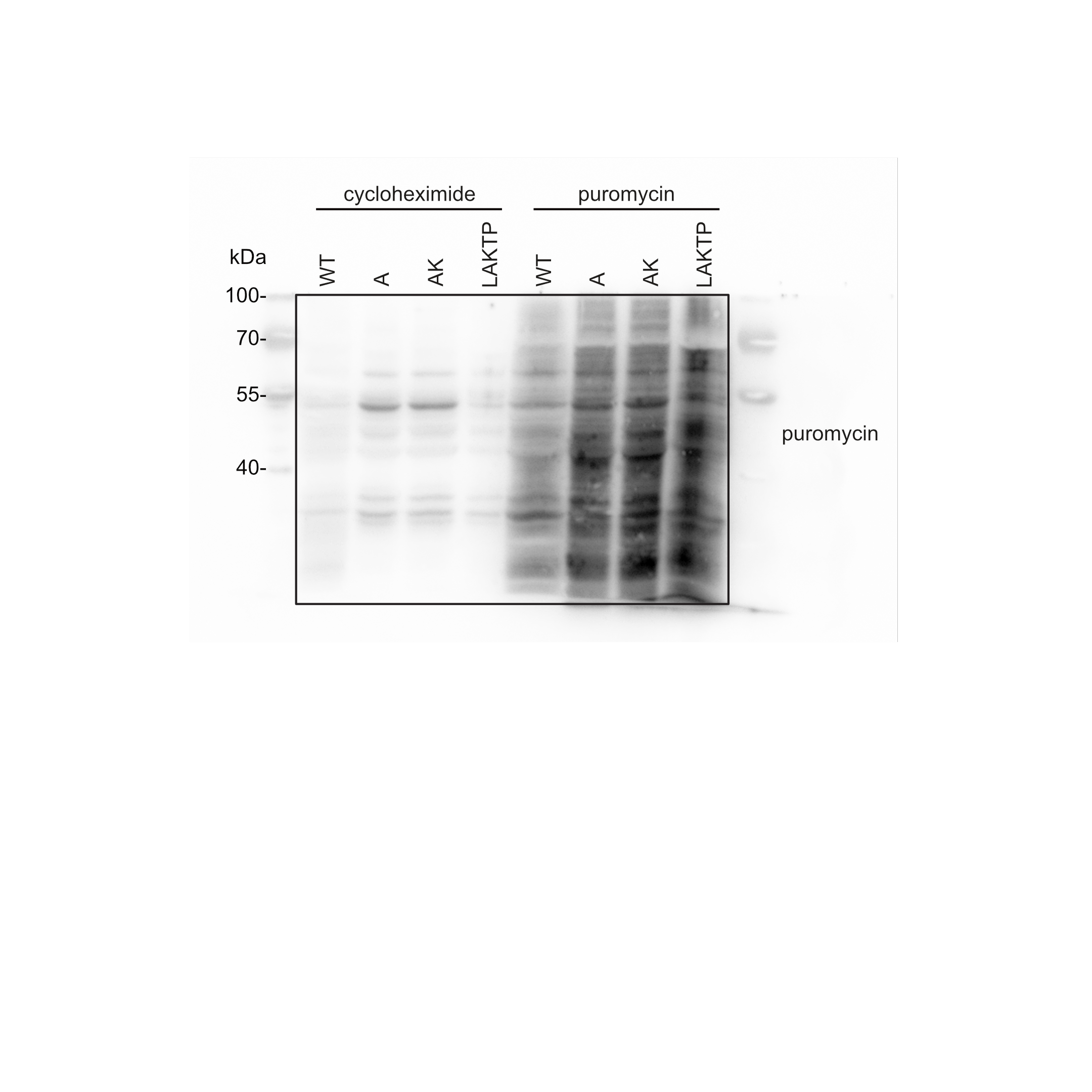

Supplement: Supplementary file 7 — Source data Fig. 1 [file 44318_2025_381_MOESM7_ESM.zip › Figure 1/1D/western puromycin.Tif]

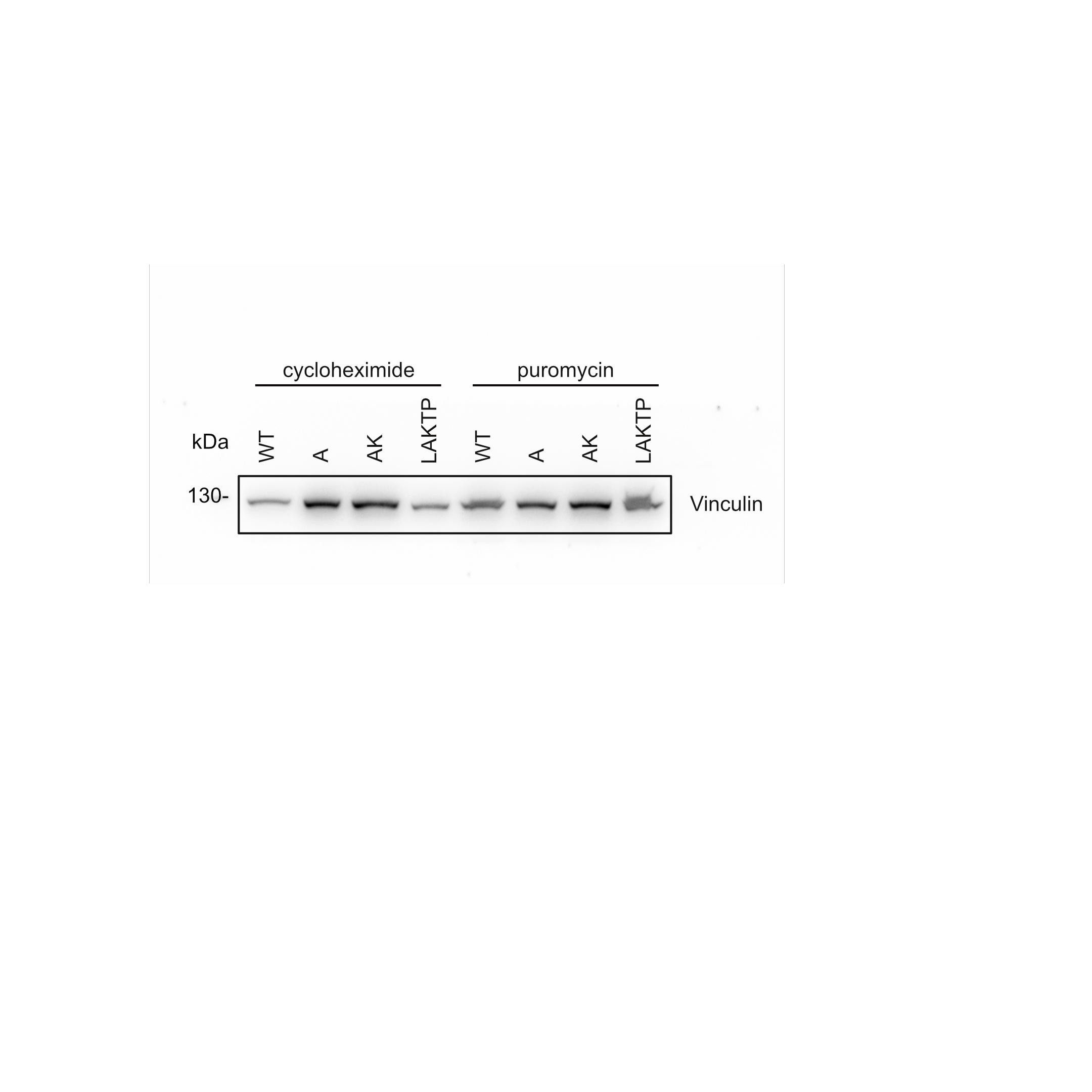

Supplement: Supplementary file 7 — Source data Fig. 1 [file 44318_2025_381_MOESM7_ESM.zip › Figure 1/1D/western vinculin.tiff]

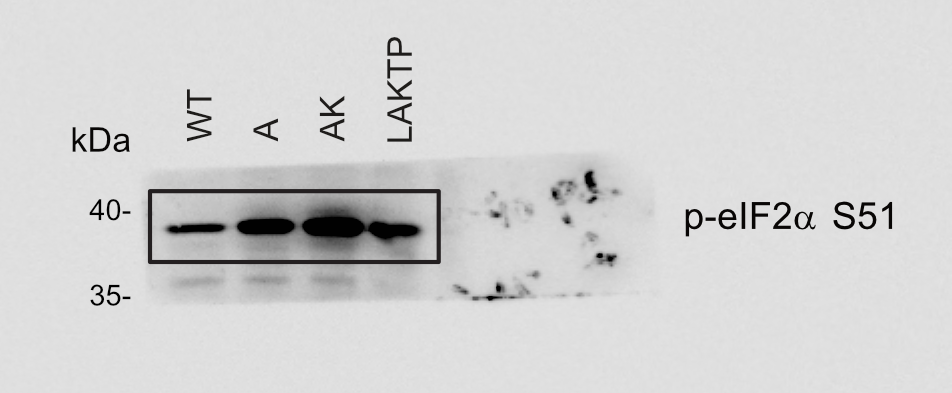

Supplement: Supplementary file 7 — Source data Fig. 1 [file 44318_2025_381_MOESM7_ESM.zip › Figure 1/1C/western p-eif2a S51.tif]

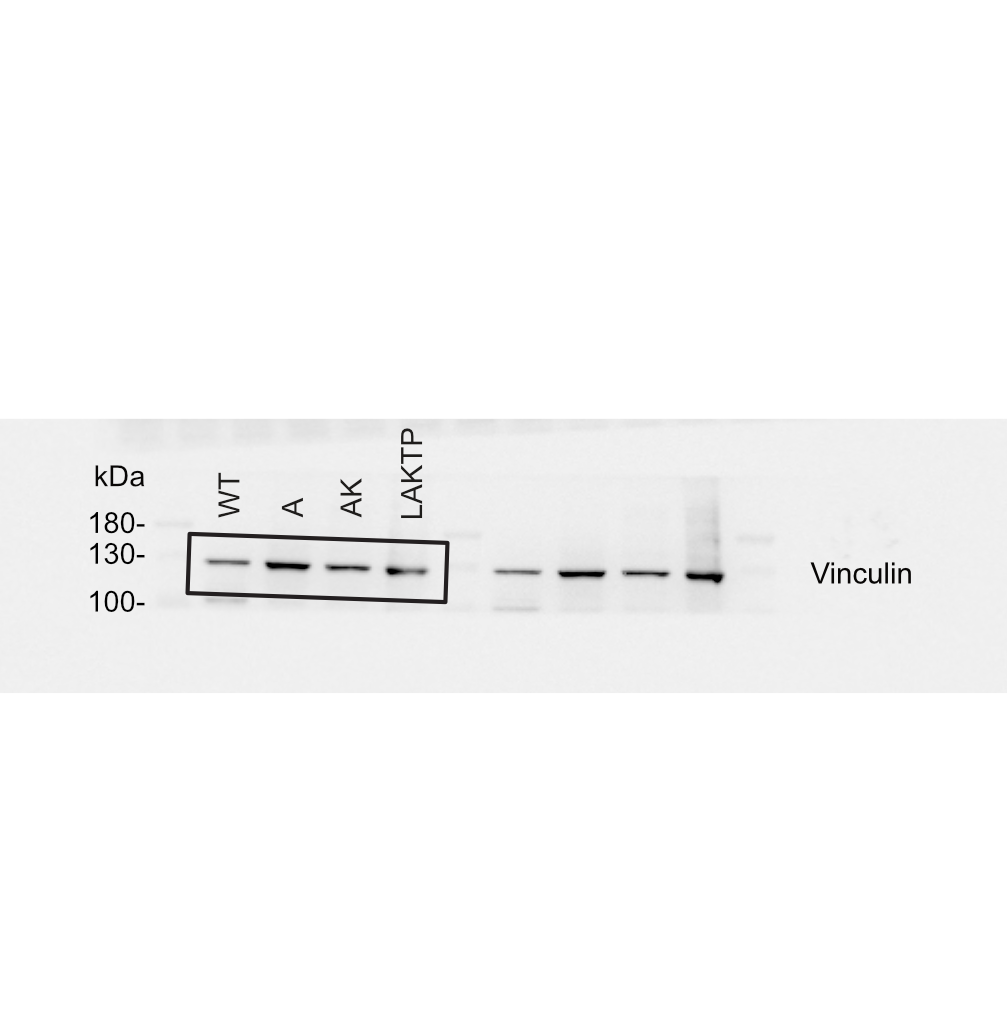

Supplement: Supplementary file 7 — Source data Fig. 1 [file 44318_2025_381_MOESM7_ESM.zip › Figure 1/1C/western vinculin.tif]

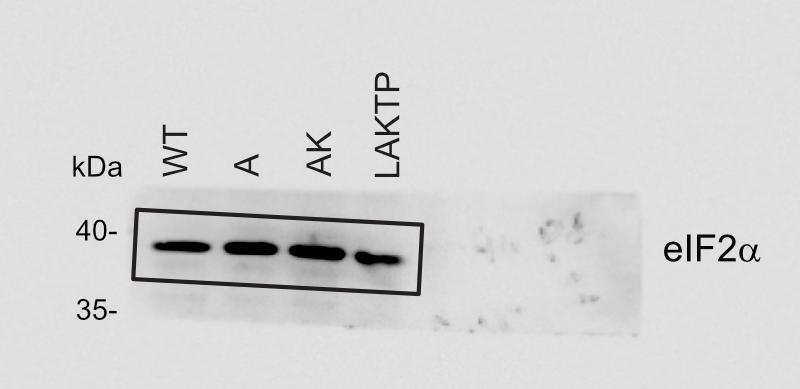

Supplement: Supplementary file 7 — Source data Fig. 1 [file 44318_2025_381_MOESM7_ESM.zip › Figure 1/1C/western eIF2a.tif]

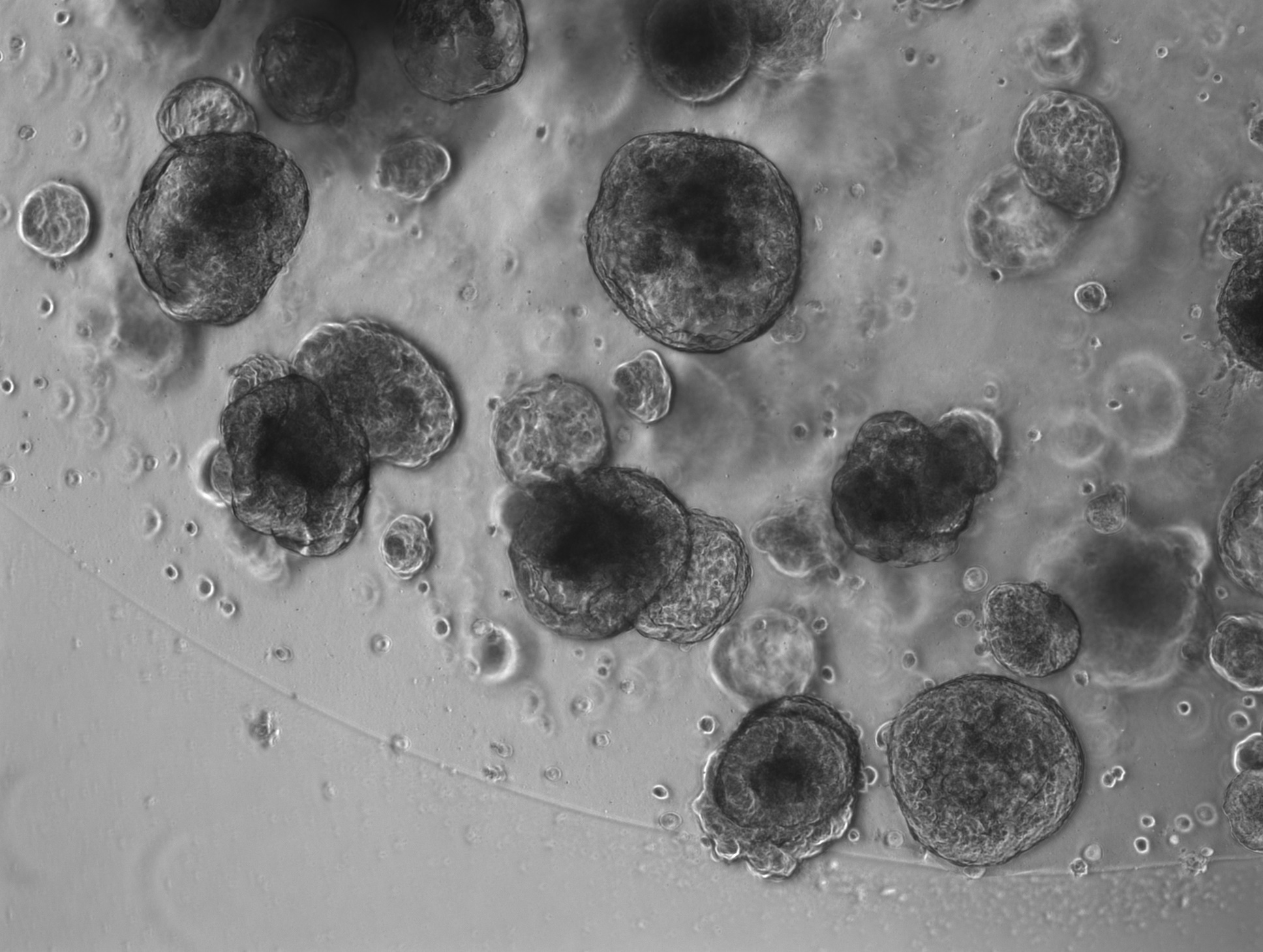

Supplement: Supplementary file 7 — Source data Fig. 1 [file 44318_2025_381_MOESM7_ESM.zip › Figure 1/1F/LAKTP/LAKTP_200nM.jpg]

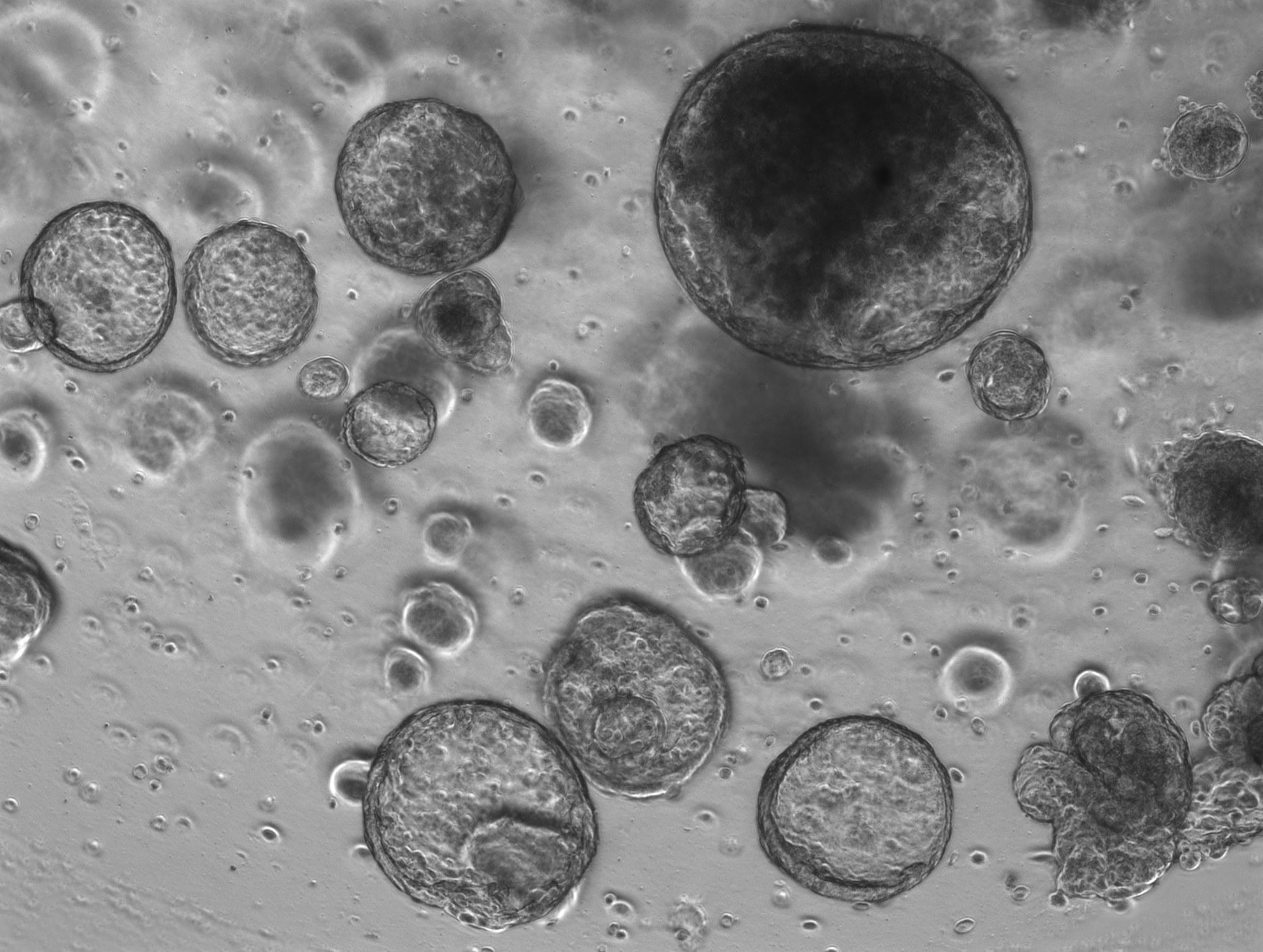

Supplement: Supplementary file 7 — Source data Fig. 1 [file 44318_2025_381_MOESM7_ESM.zip › Figure 1/1F/LAKTP/LAKTP_1000nM.jpg]

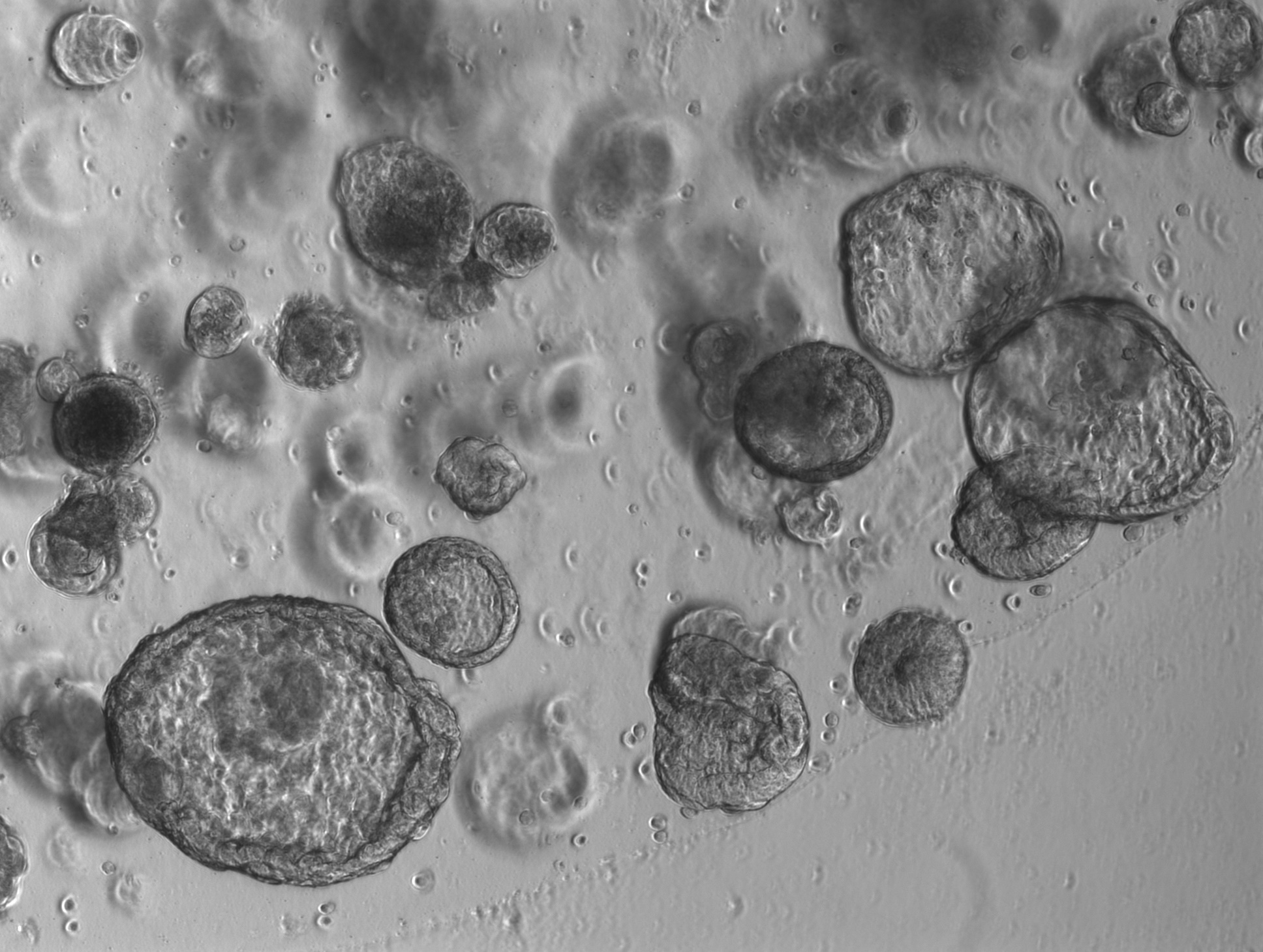

Supplement: Supplementary file 7 — Source data Fig. 1 [file 44318_2025_381_MOESM7_ESM.zip › Figure 1/1F/LAKTP/LAKTP_500nM.jpg]

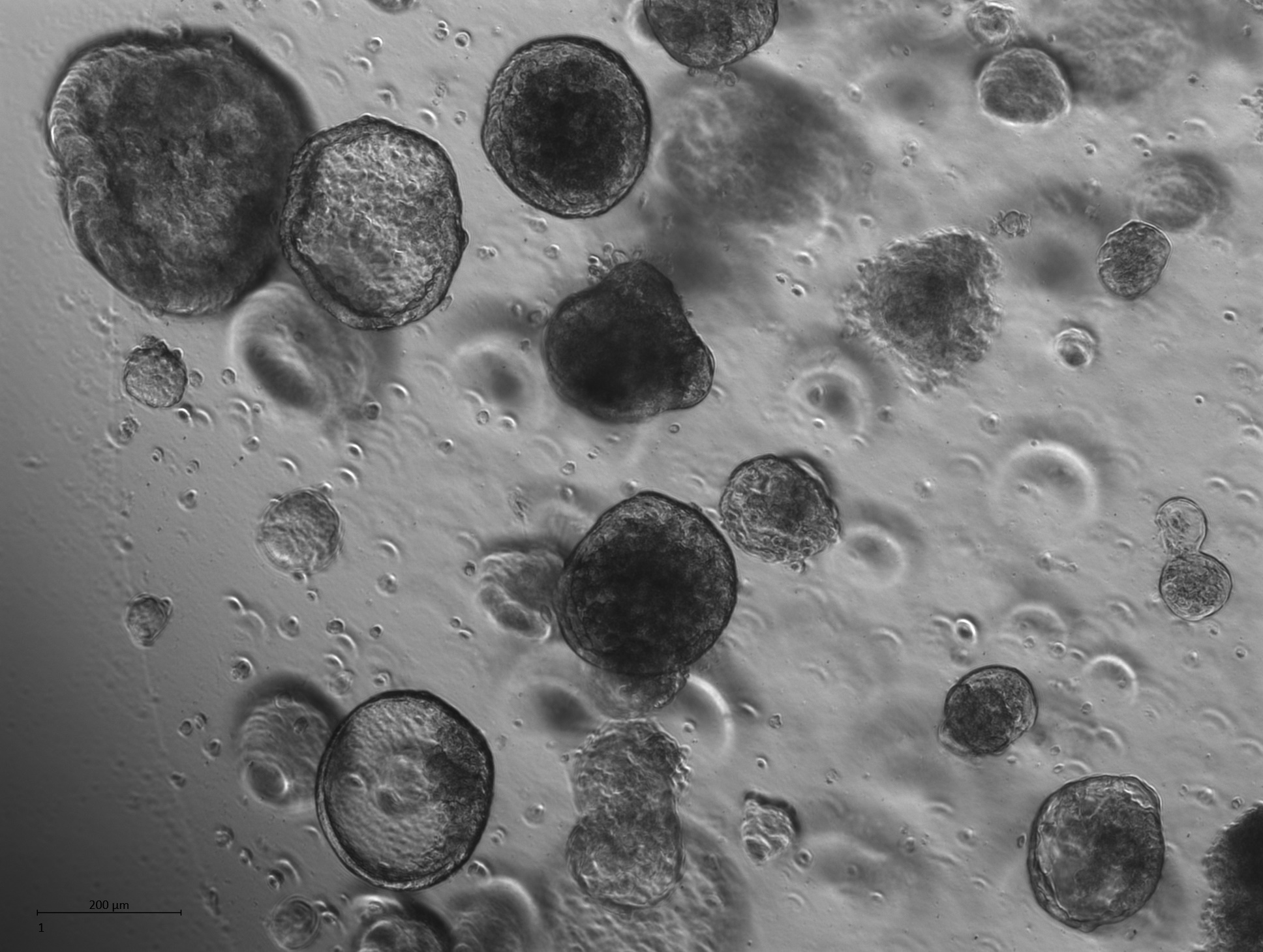

Supplement: Supplementary file 7 — Source data Fig. 1 [file 44318_2025_381_MOESM7_ESM.zip › Figure 1/1F/LAKTP/LAKTP_DMSO.jpg]

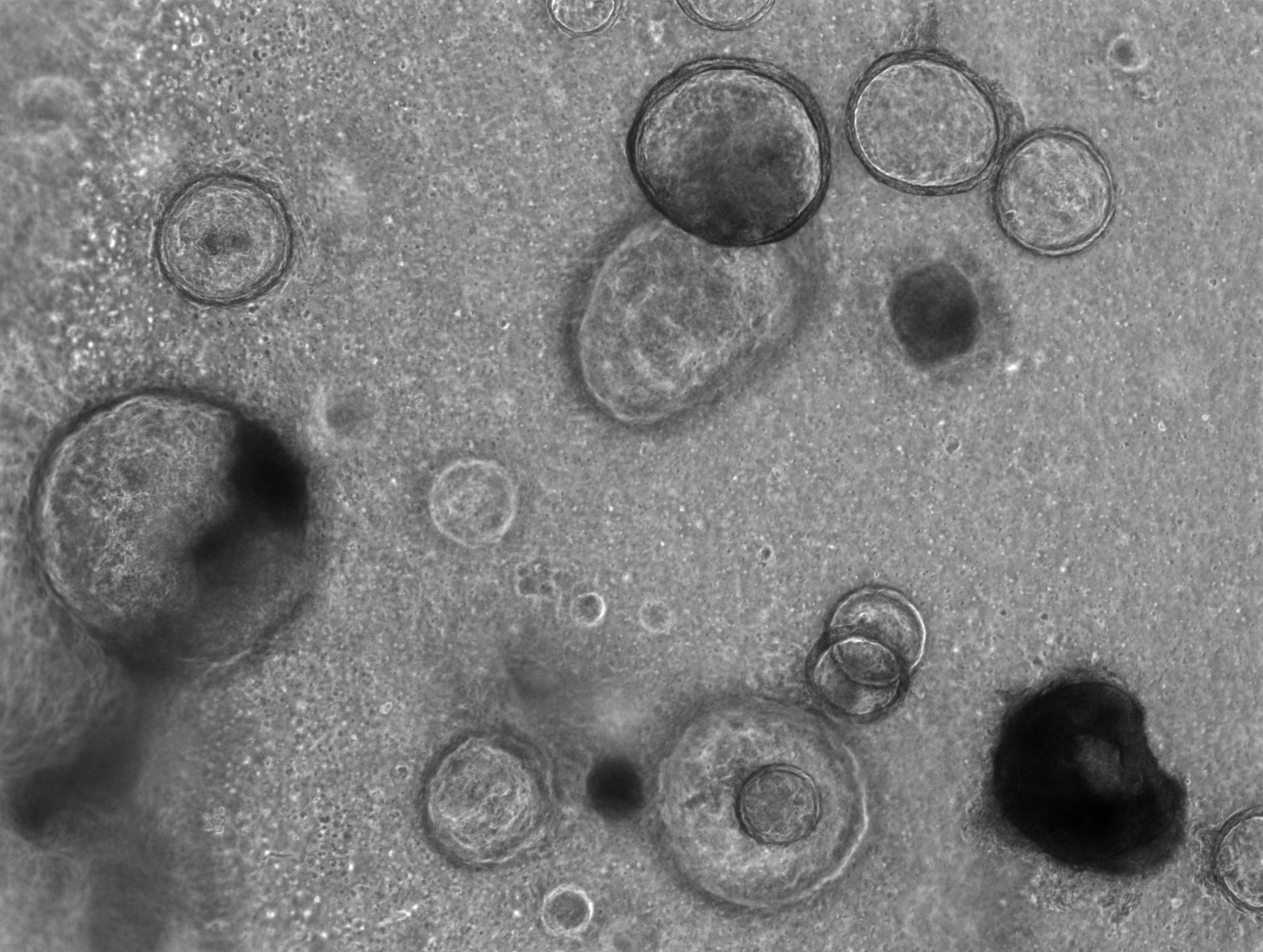

Supplement: Supplementary file 7 — Source data Fig. 1 [file 44318_2025_381_MOESM7_ESM.zip › Figure 1/1F/APC/A_DMSO.png]

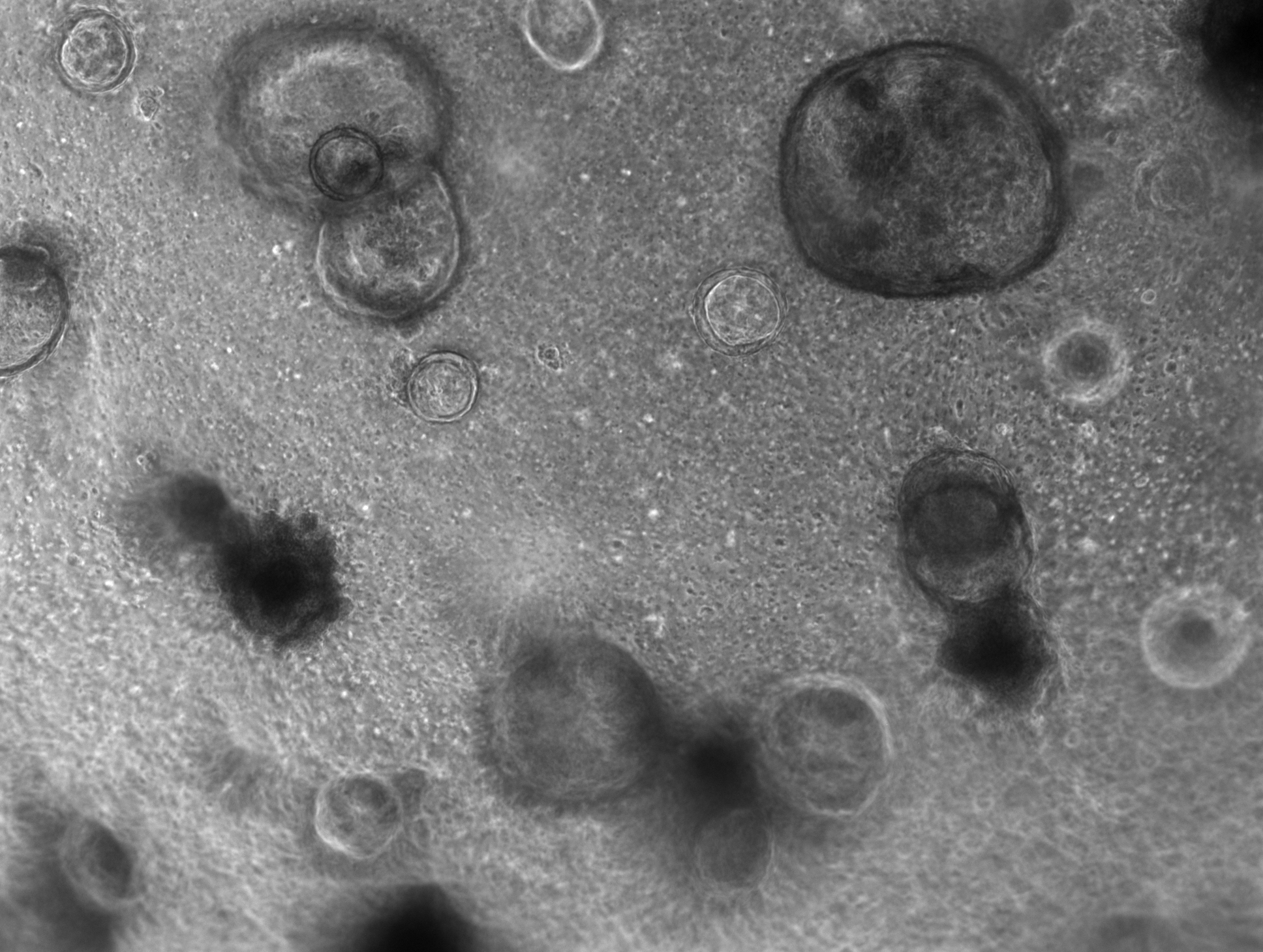

Supplement: Supplementary file 7 — Source data Fig. 1 [file 44318_2025_381_MOESM7_ESM.zip › Figure 1/1F/APC/A_500nM.png]

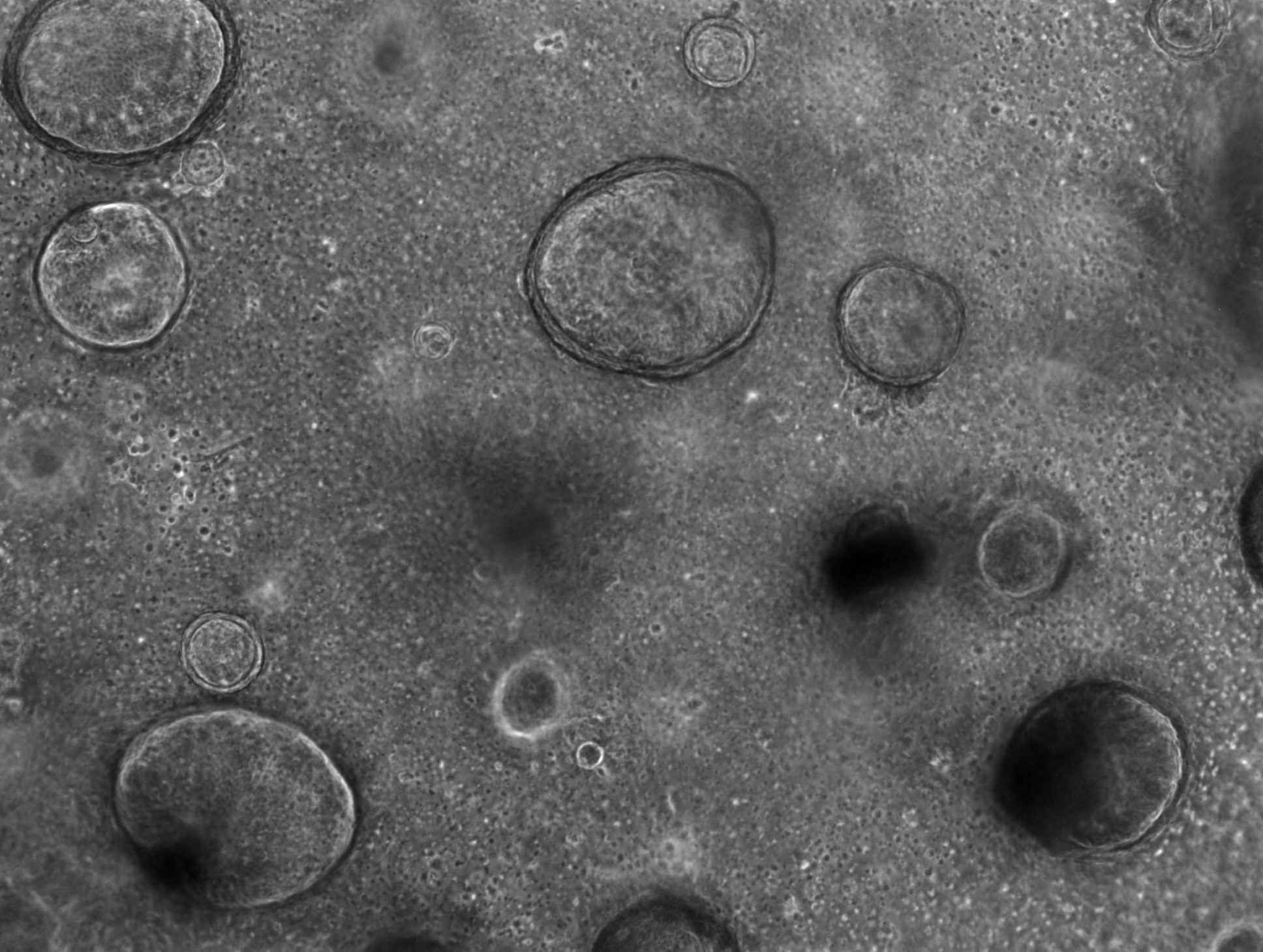

Supplement: Supplementary file 7 — Source data Fig. 1 [file 44318_2025_381_MOESM7_ESM.zip › Figure 1/1F/APC/A_200nM.png]

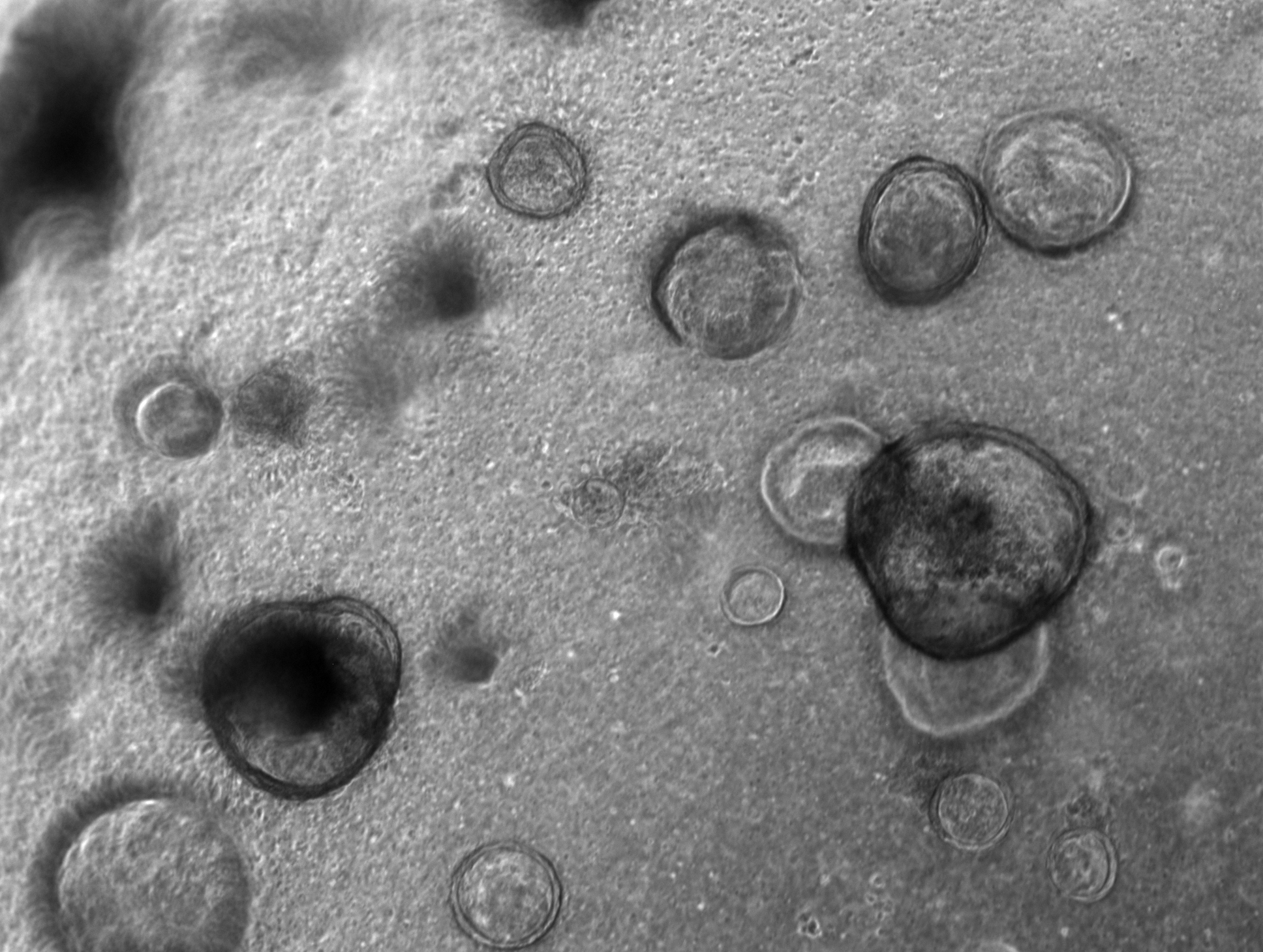

Supplement: Supplementary file 7 — Source data Fig. 1 [file 44318_2025_381_MOESM7_ESM.zip › Figure 1/1F/APC/A_1000nM.png]

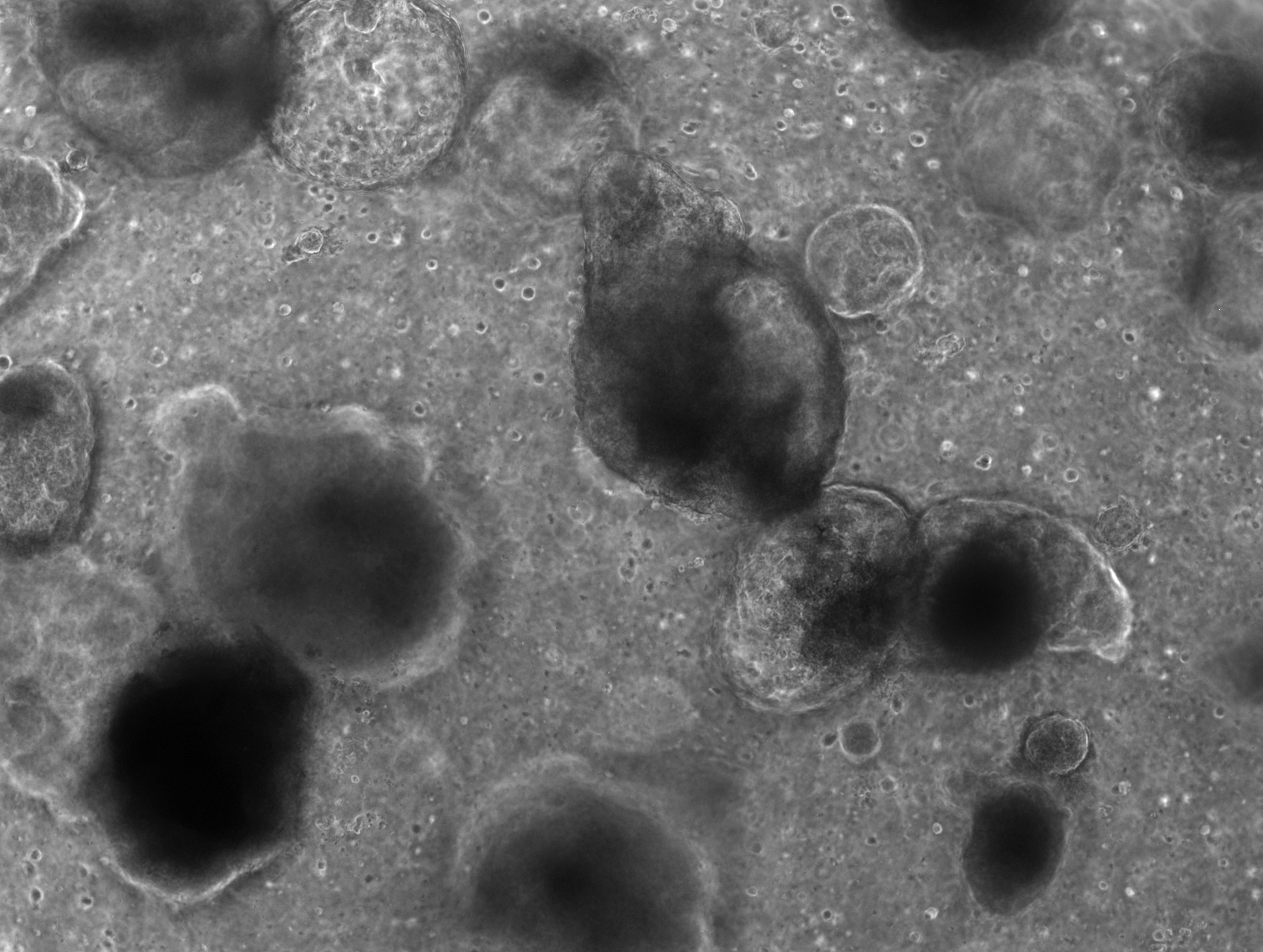

Supplement: Supplementary file 7 — Source data Fig. 1 [file 44318_2025_381_MOESM7_ESM.zip › Figure 1/1F/AK/AK_500nM.png]

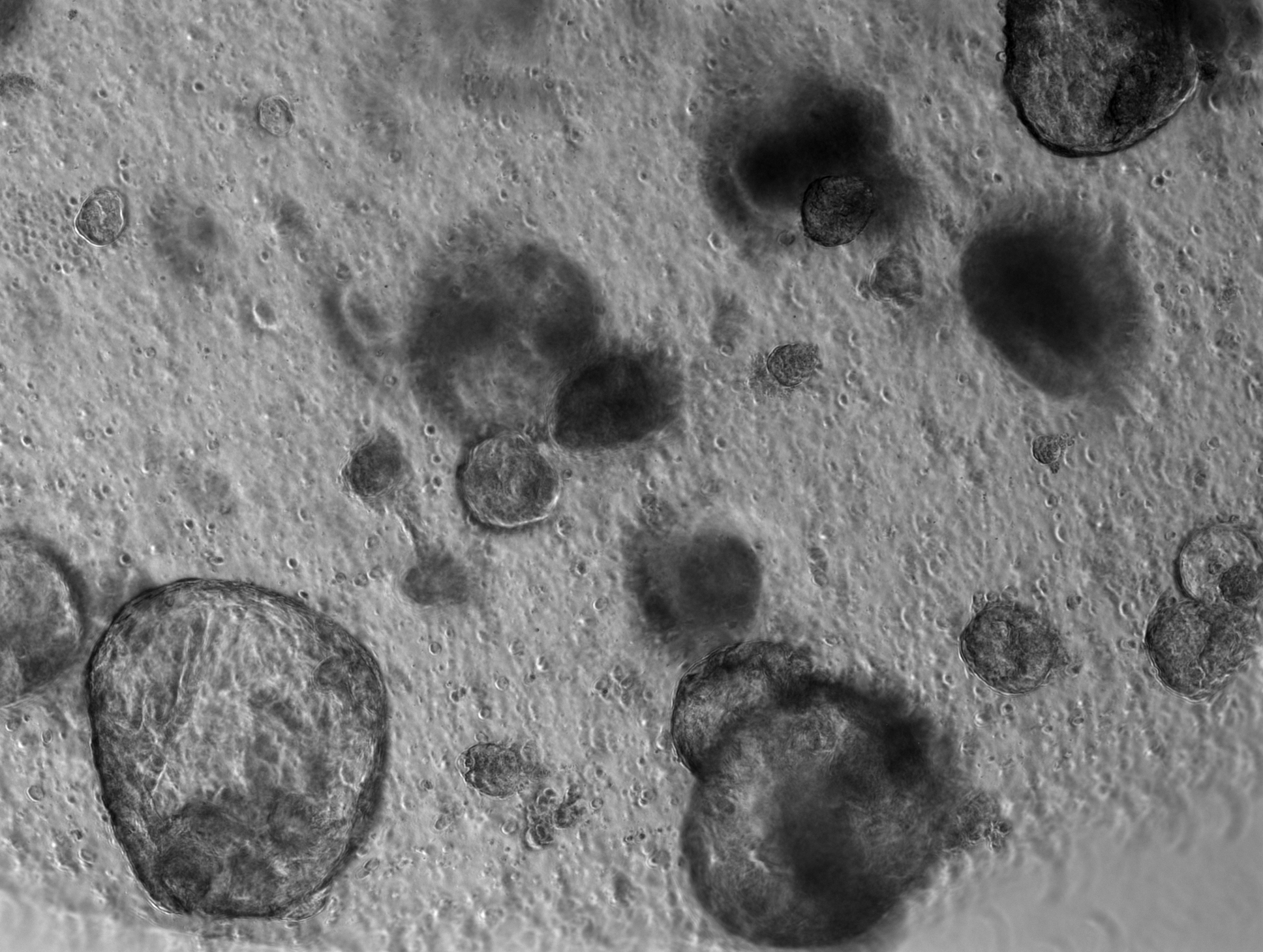

Supplement: Supplementary file 7 — Source data Fig. 1 [file 44318_2025_381_MOESM7_ESM.zip › Figure 1/1F/AK/AK_200nM.png]

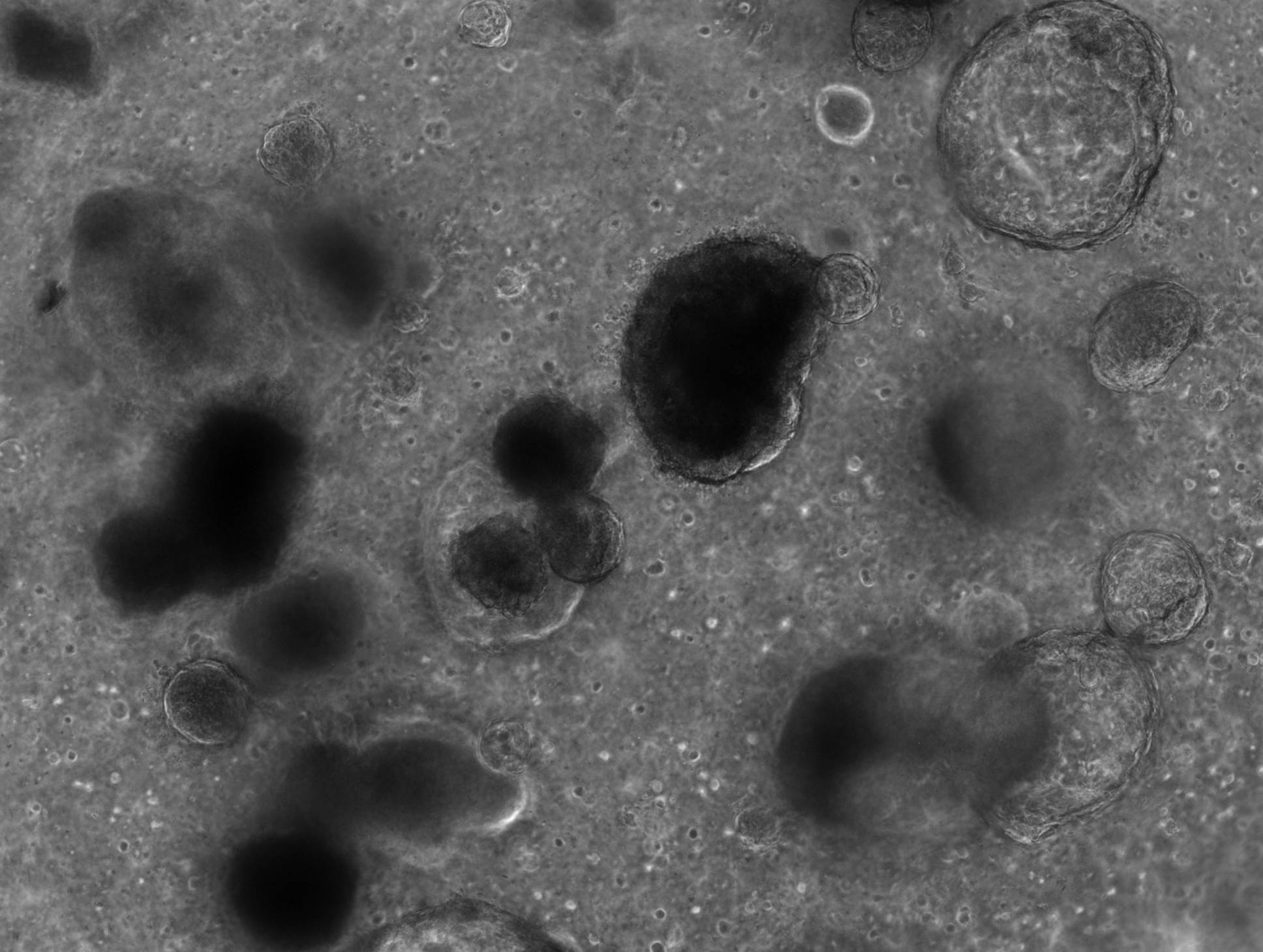

Supplement: Supplementary file 7 — Source data Fig. 1 [file 44318_2025_381_MOESM7_ESM.zip › Figure 1/1F/AK/AK_1000nM.png]

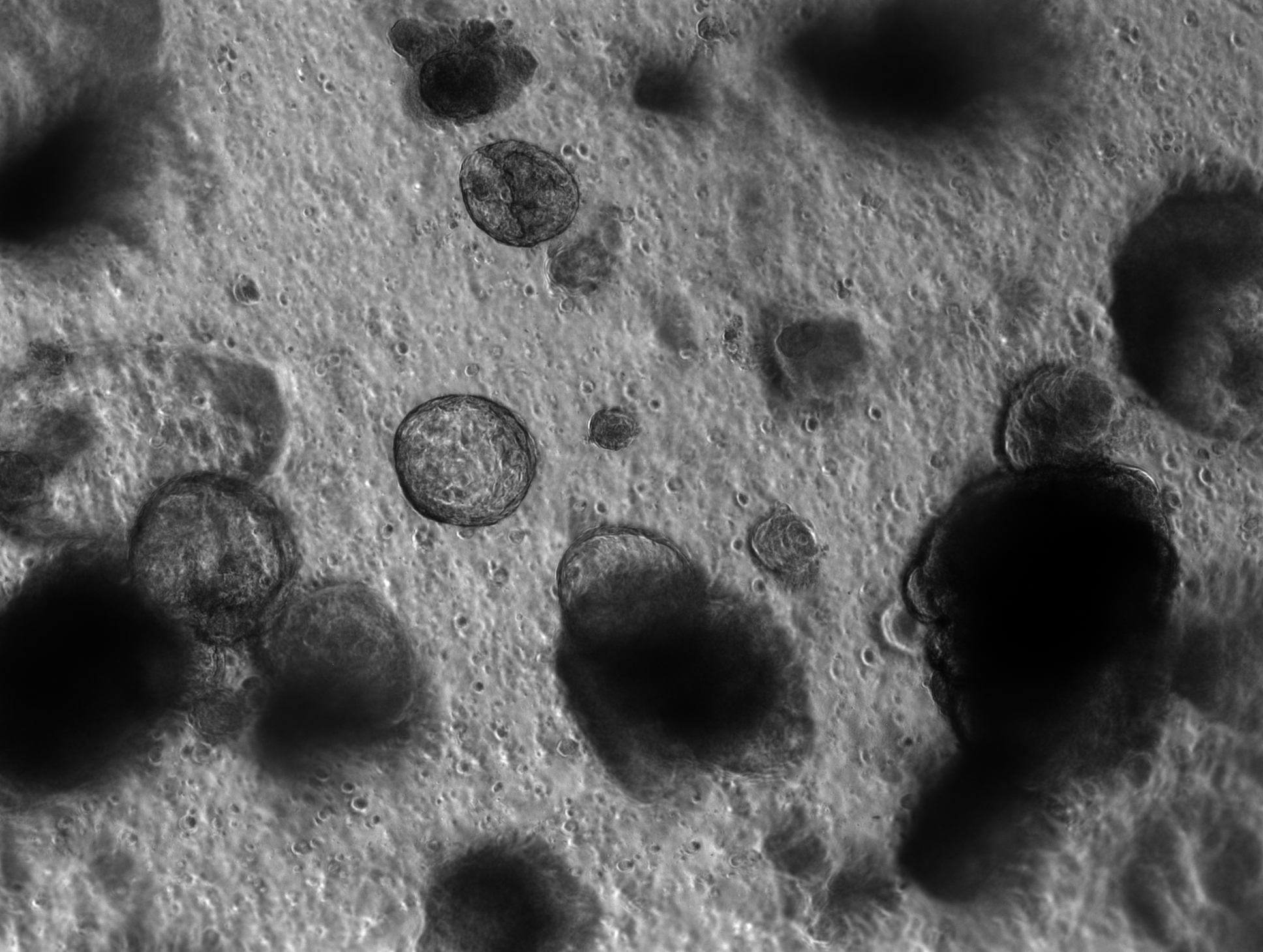

Supplement: Supplementary file 7 — Source data Fig. 1 [file 44318_2025_381_MOESM7_ESM.zip › Figure 1/1F/AK/AK_DMSO.png]

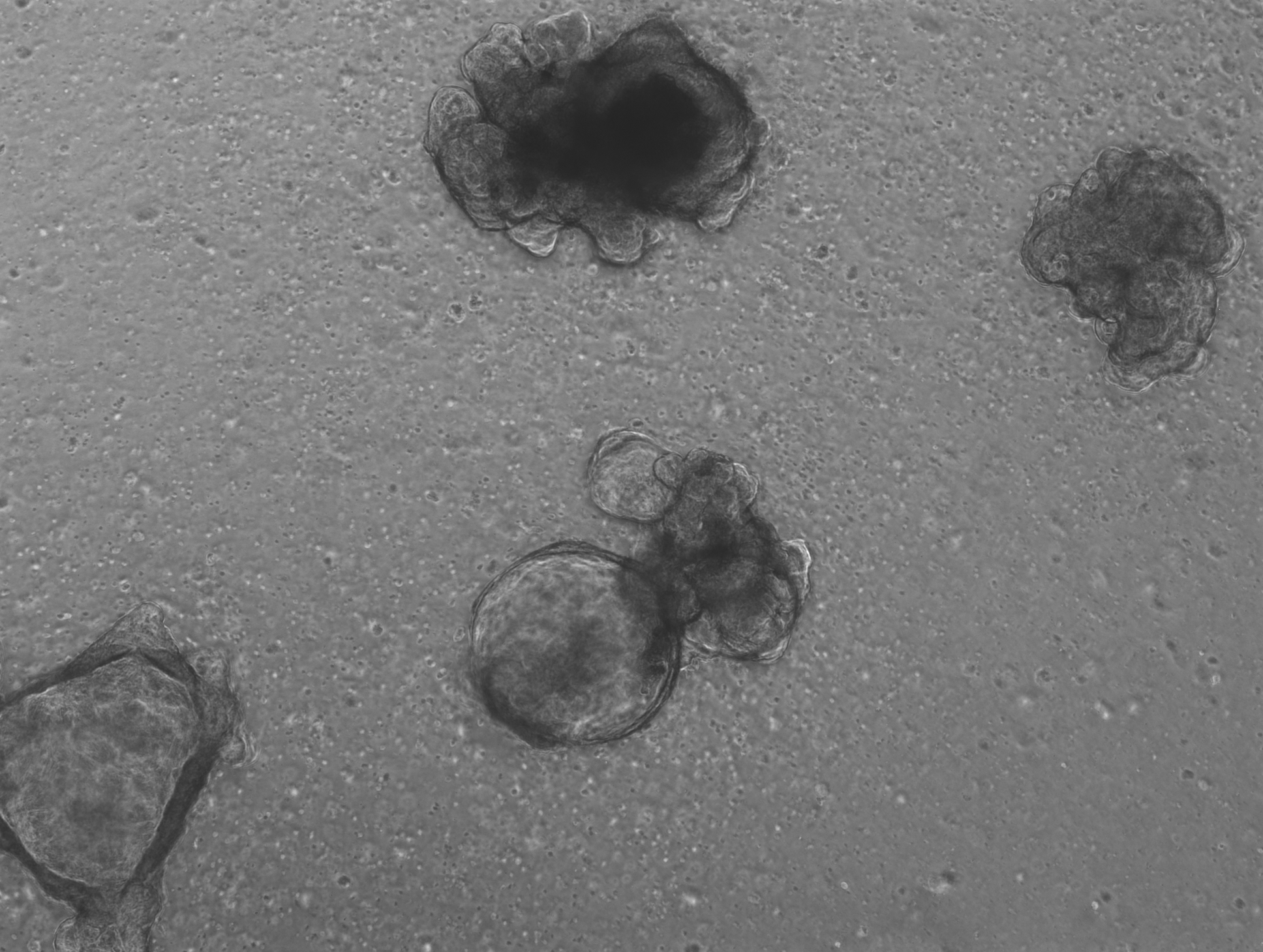

Supplement: Supplementary file 7 — Source data Fig. 1 [file 44318_2025_381_MOESM7_ESM.zip › Figure 1/1F/FAP/FAP_1000nM.png]

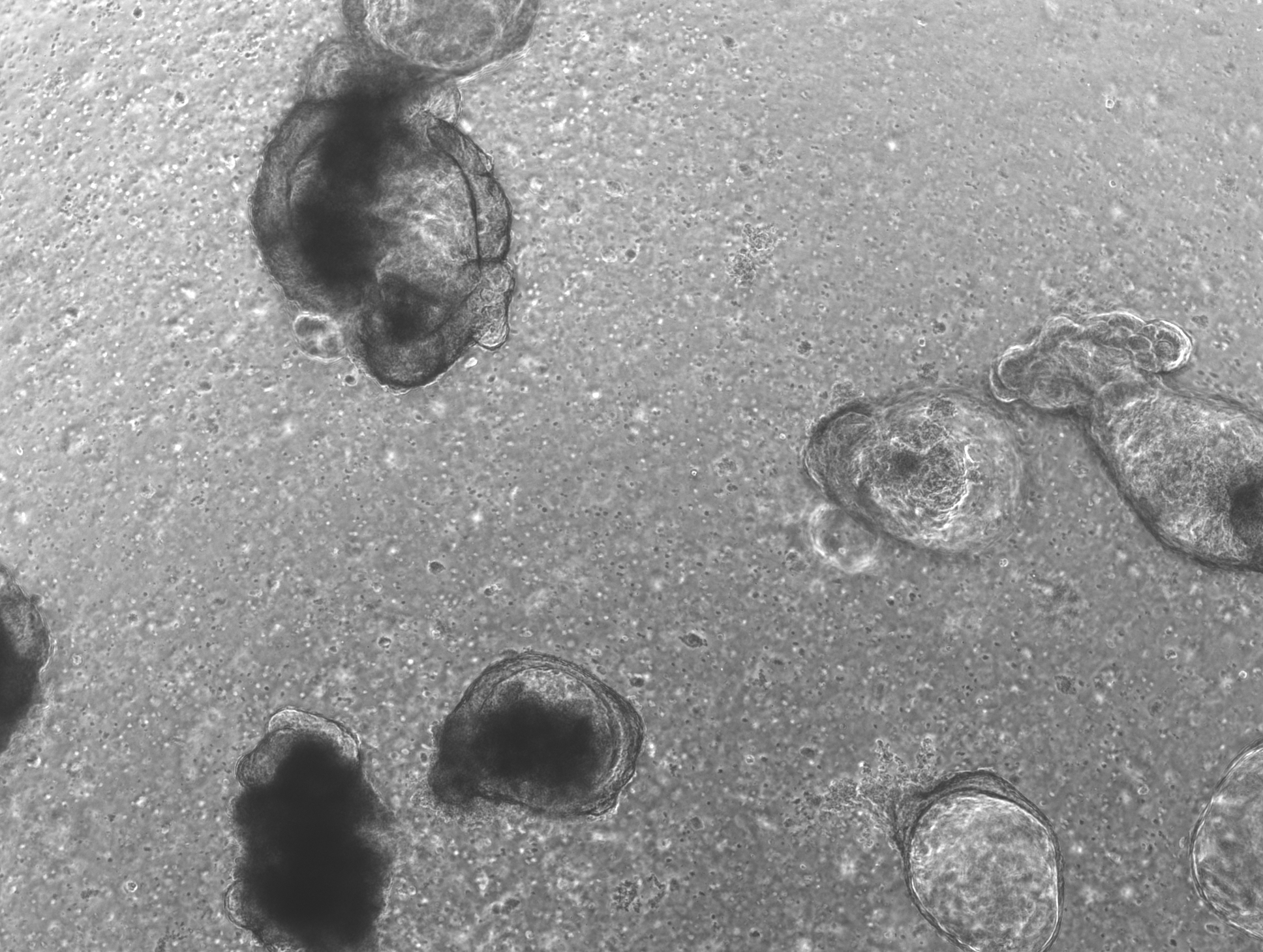

Supplement: Supplementary file 7 — Source data Fig. 1 [file 44318_2025_381_MOESM7_ESM.zip › Figure 1/1F/FAP/FAP_DMSO.png]

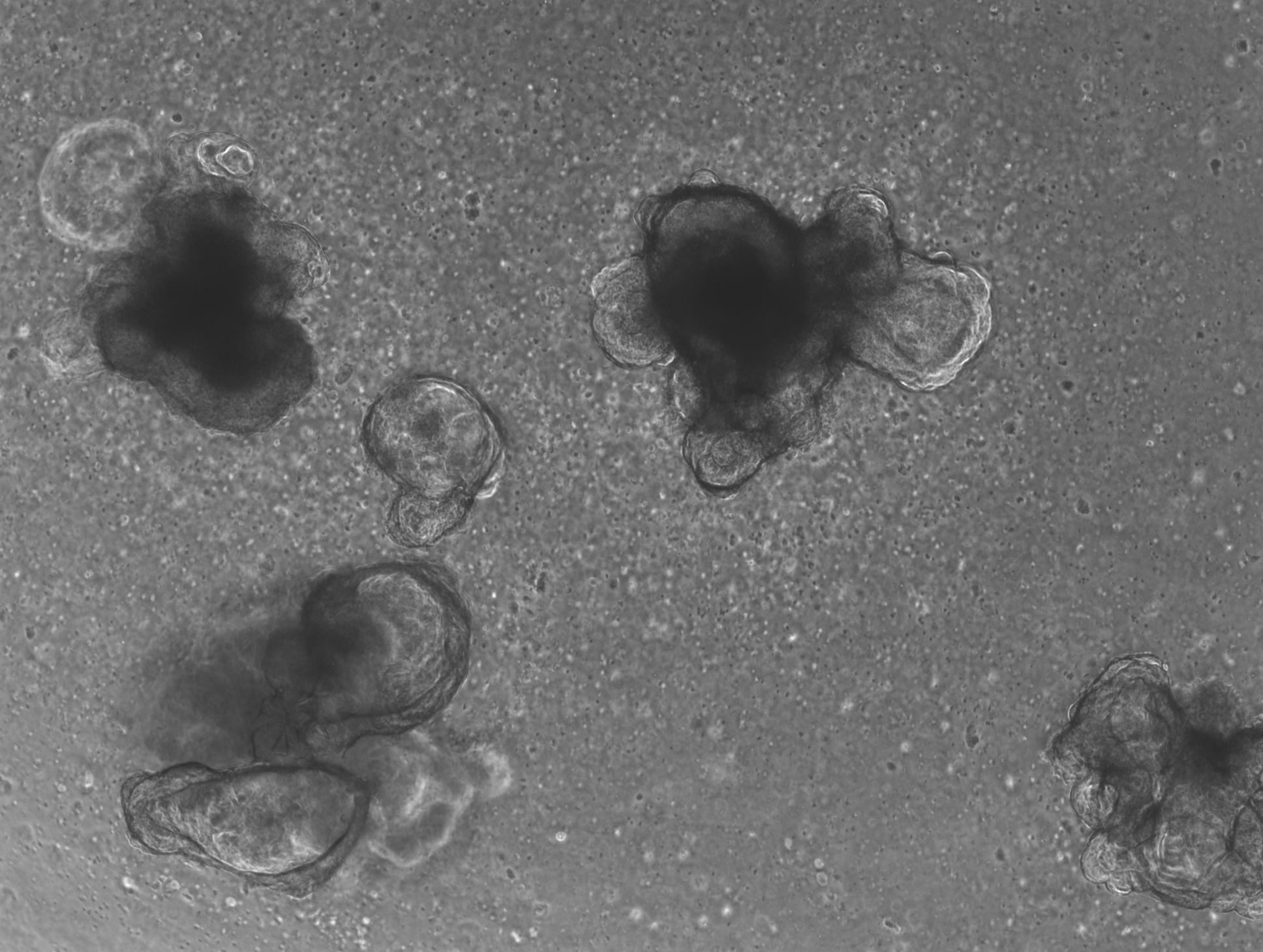

Supplement: Supplementary file 7 — Source data Fig. 1 [file 44318_2025_381_MOESM7_ESM.zip › Figure 1/1F/FAP/FAP_500nM.png]

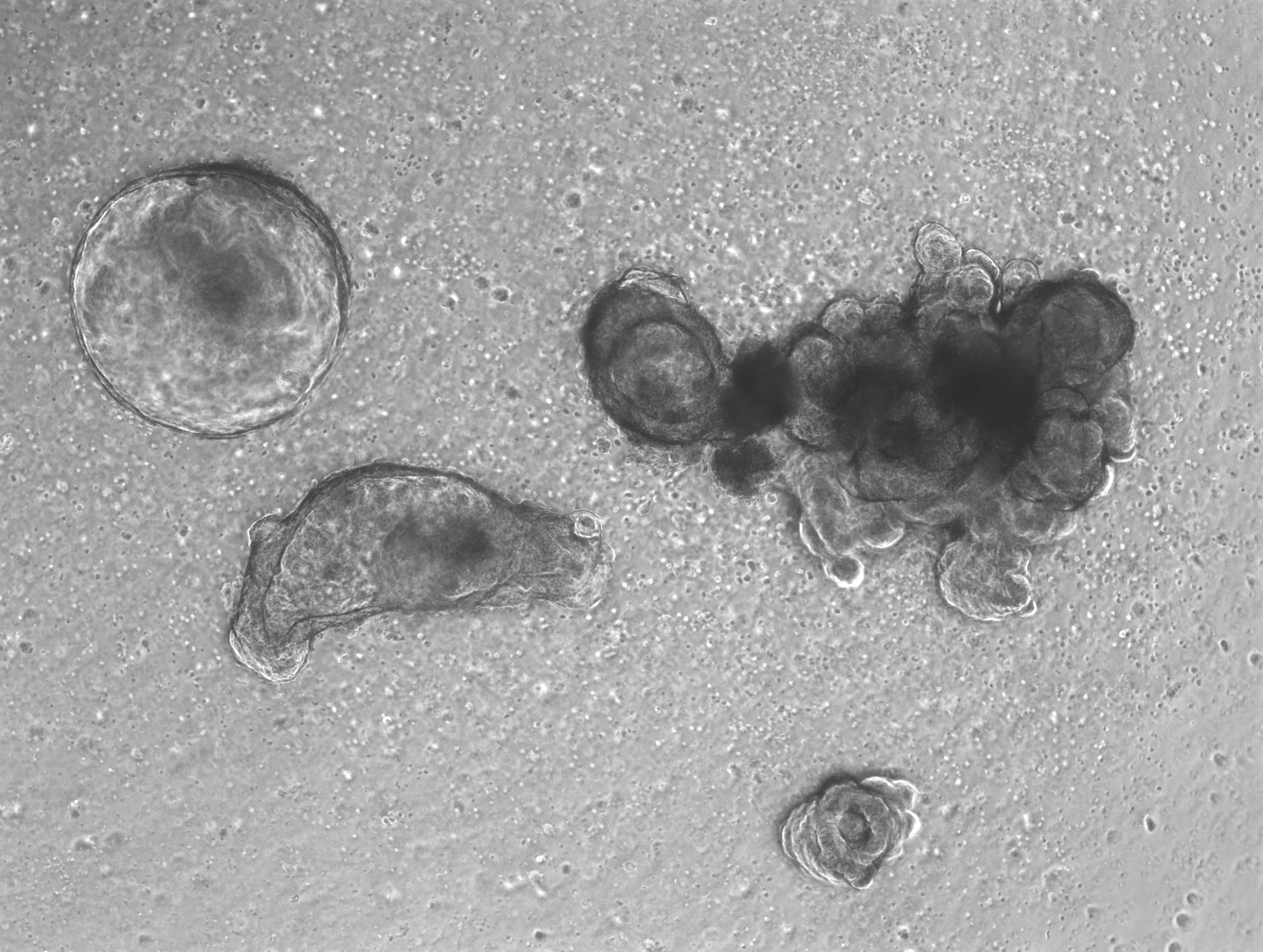

Supplement: Supplementary file 7 — Source data Fig. 1 [file 44318_2025_381_MOESM7_ESM.zip › Figure 1/1F/FAP/FAP_200nM.png]

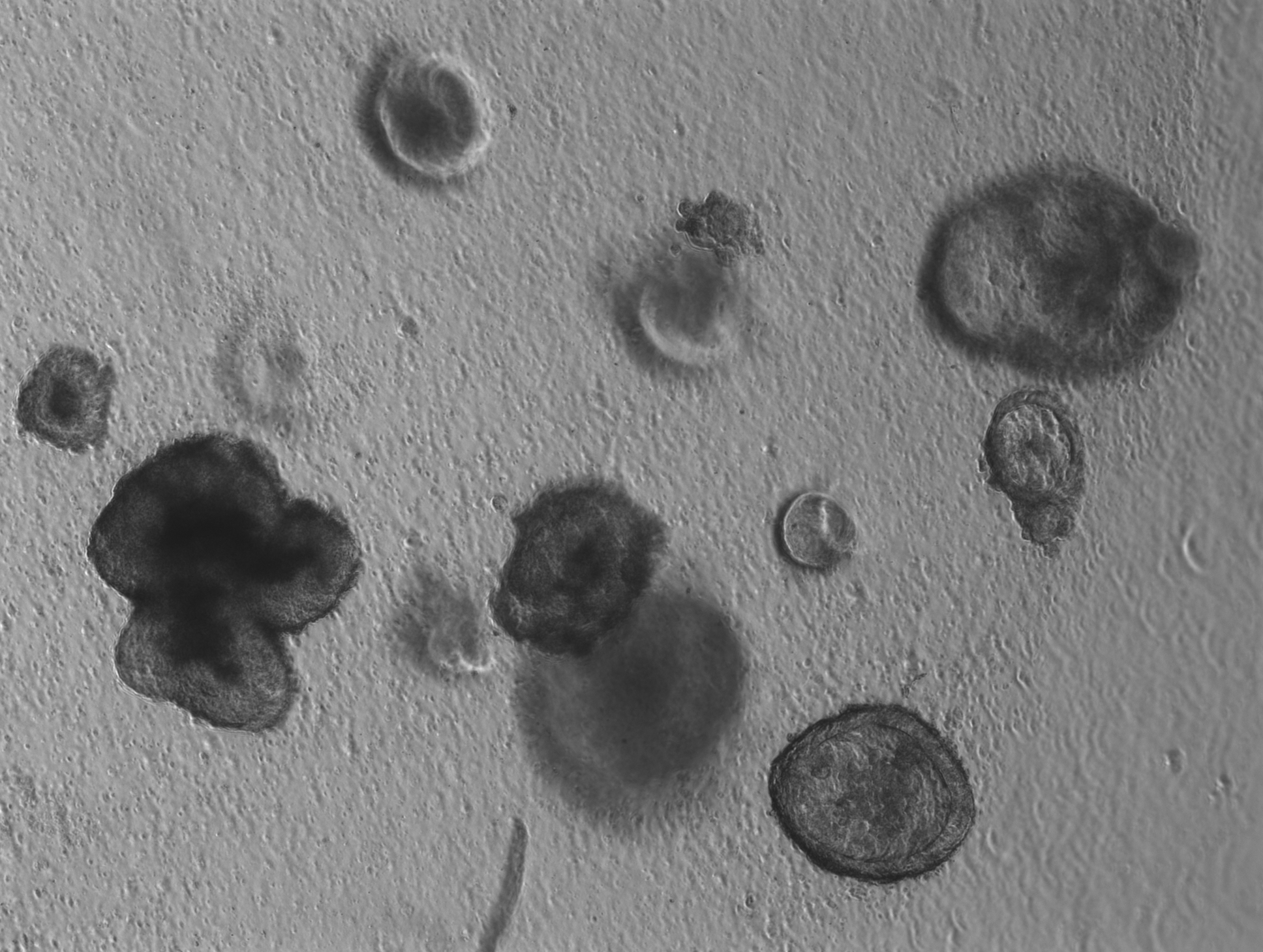

Supplement: Supplementary file 7 — Source data Fig. 1 [file 44318_2025_381_MOESM7_ESM.zip › Figure 1/1F/T4/T4_1000nM.png]

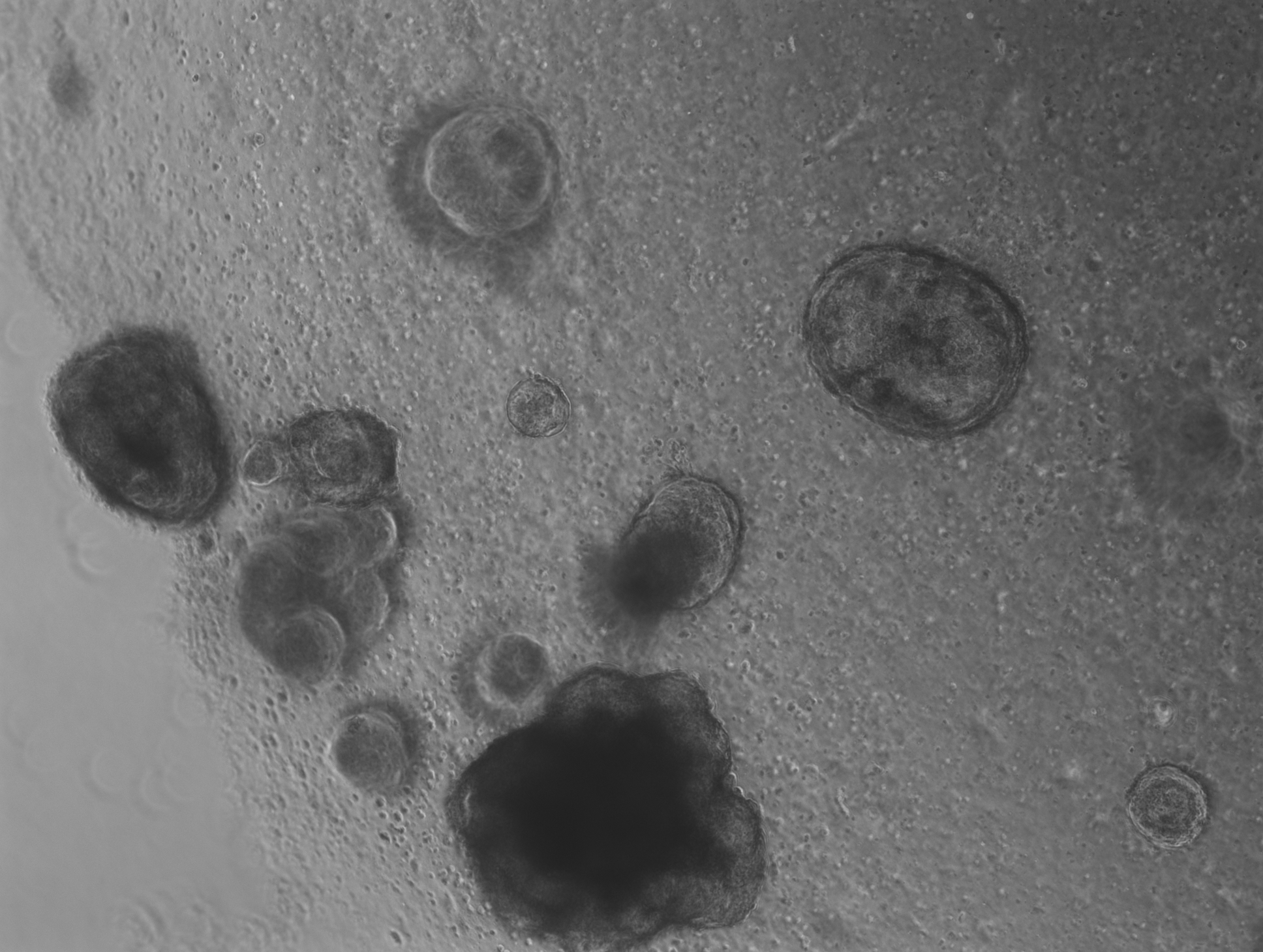

Supplement: Supplementary file 7 — Source data Fig. 1 [file 44318_2025_381_MOESM7_ESM.zip › Figure 1/1F/T4/T4_500nM.png]

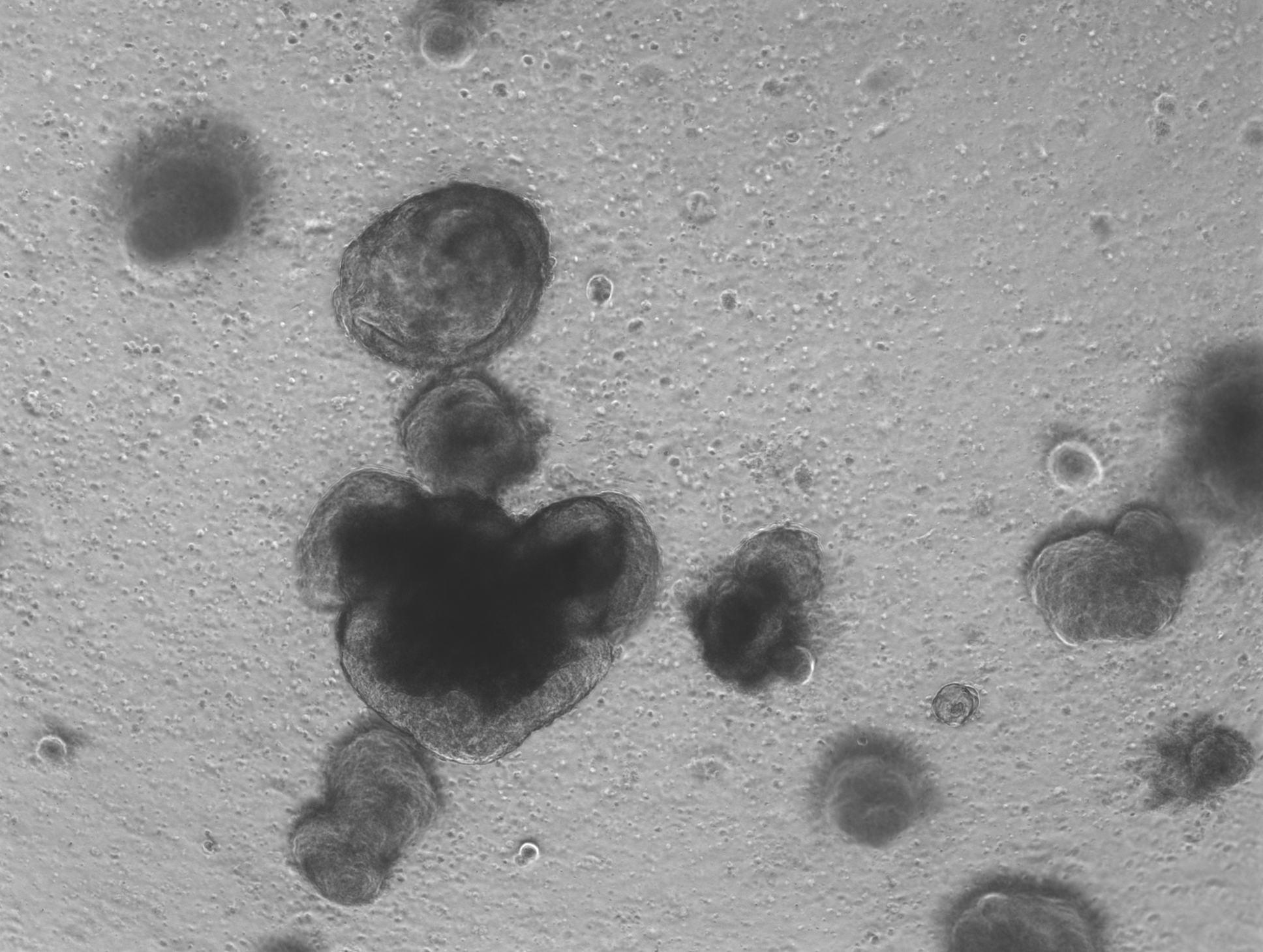

Supplement: Supplementary file 7 — Source data Fig. 1 [file 44318_2025_381_MOESM7_ESM.zip › Figure 1/1F/T4/T4_200nM.png]

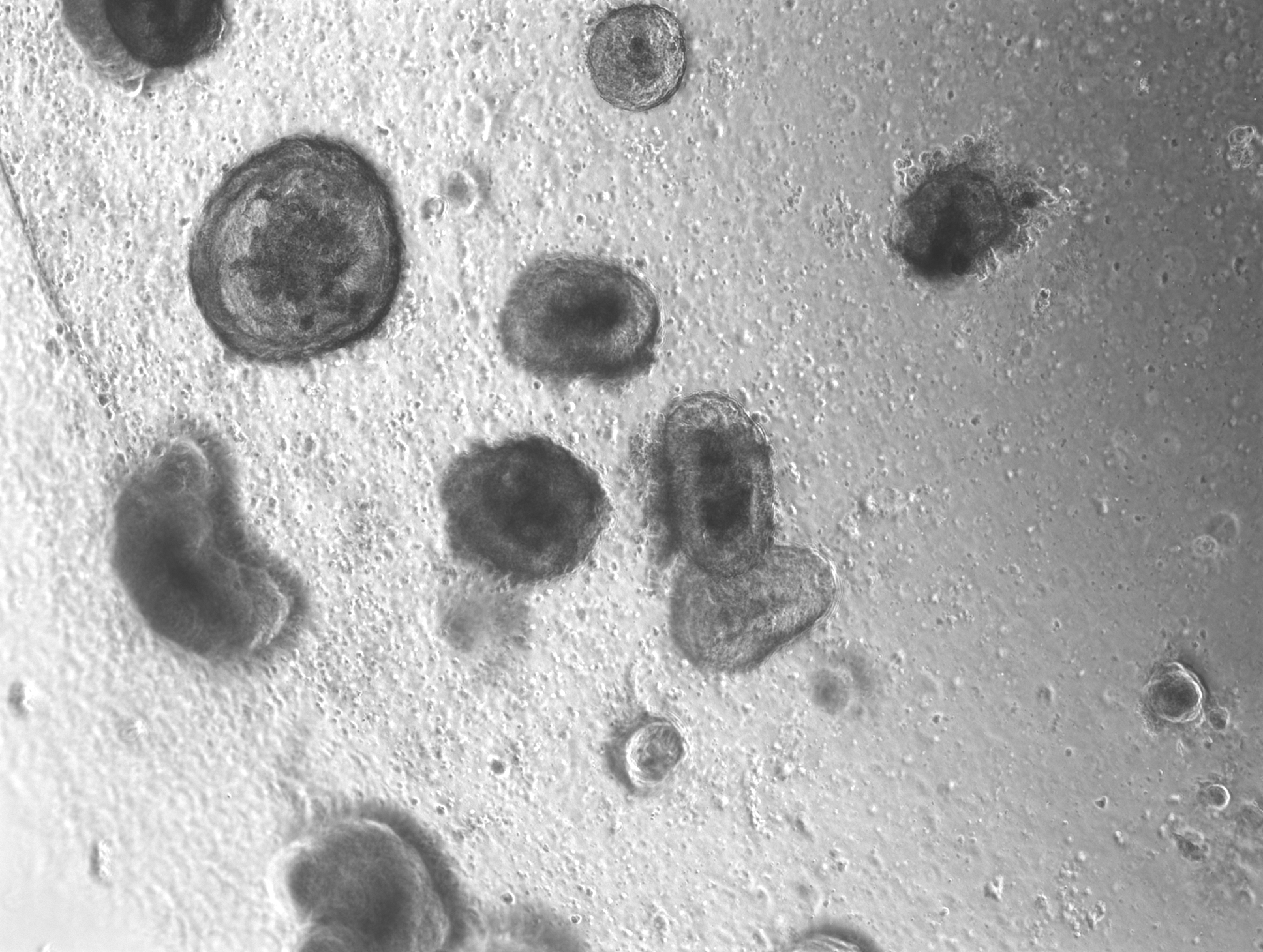

Supplement: Supplementary file 7 — Source data Fig. 1 [file 44318_2025_381_MOESM7_ESM.zip › Figure 1/1F/T4/T4_DMSO.png]

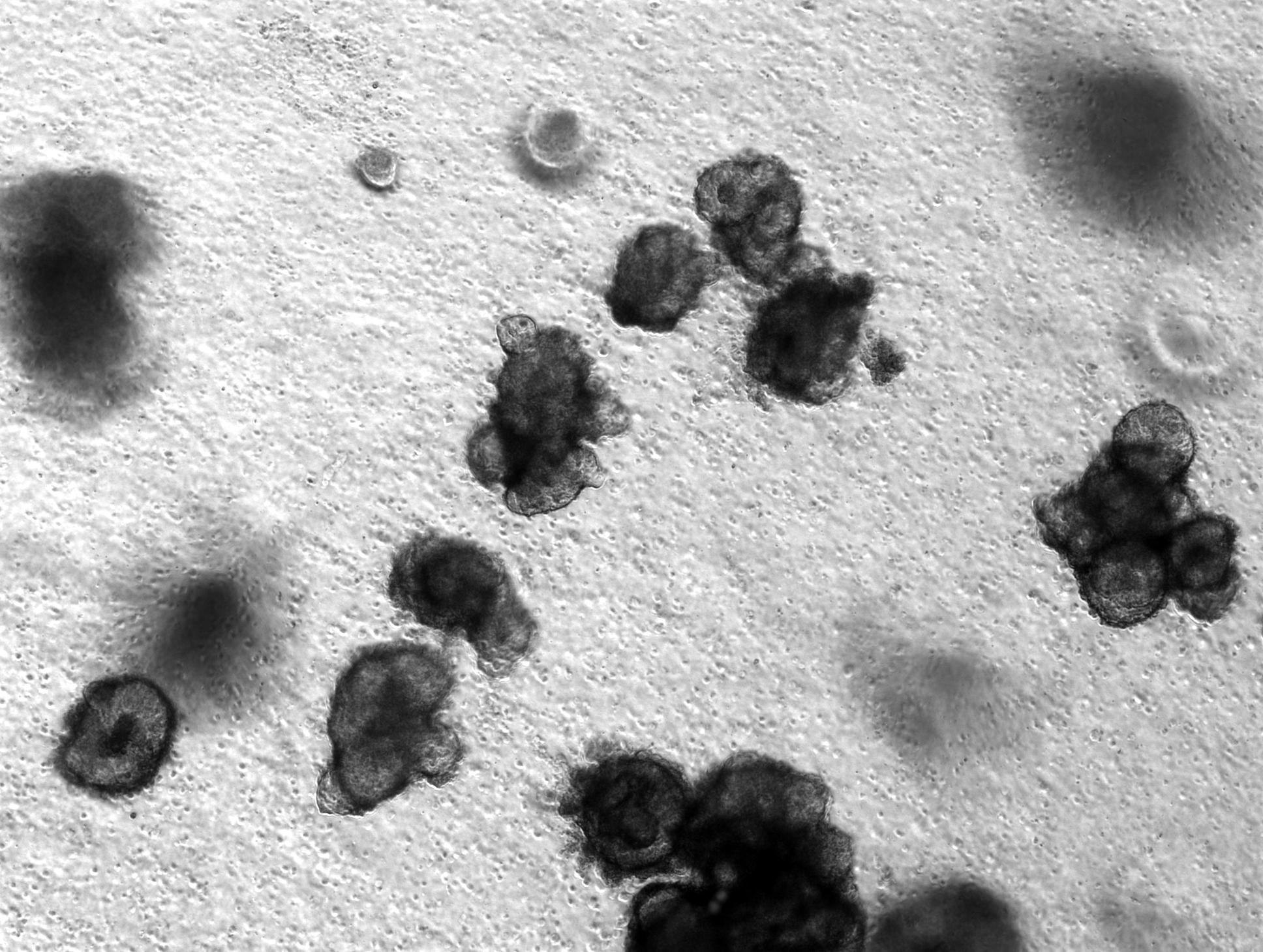

Supplement: Supplementary file 7 — Source data Fig. 1 [file 44318_2025_381_MOESM7_ESM.zip › Figure 1/1F/HD-3/HD3_1000nM.png]

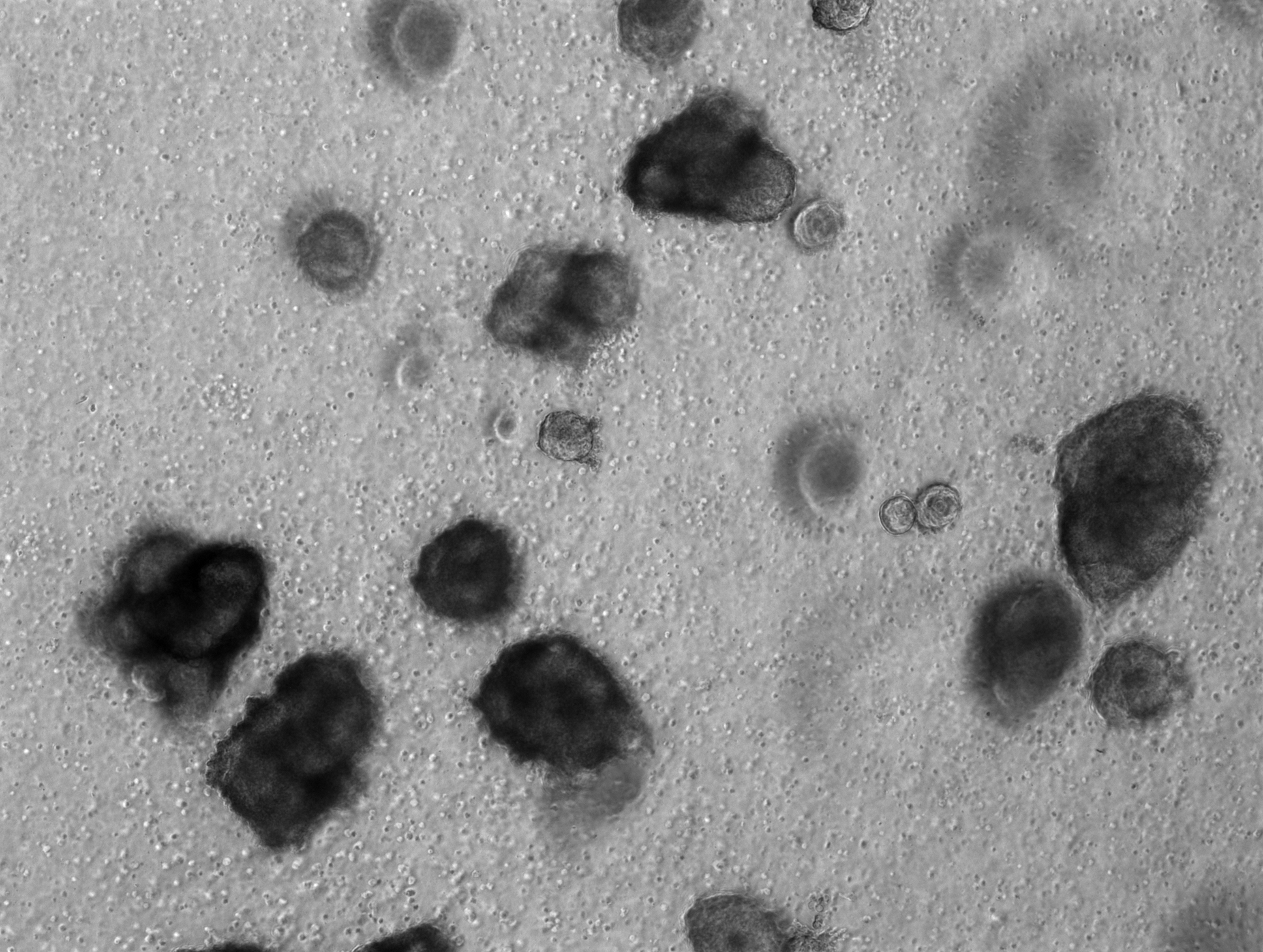

Supplement: Supplementary file 7 — Source data Fig. 1 [file 44318_2025_381_MOESM7_ESM.zip › Figure 1/1F/HD-3/HD3_200nM.png]

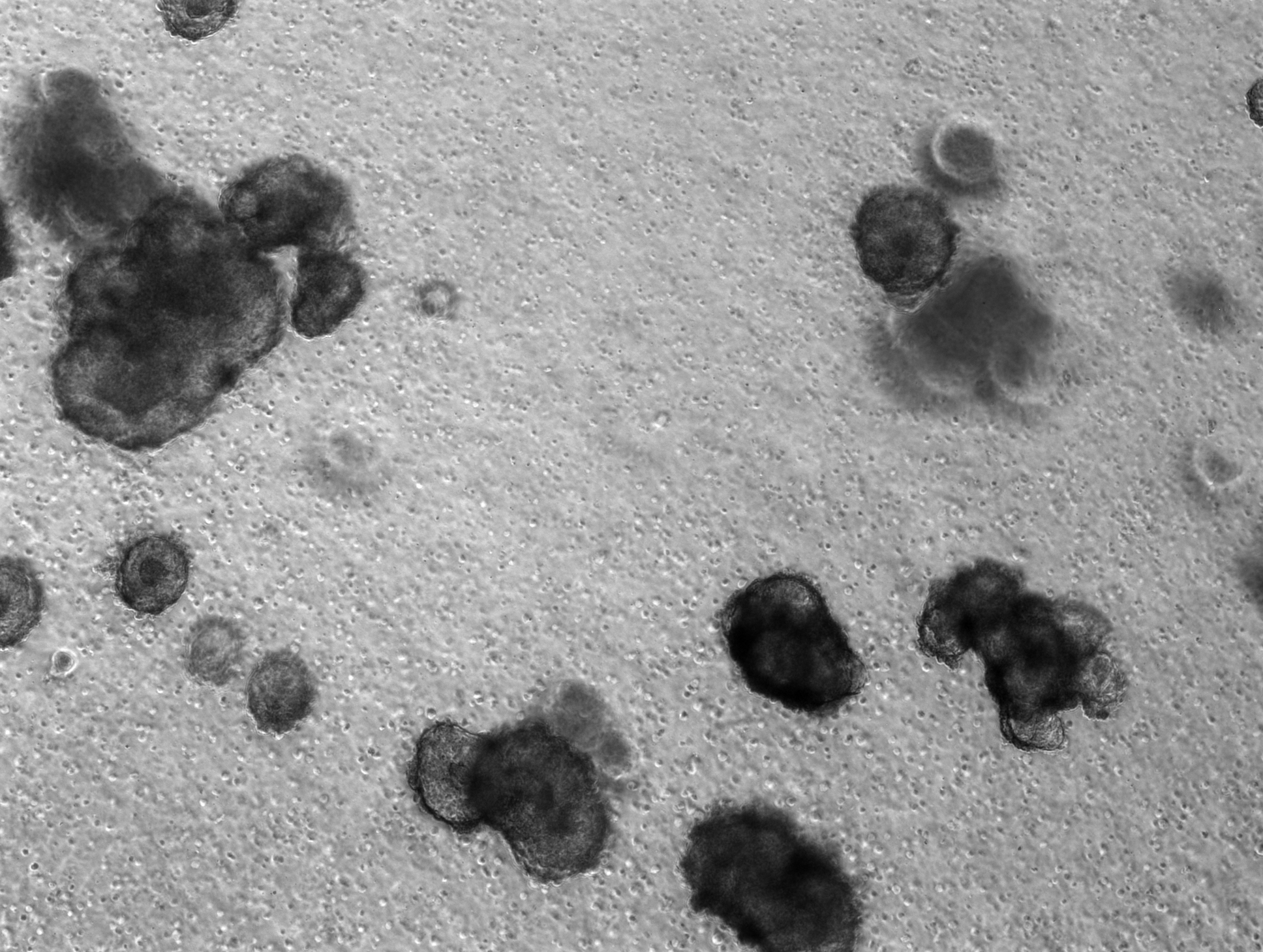

Supplement: Supplementary file 7 — Source data Fig. 1 [file 44318_2025_381_MOESM7_ESM.zip › Figure 1/1F/HD-3/HD3_DMSO.png]

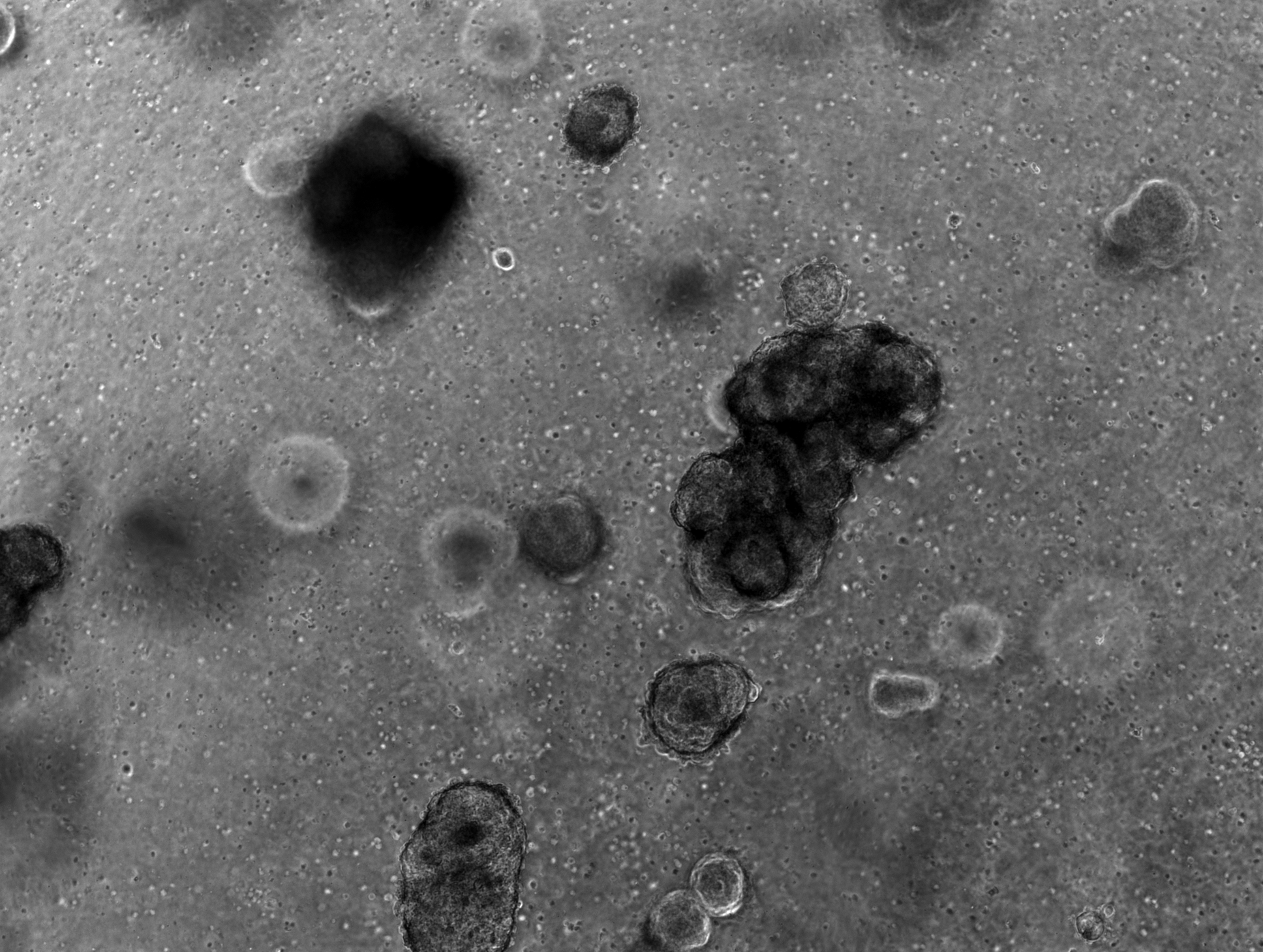

Supplement: Supplementary file 7 — Source data Fig. 1 [file 44318_2025_381_MOESM7_ESM.zip › Figure 1/1F/HD-3/HD3_500nM.png]

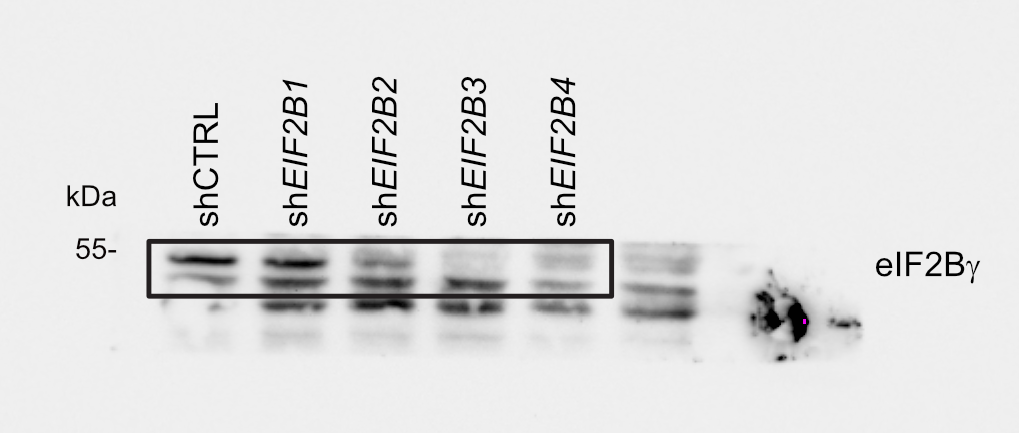

Supplement: Supplementary file 8 — Source data Fig. 2 [file 44318_2025_381_MOESM8_ESM.zip › Figure 2/2A/western eIF2Bg.tif]

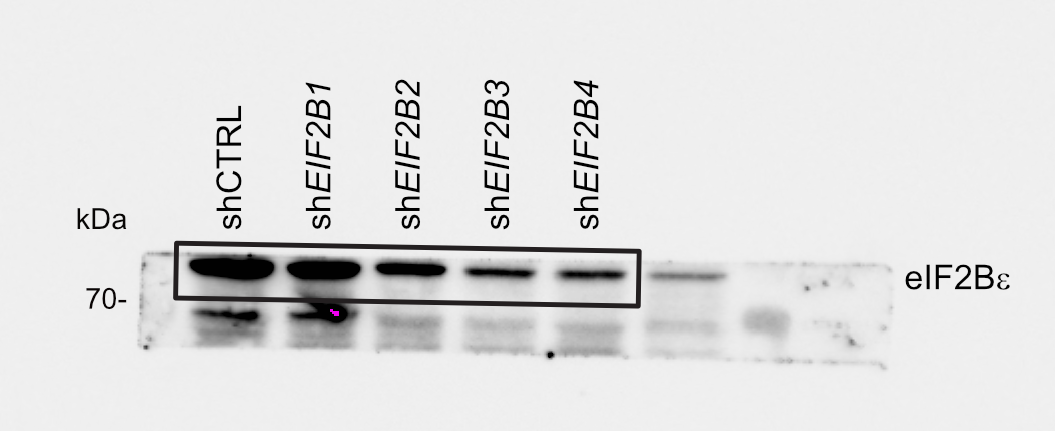

Supplement: Supplementary file 8 — Source data Fig. 2 [file 44318_2025_381_MOESM8_ESM.zip › Figure 2/2A/western eIF2Be.tif]

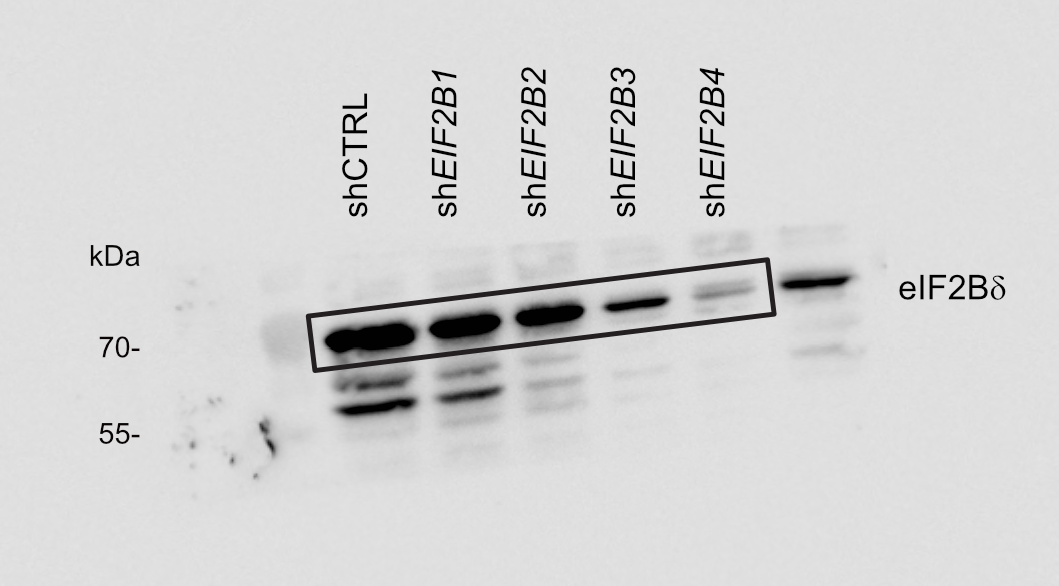

Supplement: Supplementary file 8 — Source data Fig. 2 [file 44318_2025_381_MOESM8_ESM.zip › Figure 2/2A/western eIF2Bd.tif]

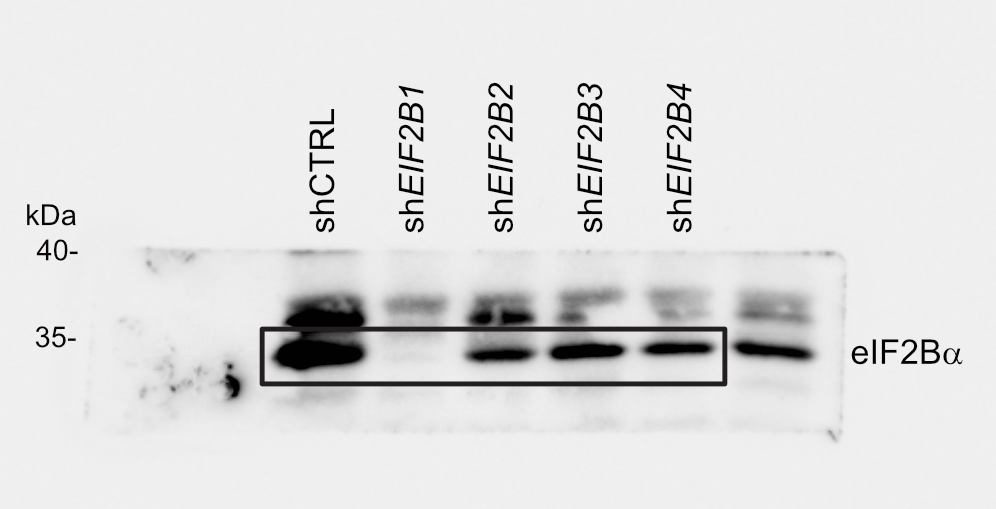

Supplement: Supplementary file 8 — Source data Fig. 2 [file 44318_2025_381_MOESM8_ESM.zip › Figure 2/2A/western eIF2Ba.tif]

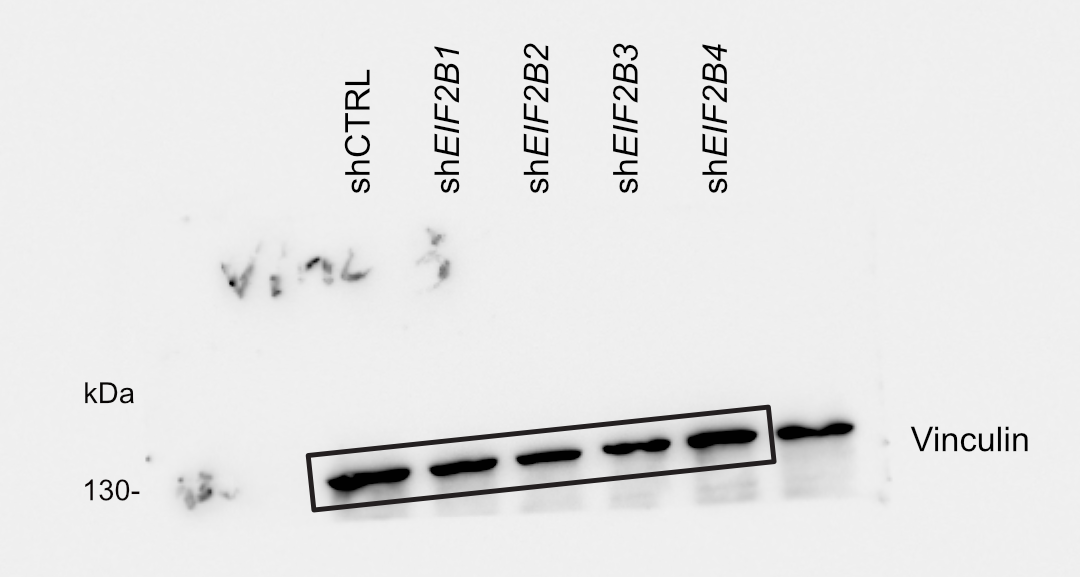

Supplement: Supplementary file 8 — Source data Fig. 2 [file 44318_2025_381_MOESM8_ESM.zip › Figure 2/2A/western vinculin.tif]

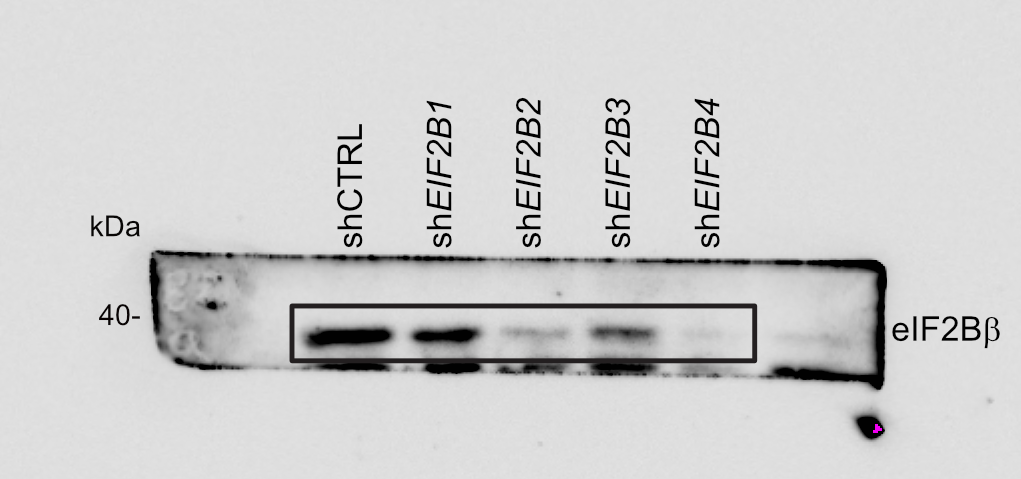

Supplement: Supplementary file 8 — Source data Fig. 2 [file 44318_2025_381_MOESM8_ESM.zip › Figure 2/2A/western eIF2Bb.tif]

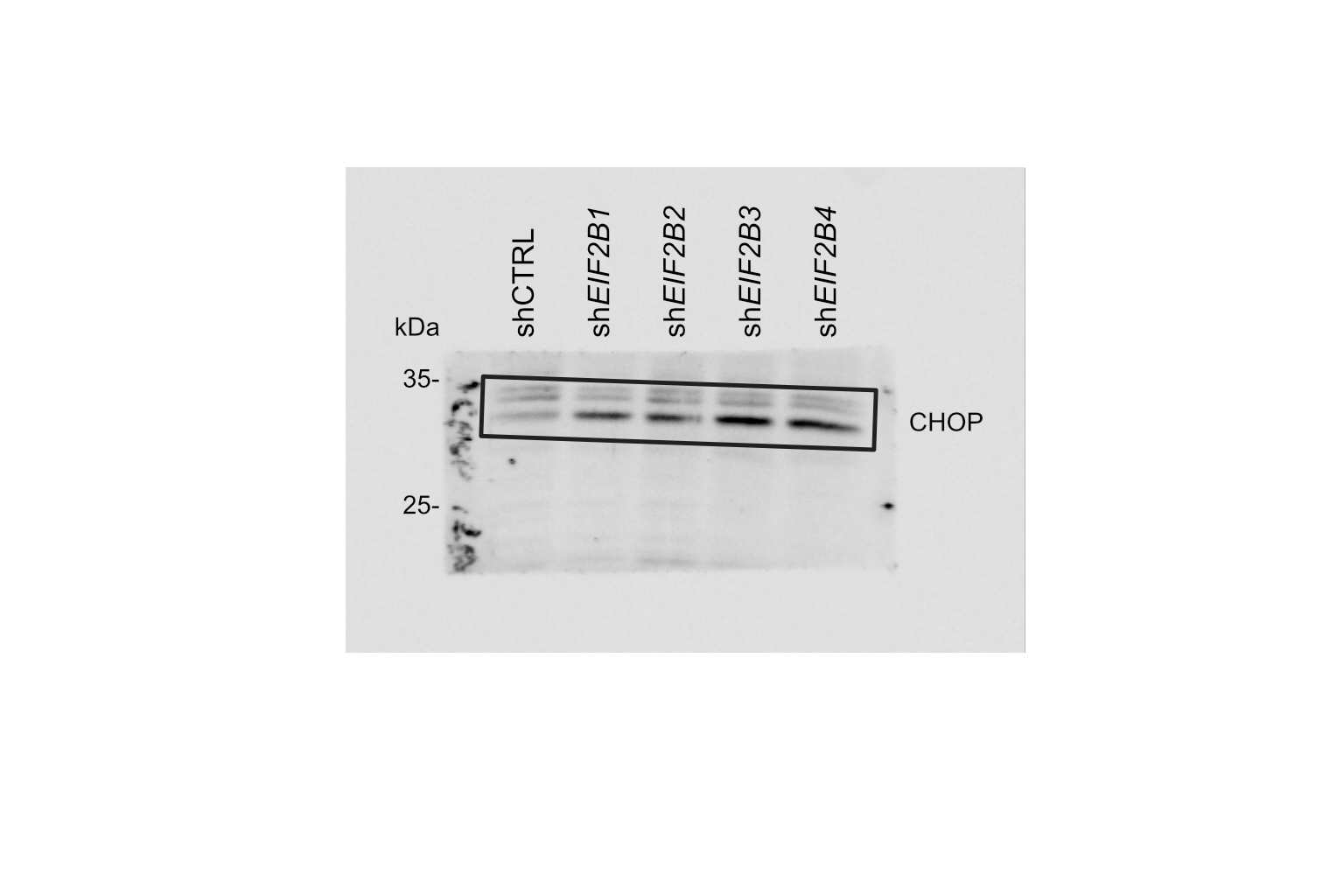

Supplement: Supplementary file 8 — Source data Fig. 2 [file 44318_2025_381_MOESM8_ESM.zip › Figure 2/2C/western chop.tif]

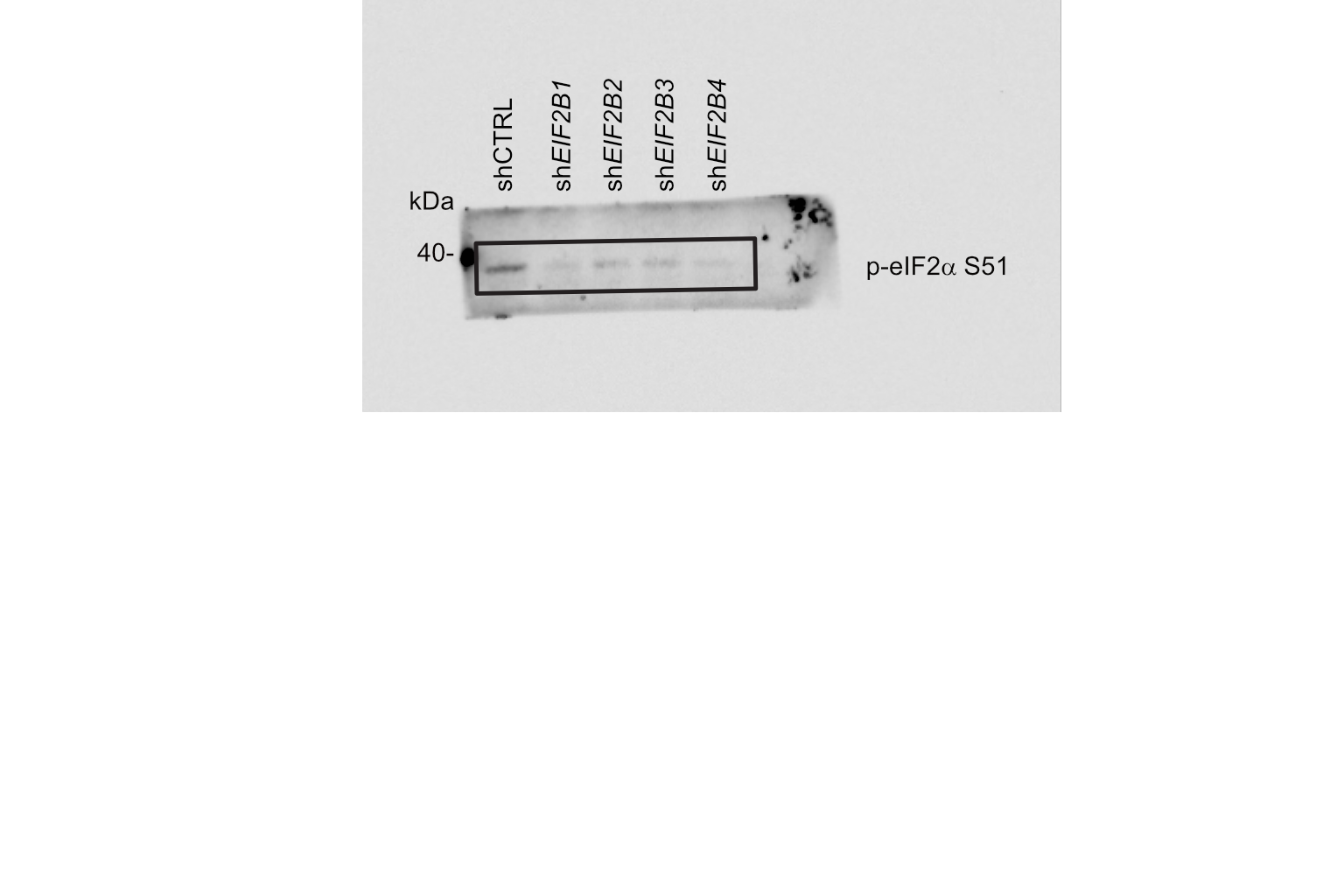

Supplement: Supplementary file 8 — Source data Fig. 2 [file 44318_2025_381_MOESM8_ESM.zip › Figure 2/2C/western p-eIF2a S51.tif]

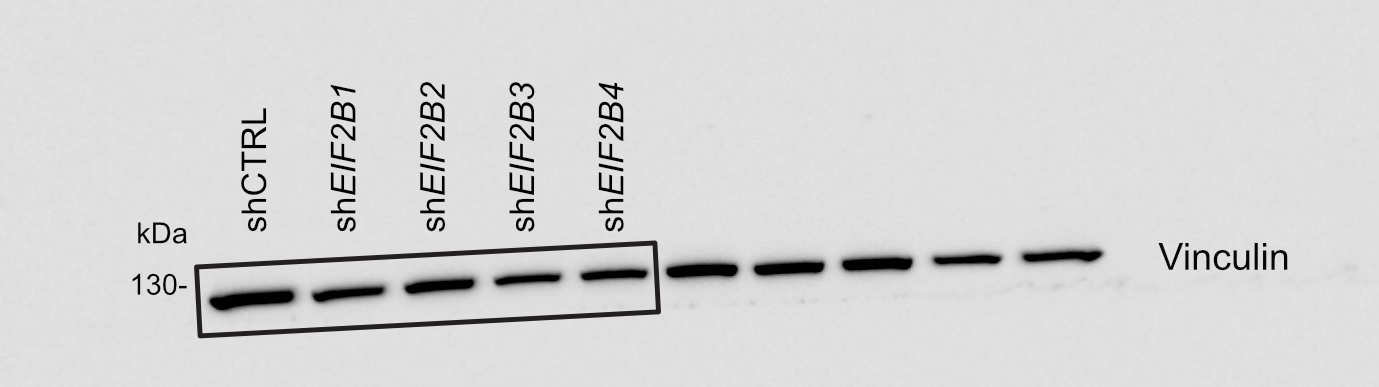

Supplement: Supplementary file 8 — Source data Fig. 2 [file 44318_2025_381_MOESM8_ESM.zip › Figure 2/2C/western vinculin.tif]

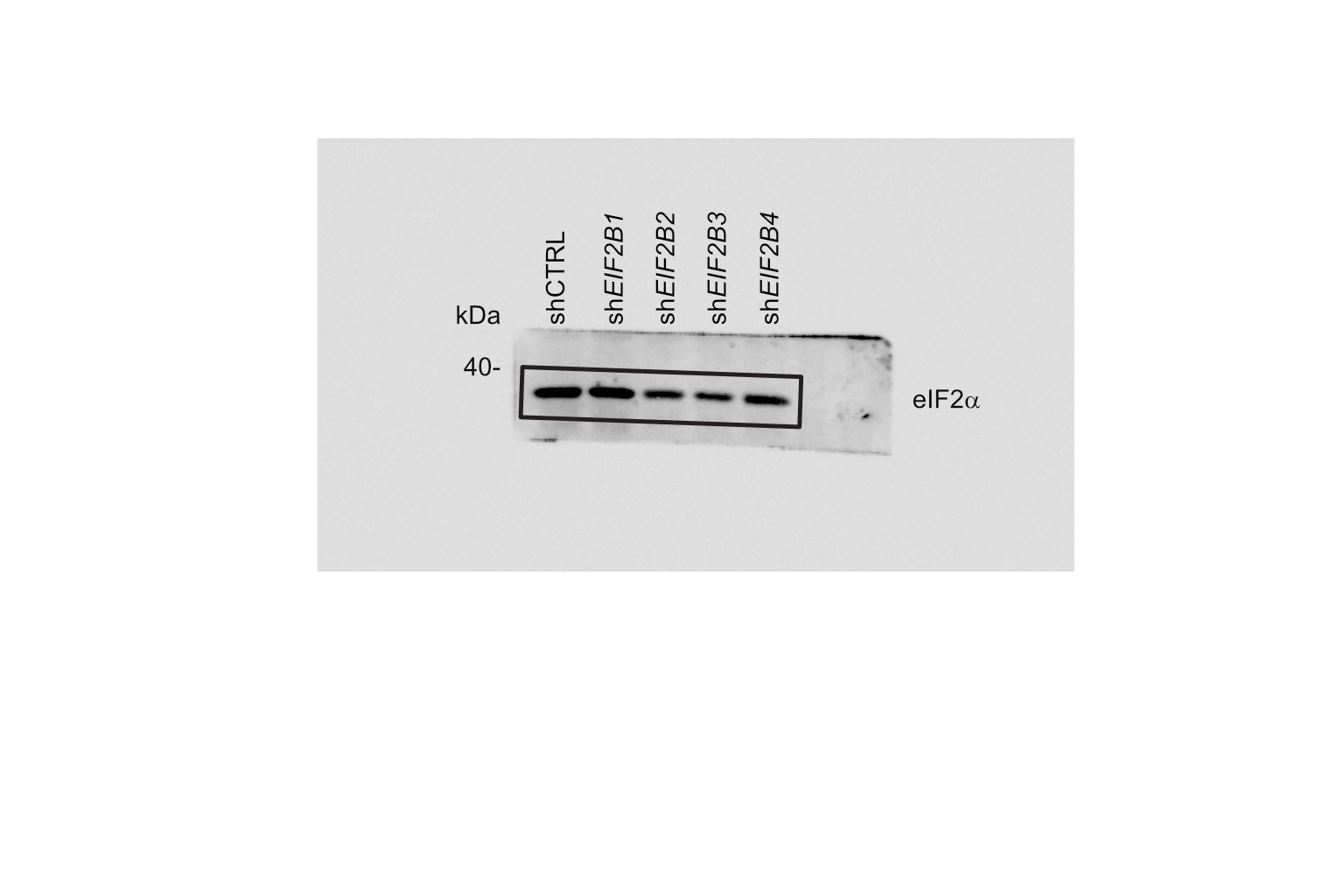

Supplement: Supplementary file 8 — Source data Fig. 2 [file 44318_2025_381_MOESM8_ESM.zip › Figure 2/2C/western eIF2a.tif]

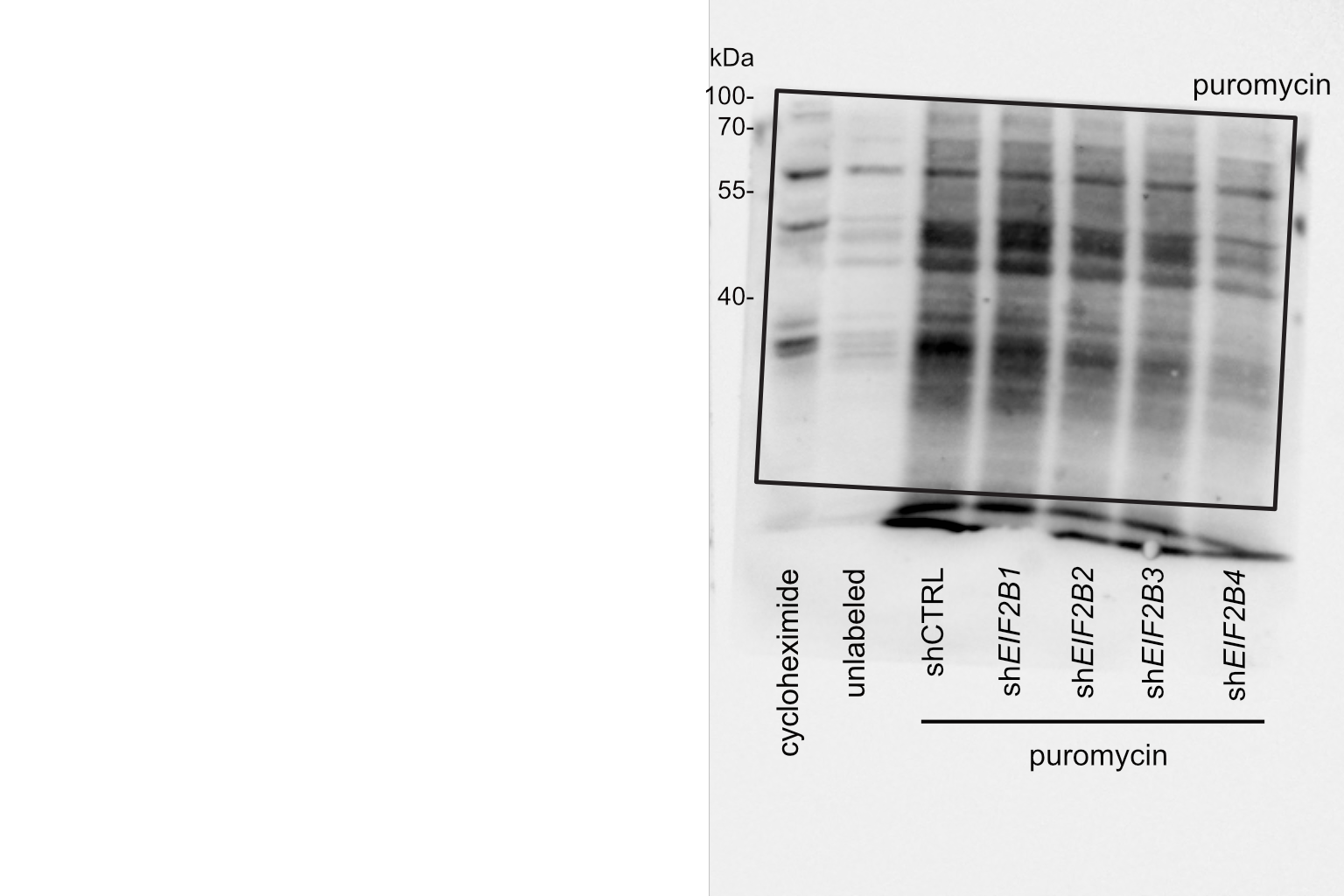

Supplement: Supplementary file 8 — Source data Fig. 2 [file 44318_2025_381_MOESM8_ESM.zip › Figure 2/2B/western puromycin.tif]

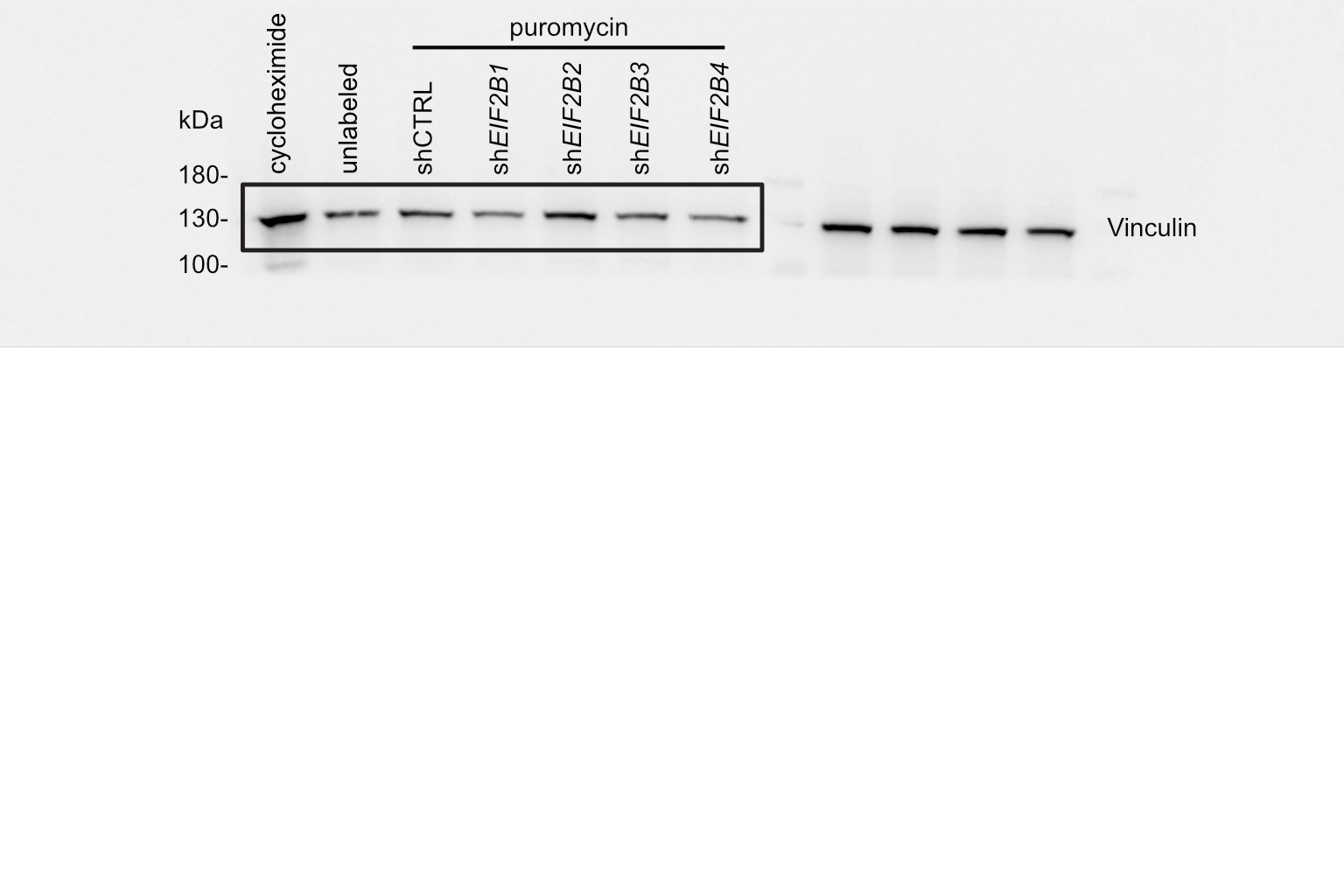

Supplement: Supplementary file 8 — Source data Fig. 2 [file 44318_2025_381_MOESM8_ESM.zip › Figure 2/2B/western vinculin.tif]

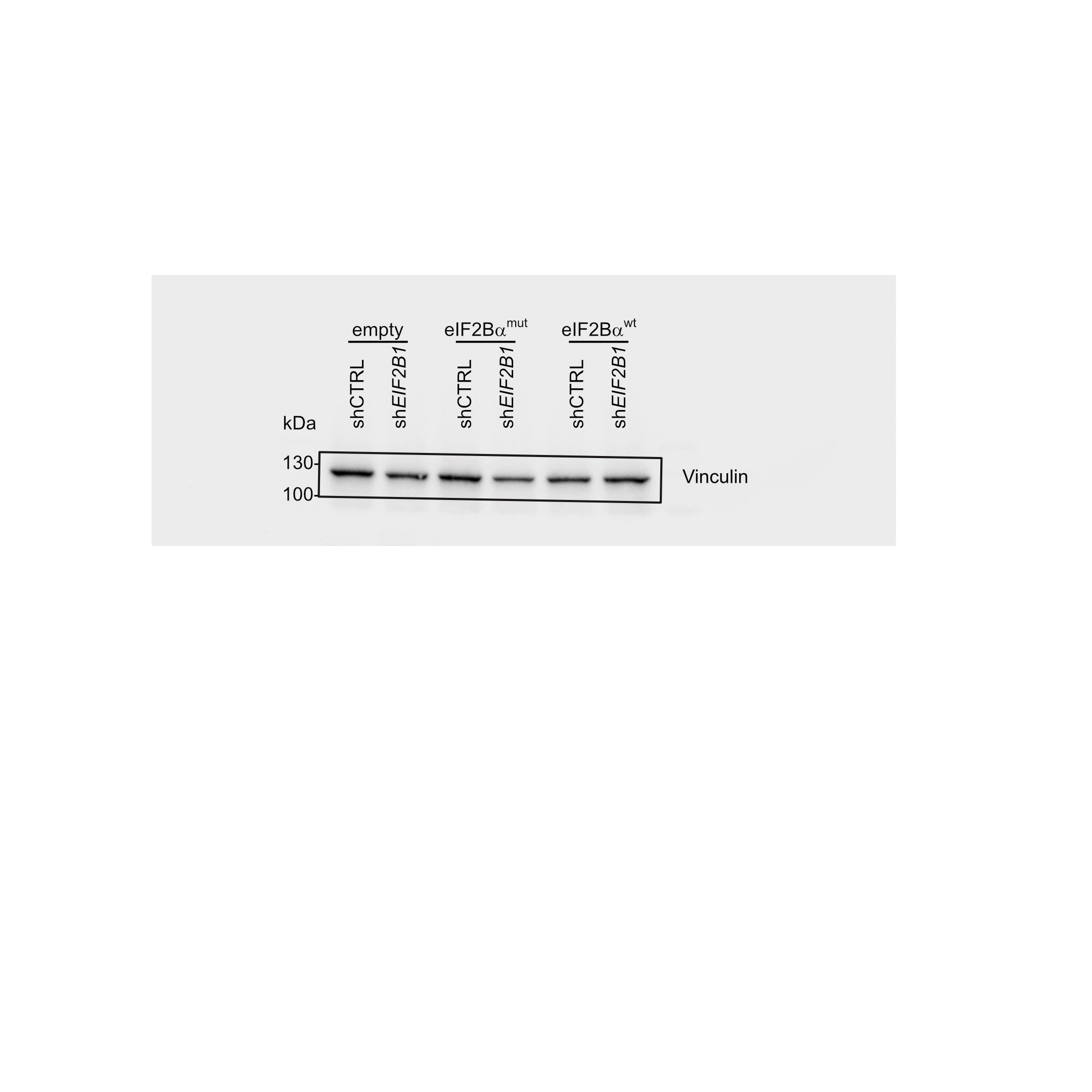

Supplement: Supplementary file 9 — Source data Fig. 3 [file 44318_2025_381_MOESM9_ESM.zip › Figure 3/3B/western vinculin.tiff]

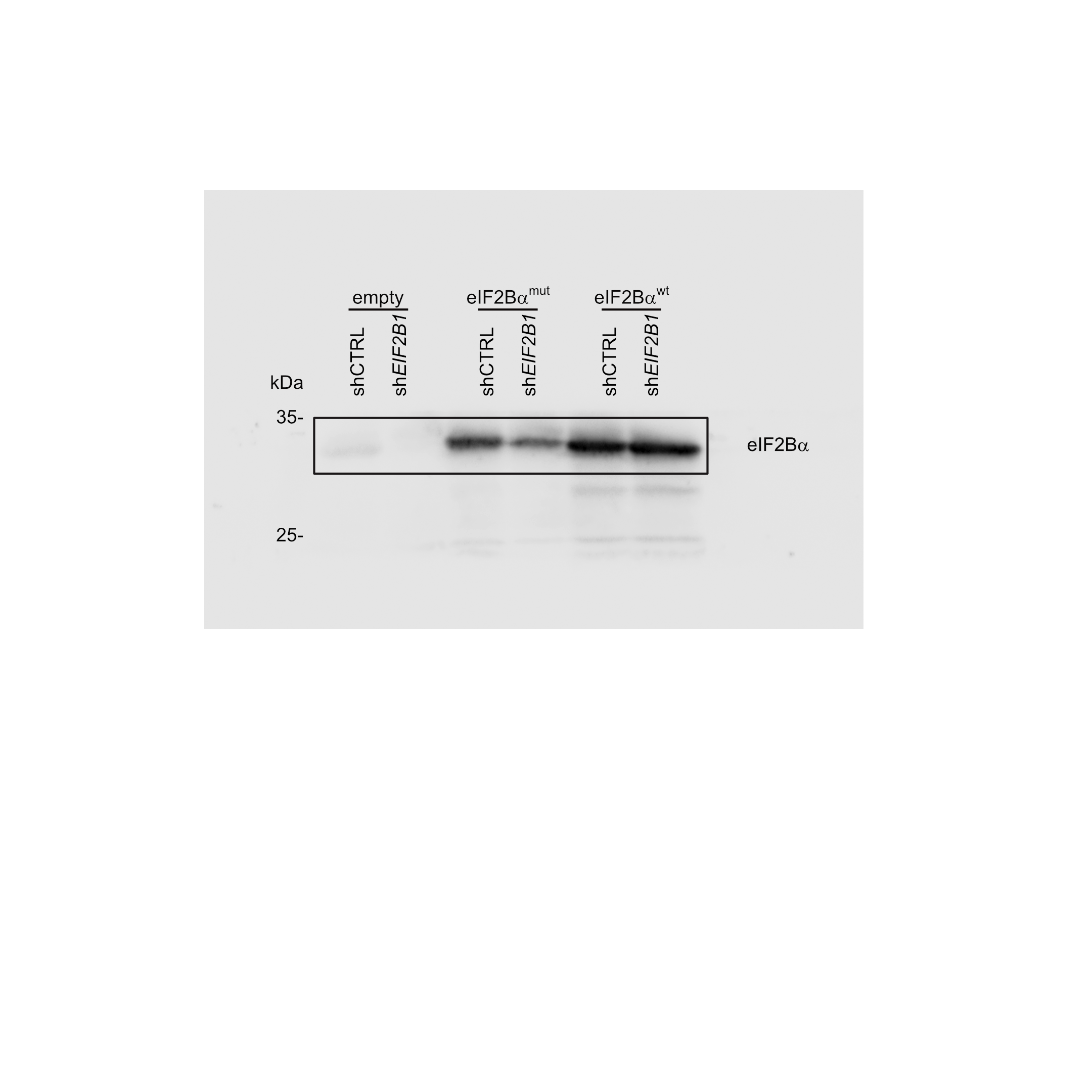

Supplement: Supplementary file 9 — Source data Fig. 3 [file 44318_2025_381_MOESM9_ESM.zip › Figure 3/3B/western eIF2Ba.tiff]

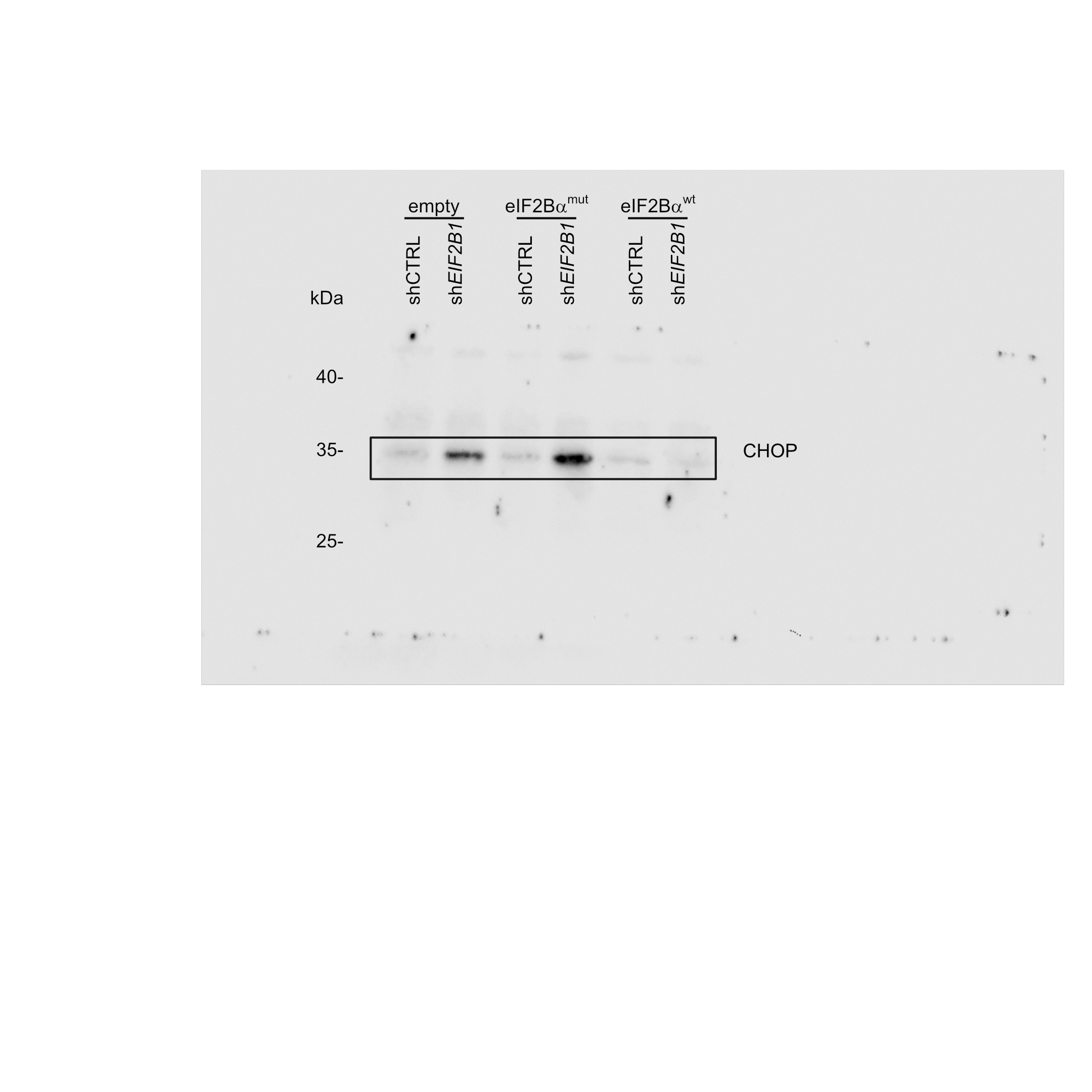

Supplement: Supplementary file 9 — Source data Fig. 3 [file 44318_2025_381_MOESM9_ESM.zip › Figure 3/3D/western chop.tiff]

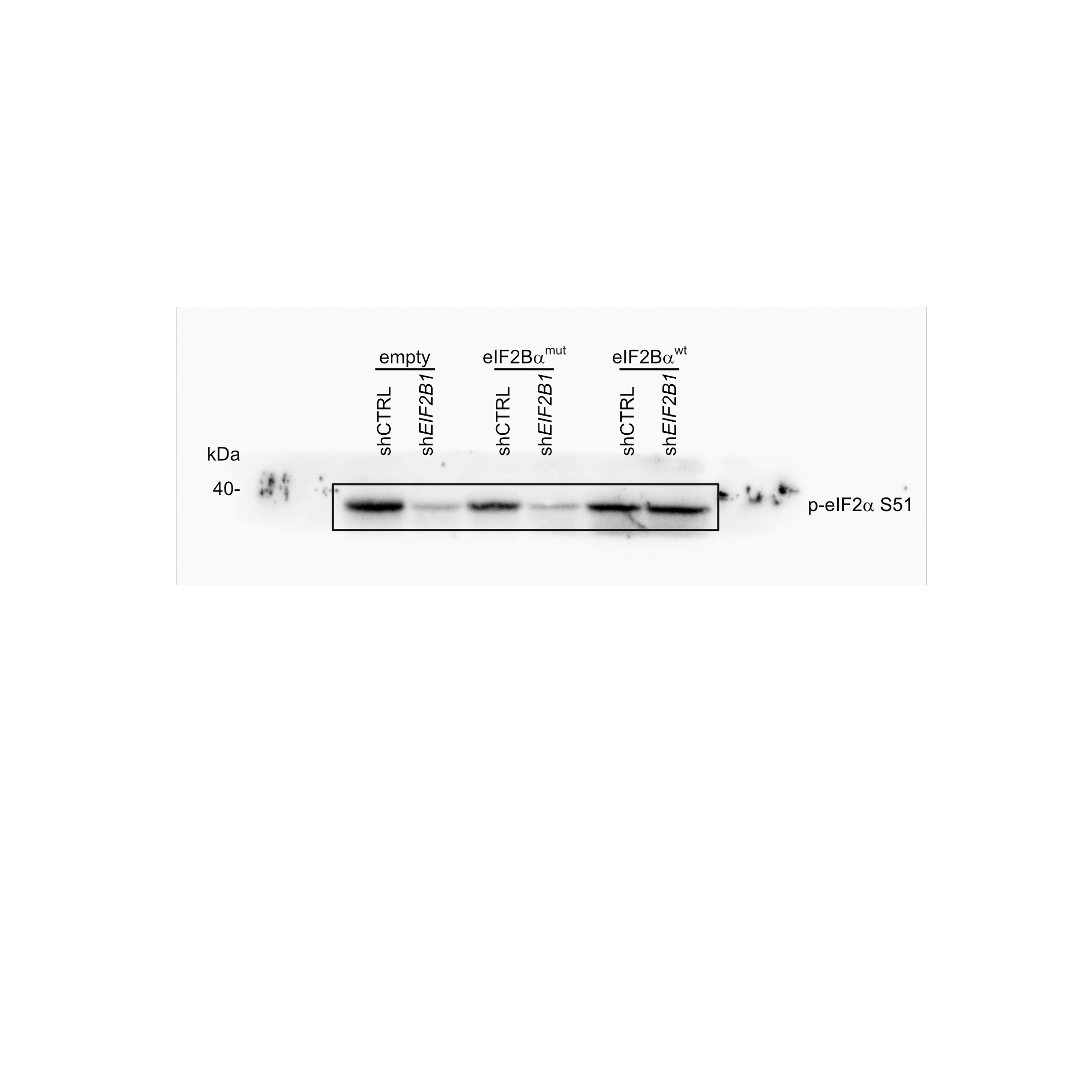

Supplement: Supplementary file 9 — Source data Fig. 3 [file 44318_2025_381_MOESM9_ESM.zip › Figure 3/3D/western p-eIF2a S51.tiff]

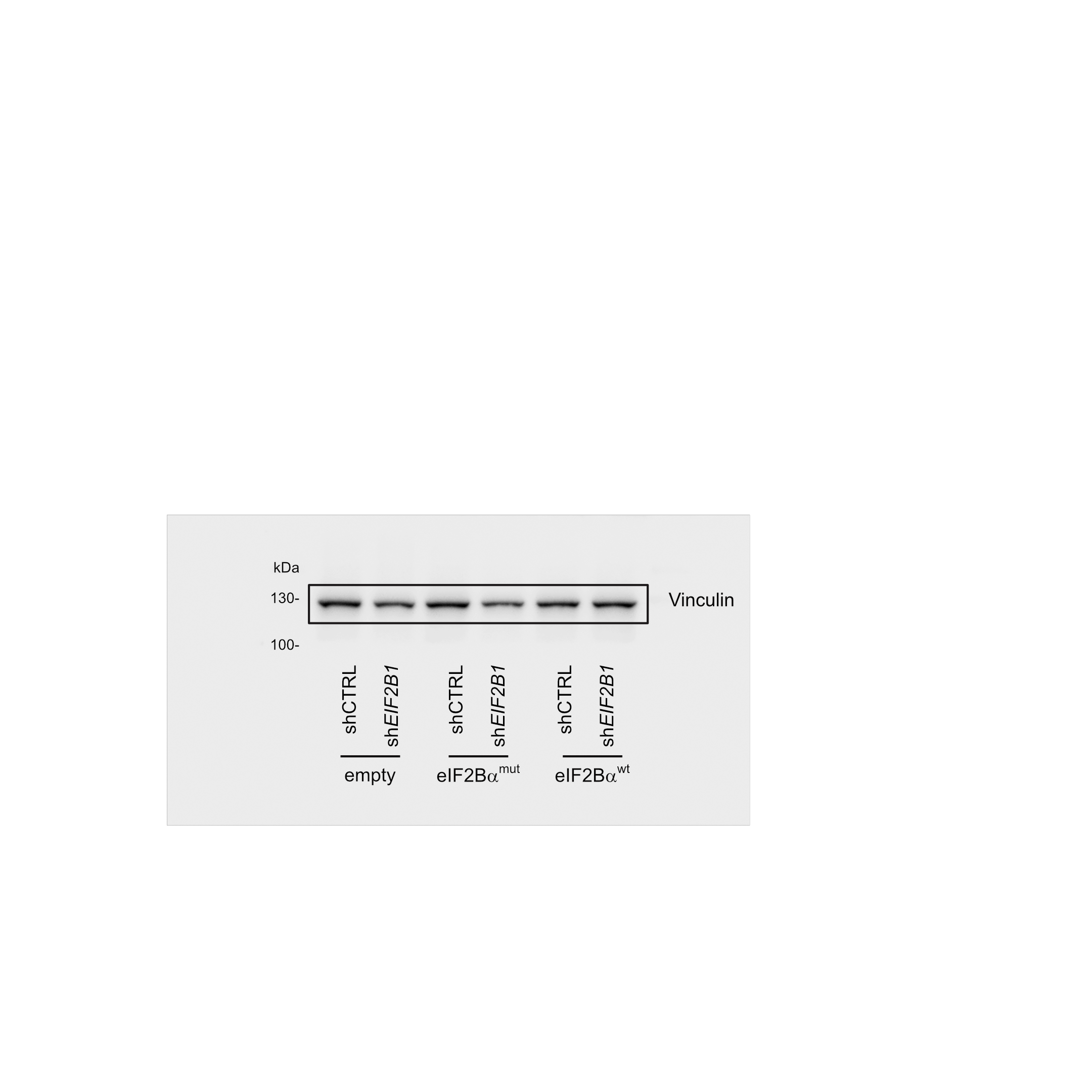

Supplement: Supplementary file 9 — Source data Fig. 3 [file 44318_2025_381_MOESM9_ESM.zip › Figure 3/3D/western vinculin.tiff]

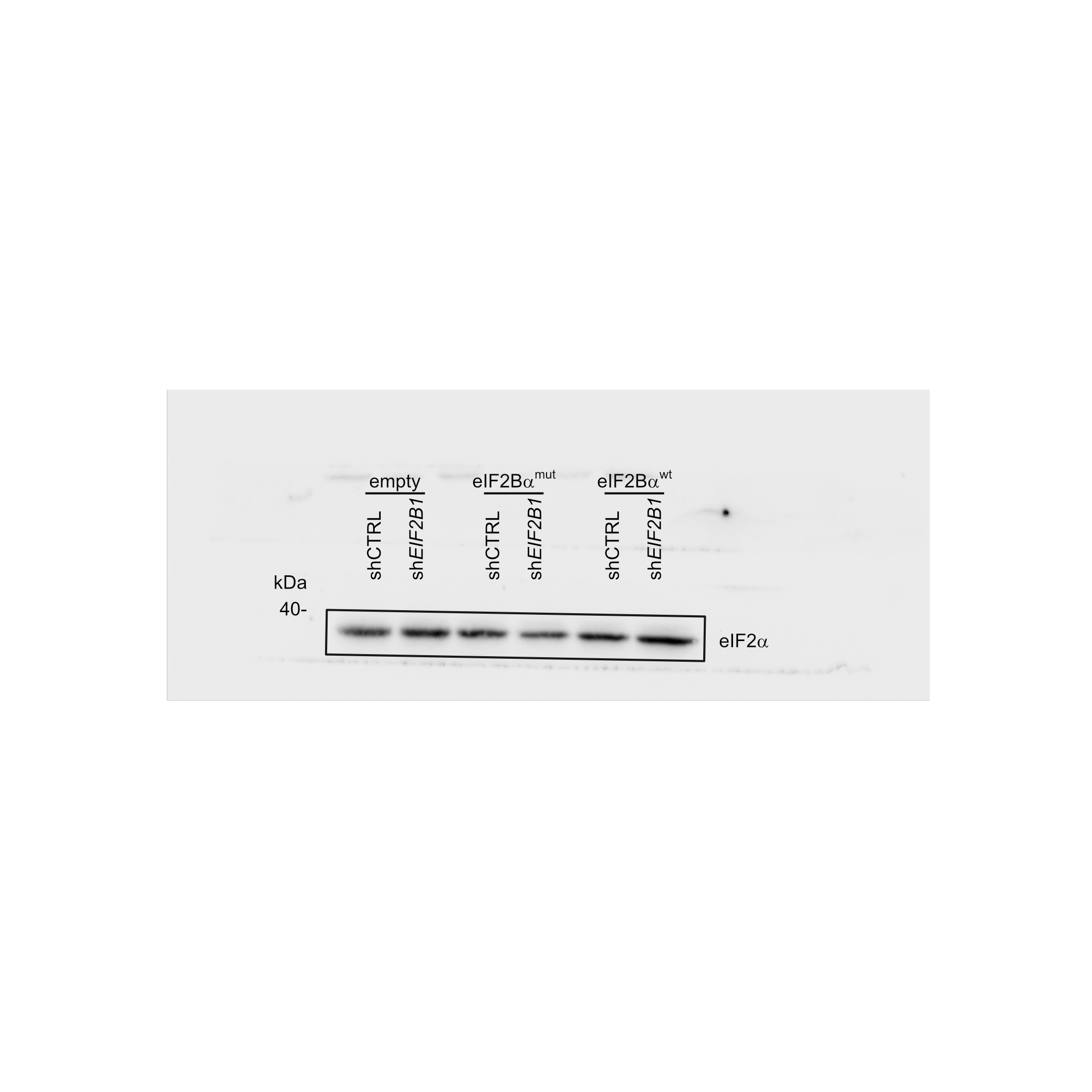

Supplement: Supplementary file 9 — Source data Fig. 3 [file 44318_2025_381_MOESM9_ESM.zip › Figure 3/3D/western eIF2a.tiff]

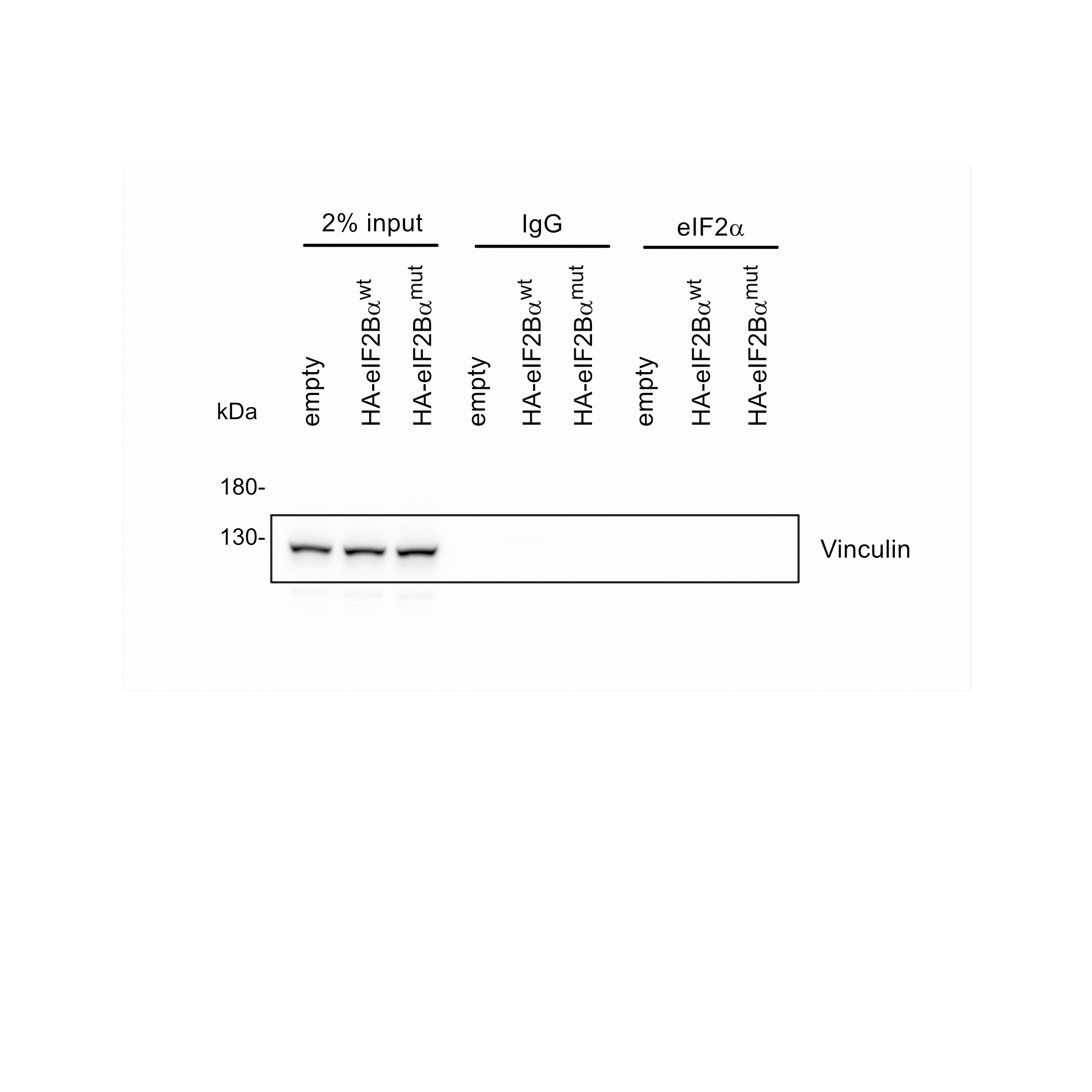

Supplement: Supplementary file 9 — Source data Fig. 3 [file 44318_2025_381_MOESM9_ESM.zip › Figure 3/3H/western vinculin.Tif]

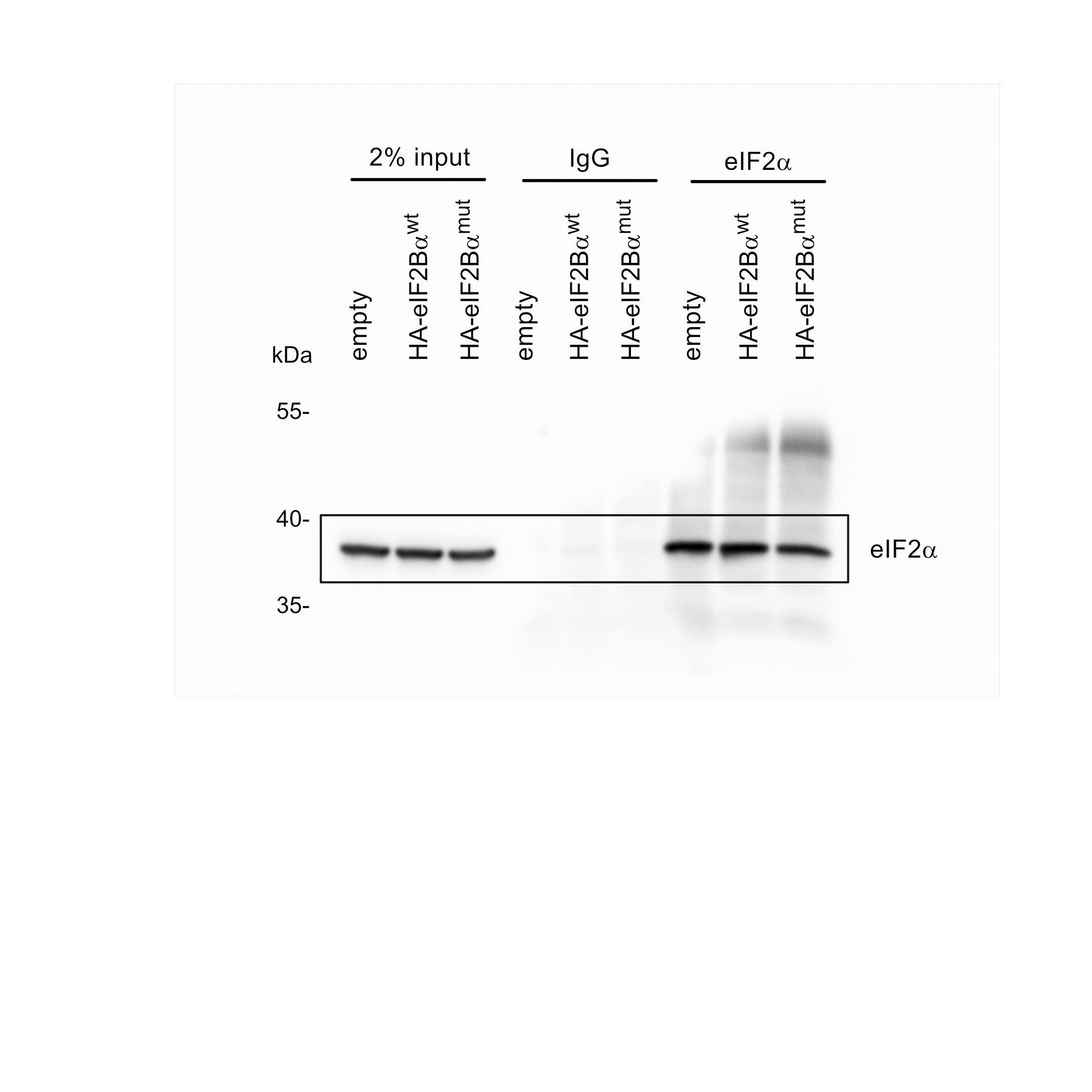

Supplement: Supplementary file 9 — Source data Fig. 3 [file 44318_2025_381_MOESM9_ESM.zip › Figure 3/3H/western eIF2a.tiff]

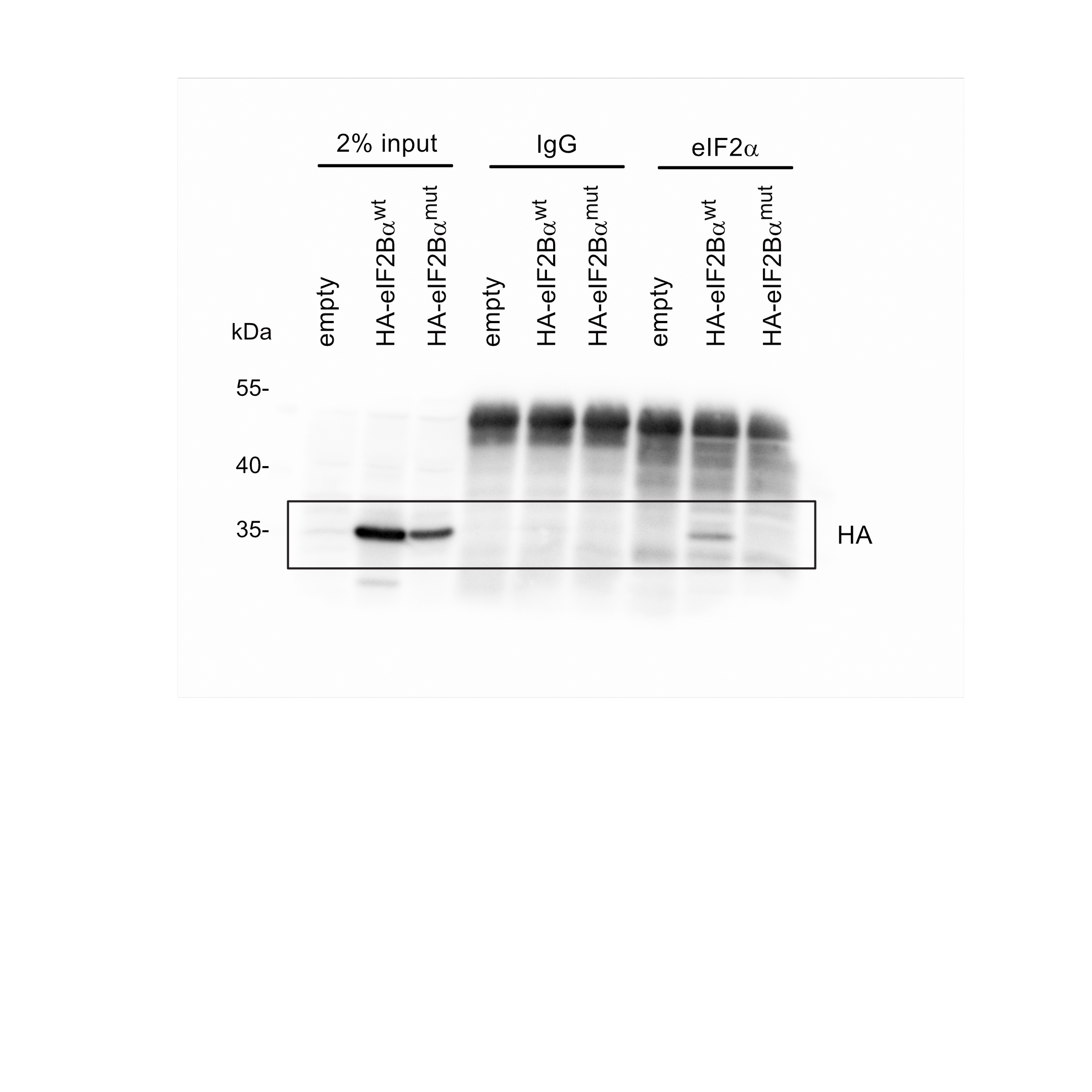

Supplement: Supplementary file 9 — Source data Fig. 3 [file 44318_2025_381_MOESM9_ESM.zip › Figure 3/3H/western HA.Tif]

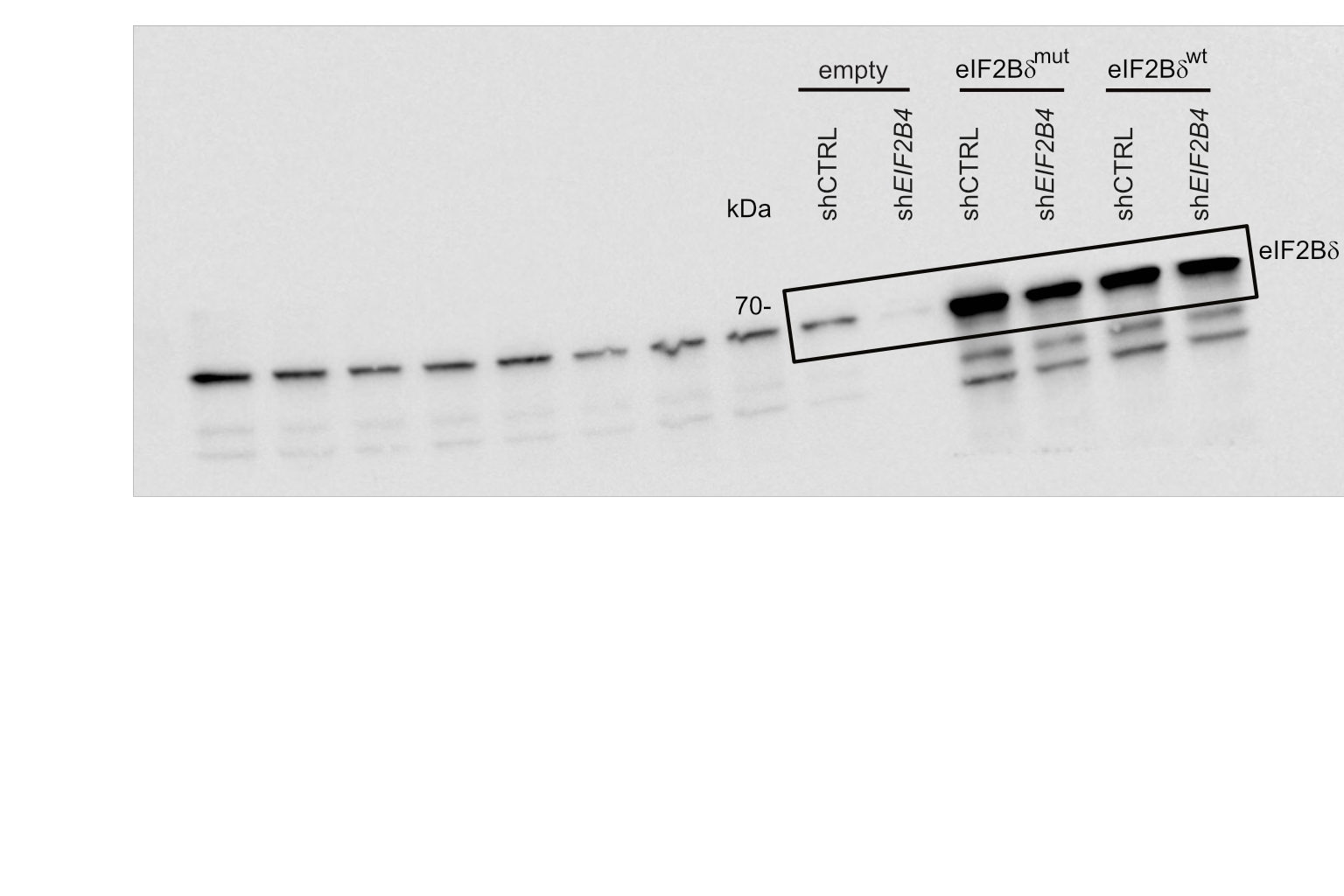

Supplement: Supplementary file 10 — Source data Fig. 4 [file 44318_2025_381_MOESM10_ESM.zip › Source data_Figure 4/Figure 4/4B/western eIF2Bd.tif]

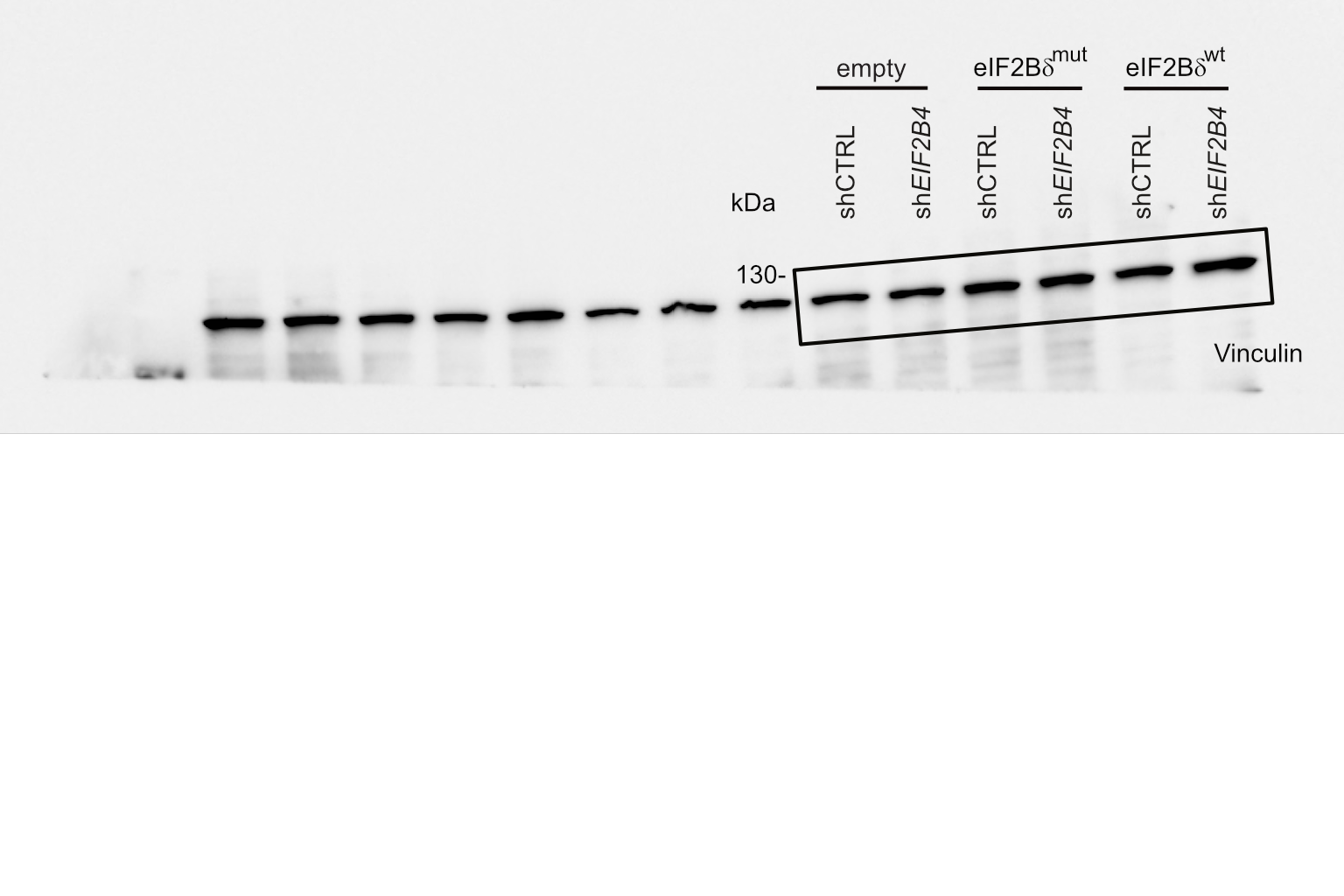

Supplement: Supplementary file 10 — Source data Fig. 4 [file 44318_2025_381_MOESM10_ESM.zip › Source data_Figure 4/Figure 4/4B/western vinculin.tif]

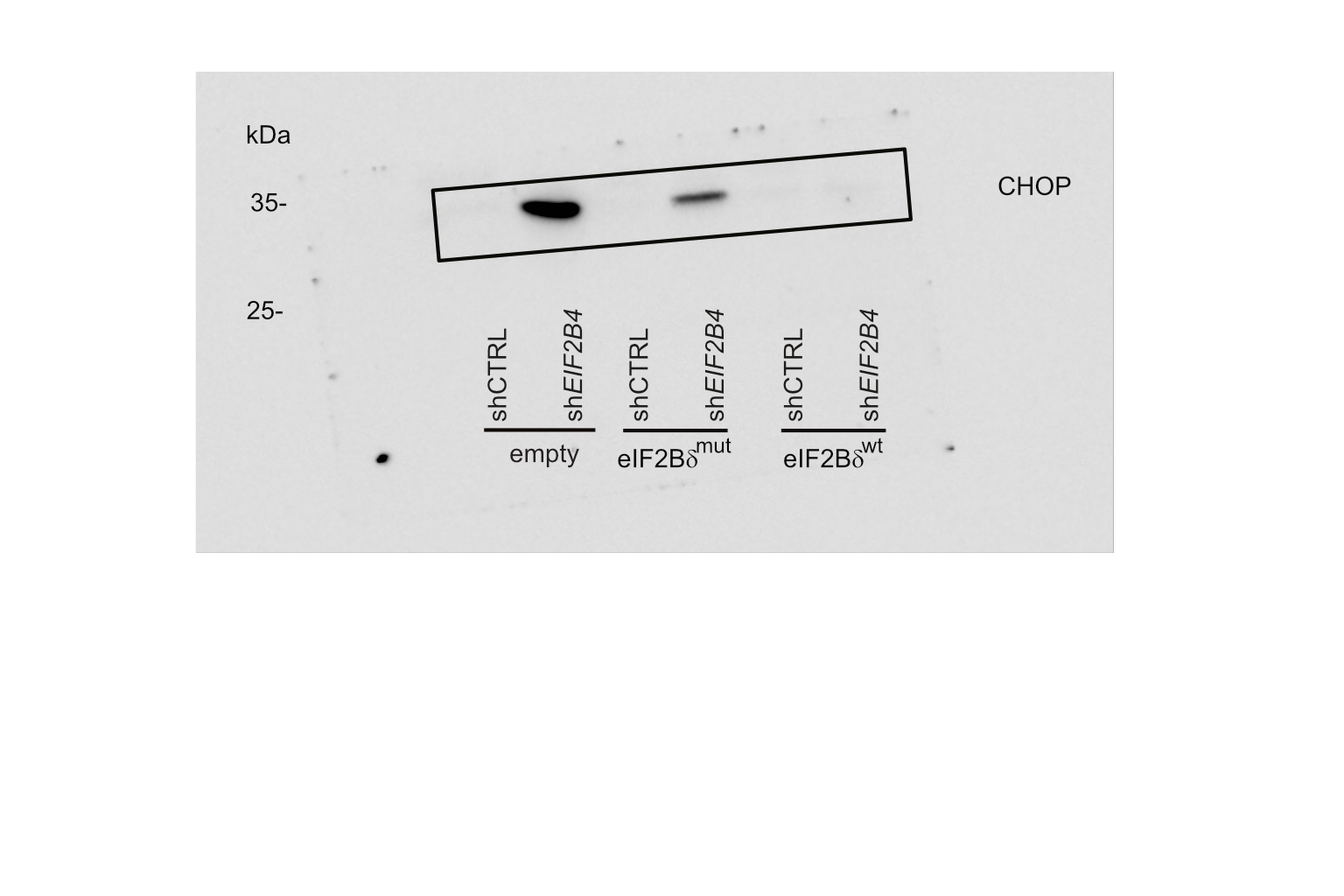

Supplement: Supplementary file 10 — Source data Fig. 4 [file 44318_2025_381_MOESM10_ESM.zip › Source data_Figure 4/Figure 4/4D/western chop.tif]

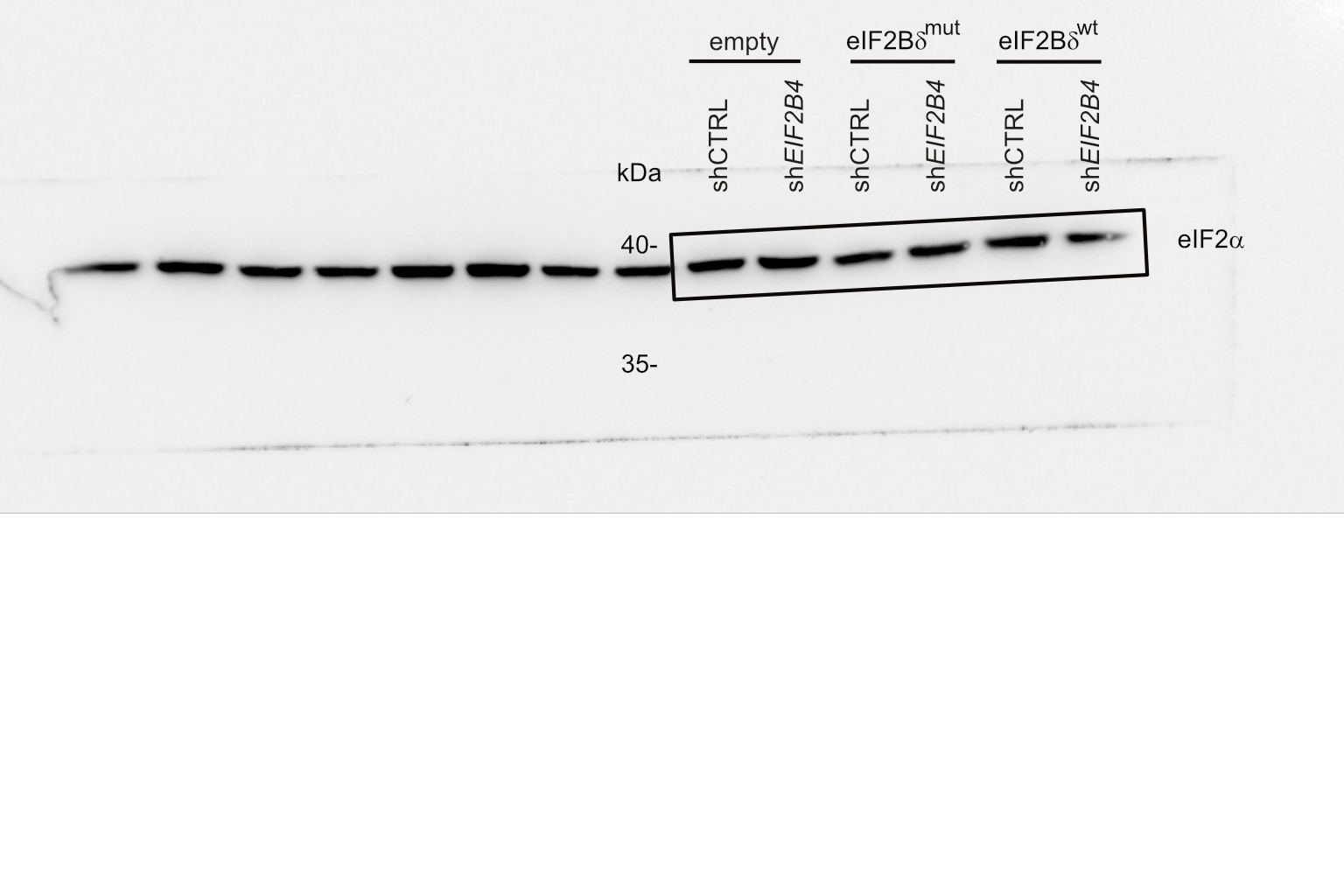

Supplement: Supplementary file 10 — Source data Fig. 4 [file 44318_2025_381_MOESM10_ESM.zip › Source data_Figure 4/Figure 4/4D/western eIF2a.tif]

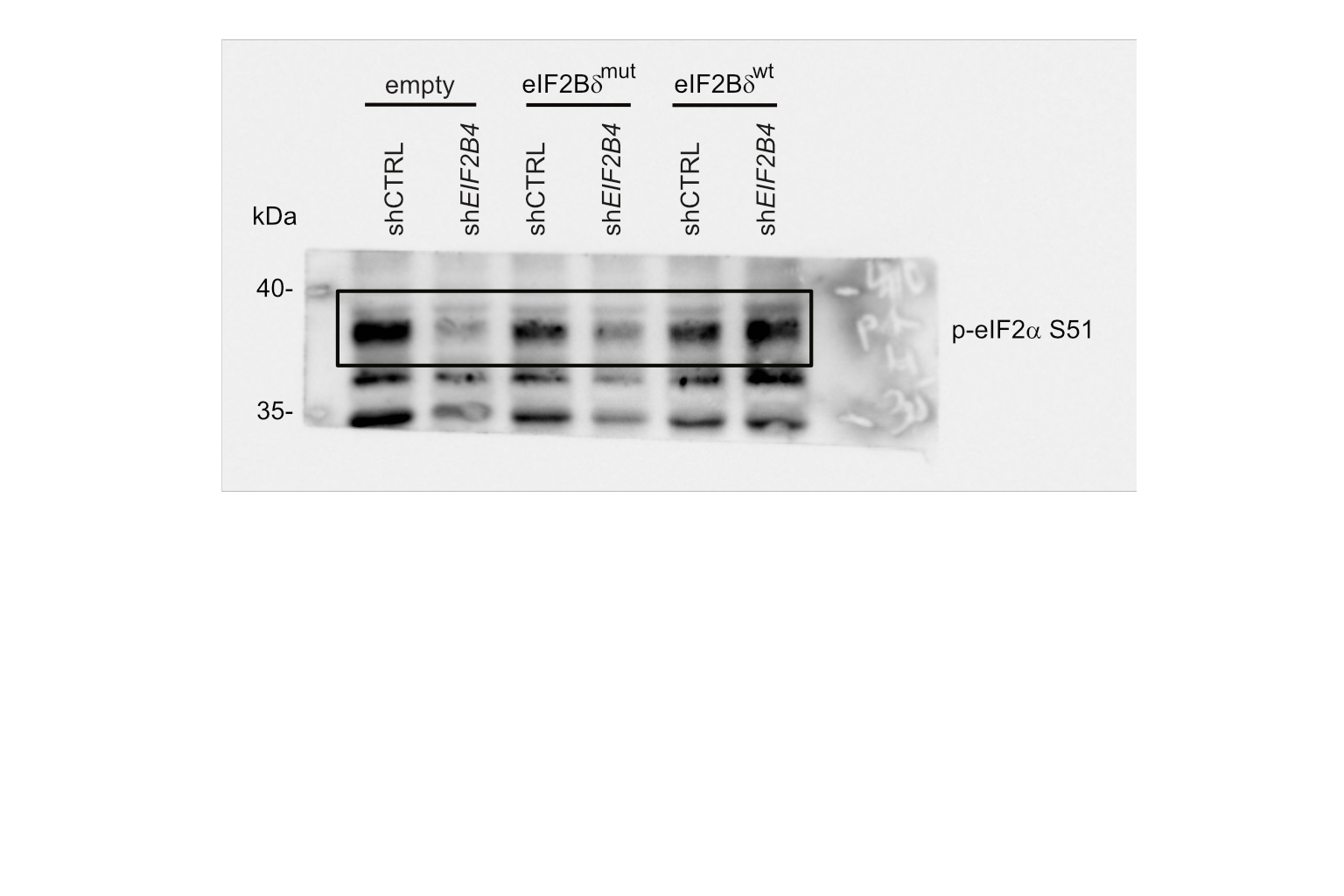

Supplement: Supplementary file 10 — Source data Fig. 4 [file 44318_2025_381_MOESM10_ESM.zip › Source data_Figure 4/Figure 4/4D/western p-eIF2a S51.tif]

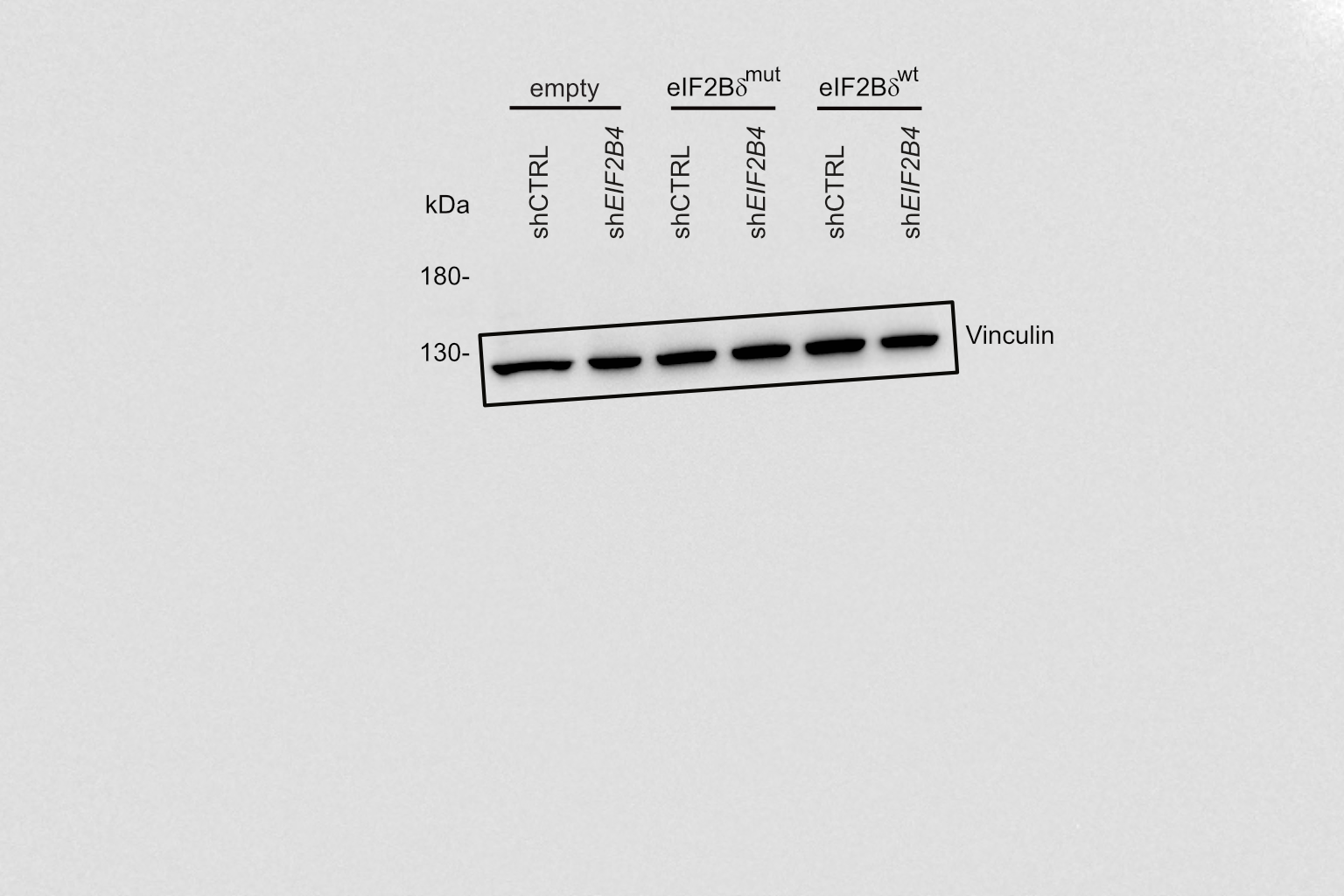

Supplement: Supplementary file 10 — Source data Fig. 4 [file 44318_2025_381_MOESM10_ESM.zip › Source data_Figure 4/Figure 4/4D/western vinculin.tiff]

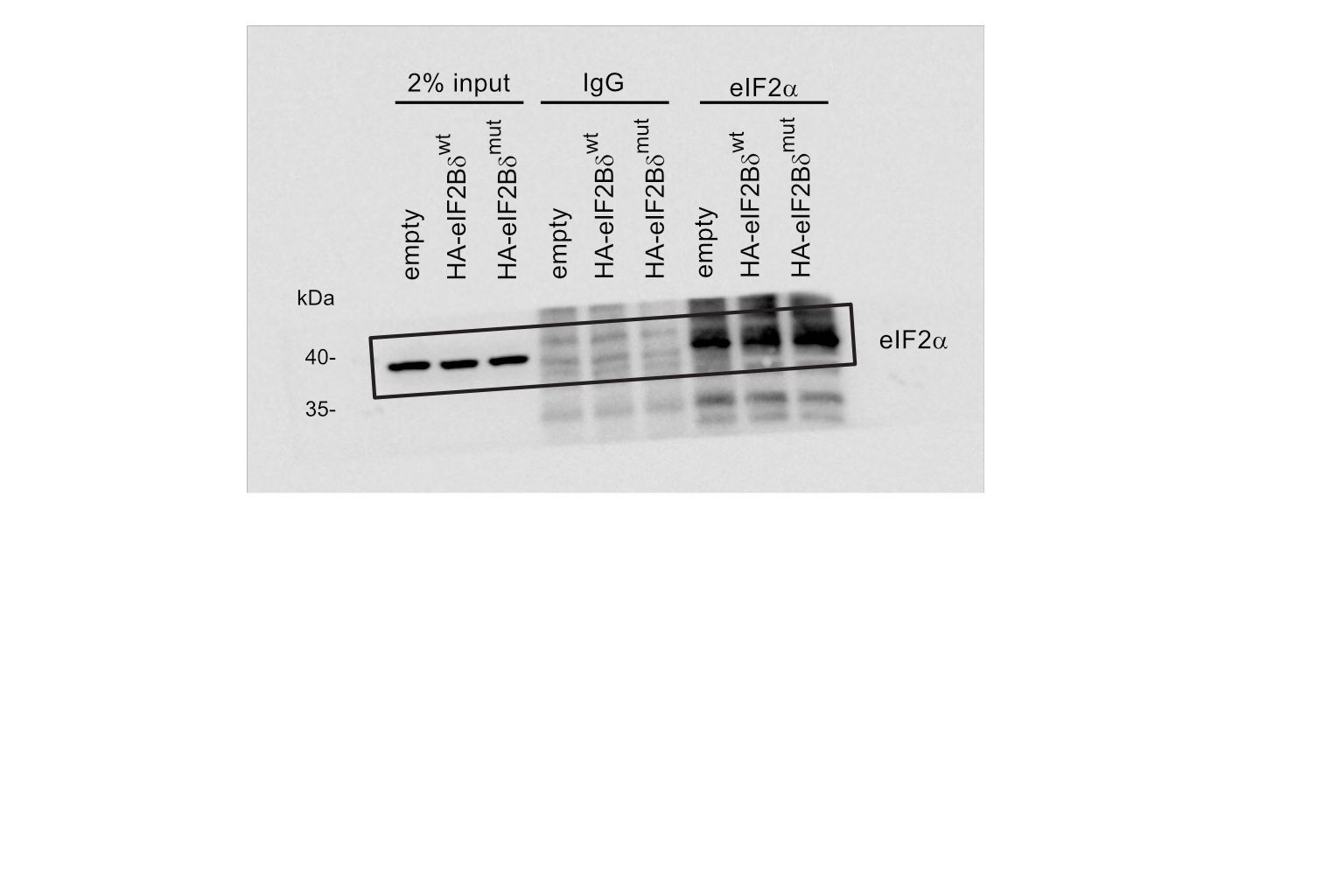

Supplement: Supplementary file 10 — Source data Fig. 4 [file 44318_2025_381_MOESM10_ESM.zip › Source data_Figure 4/Figure 4/4H/western eIF2a.tif]

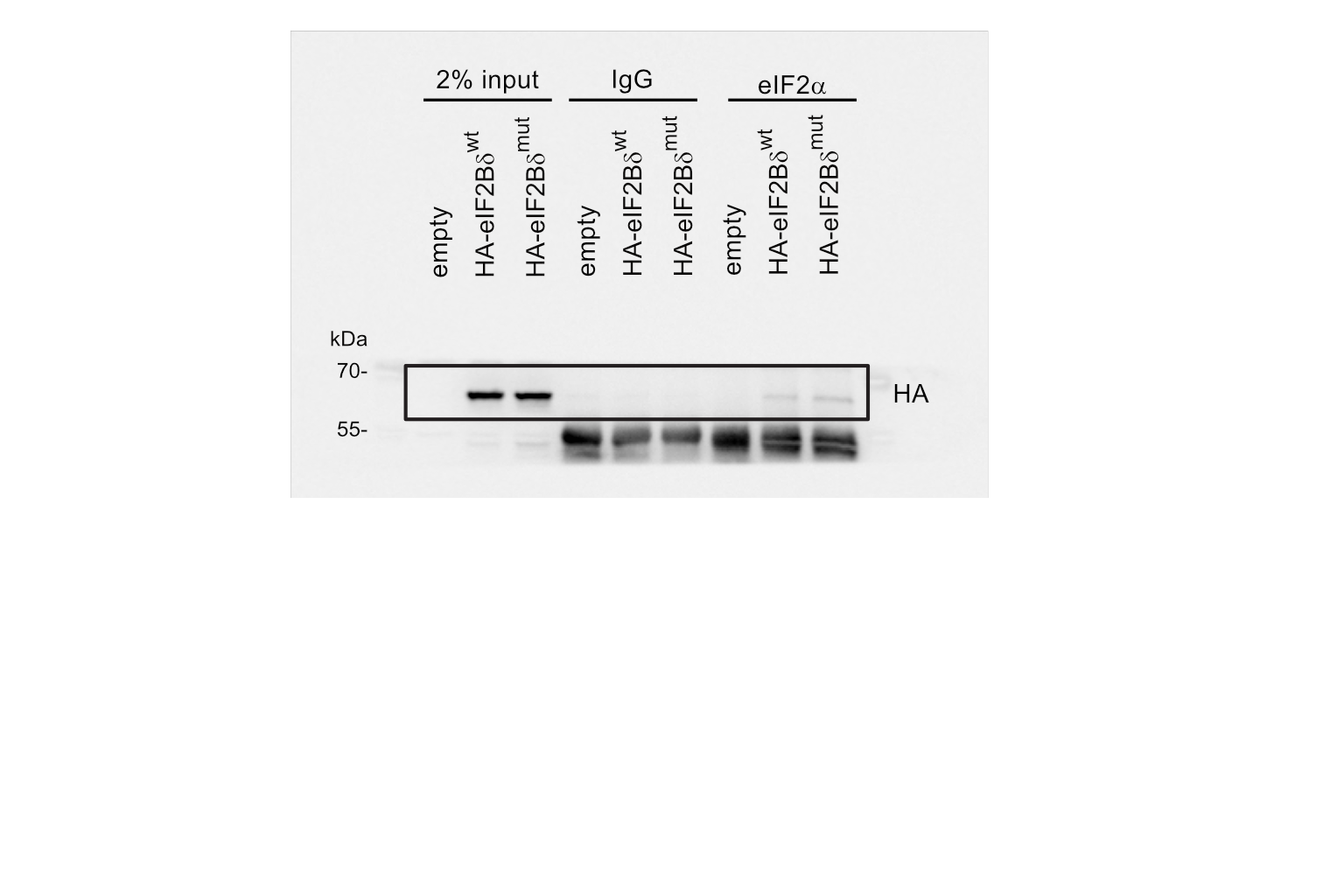

Supplement: Supplementary file 10 — Source data Fig. 4 [file 44318_2025_381_MOESM10_ESM.zip › Source data_Figure 4/Figure 4/4H/western HA.tif]

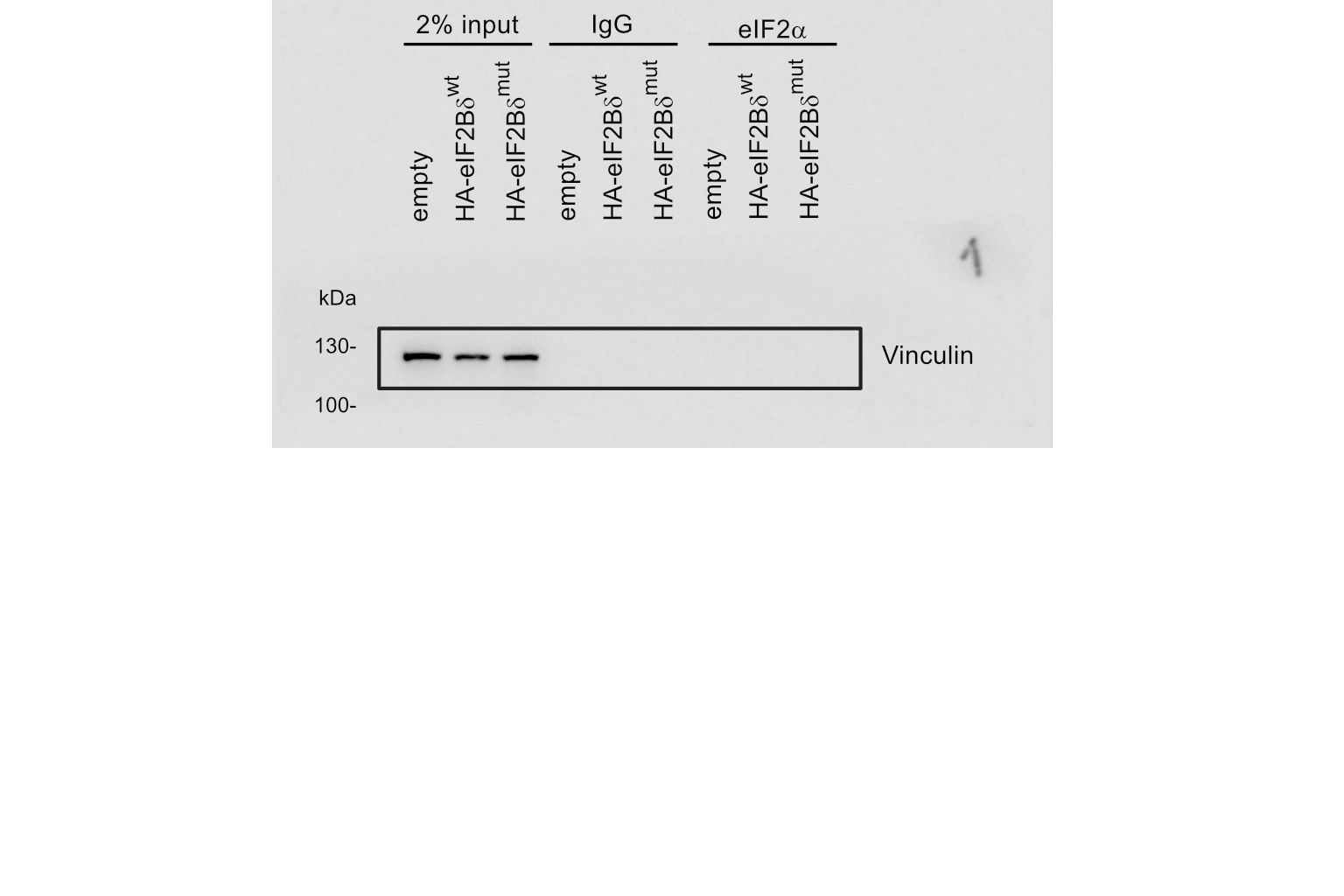

Supplement: Supplementary file 10 — Source data Fig. 4 [file 44318_2025_381_MOESM10_ESM.zip › Source data_Figure 4/Figure 4/4H/western vinculin.tif]

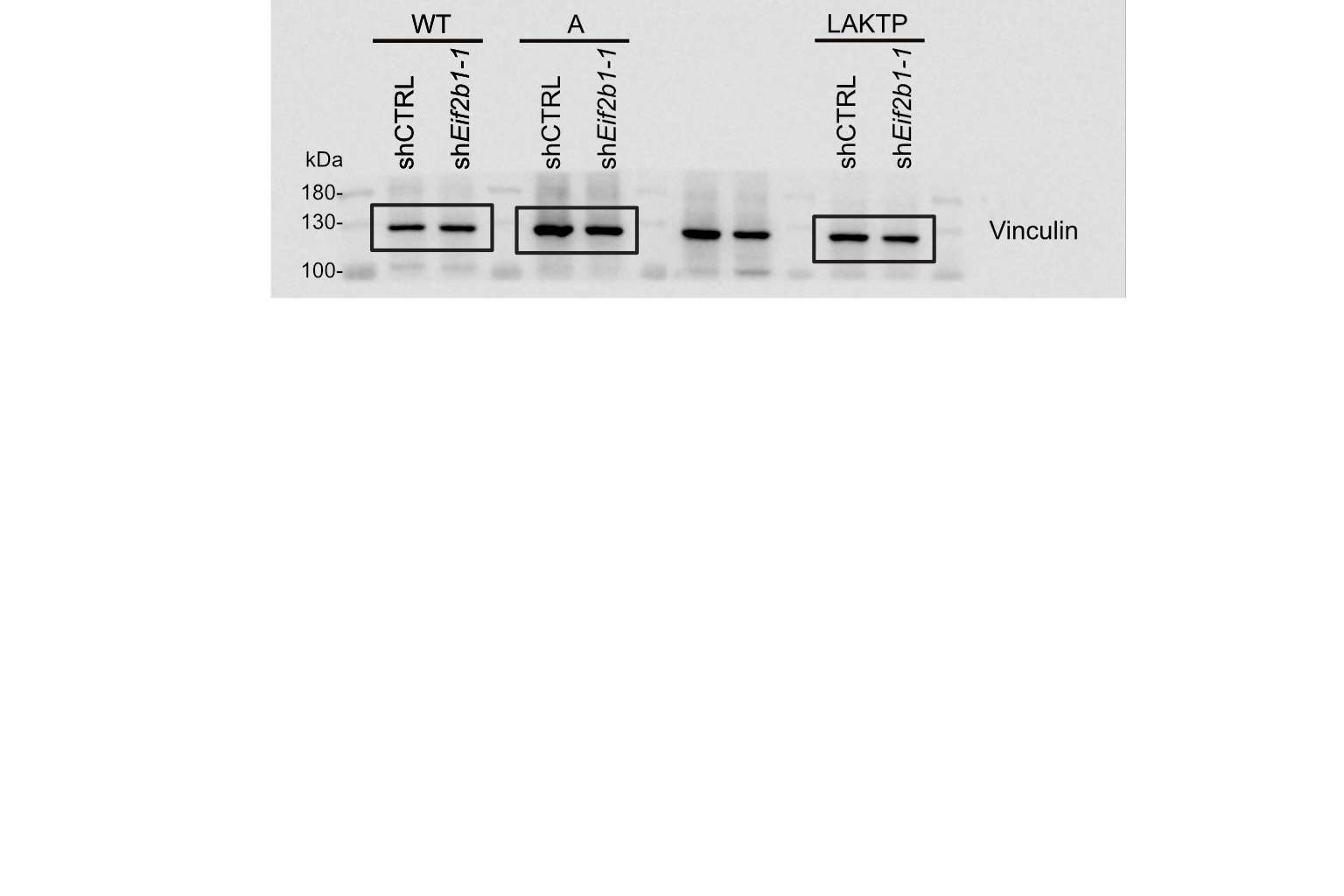

Supplement: Supplementary file 11 — Source data Fig. 6 [file 44318_2025_381_MOESM11_ESM.zip › Figure 6/6C/western vinculin WT,A,LAKTP.tif]

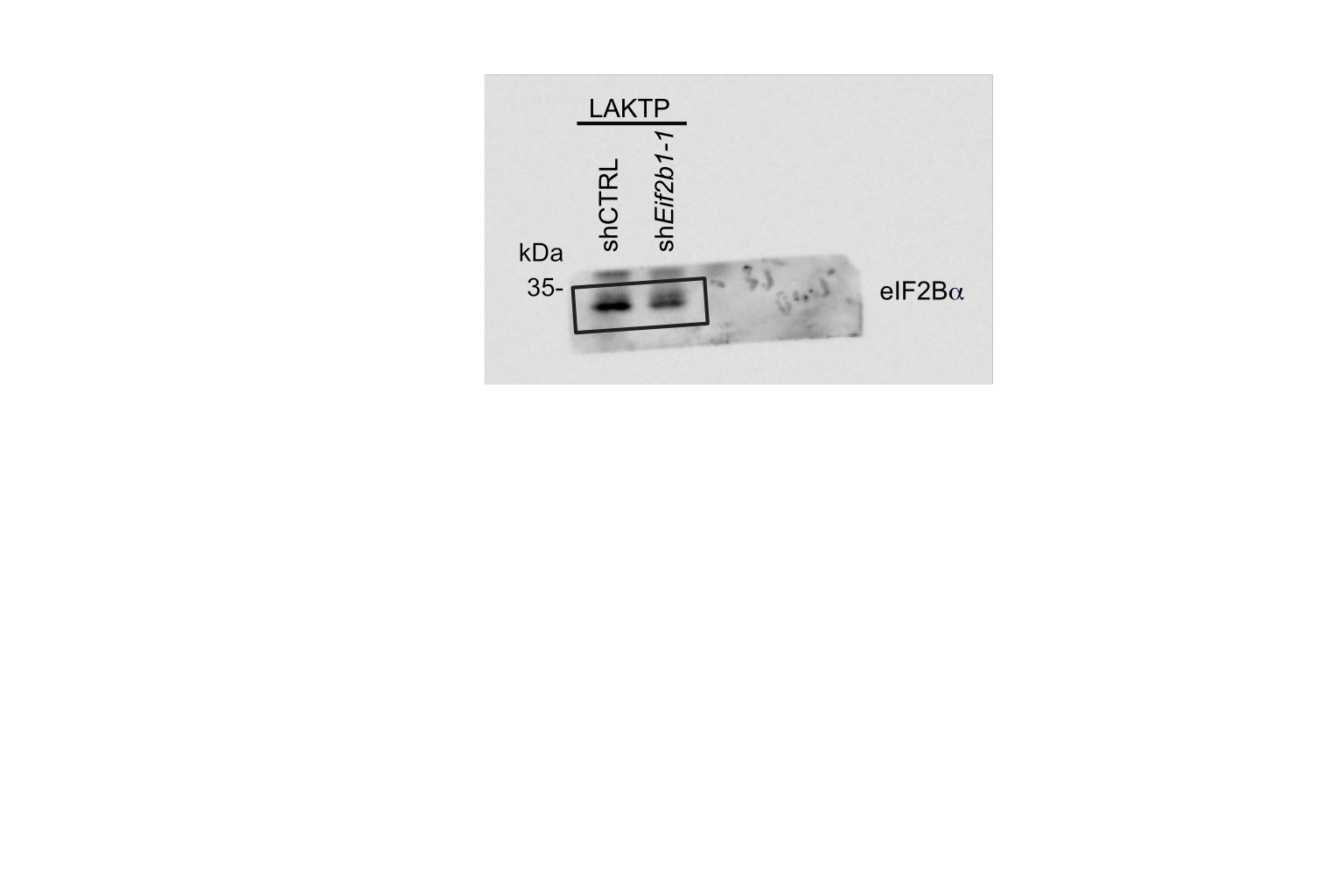

Supplement: Supplementary file 11 — Source data Fig. 6 [file 44318_2025_381_MOESM11_ESM.zip › Figure 6/6C/western eIF2Ba LAKTP.tif]

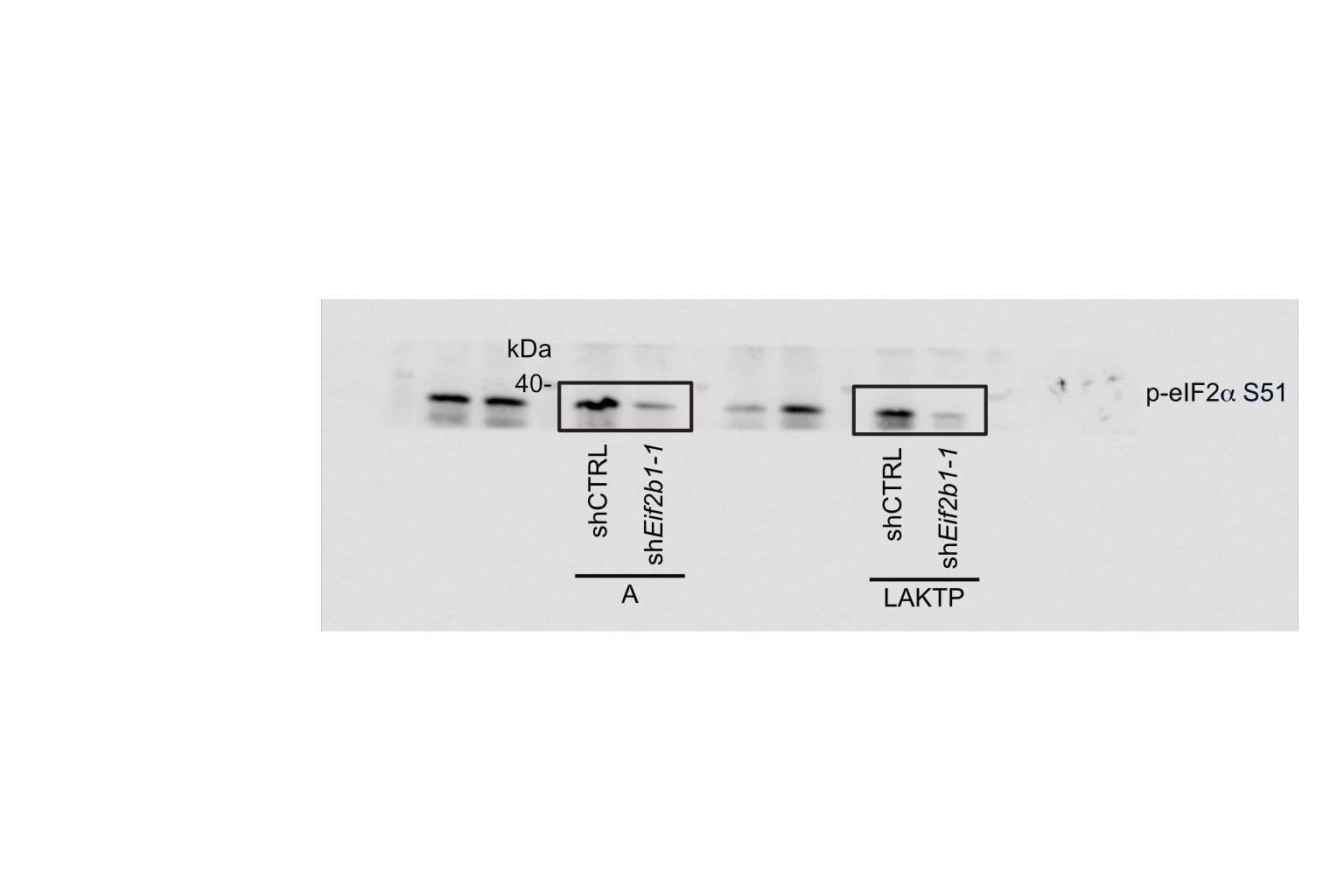

Supplement: Supplementary file 11 — Source data Fig. 6 [file 44318_2025_381_MOESM11_ESM.zip › Figure 6/6C/western p-eIF2a S51 A,LAKTP.tif]

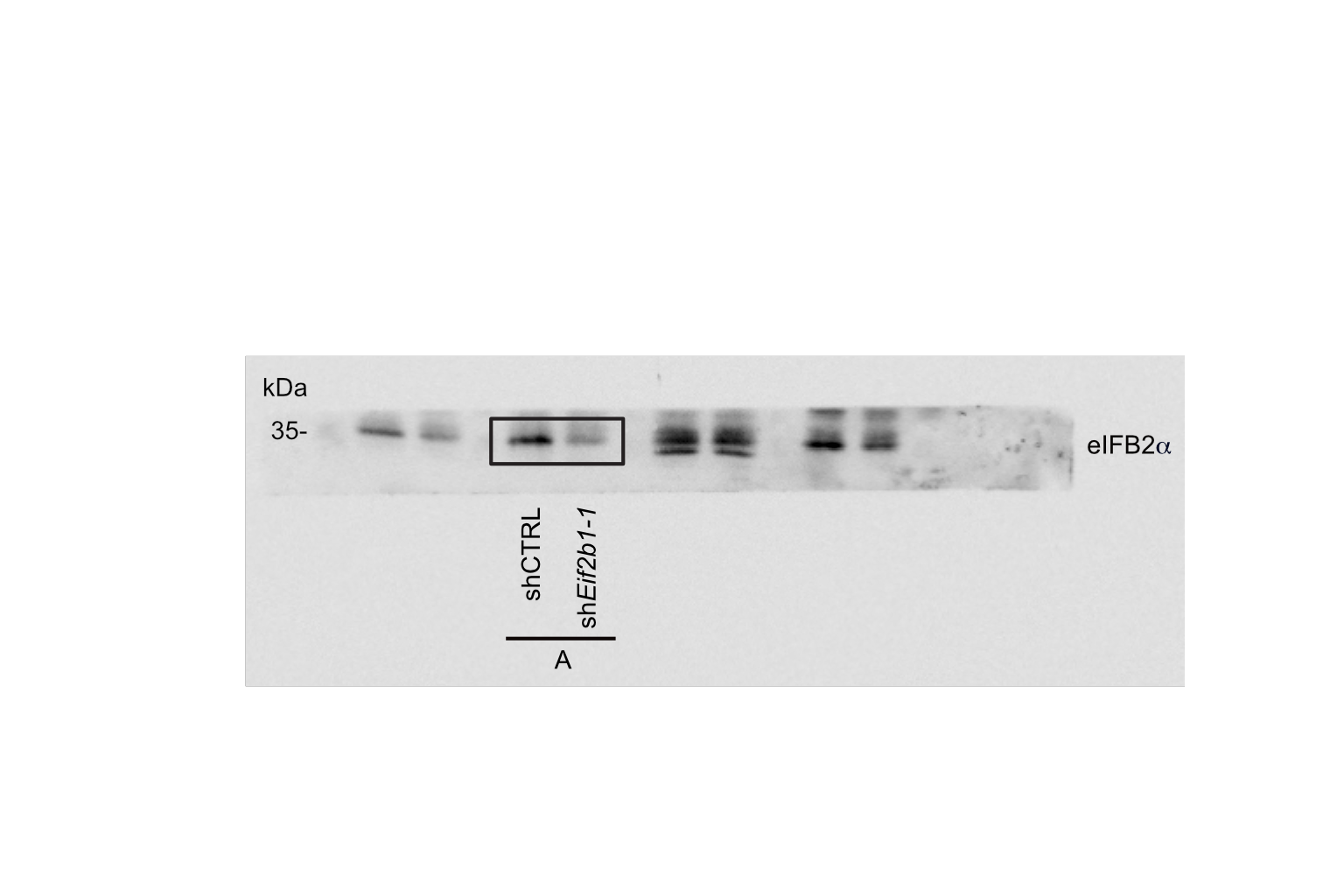

Supplement: Supplementary file 11 — Source data Fig. 6 [file 44318_2025_381_MOESM11_ESM.zip › Figure 6/6C/western eIF2Ba A.tif]

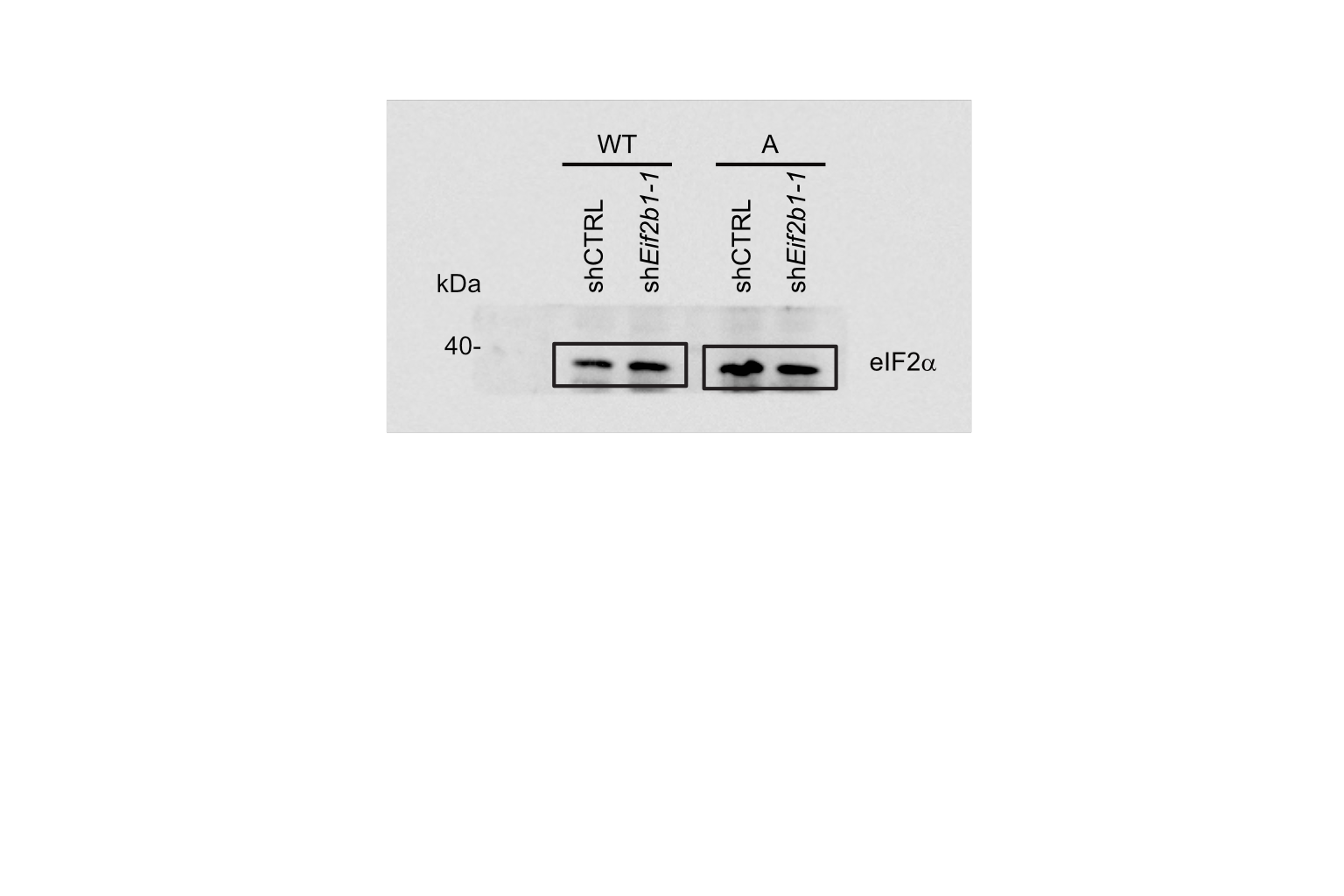

Supplement: Supplementary file 11 — Source data Fig. 6 [file 44318_2025_381_MOESM11_ESM.zip › Figure 6/6C/western eIF2a WT,A.tif]

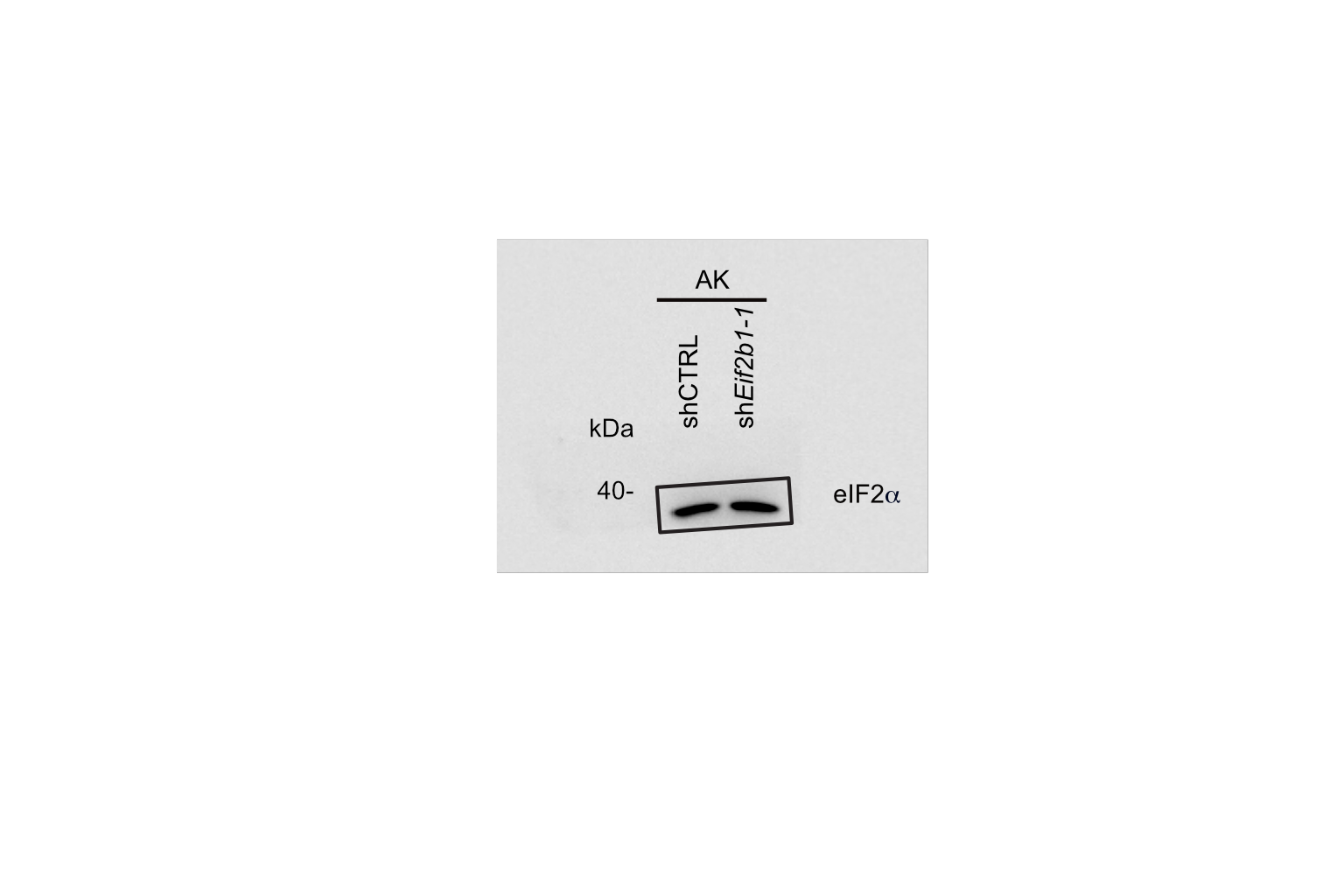

Supplement: Supplementary file 11 — Source data Fig. 6 [file 44318_2025_381_MOESM11_ESM.zip › Figure 6/6C/western eIF2a AK.tif]

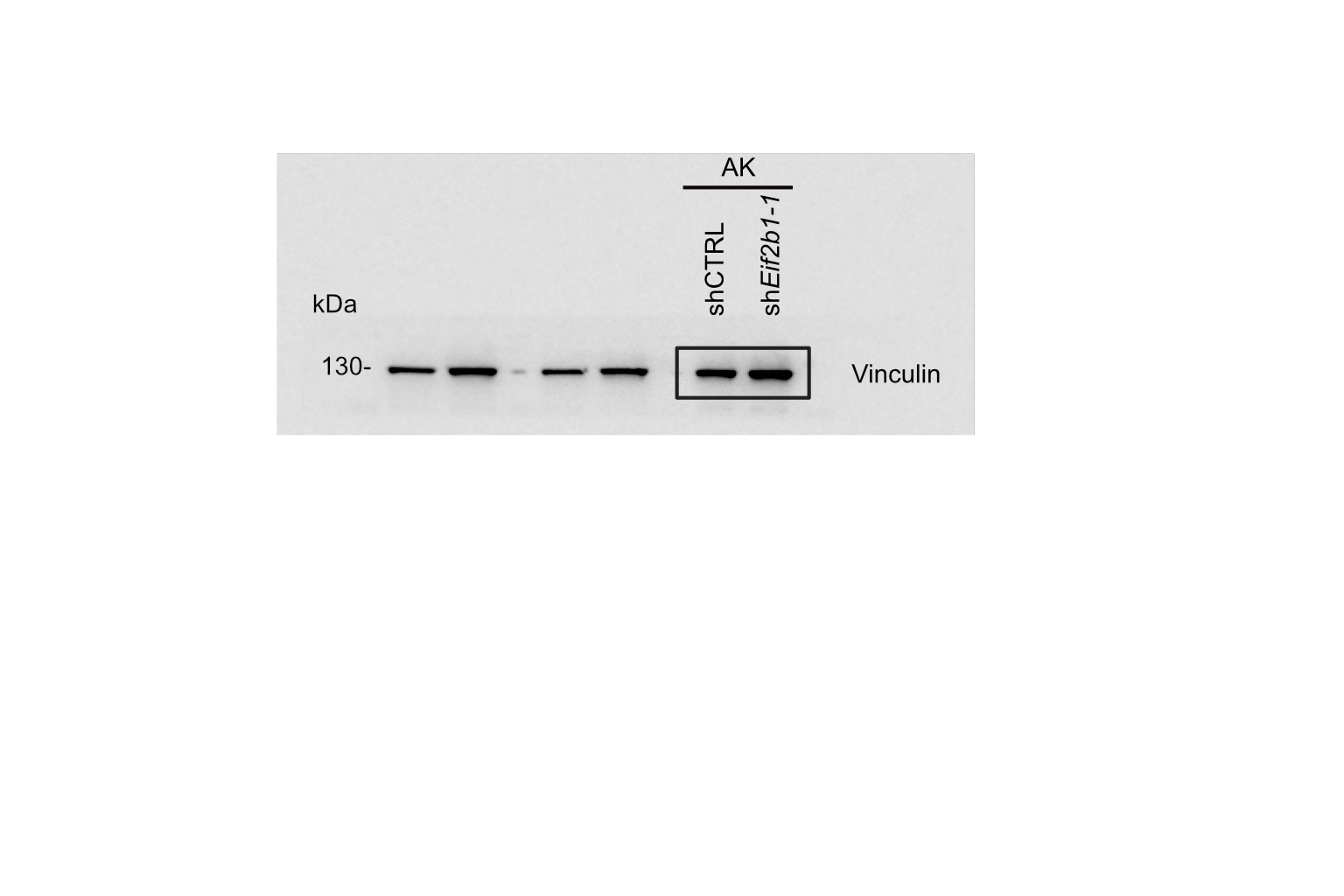

Supplement: Supplementary file 11 — Source data Fig. 6 [file 44318_2025_381_MOESM11_ESM.zip › Figure 6/6C/western vinculin AK.tif]

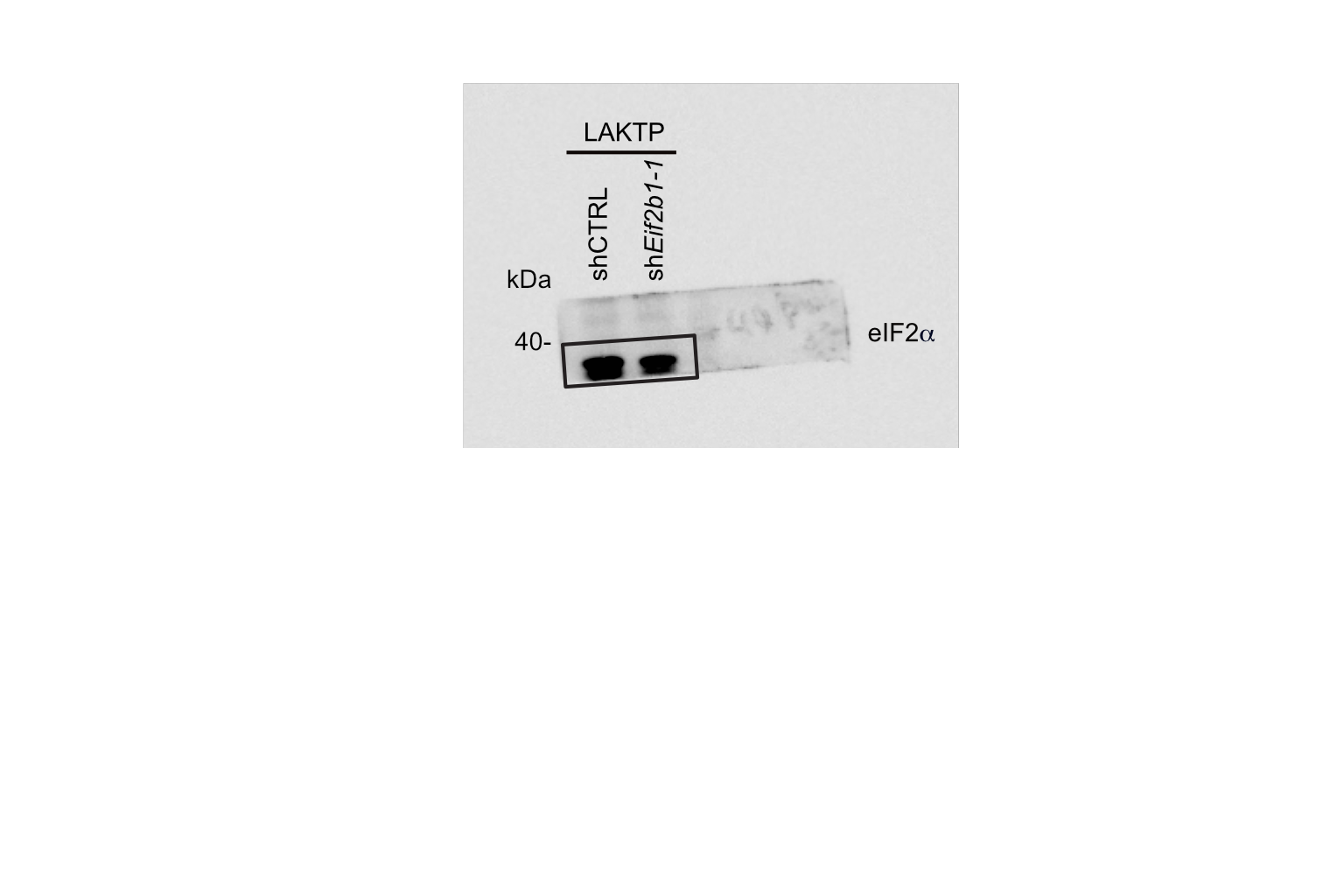

Supplement: Supplementary file 11 — Source data Fig. 6 [file 44318_2025_381_MOESM11_ESM.zip › Figure 6/6C/western eIF2a LAKTP.tif]

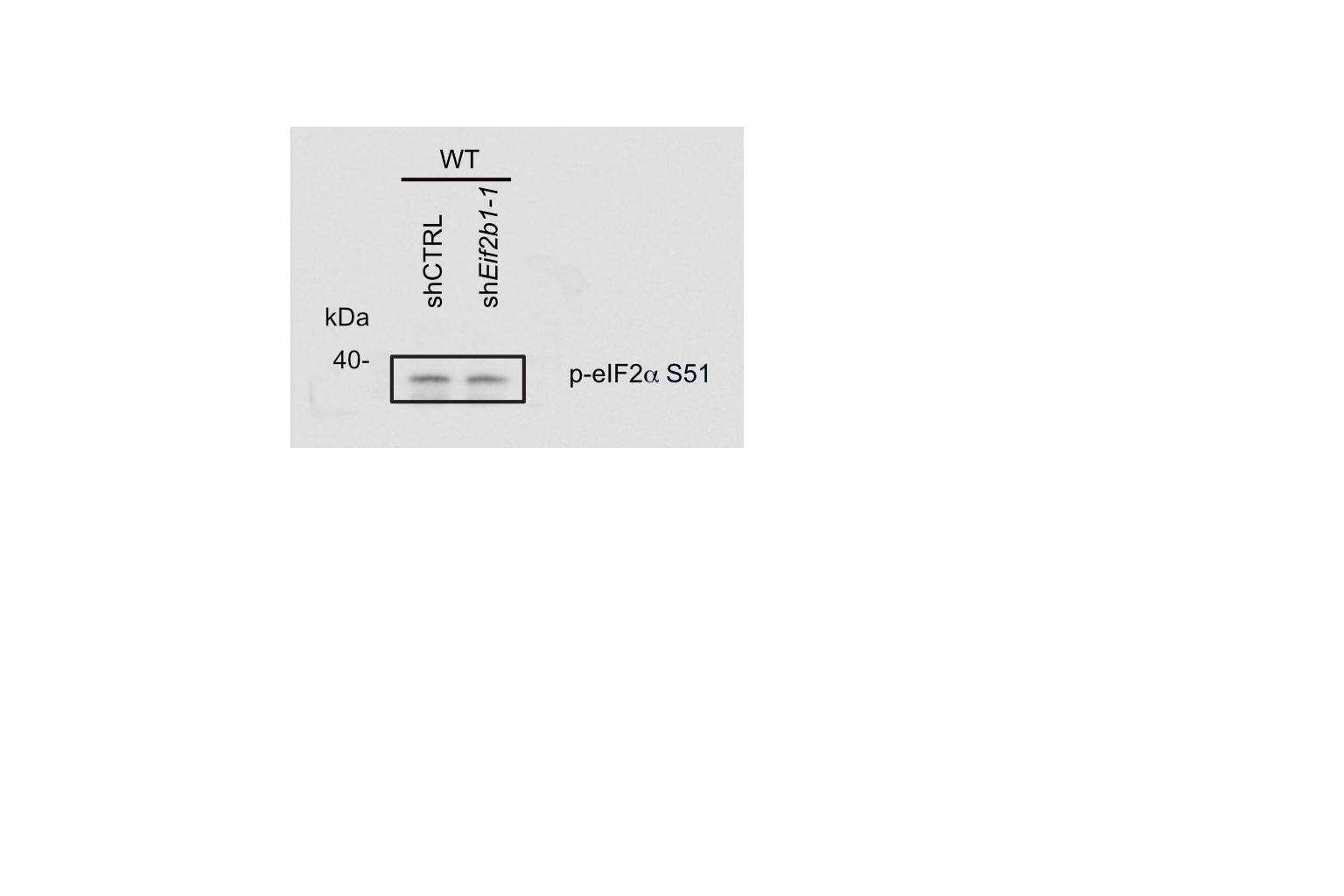

Supplement: Supplementary file 11 — Source data Fig. 6 [file 44318_2025_381_MOESM11_ESM.zip › Figure 6/6C/western p-eIF2a S51 WT.tif]

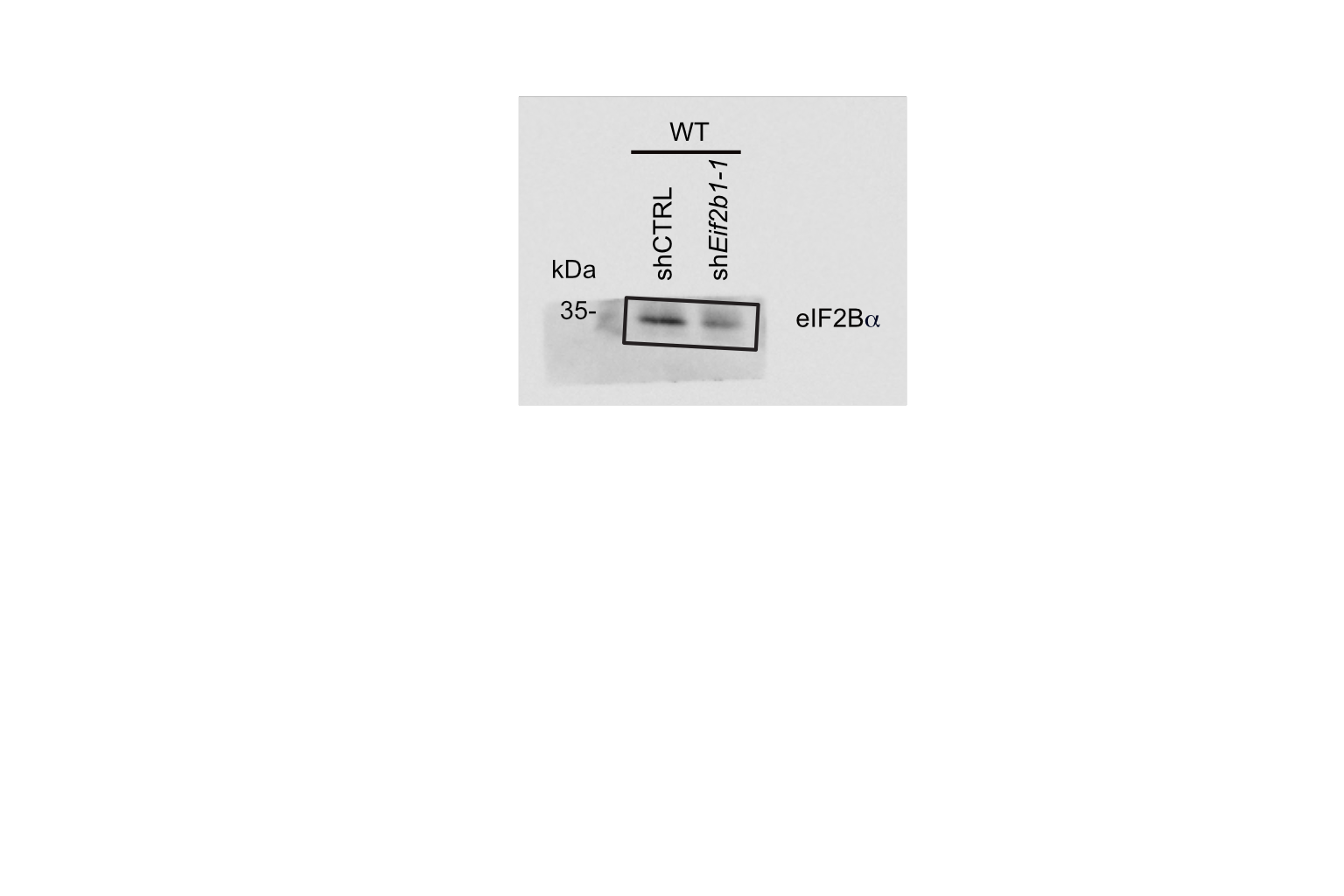

Supplement: Supplementary file 11 — Source data Fig. 6 [file 44318_2025_381_MOESM11_ESM.zip › Figure 6/6C/western eIF2Ba WT.tif]

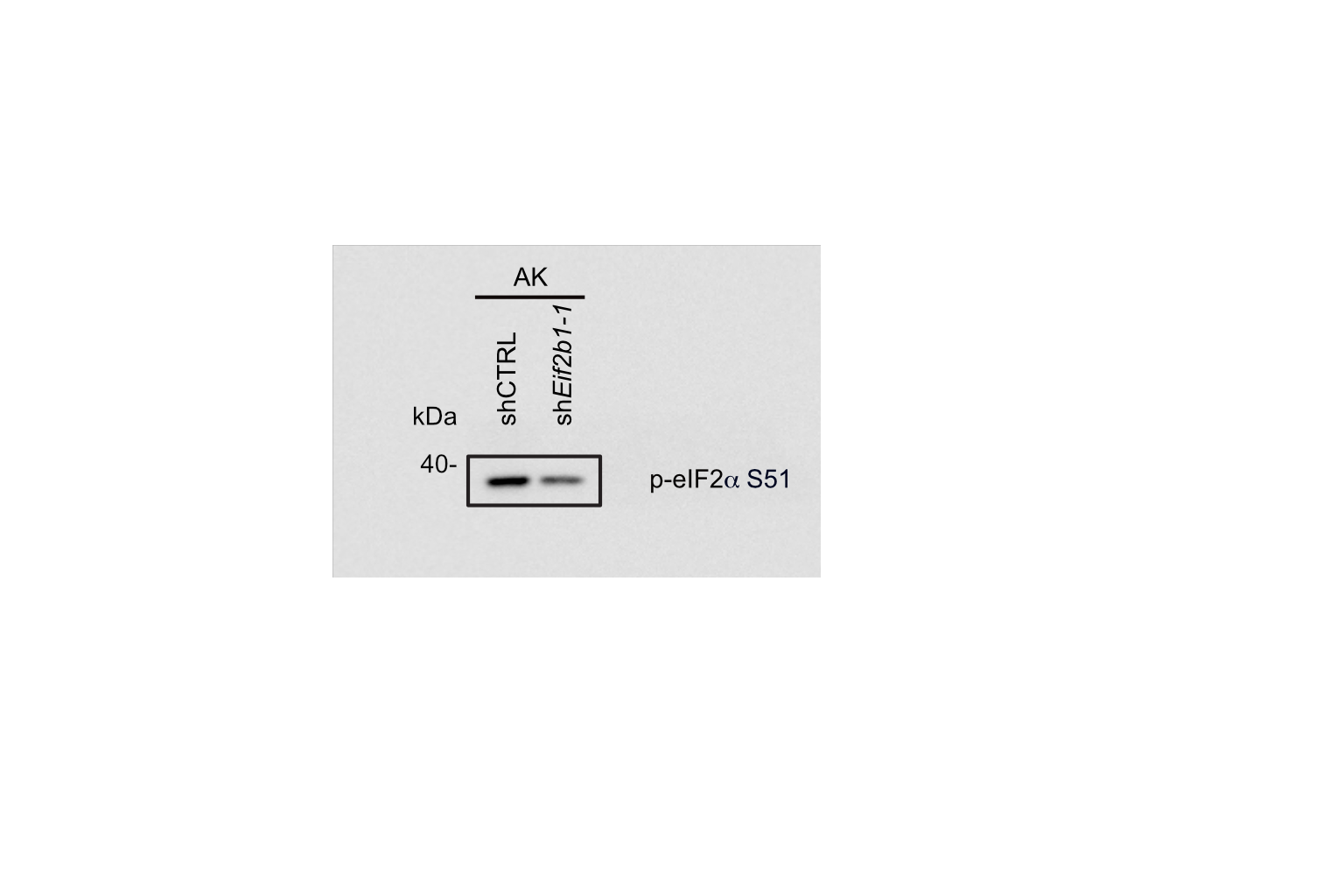

Supplement: Supplementary file 11 — Source data Fig. 6 [file 44318_2025_381_MOESM11_ESM.zip › Figure 6/6C/western p-eIF2a S51 AK.tif]

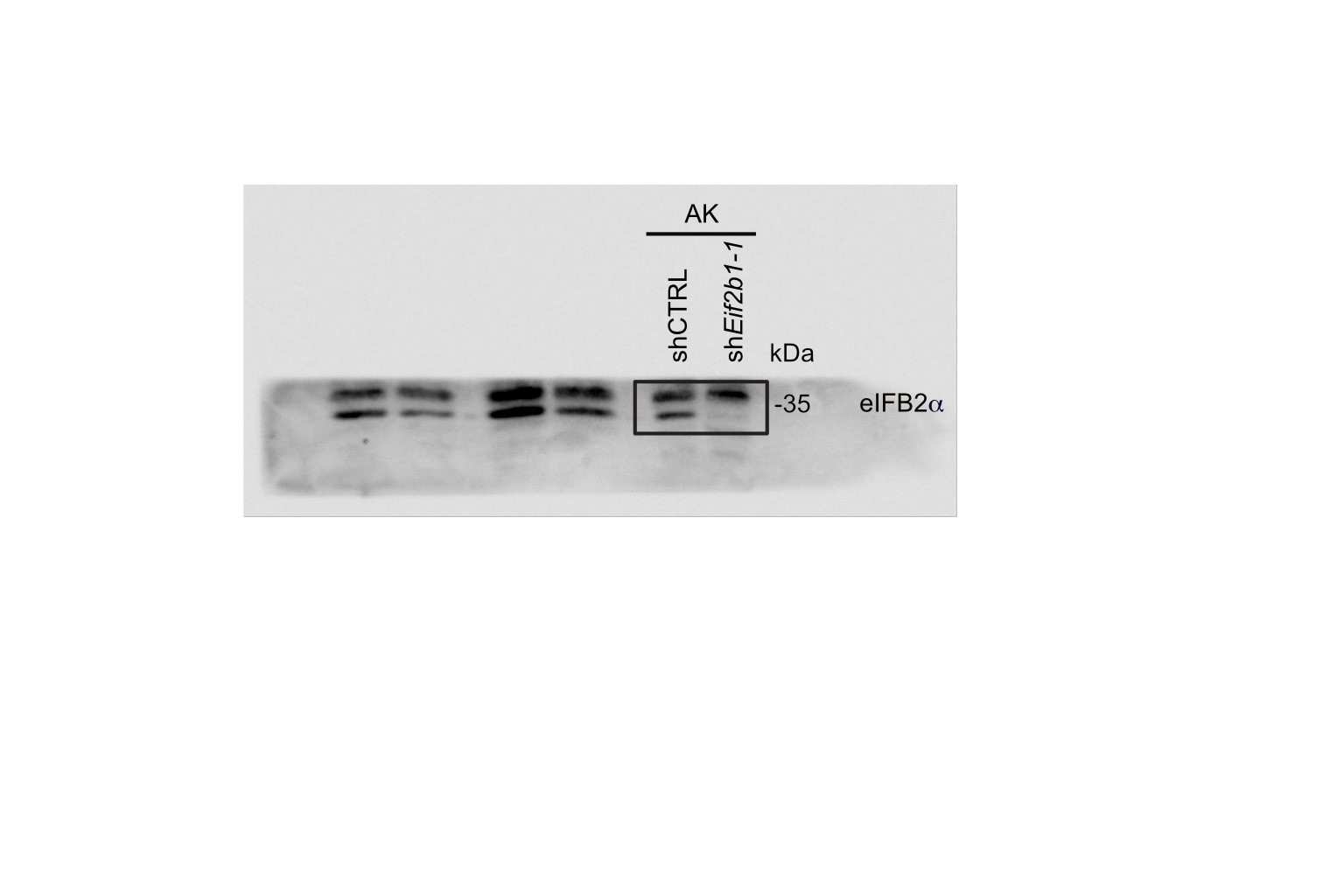

Supplement: Supplementary file 11 — Source data Fig. 6 [file 44318_2025_381_MOESM11_ESM.zip › Figure 6/6C/western eIF2Ba AK.tif]

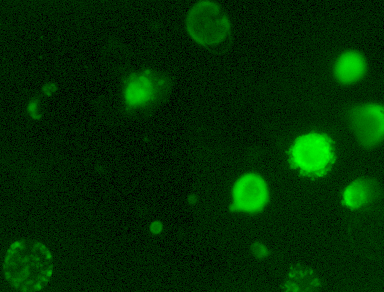

Supplement: Supplementary file 11 — Source data Fig. 6 [file 44318_2025_381_MOESM11_ESM.zip › Figure 6/6A/LAKTP/LAKTP_shEif2b1-1.png]

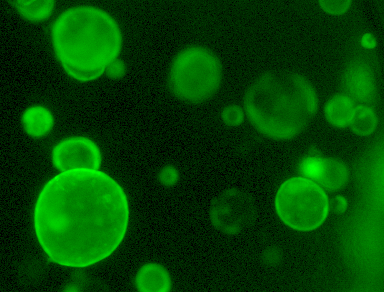

Supplement: Supplementary file 11 — Source data Fig. 6 [file 44318_2025_381_MOESM11_ESM.zip › Figure 6/6A/LAKTP/LAKTP_shCTRL.png]

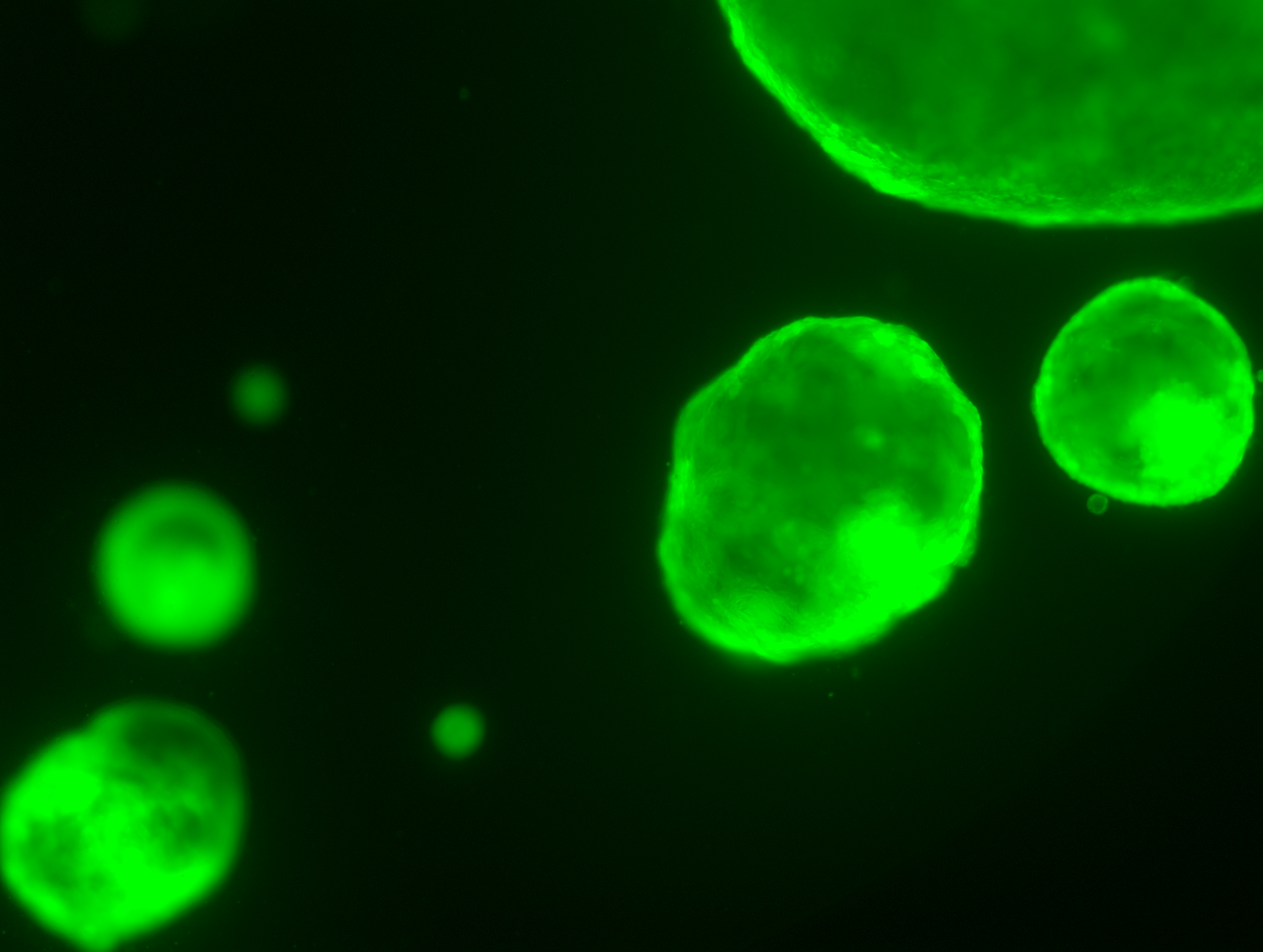

Supplement: Supplementary file 11 — Source data Fig. 6 [file 44318_2025_381_MOESM11_ESM.zip › Figure 6/6A/AK/AK_shCTRL.png]

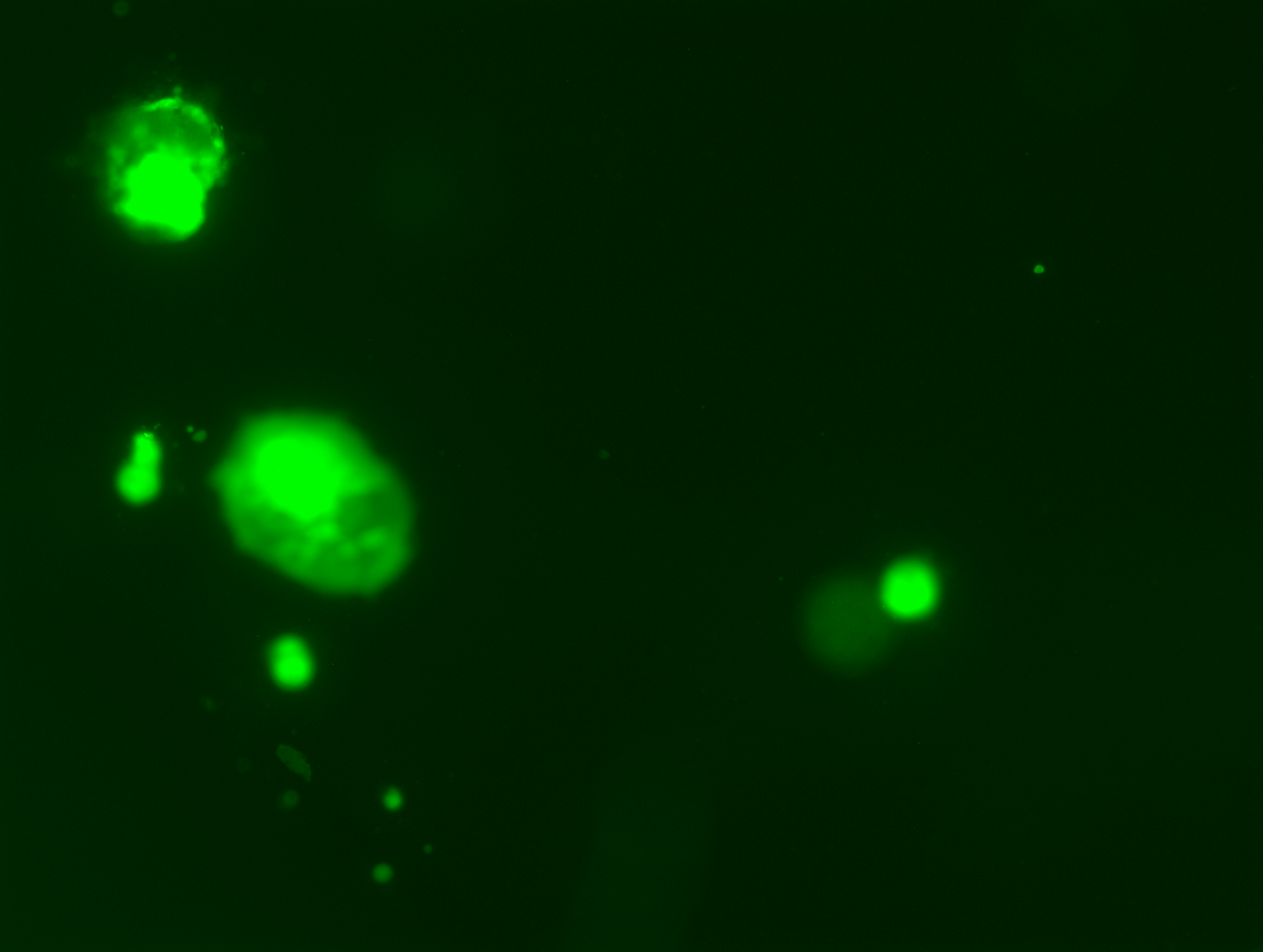

Supplement: Supplementary file 11 — Source data Fig. 6 [file 44318_2025_381_MOESM11_ESM.zip › Figure 6/6A/AK/AK_shEif2b1-1.png]

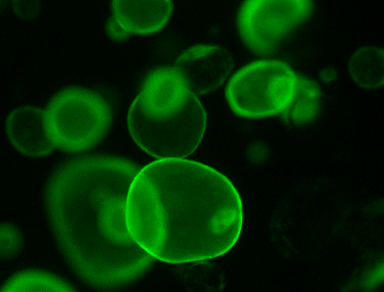

Supplement: Supplementary file 11 — Source data Fig. 6 [file 44318_2025_381_MOESM11_ESM.zip › Figure 6/6A/A/A_shCTRL.png]

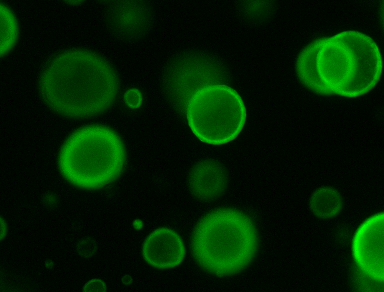

Supplement: Supplementary file 11 — Source data Fig. 6 [file 44318_2025_381_MOESM11_ESM.zip › Figure 6/6A/A/A_shEif2b1-1.png]

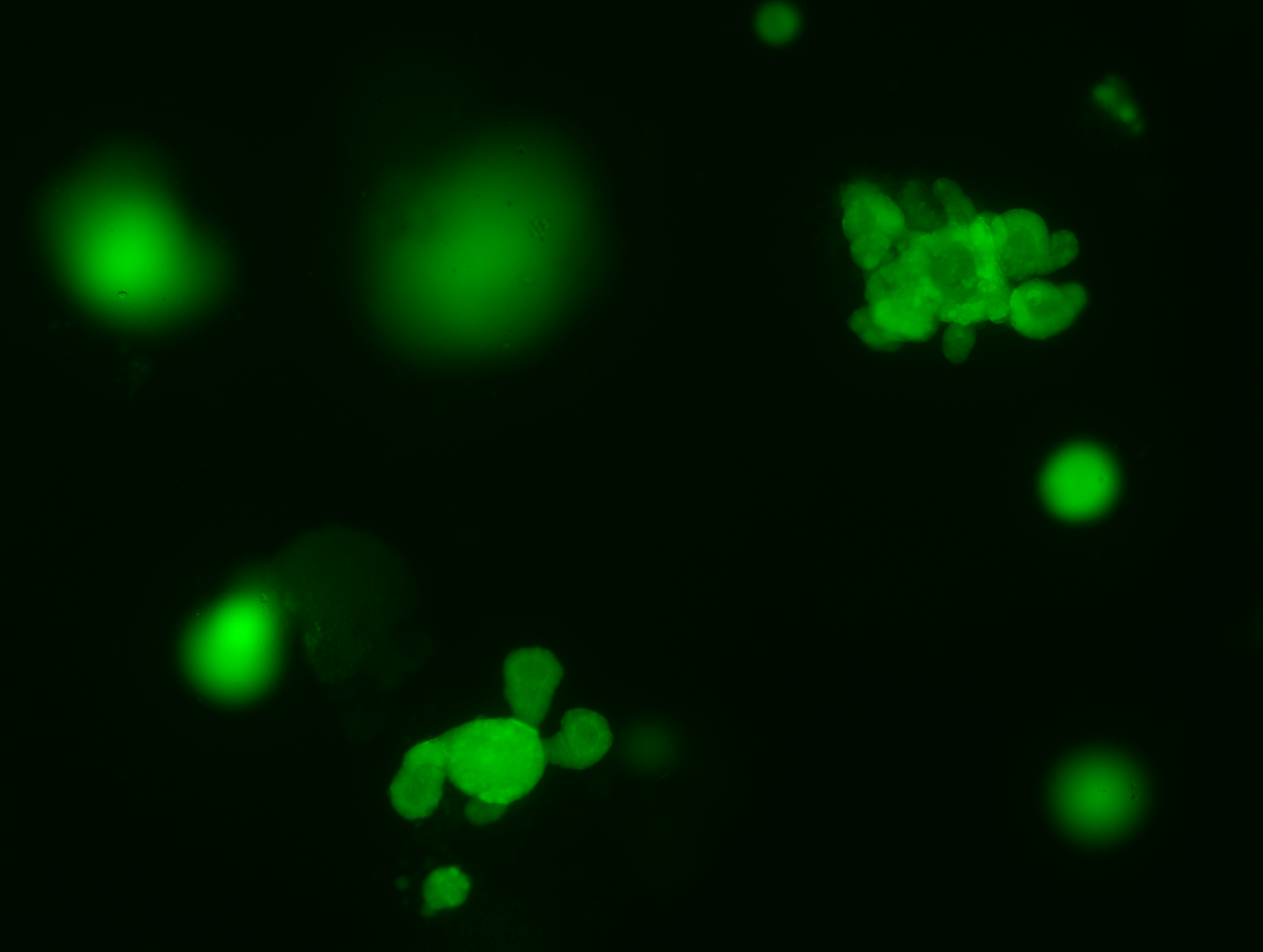

Supplement: Supplementary file 11 — Source data Fig. 6 [file 44318_2025_381_MOESM11_ESM.zip › Figure 6/6A/WT/WT_shEif2b1-1.png]

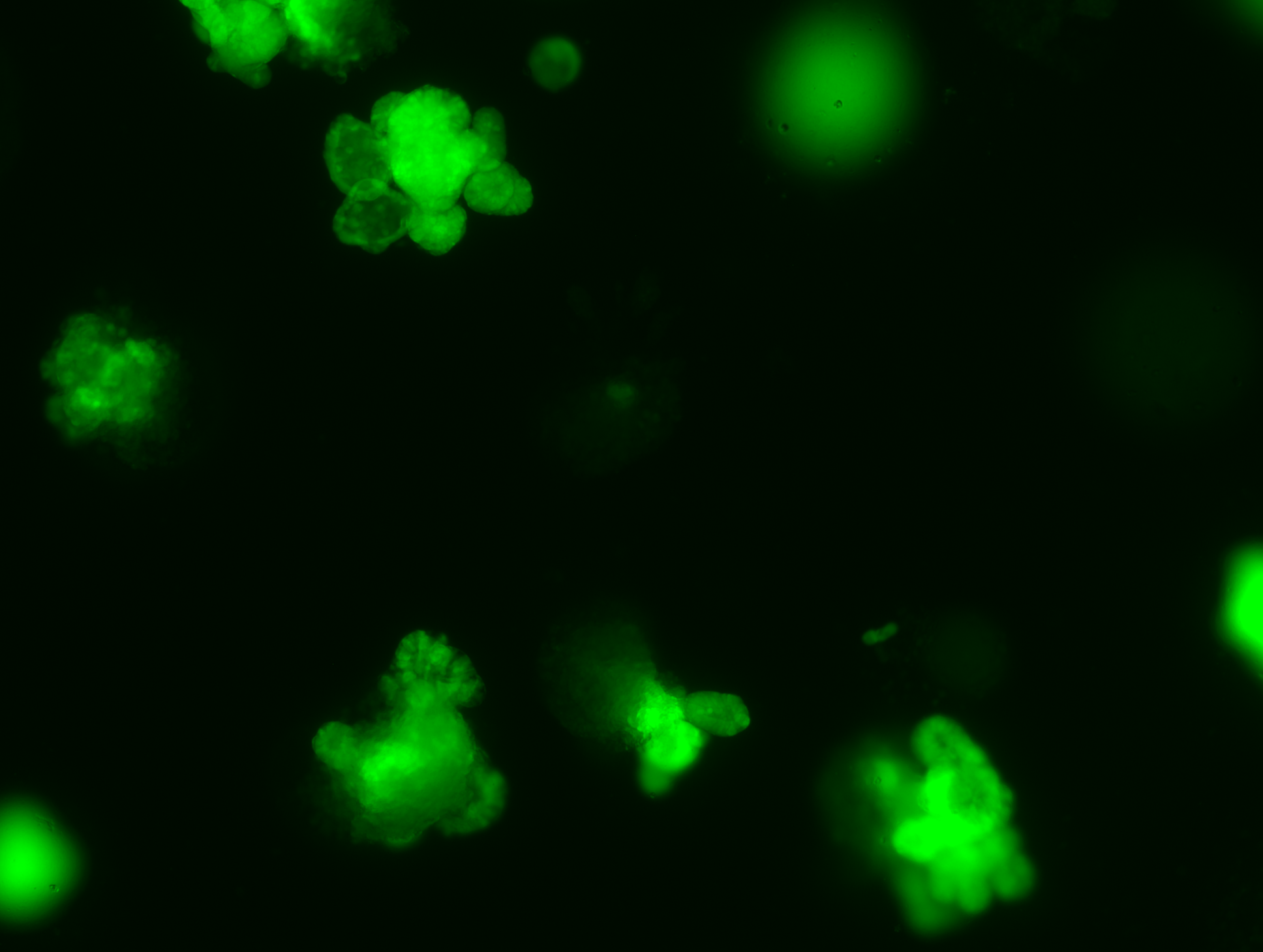

Supplement: Supplementary file 11 — Source data Fig. 6 [file 44318_2025_381_MOESM11_ESM.zip › Figure 6/6A/WT/WT_shCTRL.png]

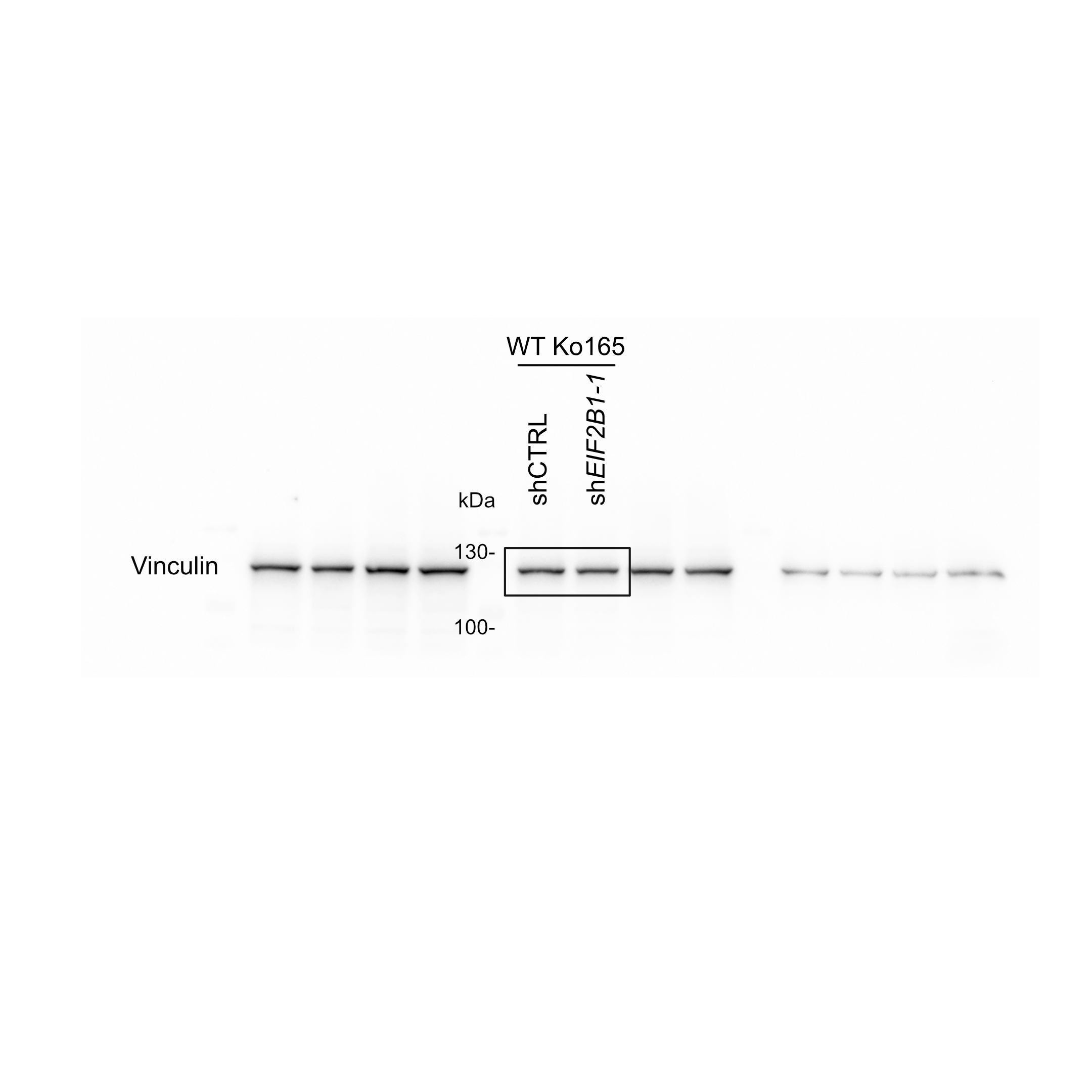

Supplement: Supplementary file 11 — Source data Fig. 6 [file 44318_2025_381_MOESM11_ESM.zip › Figure 6/6G/WT Ko165/western vinculin Ko165.tiff]

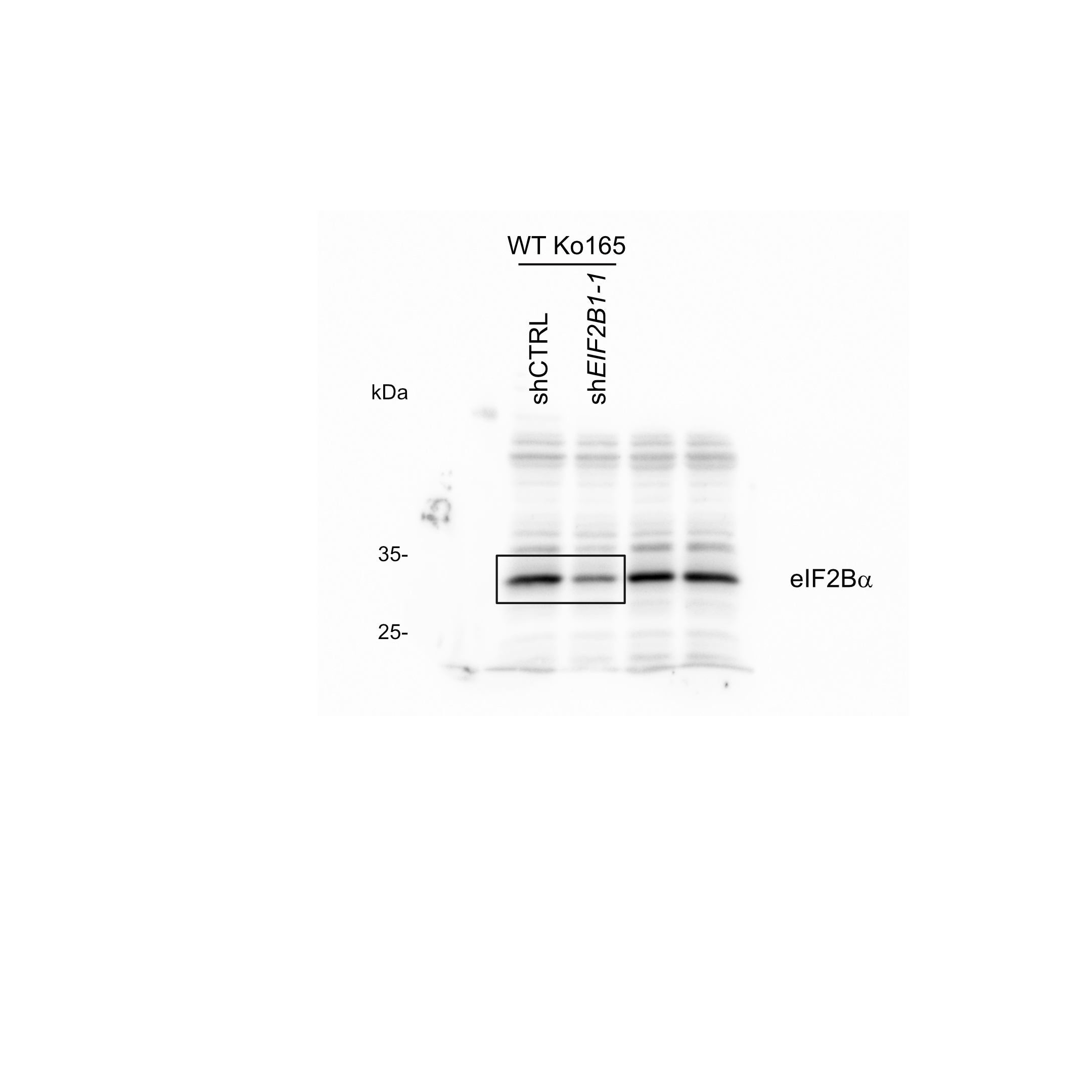

Supplement: Supplementary file 11 — Source data Fig. 6 [file 44318_2025_381_MOESM11_ESM.zip › Figure 6/6G/WT Ko165/western eIF2Ba Ko165.tiff]

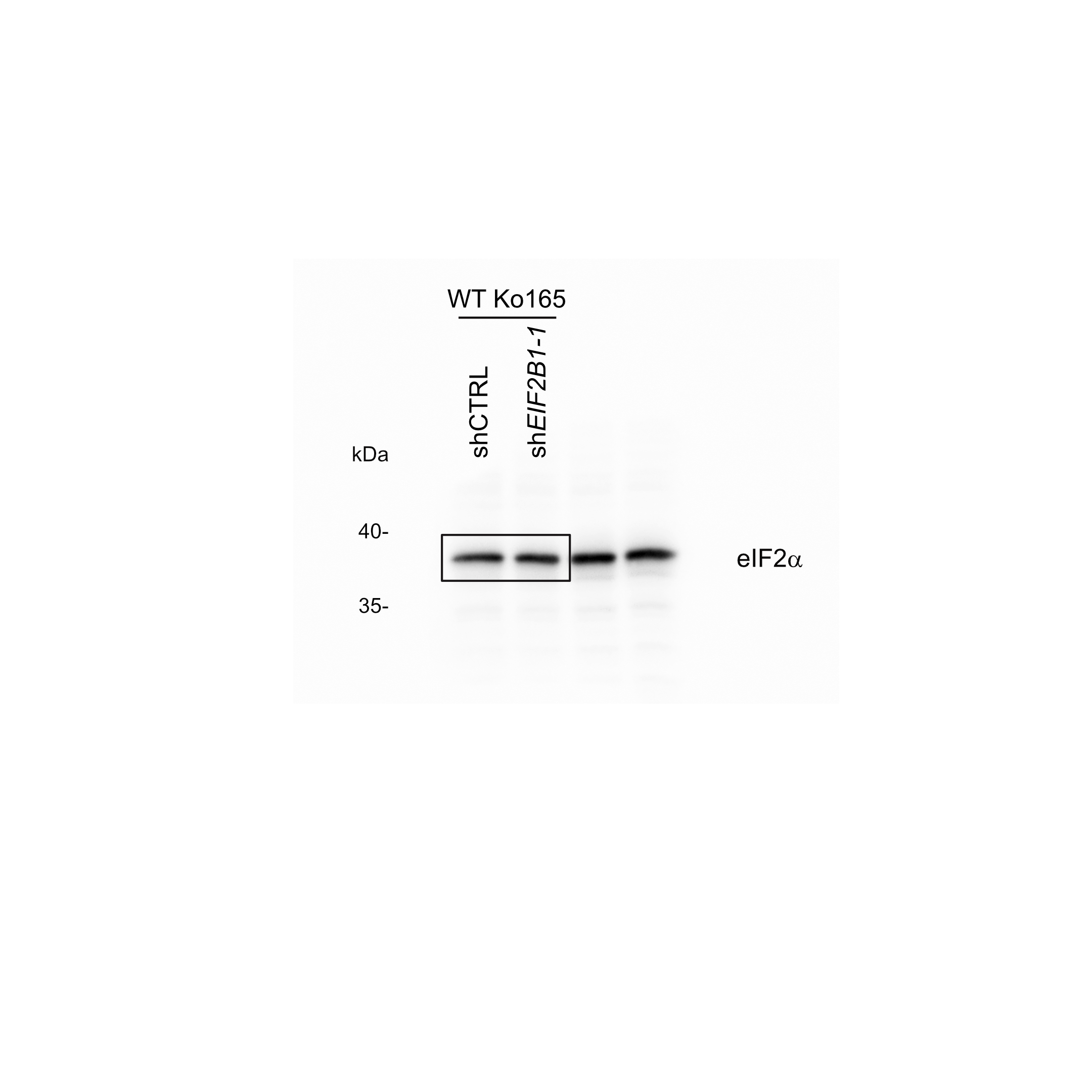

Supplement: Supplementary file 11 — Source data Fig. 6 [file 44318_2025_381_MOESM11_ESM.zip › Figure 6/6G/WT Ko165/western eIF2a Ko165.tiff]

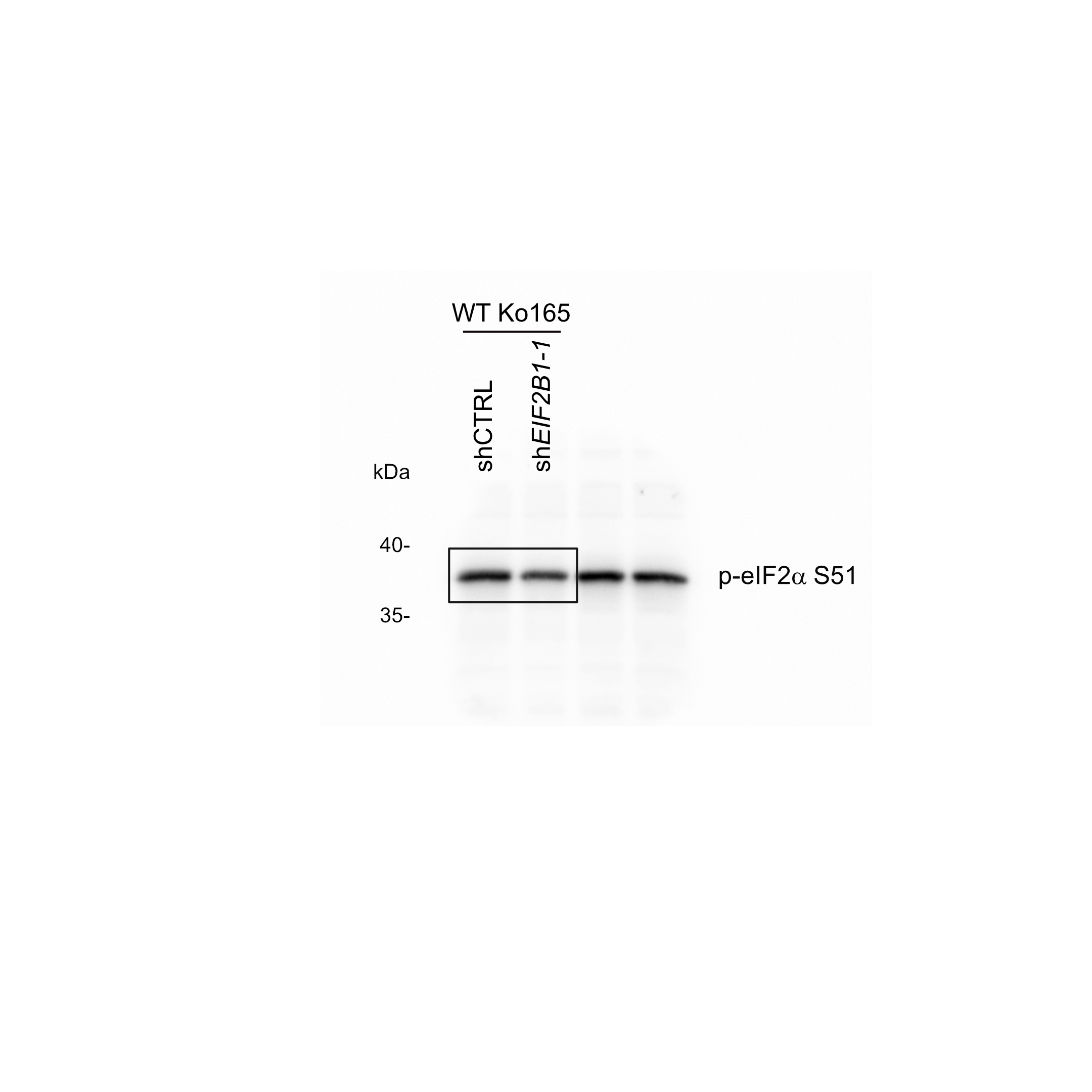

Supplement: Supplementary file 11 — Source data Fig. 6 [file 44318_2025_381_MOESM11_ESM.zip › Figure 6/6G/WT Ko165/western p-eIF2a S51 Ko165.tiff]

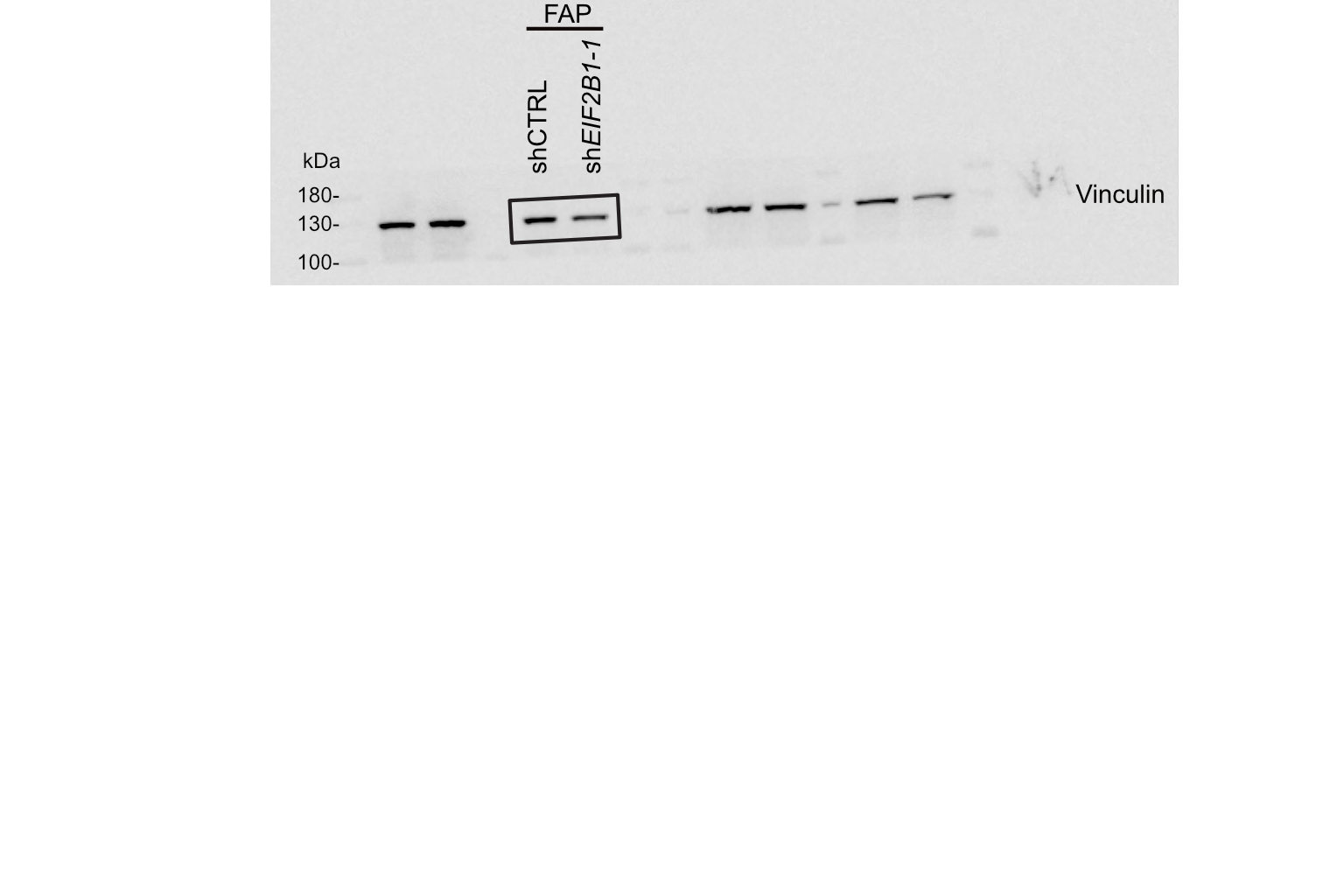

Supplement: Supplementary file 11 — Source data Fig. 6 [file 44318_2025_381_MOESM11_ESM.zip › Figure 6/6G/FAP/western vinculin FAP.tif]

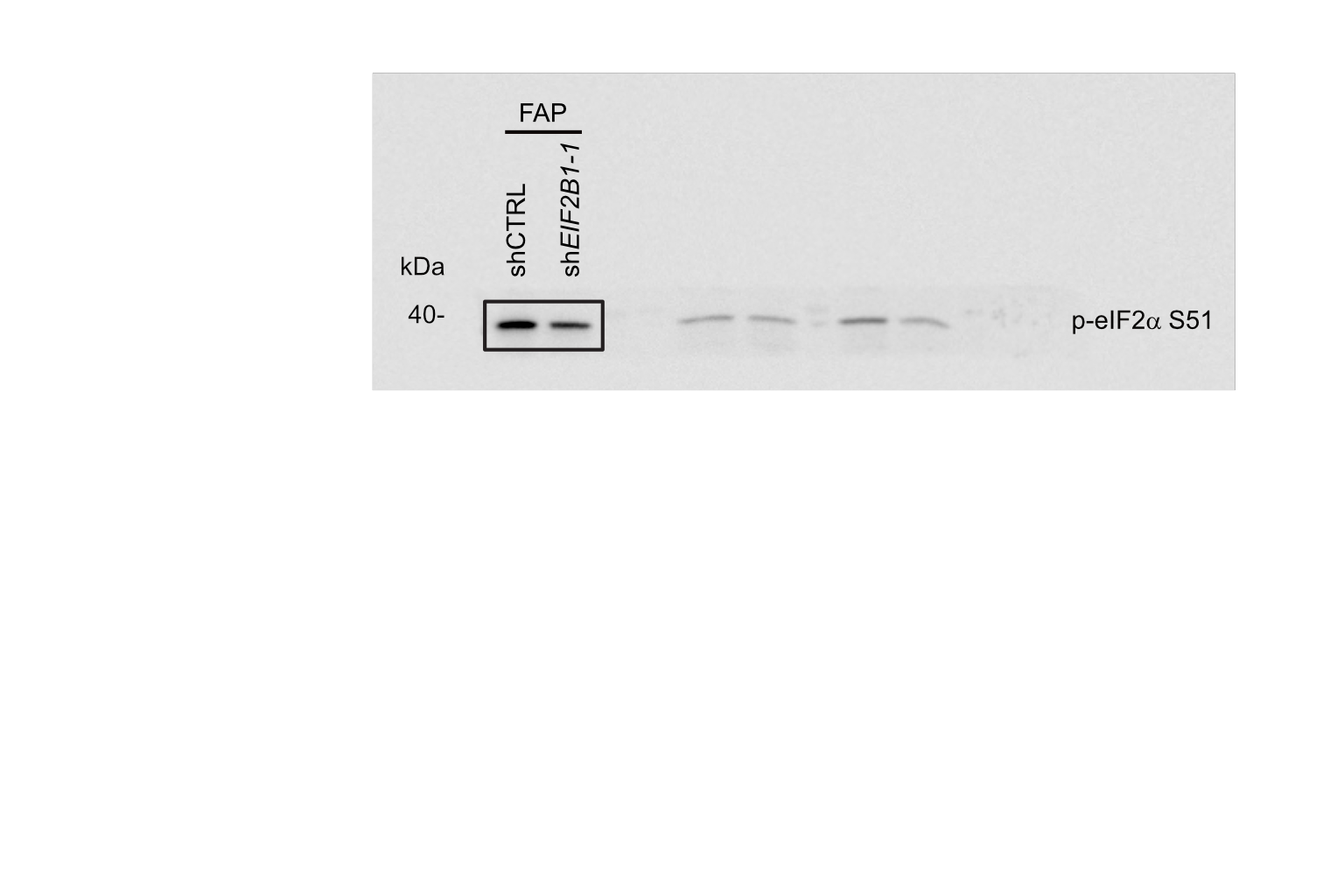

Supplement: Supplementary file 11 — Source data Fig. 6 [file 44318_2025_381_MOESM11_ESM.zip › Figure 6/6G/FAP/western p-eIF2a S51 FAP.tif]

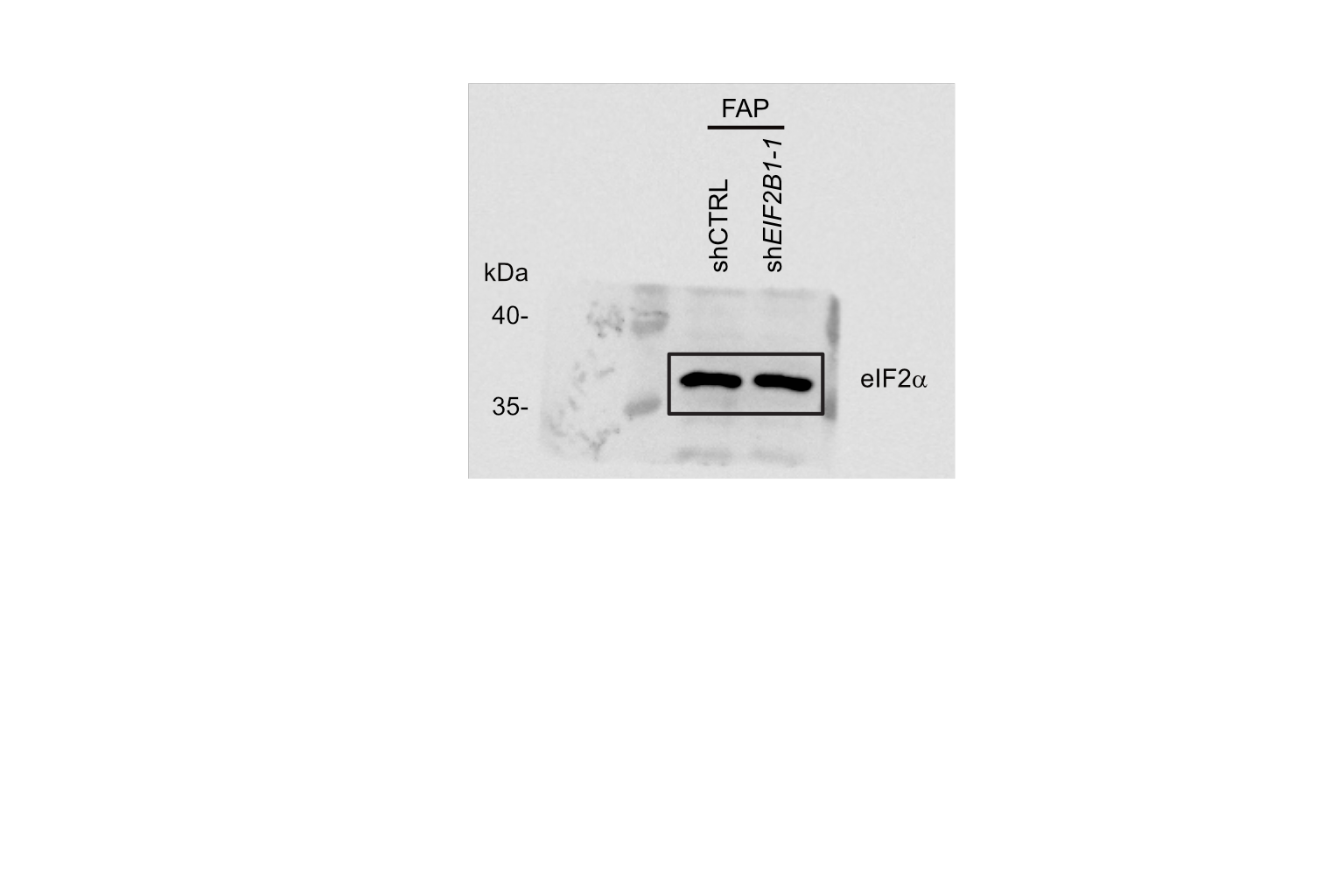

Supplement: Supplementary file 11 — Source data Fig. 6 [file 44318_2025_381_MOESM11_ESM.zip › Figure 6/6G/FAP/western eIF2a FAP.tif]

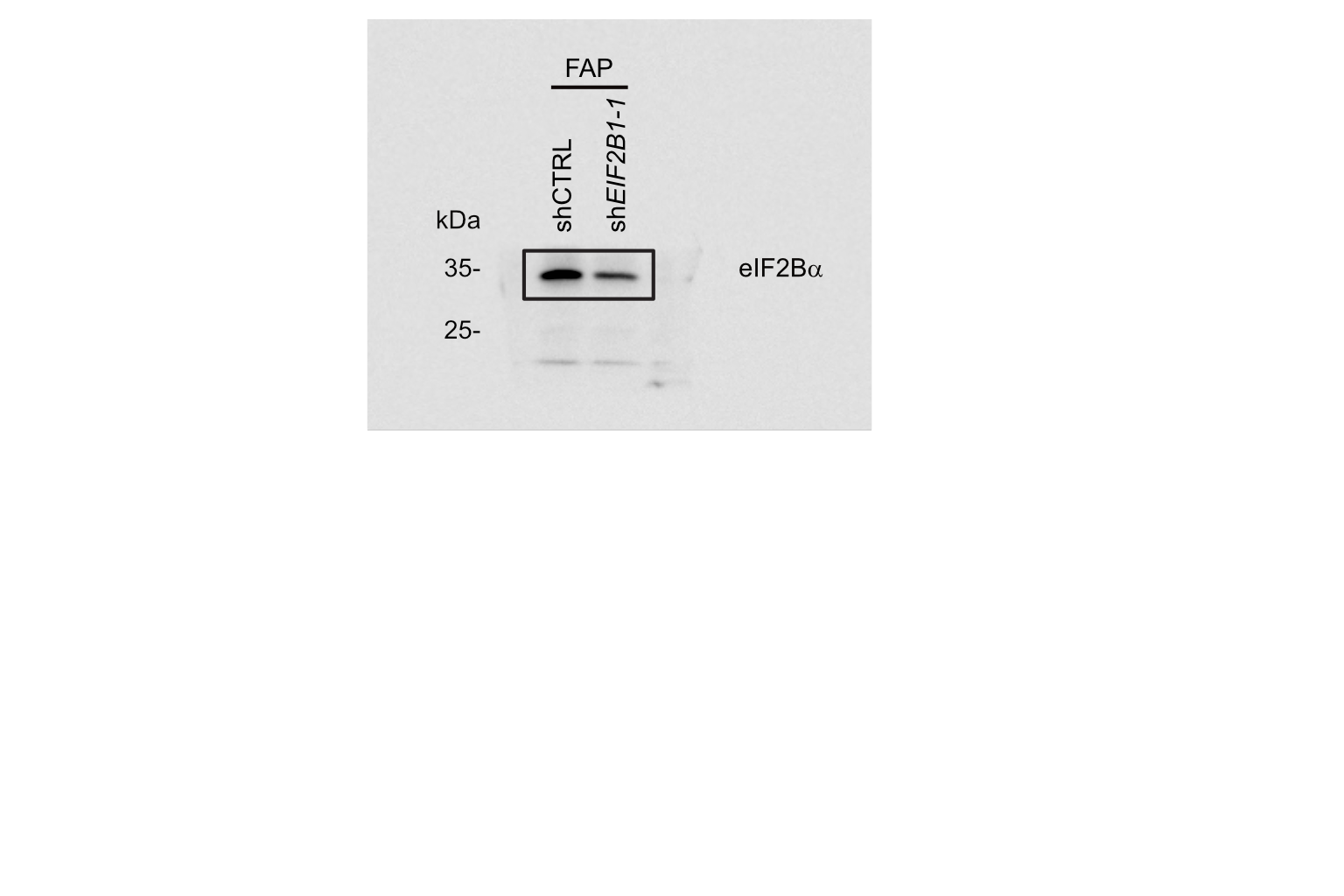

Supplement: Supplementary file 11 — Source data Fig. 6 [file 44318_2025_381_MOESM11_ESM.zip › Figure 6/6G/FAP/western eIF2Ba FAP.tif]

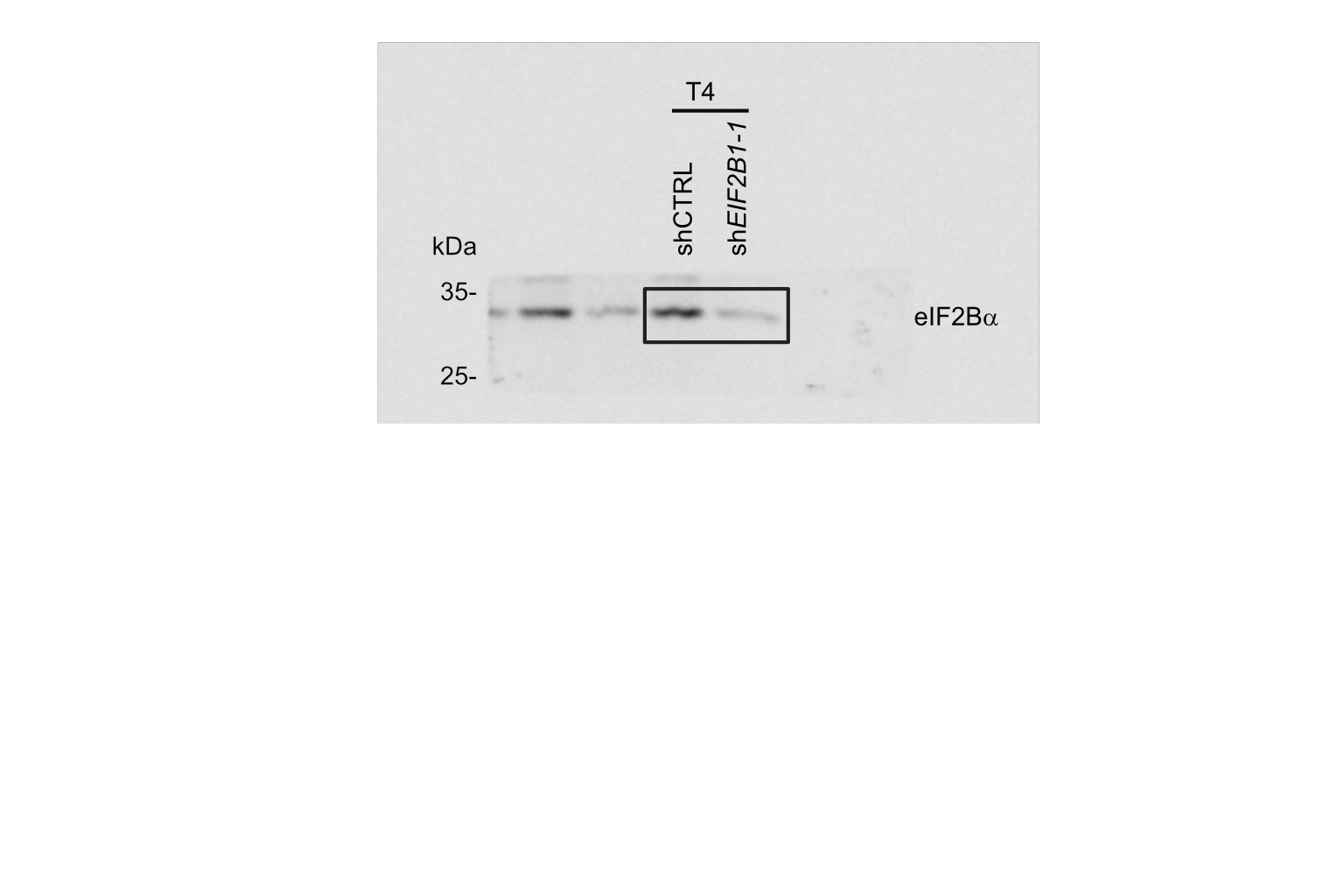

Supplement: Supplementary file 11 — Source data Fig. 6 [file 44318_2025_381_MOESM11_ESM.zip › Figure 6/6G/T4/western eIF2Ba T4.tif]

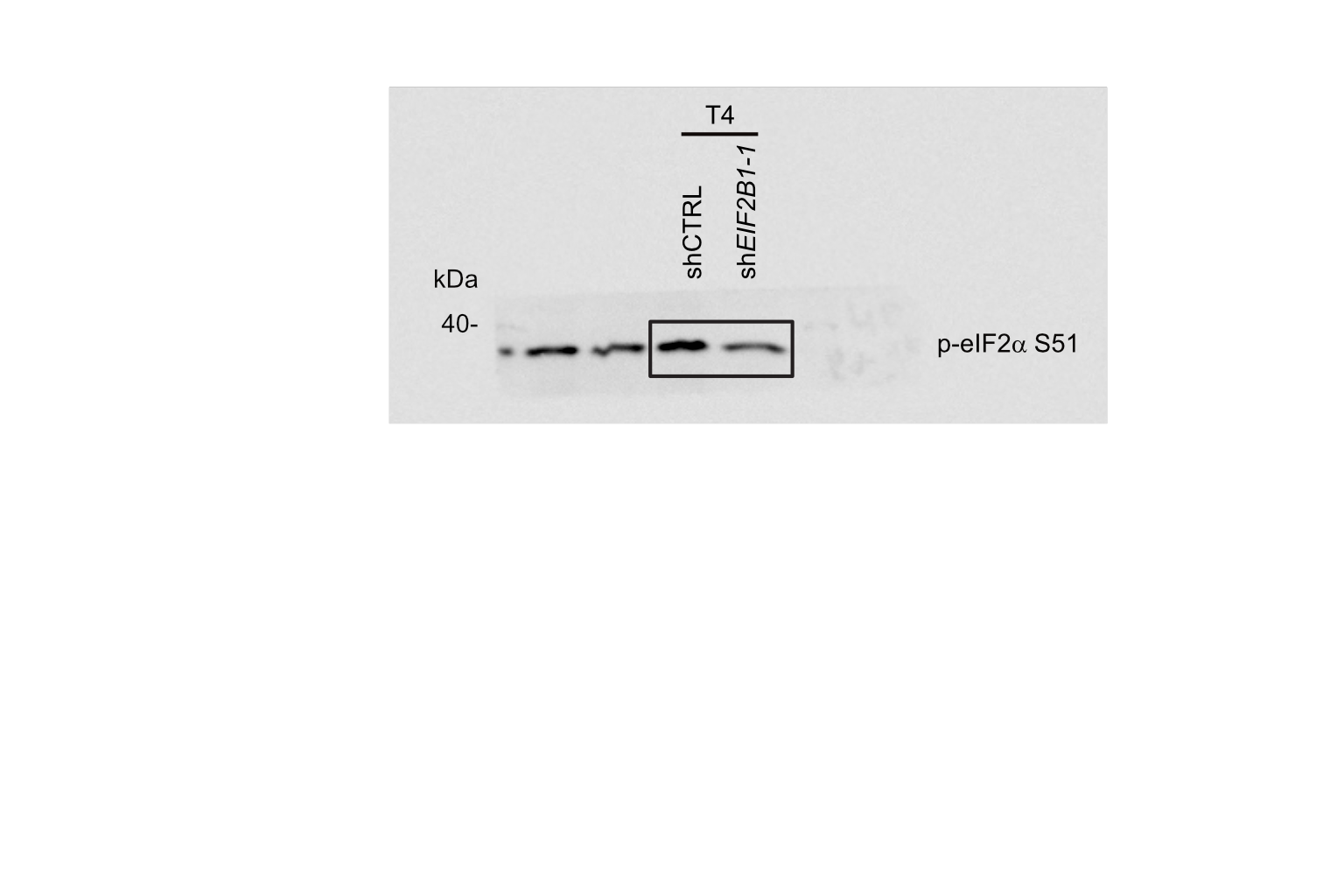

Supplement: Supplementary file 11 — Source data Fig. 6 [file 44318_2025_381_MOESM11_ESM.zip › Figure 6/6G/T4/western p-eIF2a S51 T4.tif]

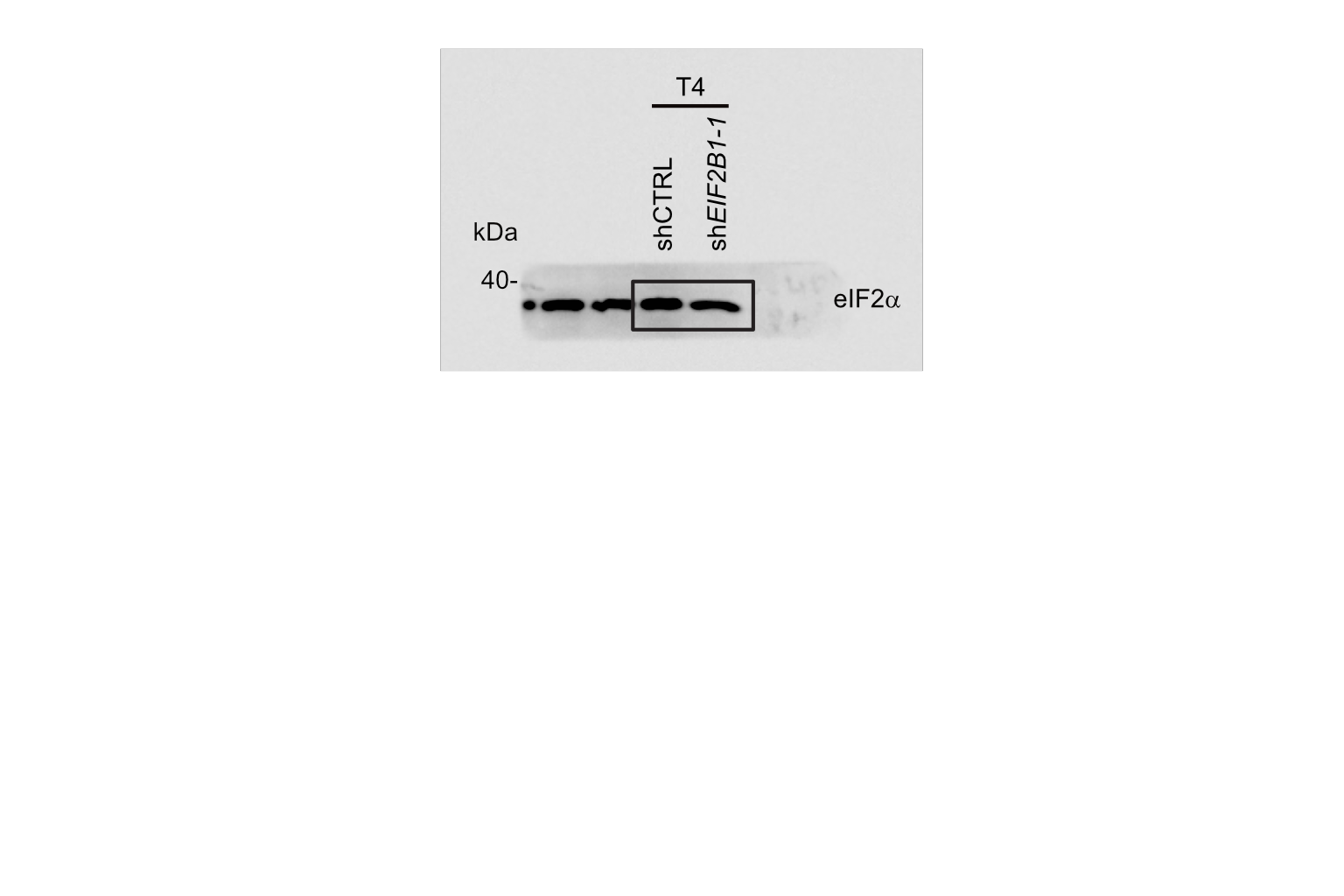

Supplement: Supplementary file 11 — Source data Fig. 6 [file 44318_2025_381_MOESM11_ESM.zip › Figure 6/6G/T4/western eIF2a T4.tif]

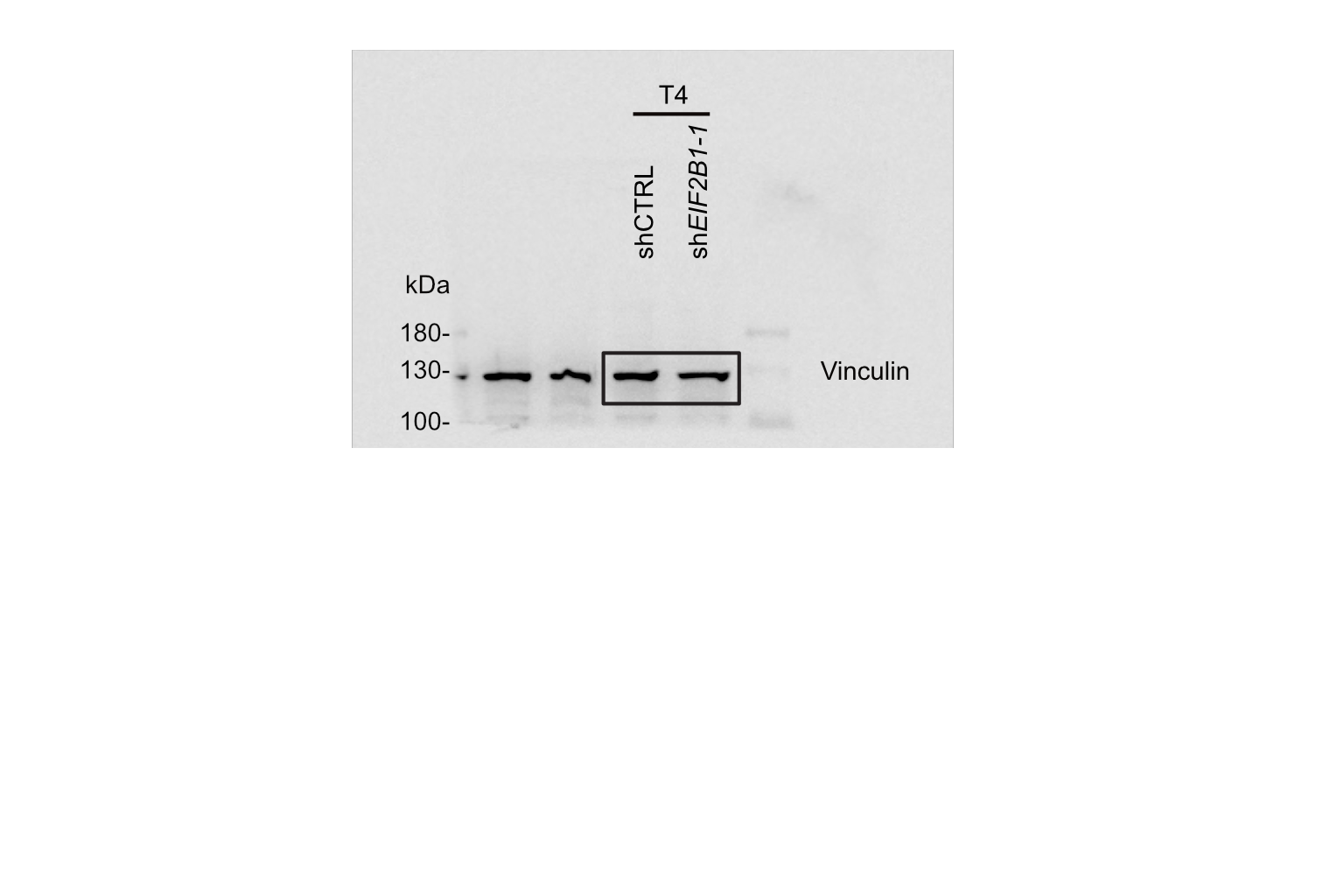

Supplement: Supplementary file 11 — Source data Fig. 6 [file 44318_2025_381_MOESM11_ESM.zip › Figure 6/6G/T4/western vinculin T4.tif]

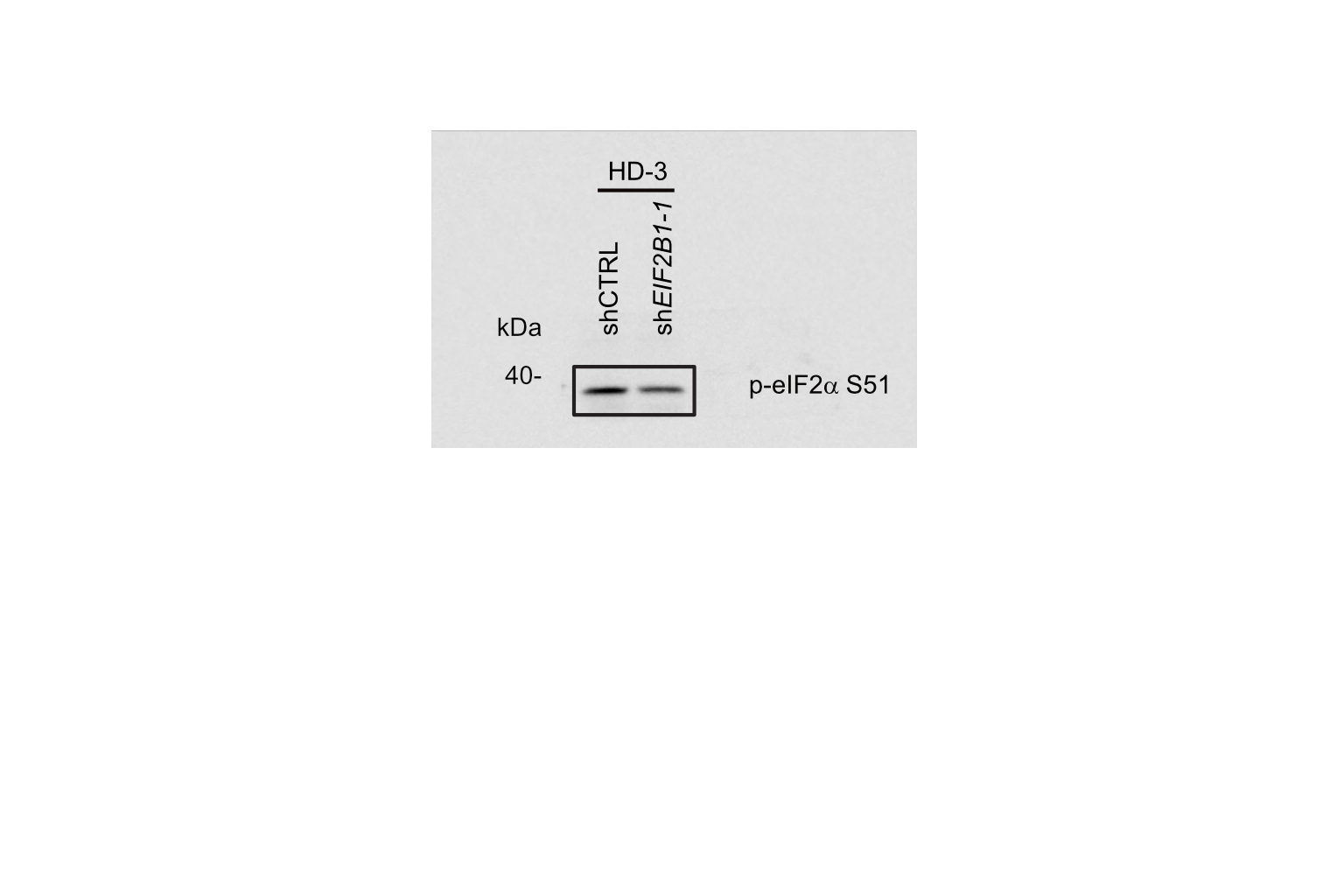

Supplement: Supplementary file 11 — Source data Fig. 6 [file 44318_2025_381_MOESM11_ESM.zip › Figure 6/6G/HD-3/western p-eIF2a S51 HD-3.tif]

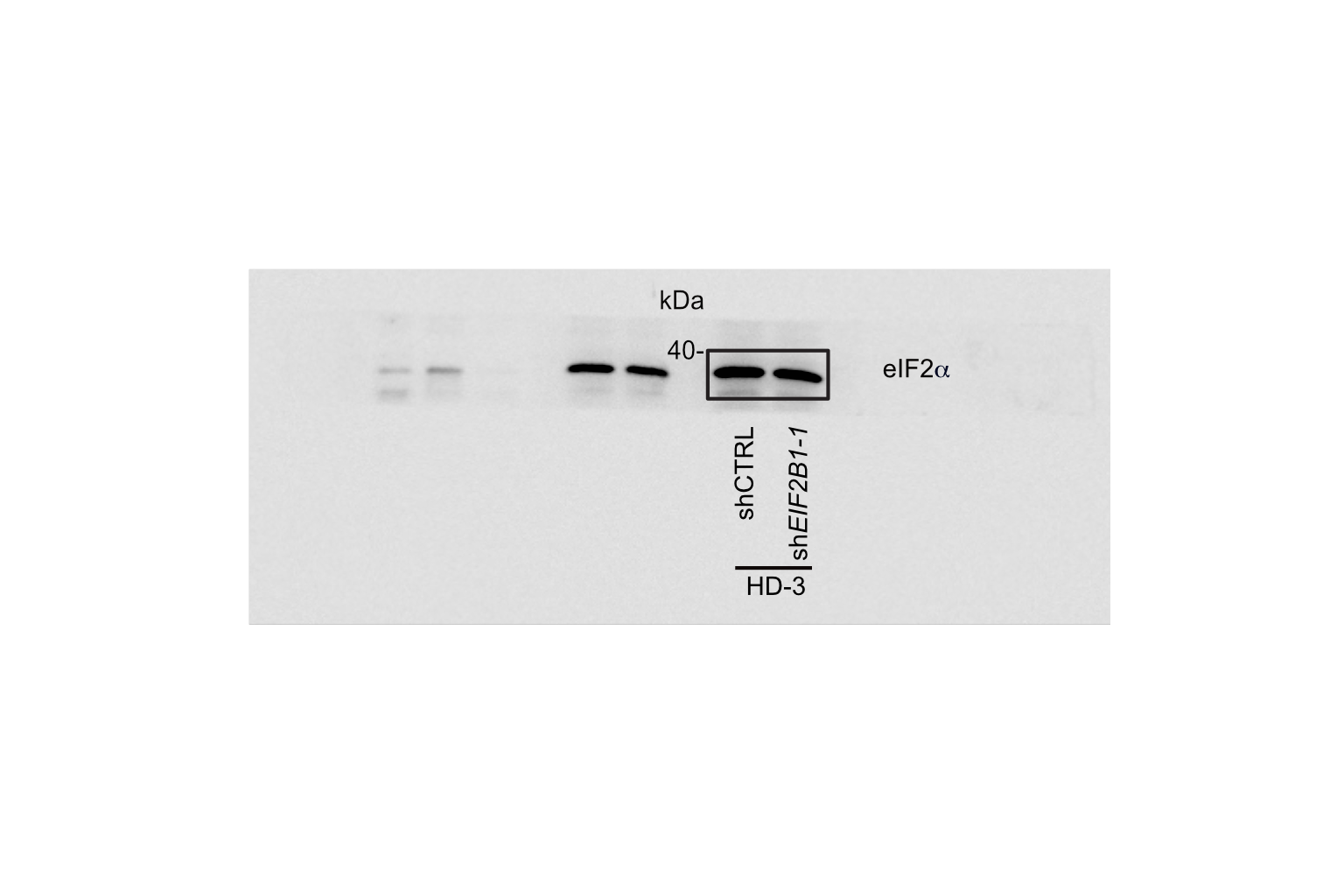

Supplement: Supplementary file 11 — Source data Fig. 6 [file 44318_2025_381_MOESM11_ESM.zip › Figure 6/6G/HD-3/western eIF2a HD-3.tif]

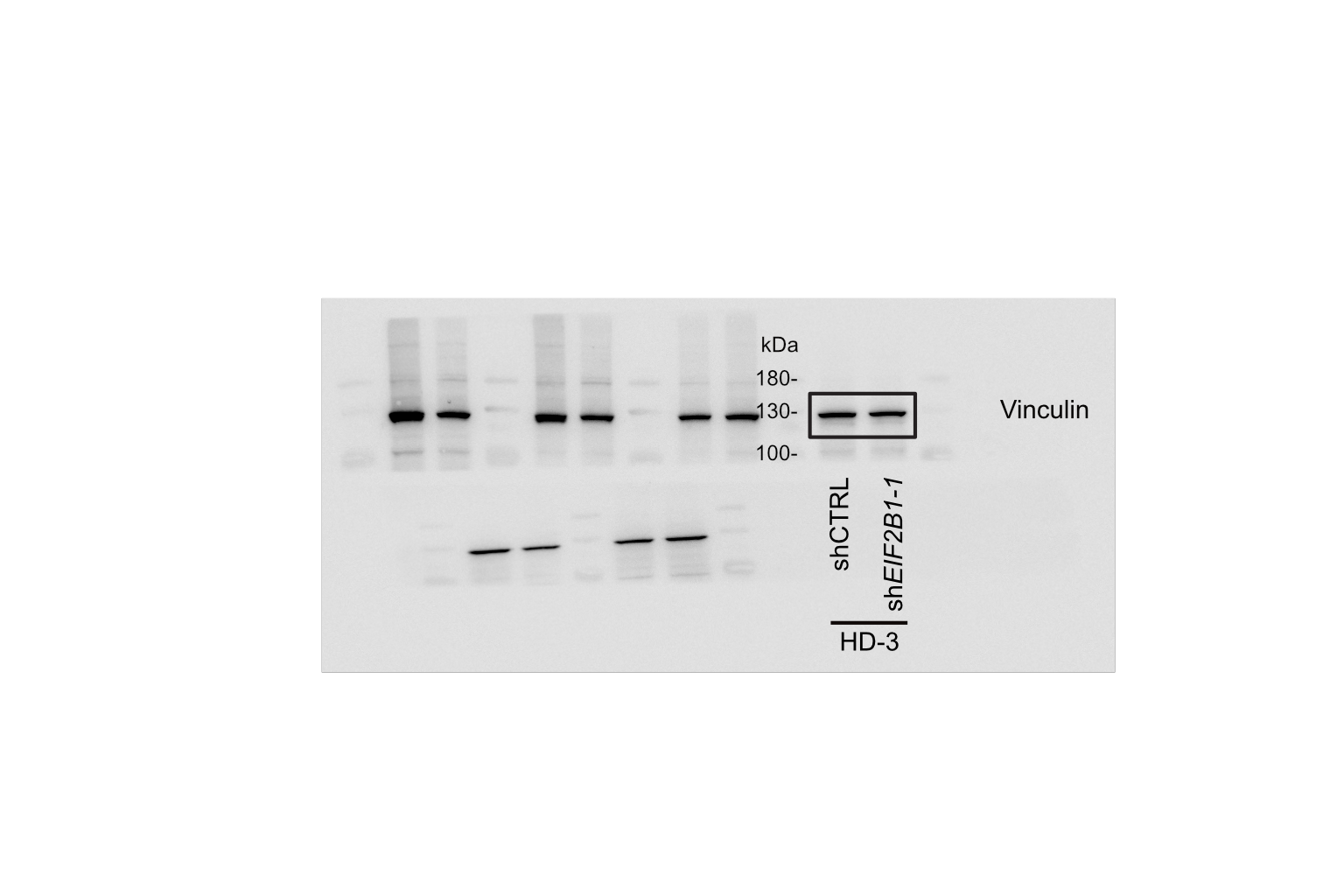

Supplement: Supplementary file 11 — Source data Fig. 6 [file 44318_2025_381_MOESM11_ESM.zip › Figure 6/6G/HD-3/western vinculin HD-3.tif]

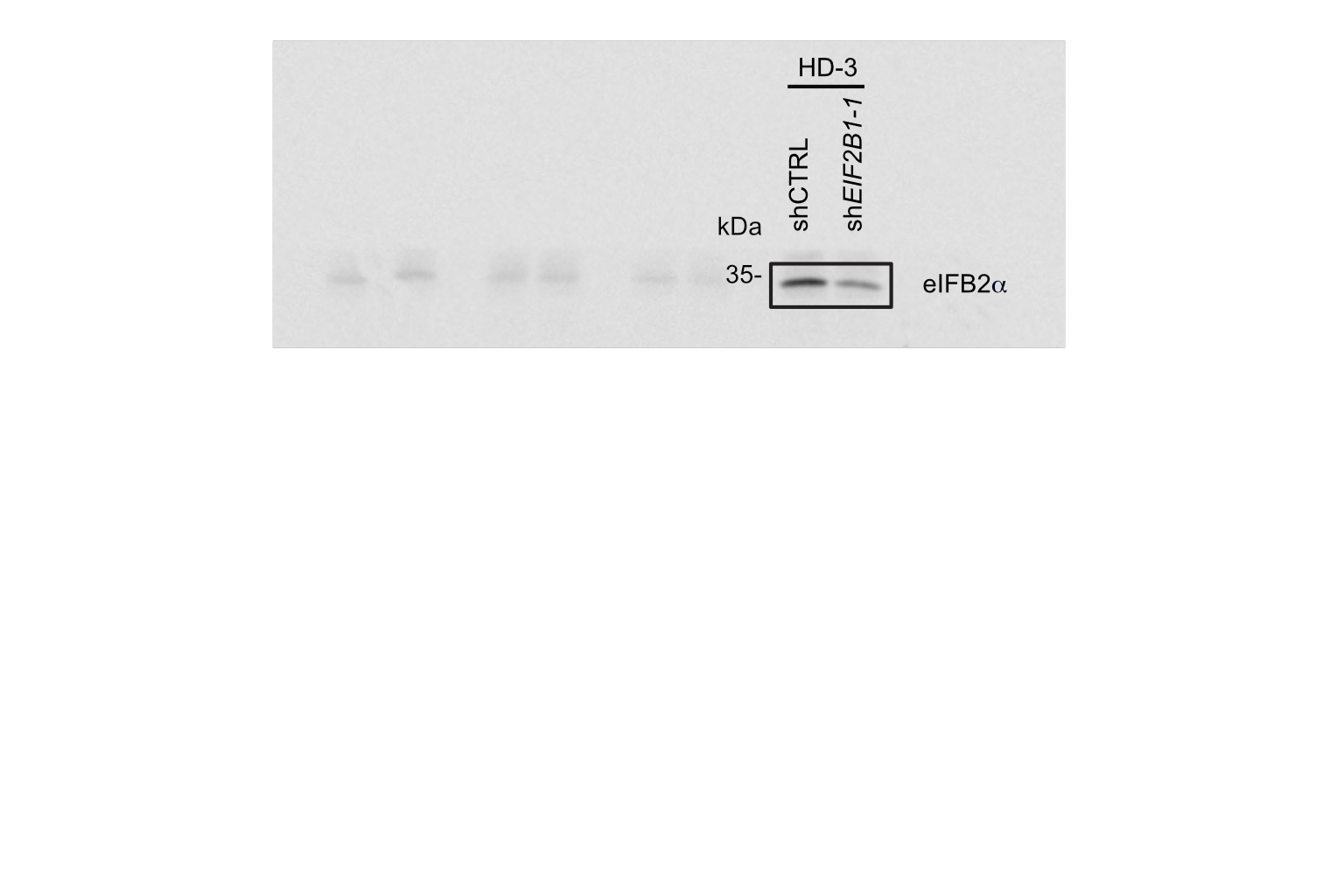

Supplement: Supplementary file 11 — Source data Fig. 6 [file 44318_2025_381_MOESM11_ESM.zip › Figure 6/6G/HD-3/western eIF2Ba HD-3.tif]

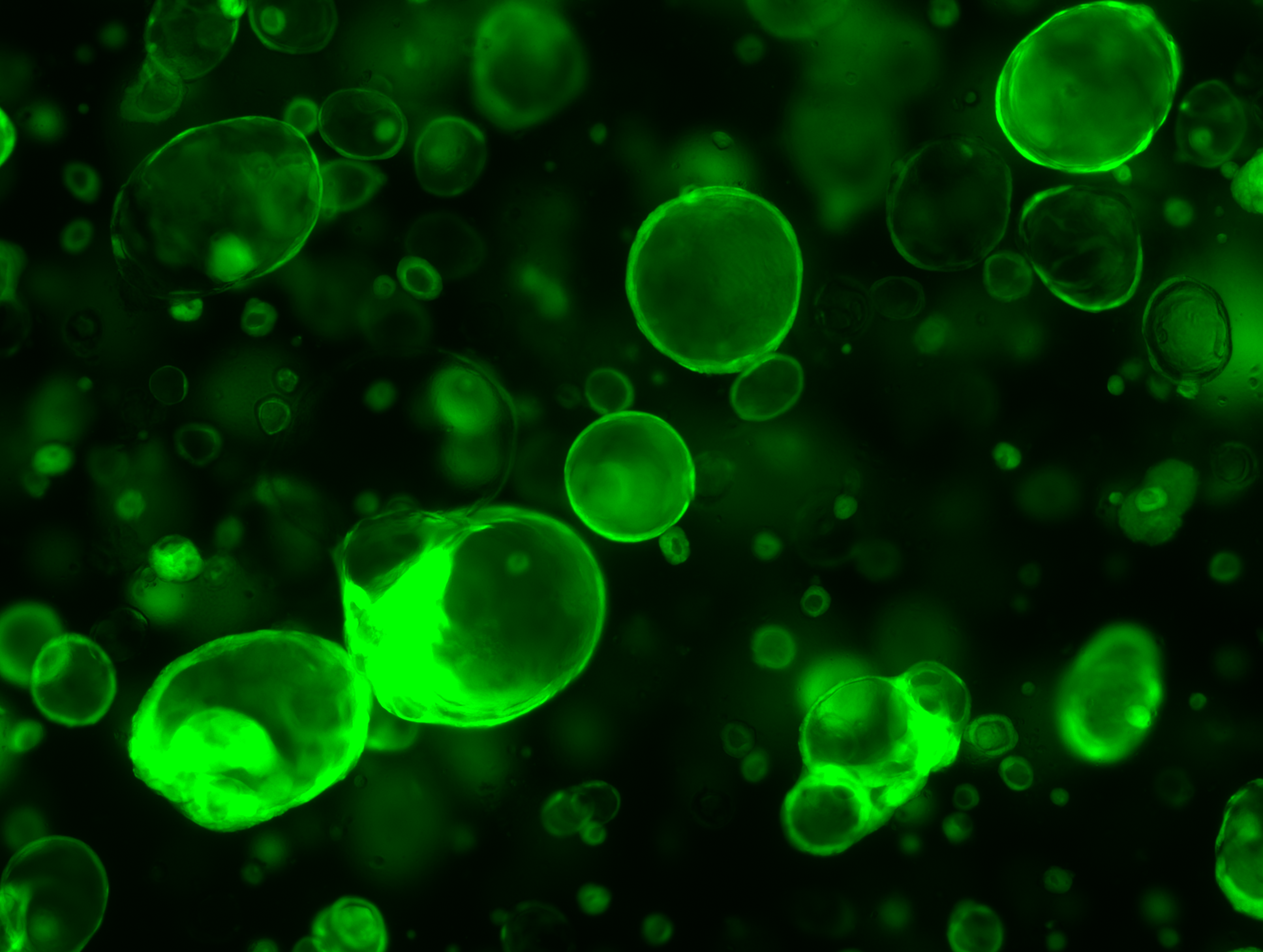

Supplement: Supplementary file 11 — Source data Fig. 6 [file 44318_2025_381_MOESM11_ESM.zip › Figure 6/6E/WT Ko165/Ko165_shEIF2B1-1.tif]
