# Supplementary figures and images for: Maintenance of p-eIF2α levels by the eIF2B complex is vital for colorectal cancer (part 2 of 2)
Source: EMBO J. 2025 Feb 27;44(7):2075–105. doi: 10.1038/s44318-025-00381-9 (PMC11962125; doi:10.1038/s44318-025-00381-9)

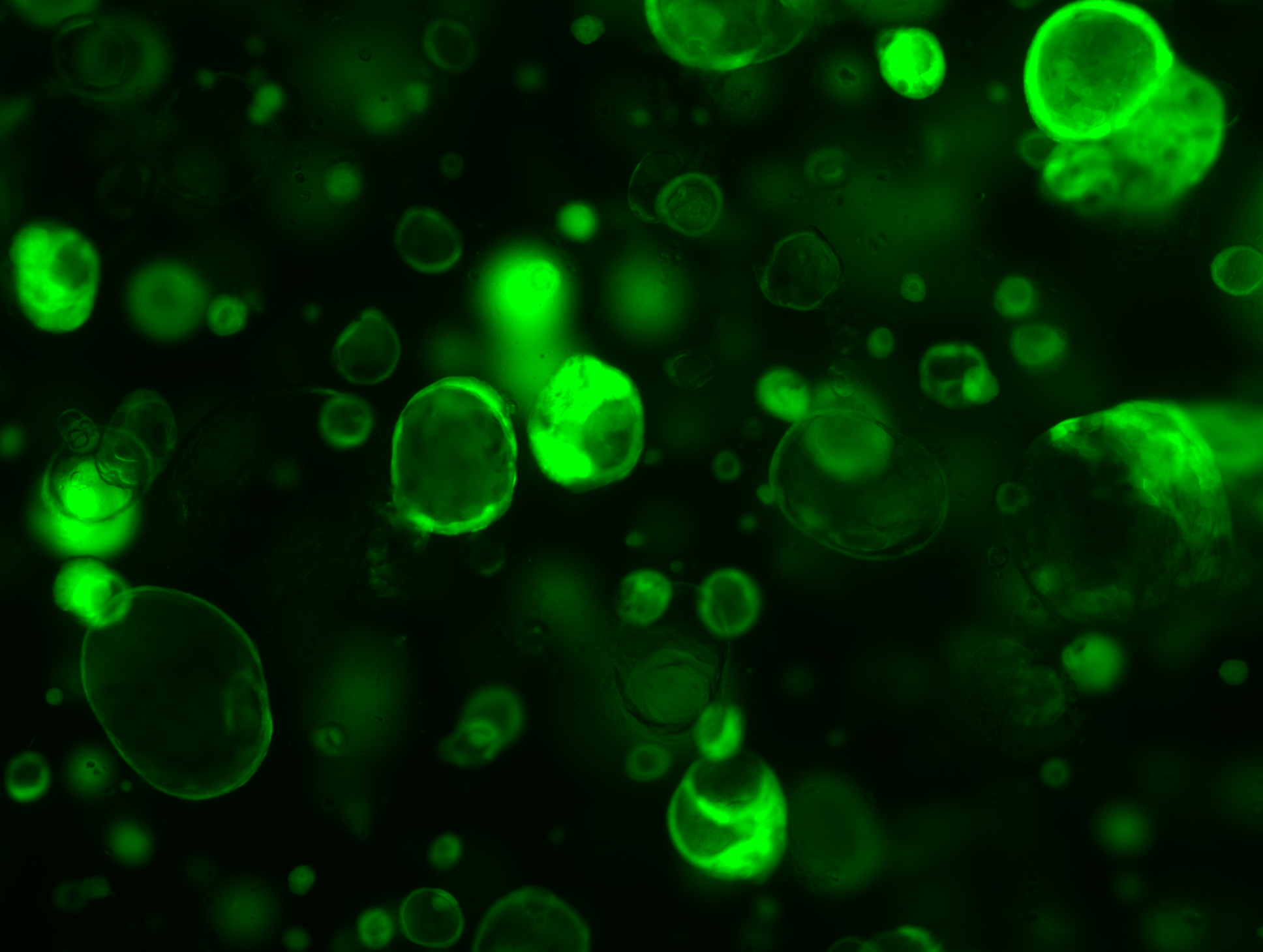

Supplement: Supplementary file 11 — Source data Fig. 6 [file 44318_2025_381_MOESM11_ESM.zip › Figure 6/6E/WT Ko165/Ko165_shCTRL.tif]

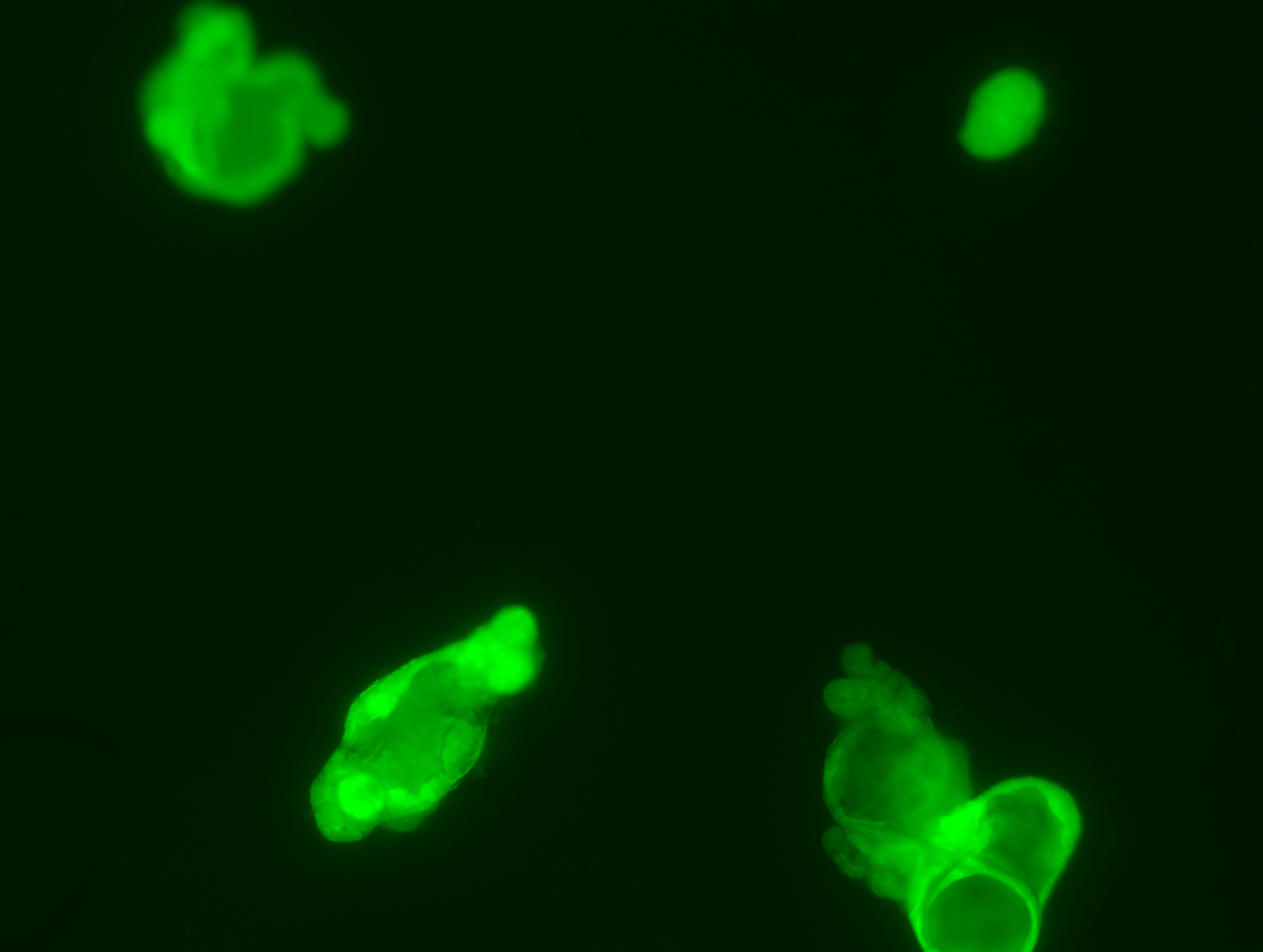

Supplement: Supplementary file 11 — Source data Fig. 6 [file 44318_2025_381_MOESM11_ESM.zip › Figure 6/6E/FAP/FAP_shEIF2B1-1.png]

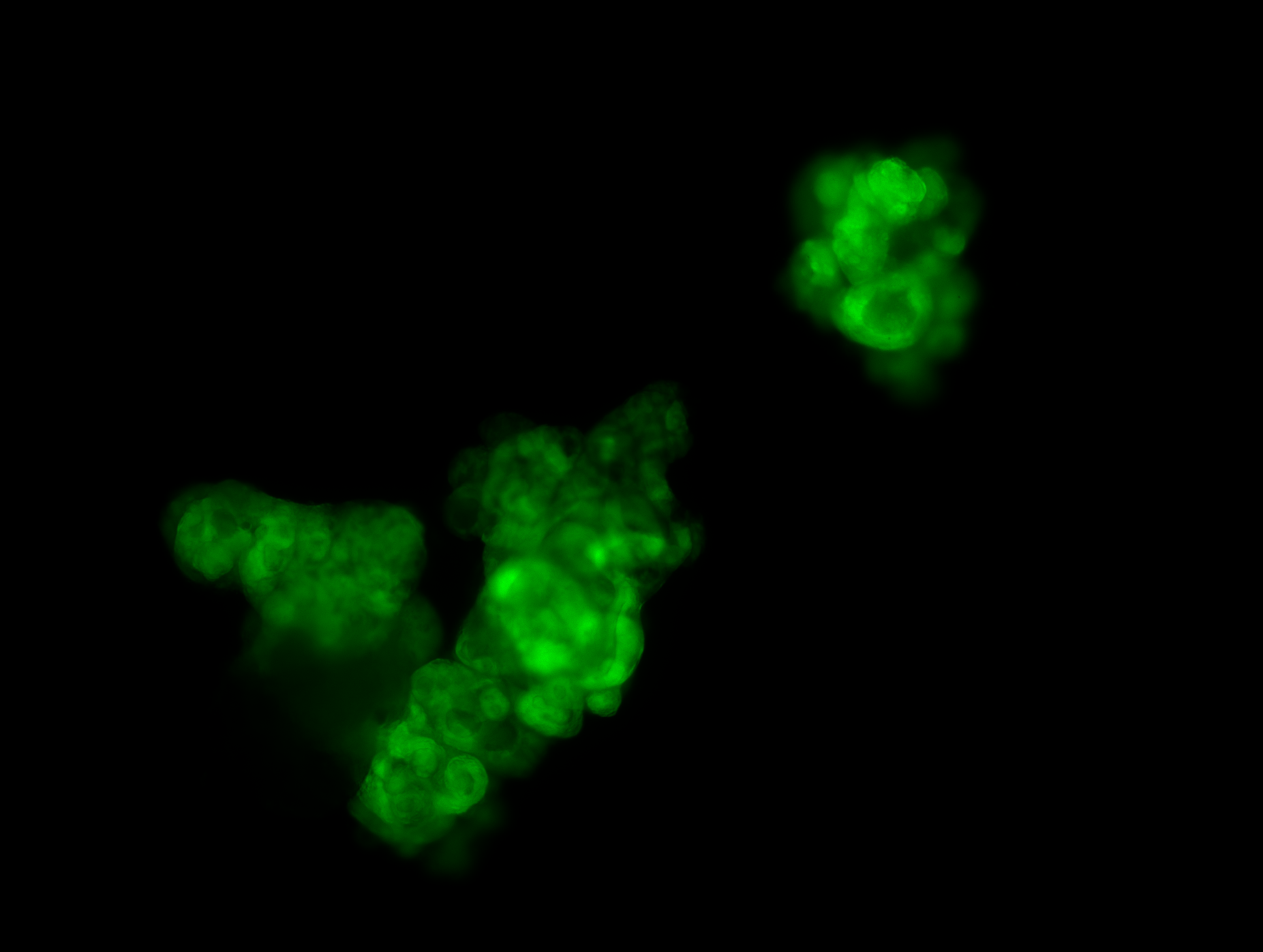

Supplement: Supplementary file 11 — Source data Fig. 6 [file 44318_2025_381_MOESM11_ESM.zip › Figure 6/6E/FAP/FAP_shCTRL.png]

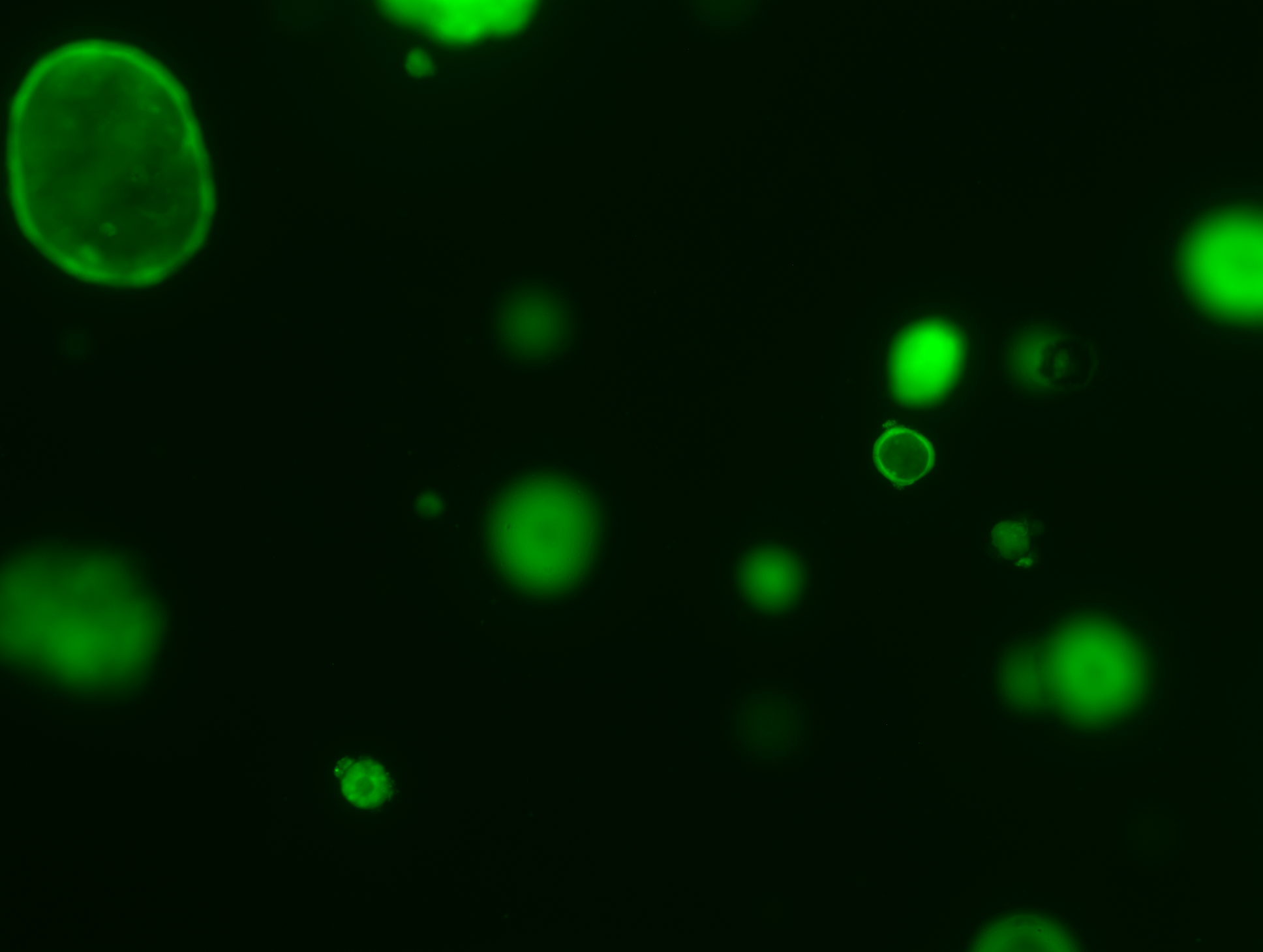

Supplement: Supplementary file 11 — Source data Fig. 6 [file 44318_2025_381_MOESM11_ESM.zip › Figure 6/6E/T4/T4_shEIF2B1-1.png]

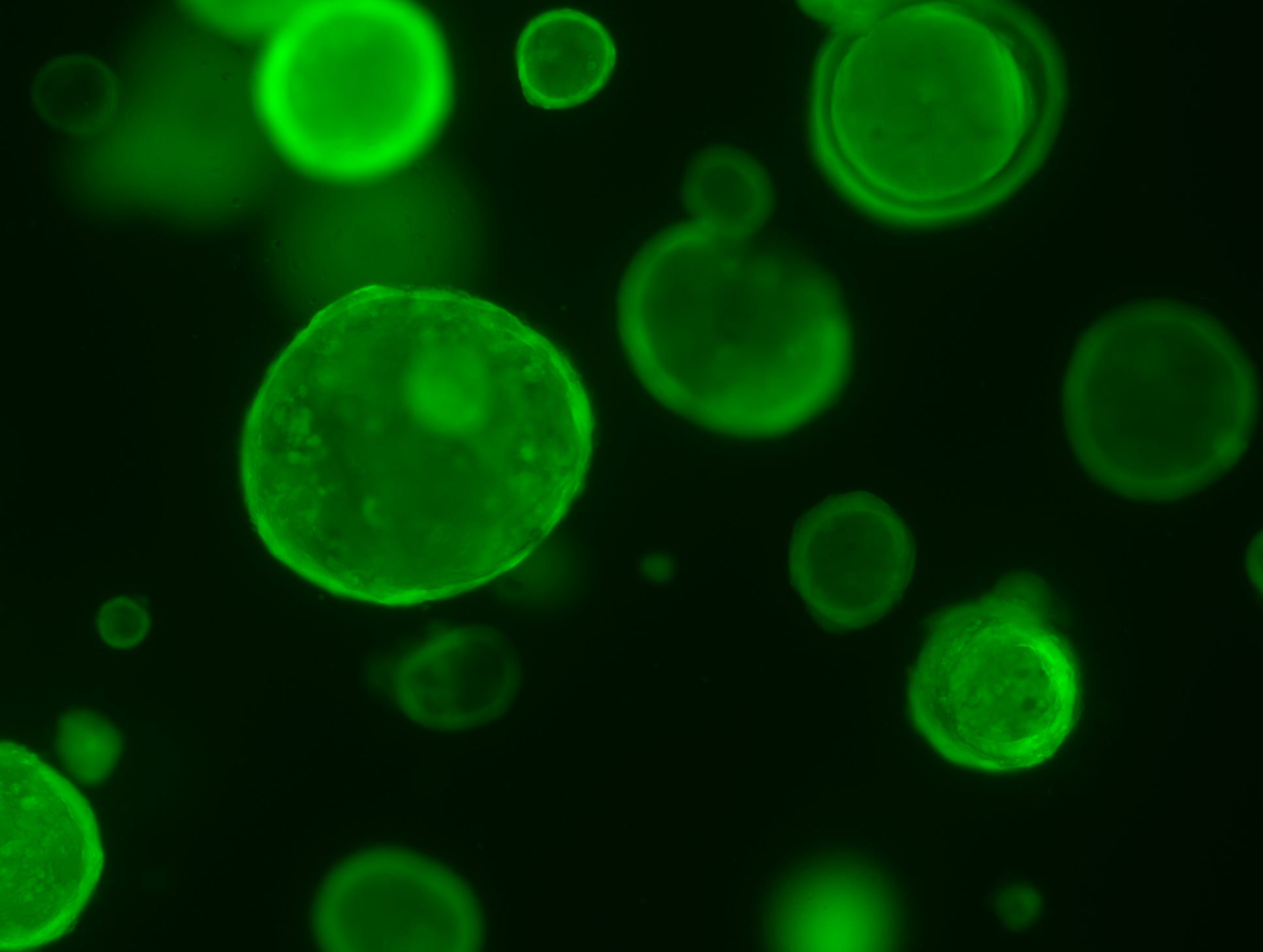

Supplement: Supplementary file 11 — Source data Fig. 6 [file 44318_2025_381_MOESM11_ESM.zip › Figure 6/6E/T4/T4_shCTRL.png]

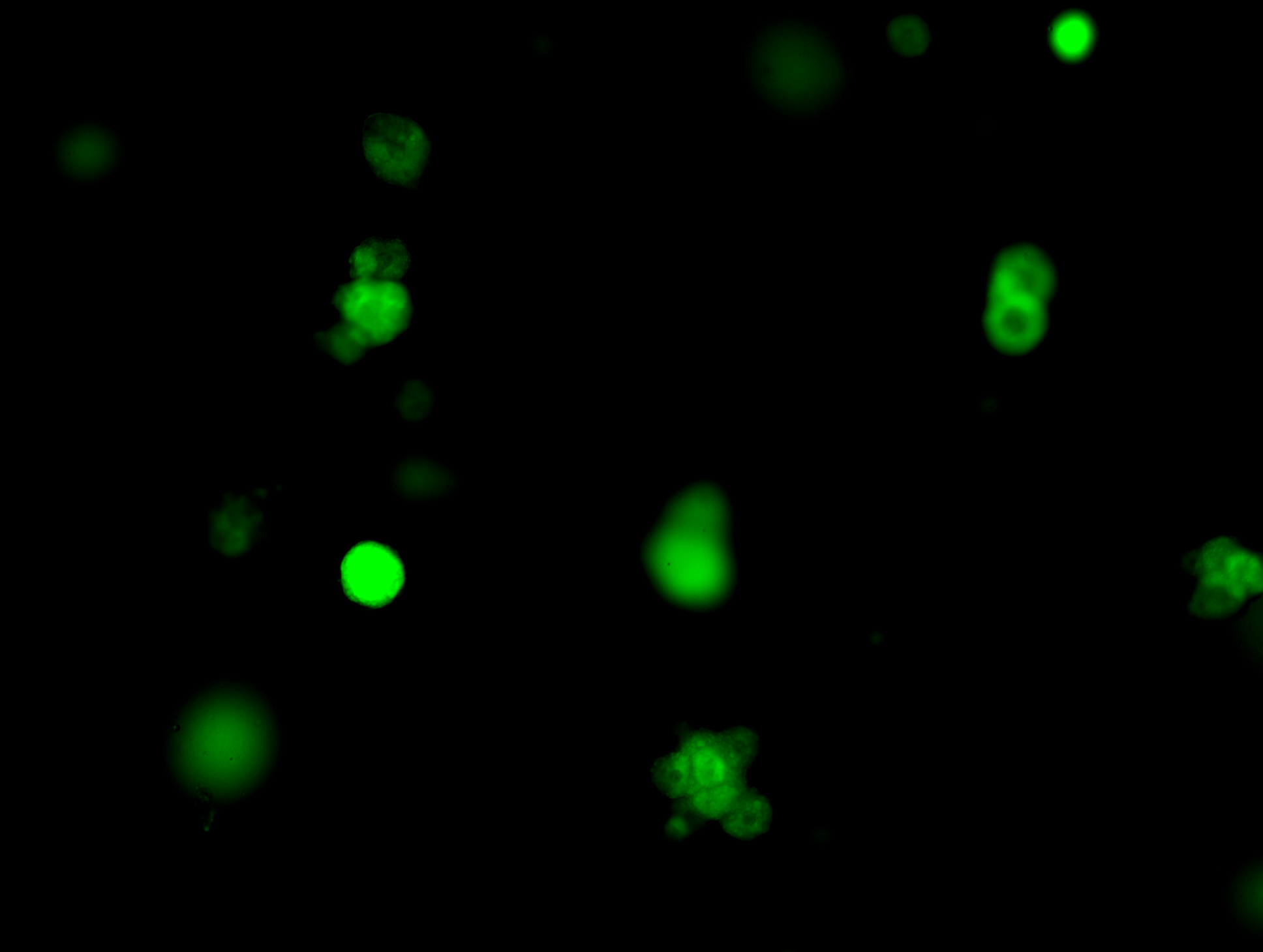

Supplement: Supplementary file 11 — Source data Fig. 6 [file 44318_2025_381_MOESM11_ESM.zip › Figure 6/6E/HD-3/HD3_shEIF2B1-1.png]

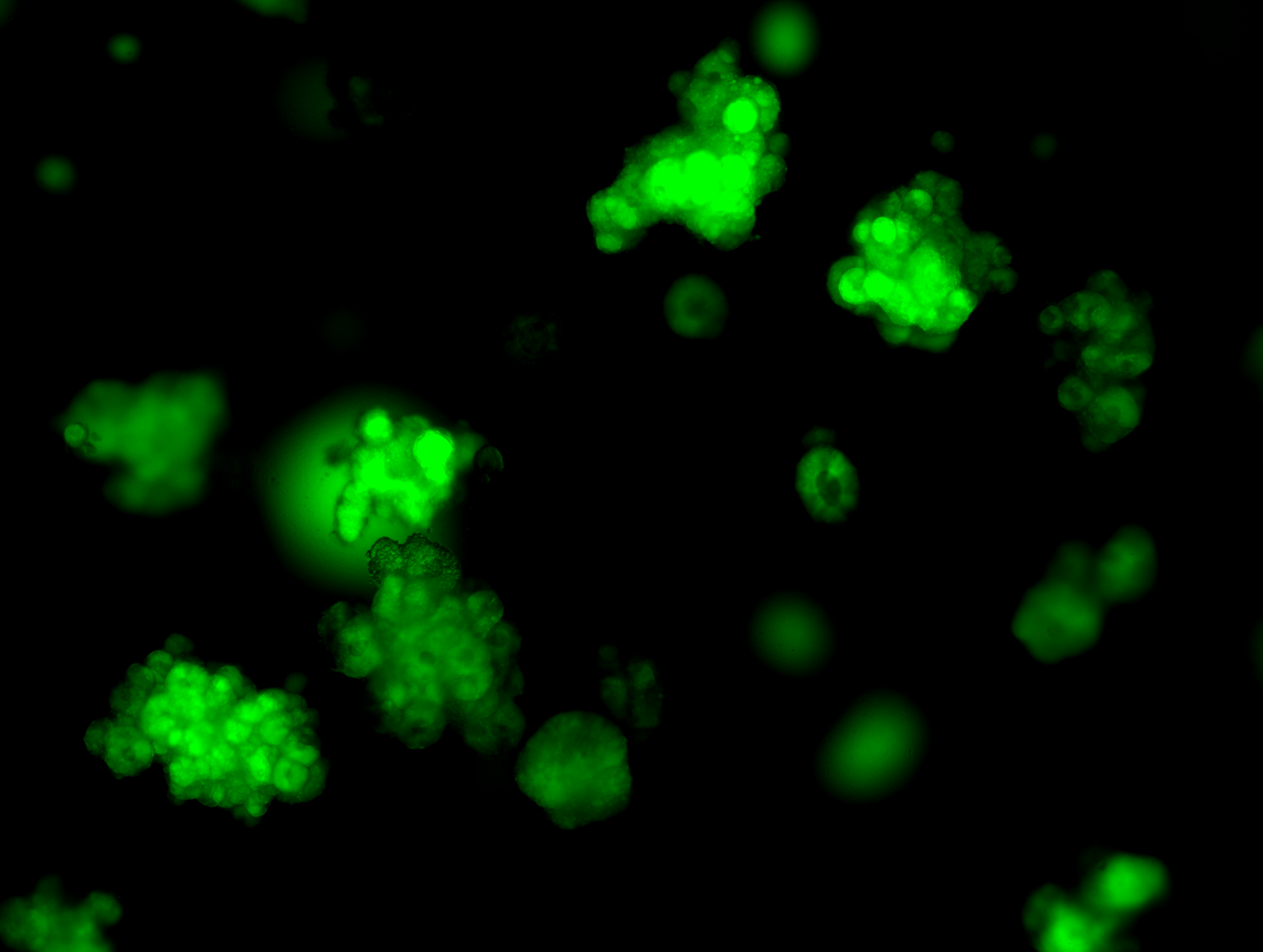

Supplement: Supplementary file 11 — Source data Fig. 6 [file 44318_2025_381_MOESM11_ESM.zip › Figure 6/6E/HD-3/HD3_shCTRL.png]

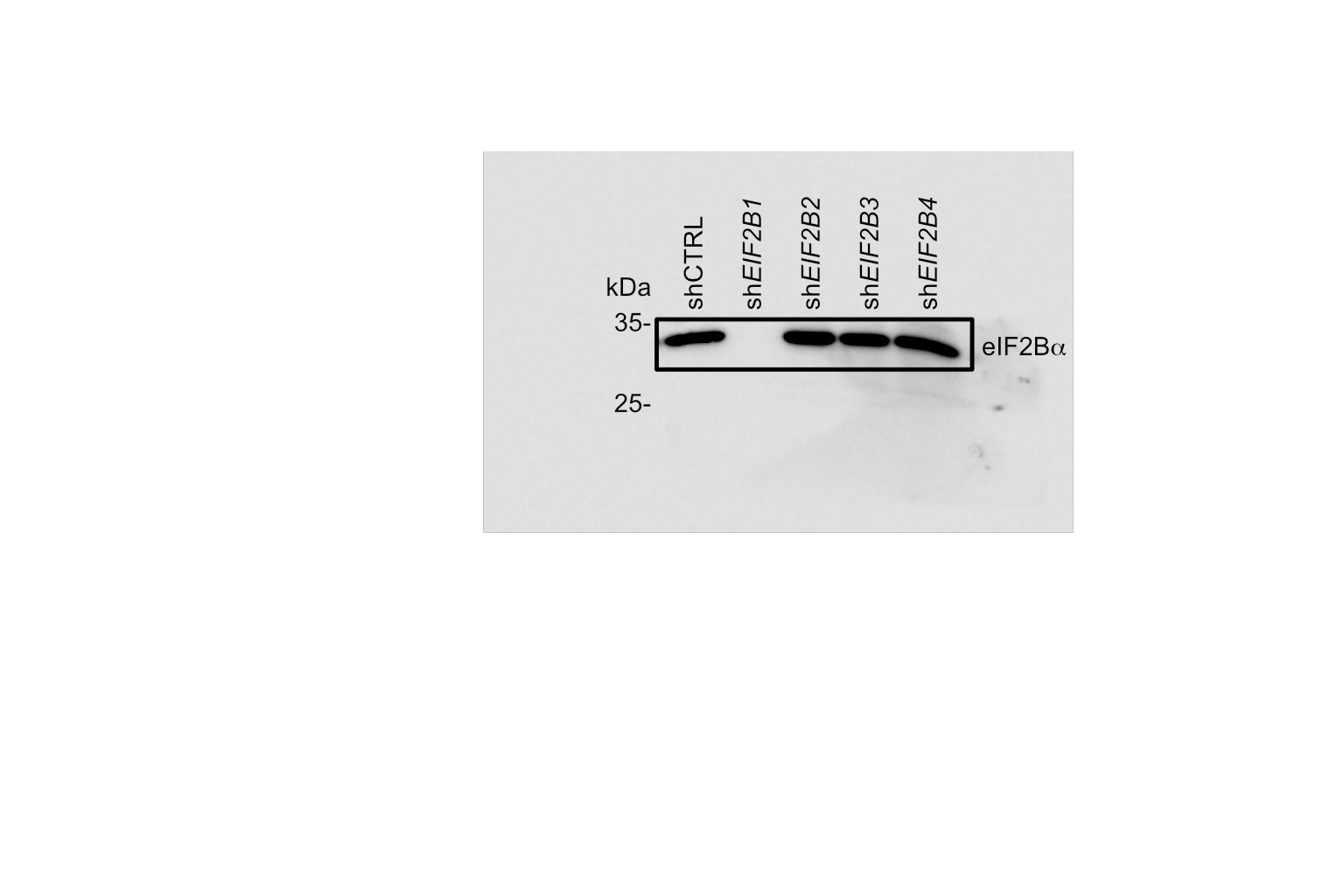

Supplement: Supplementary file 12 — EV Figure Source Data [file 44318_2025_381_MOESM12_ESM.zip › 44318_2025_381_MOESM12_ESM/Figure EV1/EV1B/western eIF2Ba.tif]

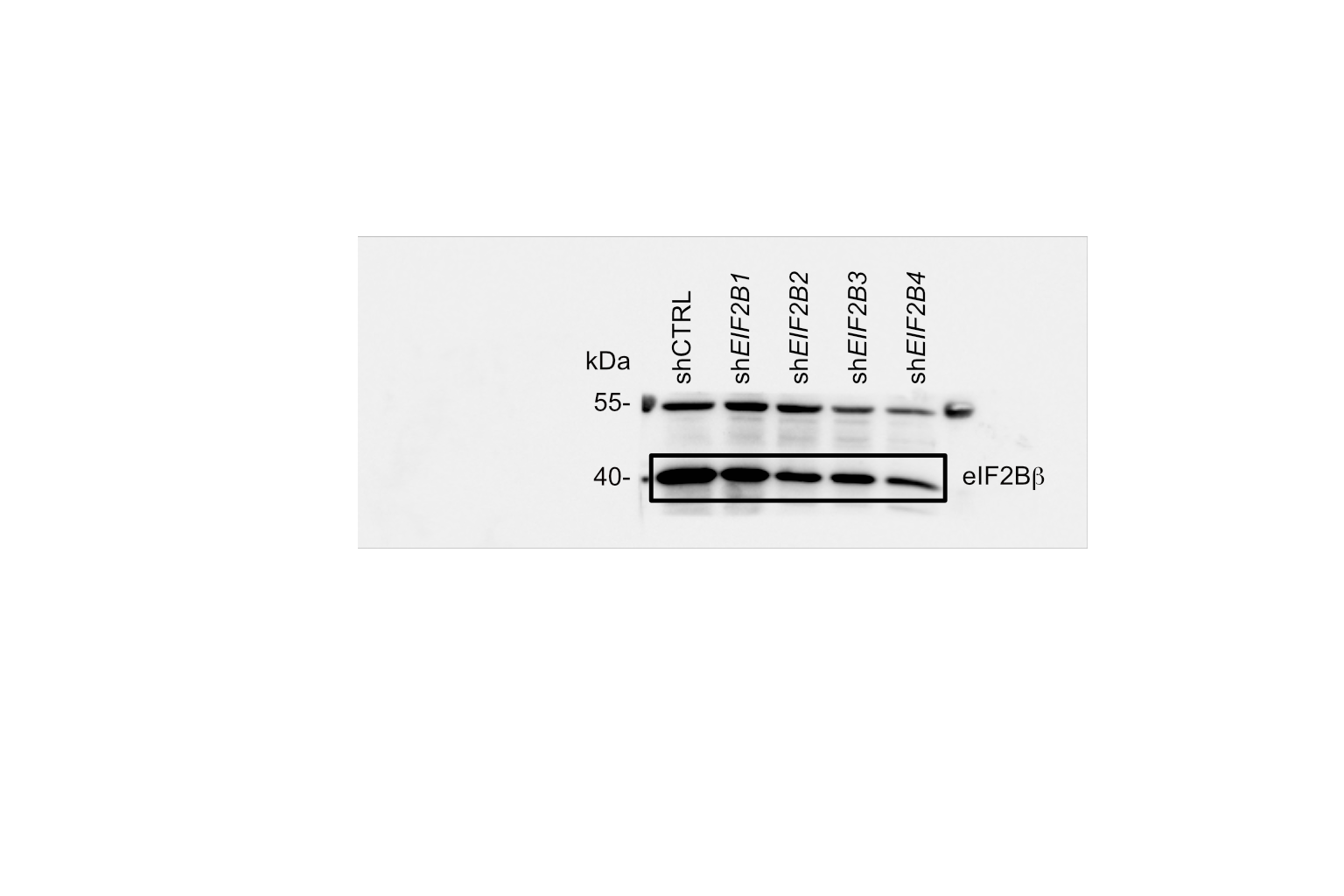

Supplement: Supplementary file 12 — EV Figure Source Data [file 44318_2025_381_MOESM12_ESM.zip › 44318_2025_381_MOESM12_ESM/Figure EV1/EV1B/western eIF2Bb.tif]

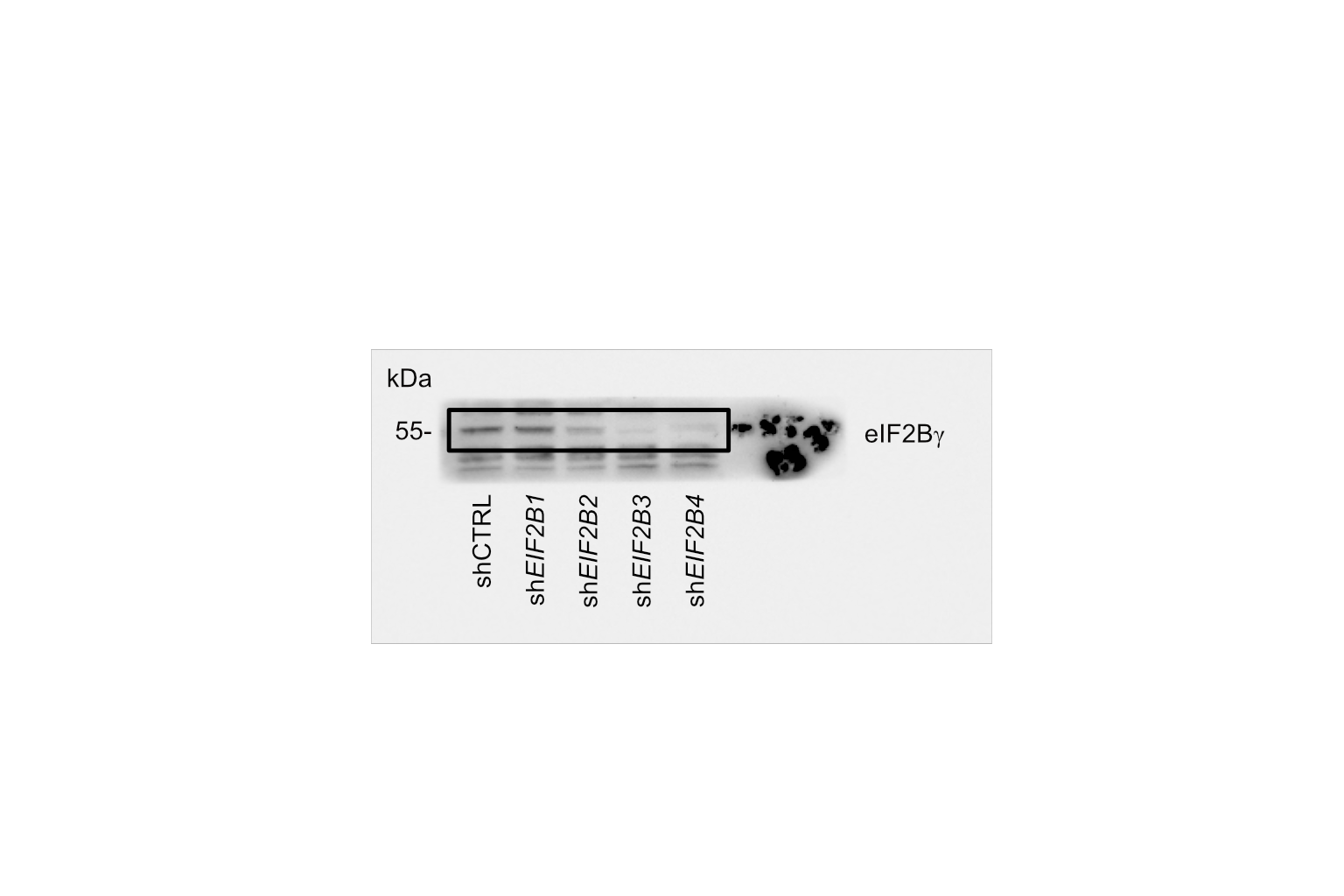

Supplement: Supplementary file 12 — EV Figure Source Data [file 44318_2025_381_MOESM12_ESM.zip › 44318_2025_381_MOESM12_ESM/Figure EV1/EV1B/western eIF2Bc.tif]

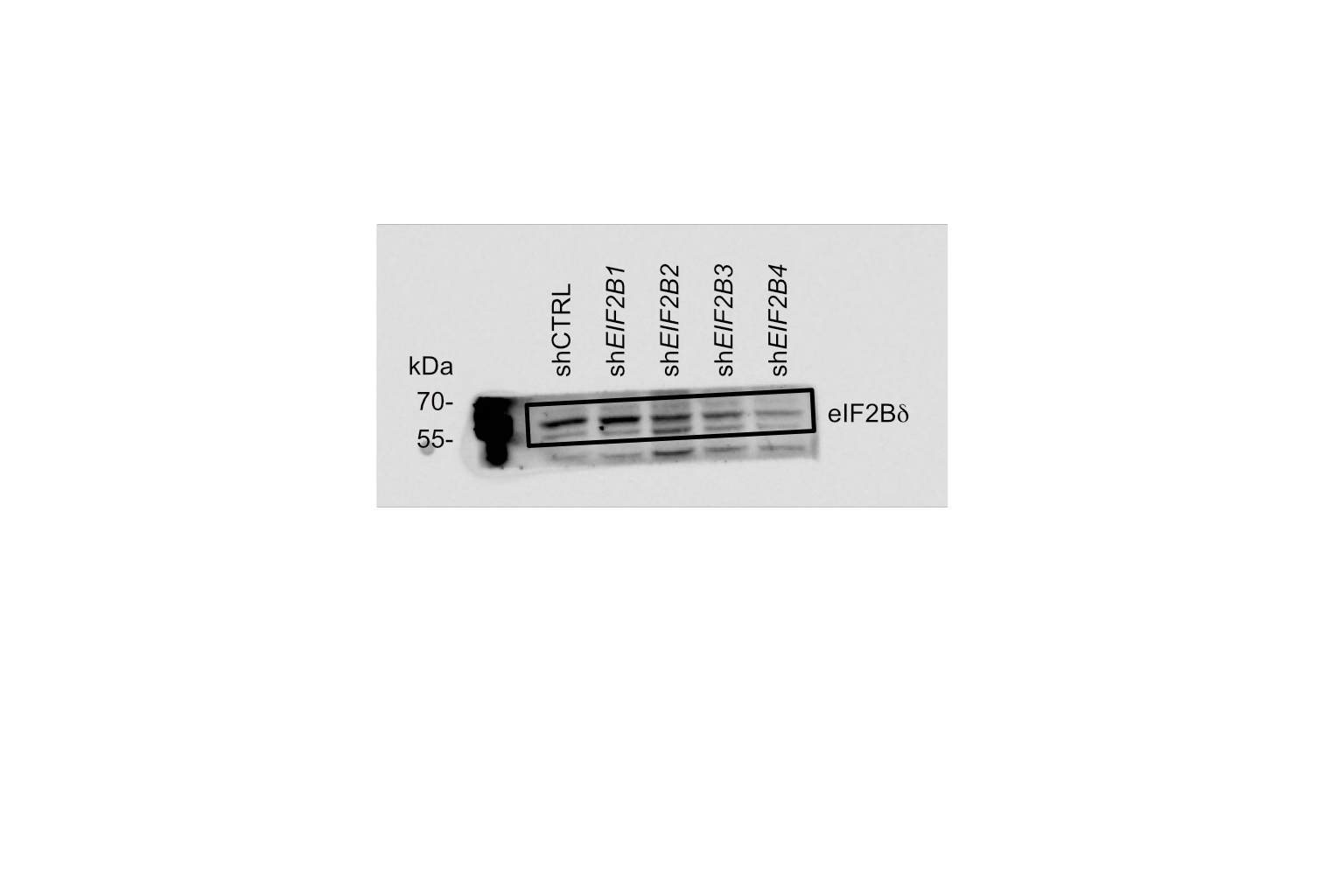

Supplement: Supplementary file 12 — EV Figure Source Data [file 44318_2025_381_MOESM12_ESM.zip › 44318_2025_381_MOESM12_ESM/Figure EV1/EV1B/western eIF2Bd.tif]

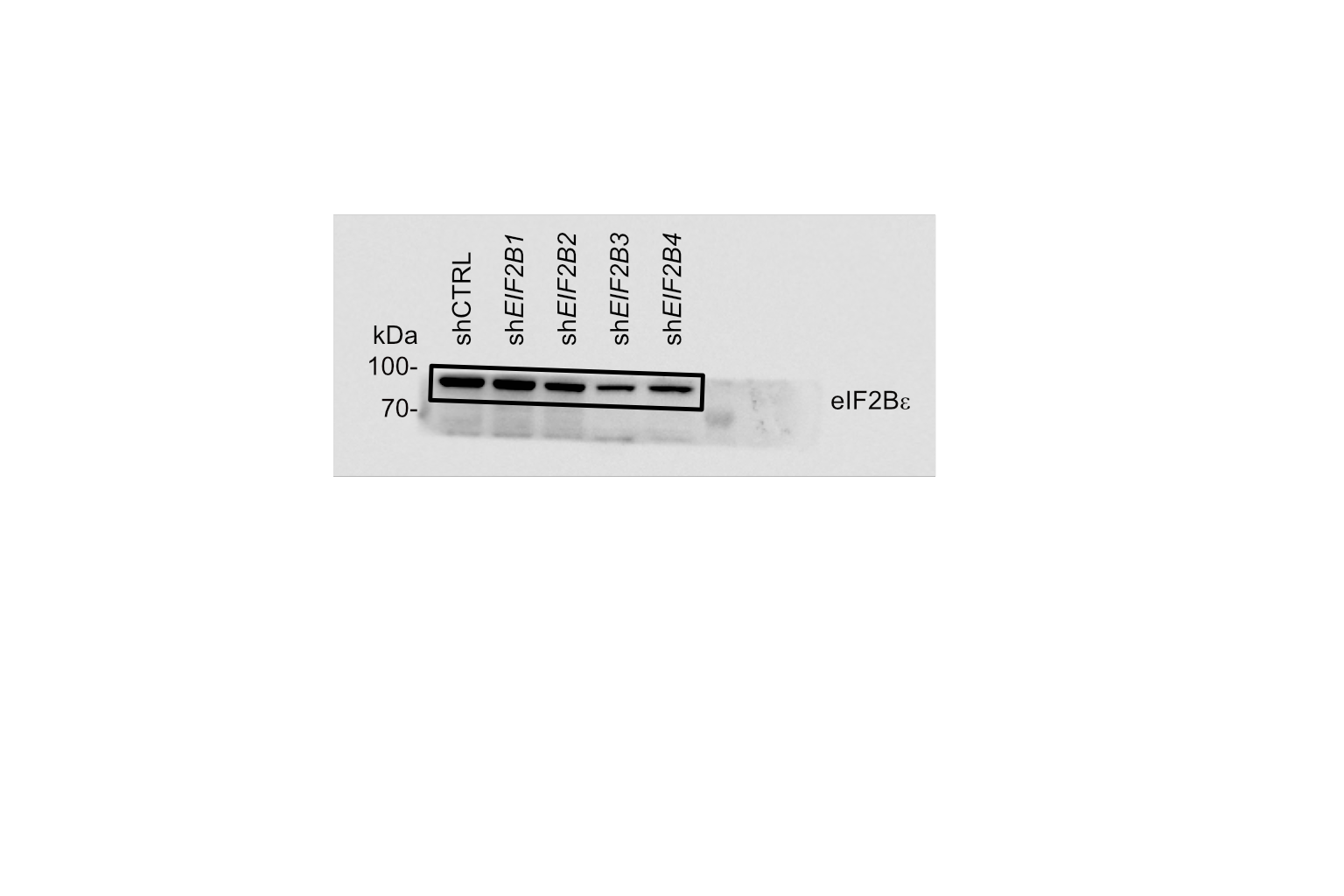

Supplement: Supplementary file 12 — EV Figure Source Data [file 44318_2025_381_MOESM12_ESM.zip › 44318_2025_381_MOESM12_ESM/Figure EV1/EV1B/western eIF2Be.tif]

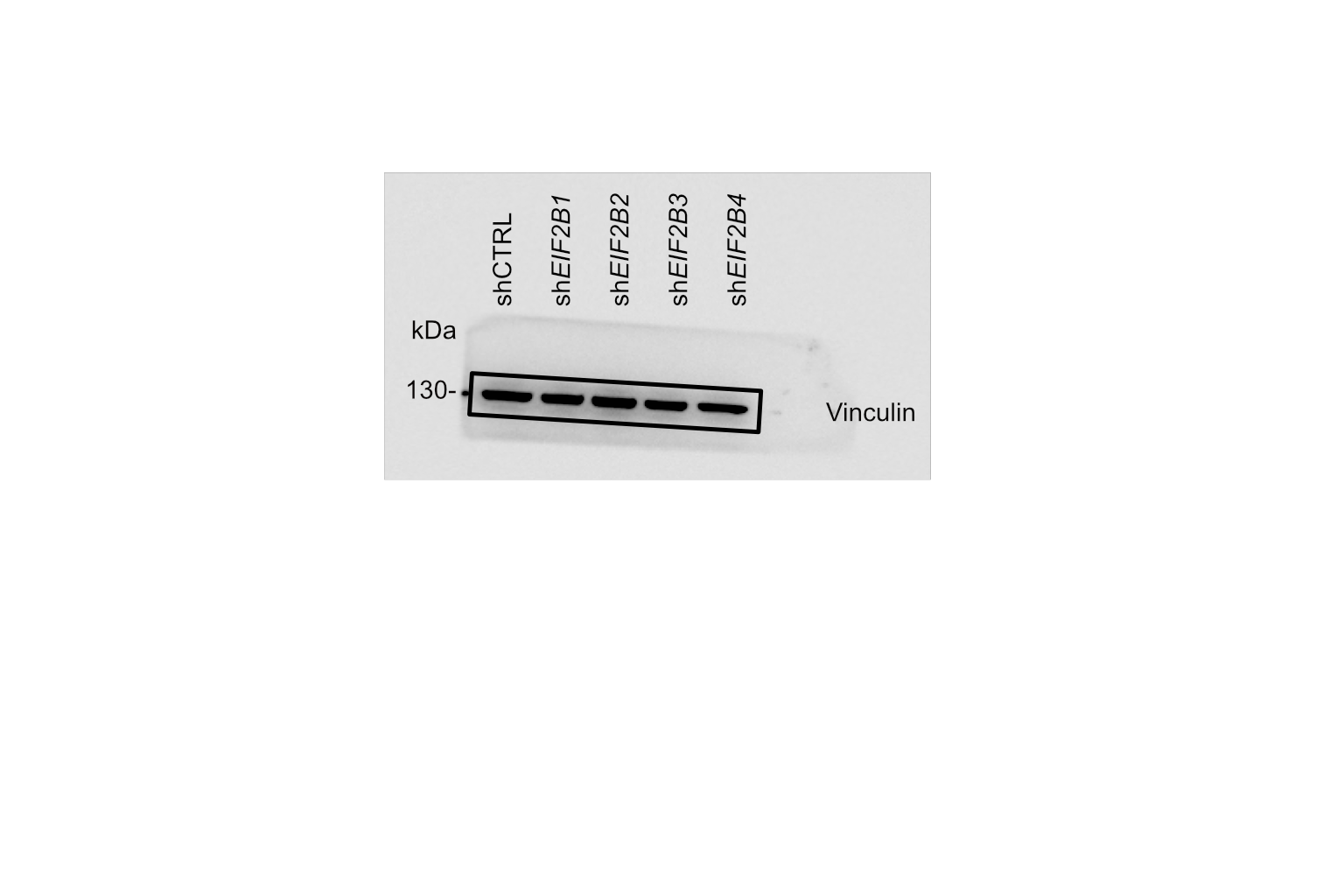

Supplement: Supplementary file 12 — EV Figure Source Data [file 44318_2025_381_MOESM12_ESM.zip › 44318_2025_381_MOESM12_ESM/Figure EV1/EV1B/western vinculin.tif]

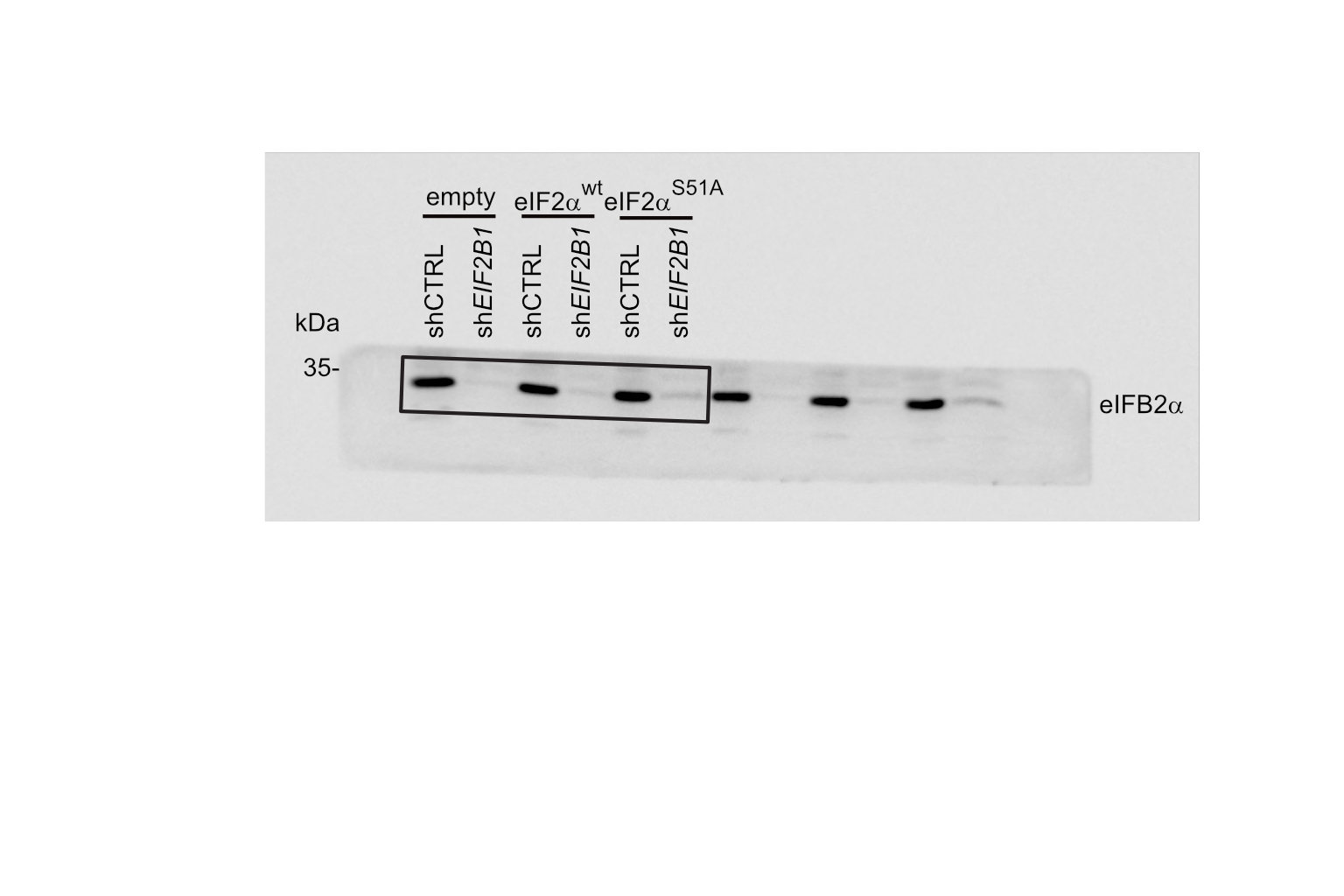

Supplement: Supplementary file 12 — EV Figure Source Data [file 44318_2025_381_MOESM12_ESM.zip › 44318_2025_381_MOESM12_ESM/Figure EV2/EV2A/western eIF2Ba.tif]

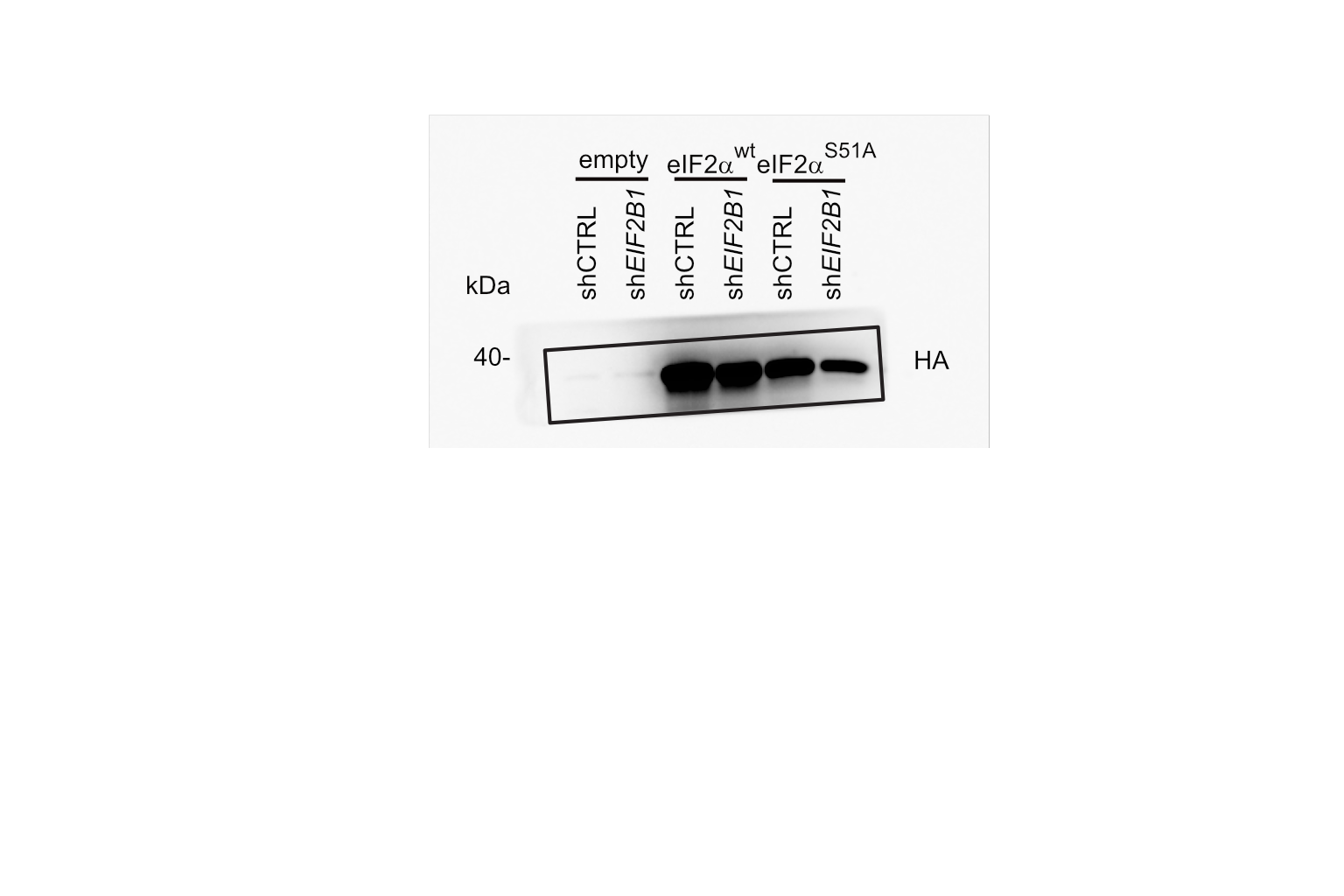

Supplement: Supplementary file 12 — EV Figure Source Data [file 44318_2025_381_MOESM12_ESM.zip › 44318_2025_381_MOESM12_ESM/Figure EV2/EV2A/western HA.tif]

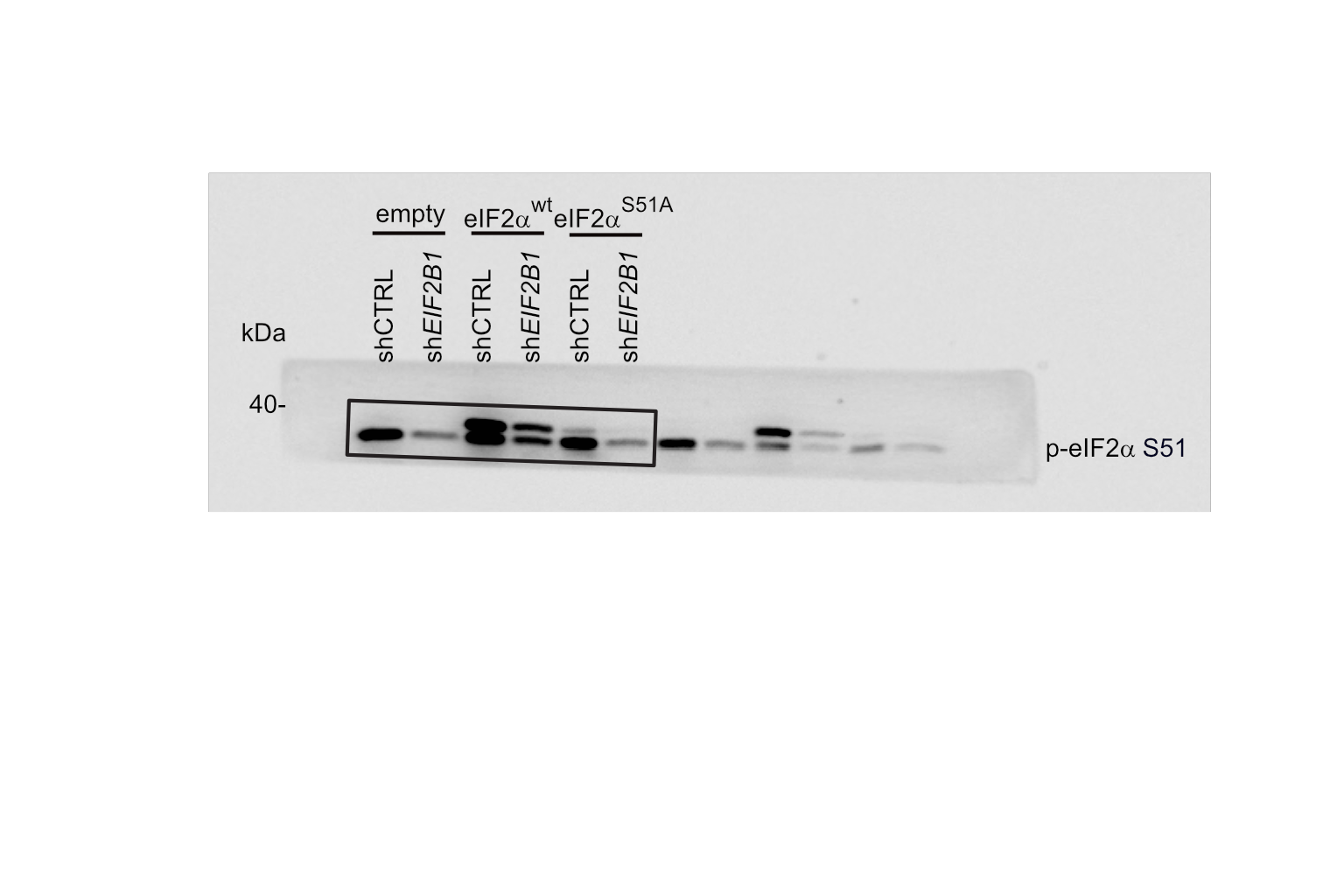

Supplement: Supplementary file 12 — EV Figure Source Data [file 44318_2025_381_MOESM12_ESM.zip › 44318_2025_381_MOESM12_ESM/Figure EV2/EV2A/western p-eIF2a S51.tif]

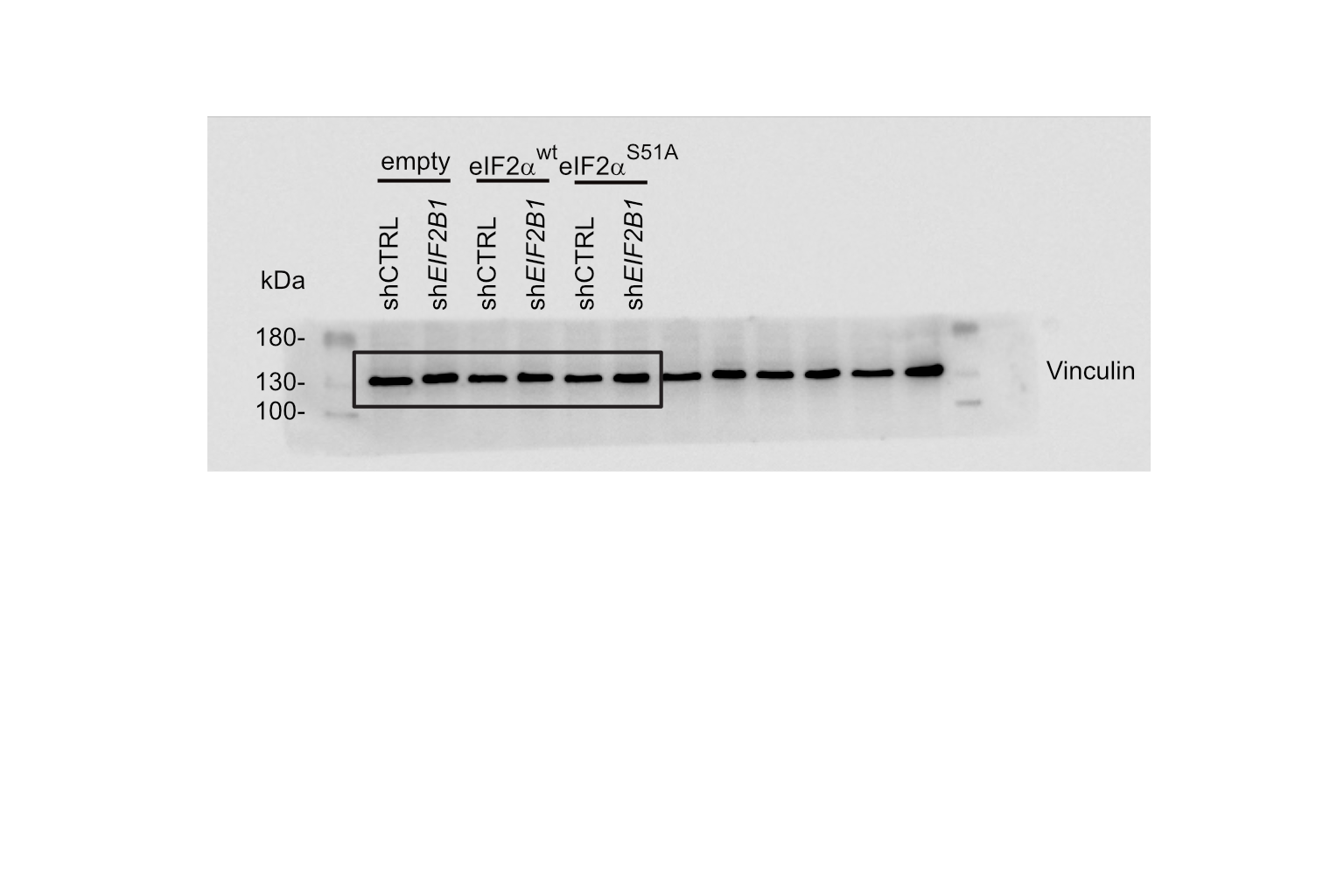

Supplement: Supplementary file 12 — EV Figure Source Data [file 44318_2025_381_MOESM12_ESM.zip › 44318_2025_381_MOESM12_ESM/Figure EV2/EV2A/western vinculin.tif]

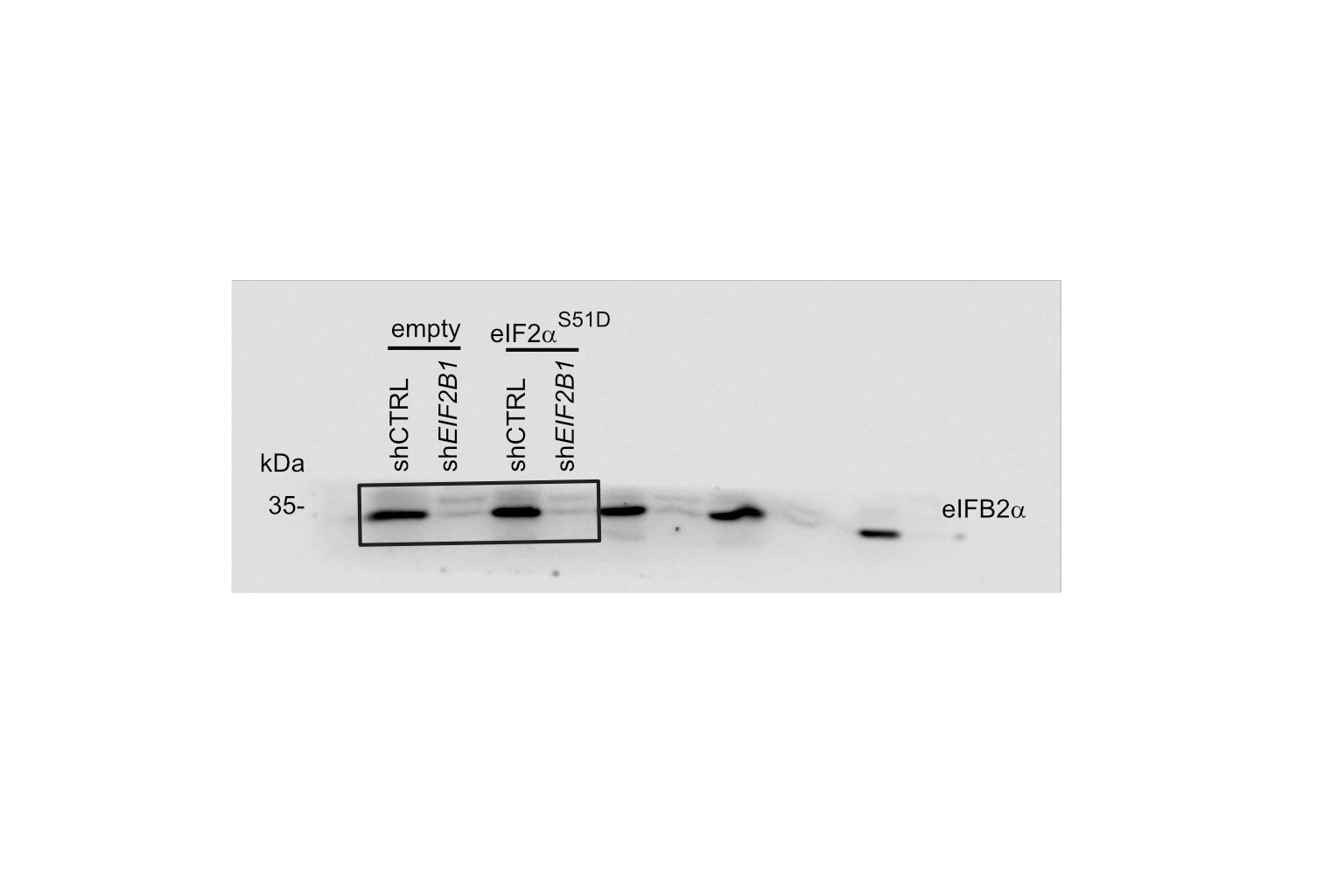

Supplement: Supplementary file 12 — EV Figure Source Data [file 44318_2025_381_MOESM12_ESM.zip › 44318_2025_381_MOESM12_ESM/Figure EV2/EV2C/western eIF2Ba.tif]

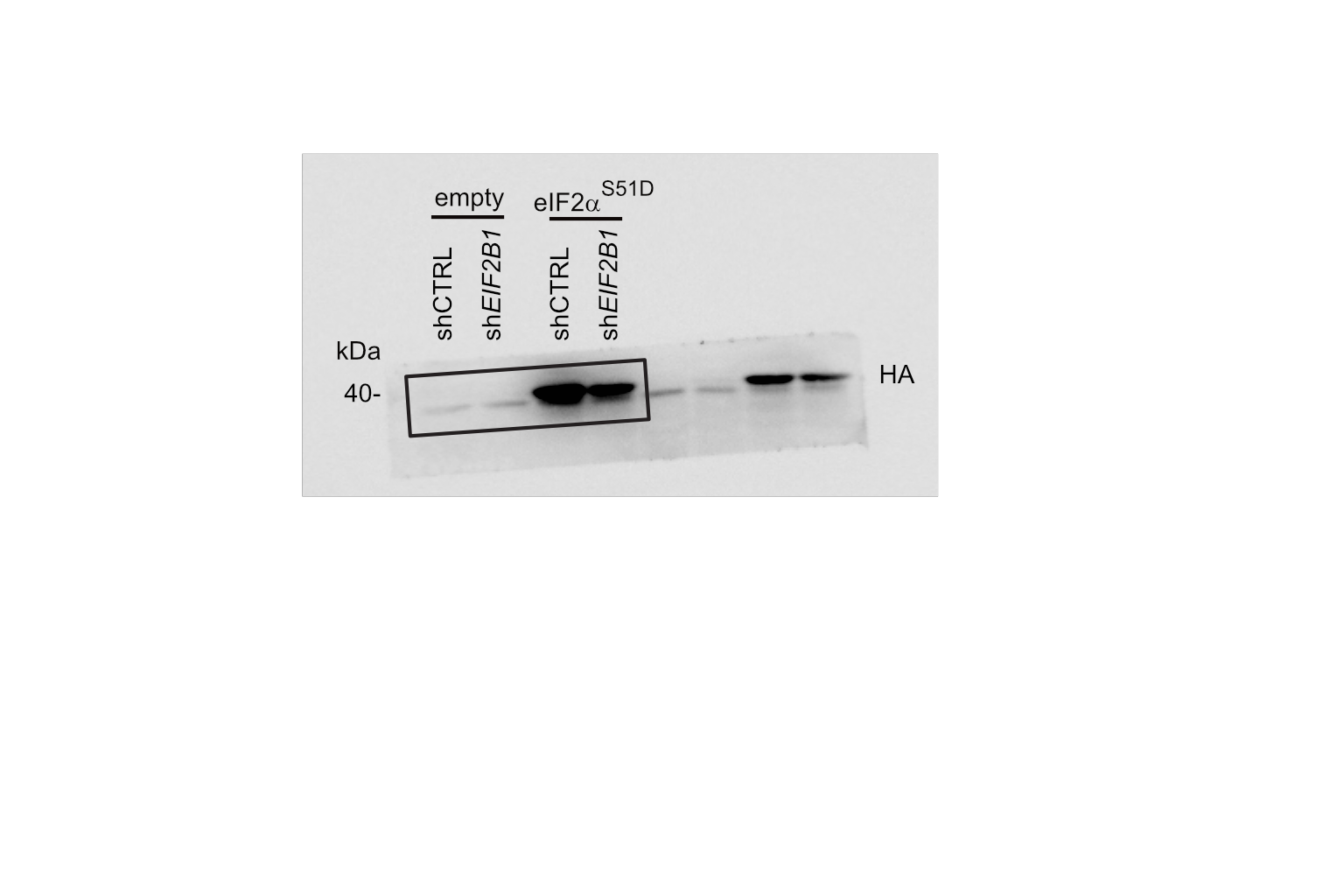

Supplement: Supplementary file 12 — EV Figure Source Data [file 44318_2025_381_MOESM12_ESM.zip › 44318_2025_381_MOESM12_ESM/Figure EV2/EV2C/western HA.tif]

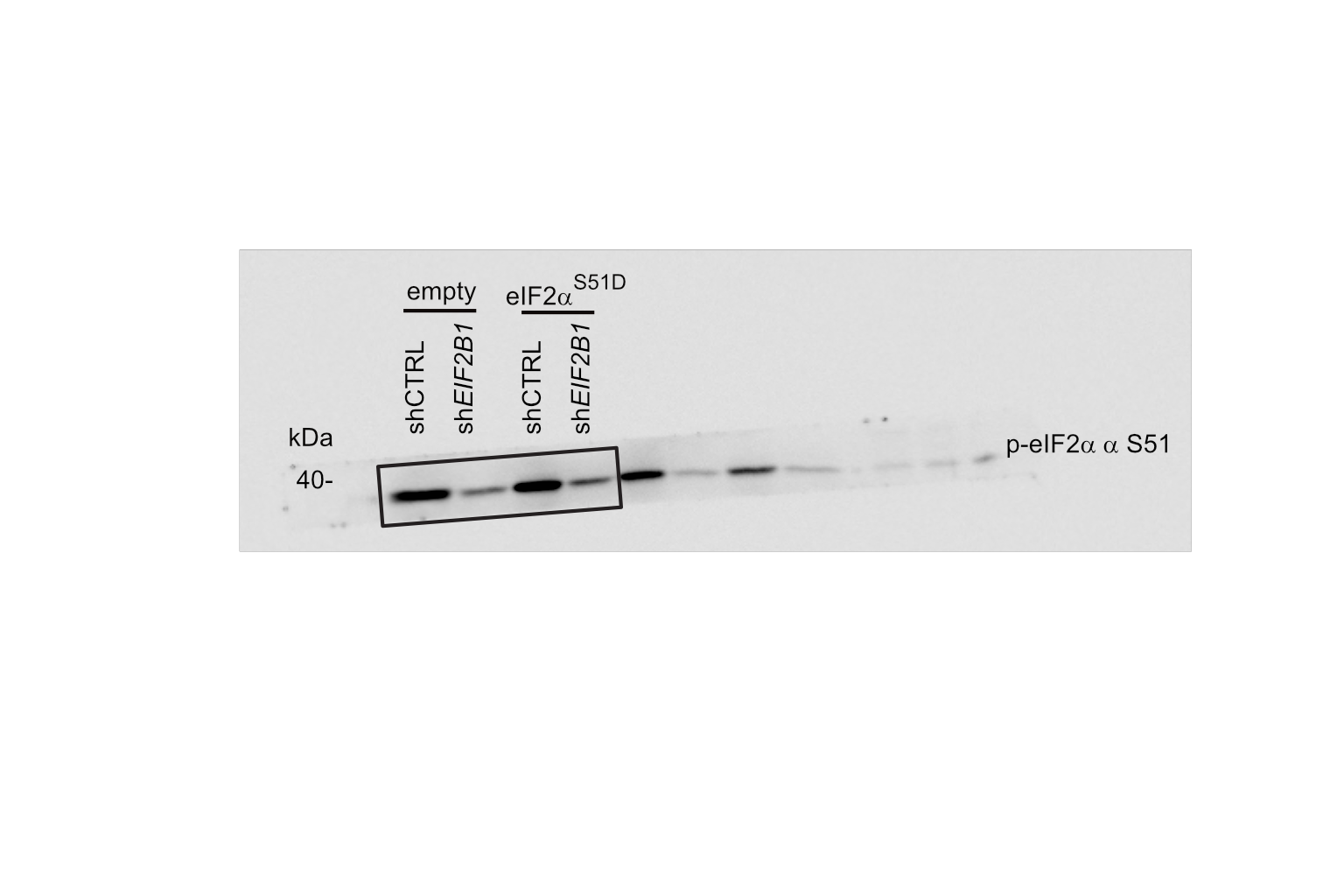

Supplement: Supplementary file 12 — EV Figure Source Data [file 44318_2025_381_MOESM12_ESM.zip › 44318_2025_381_MOESM12_ESM/Figure EV2/EV2C/western p-eIF2a S51.tif]

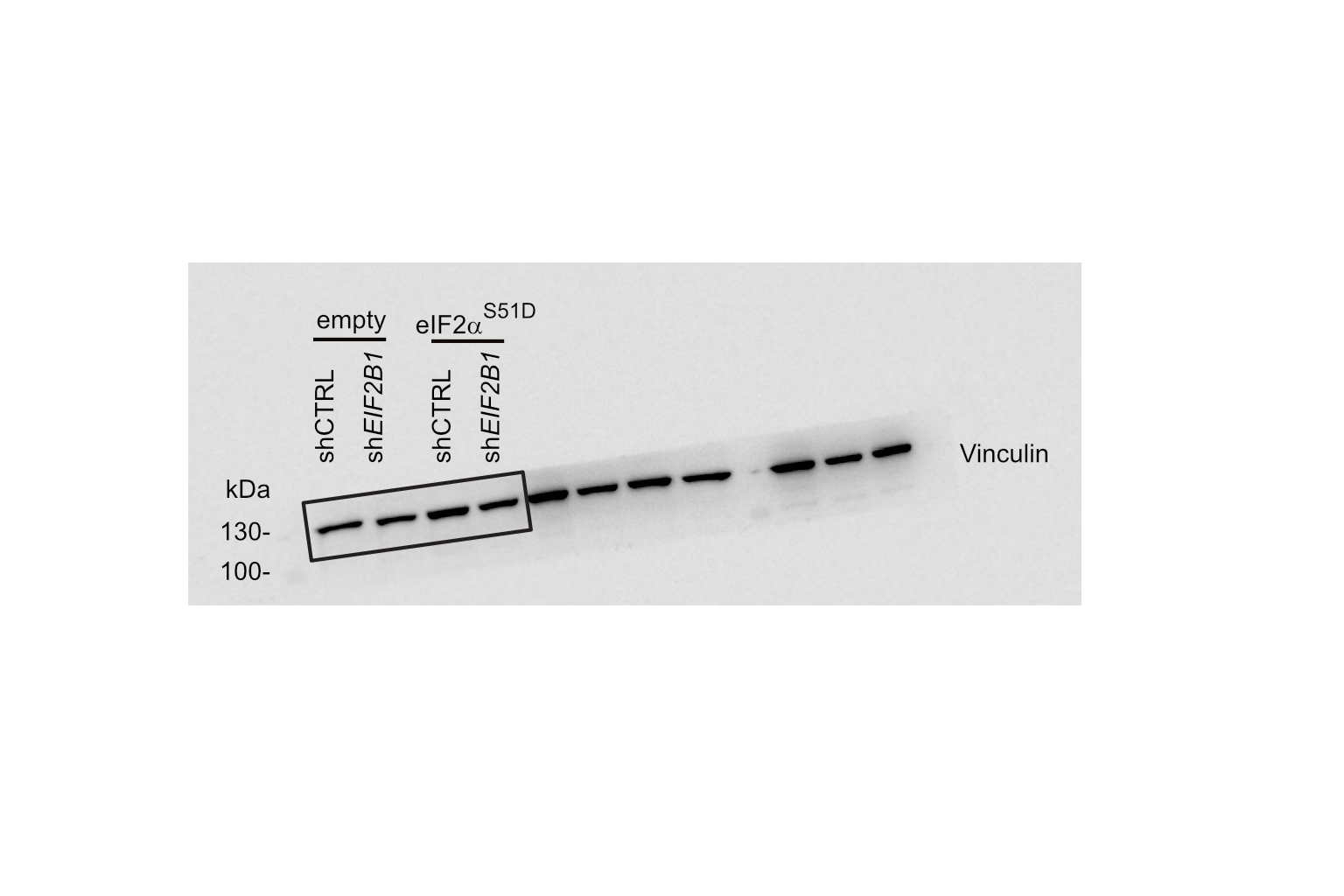

Supplement: Supplementary file 12 — EV Figure Source Data [file 44318_2025_381_MOESM12_ESM.zip › 44318_2025_381_MOESM12_ESM/Figure EV2/EV2C/western vinculin.tif]

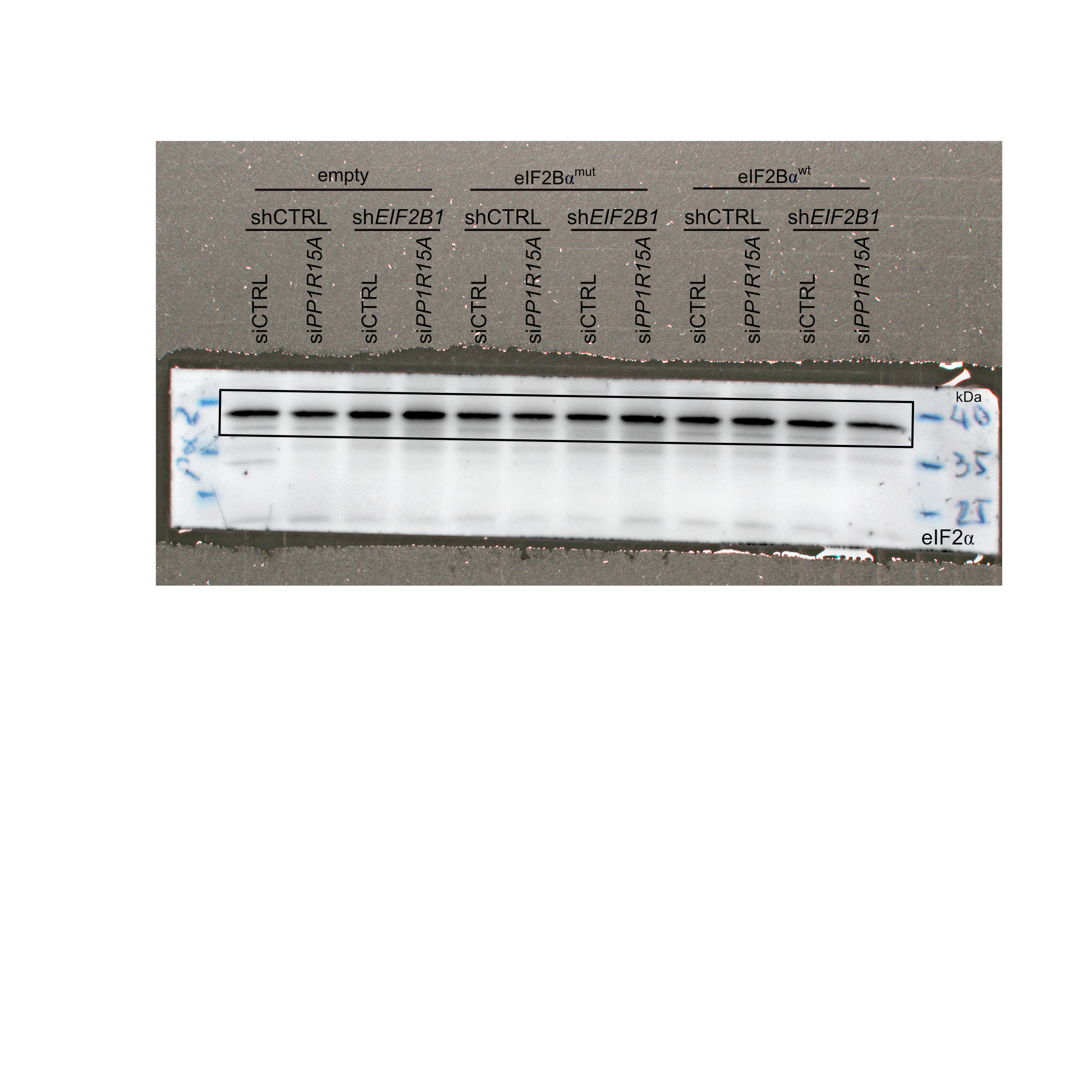

Supplement: Supplementary file 12 — EV Figure Source Data [file 44318_2025_381_MOESM12_ESM.zip › 44318_2025_381_MOESM12_ESM/Figure EV2/EV2F/western eIF2a.tiff]

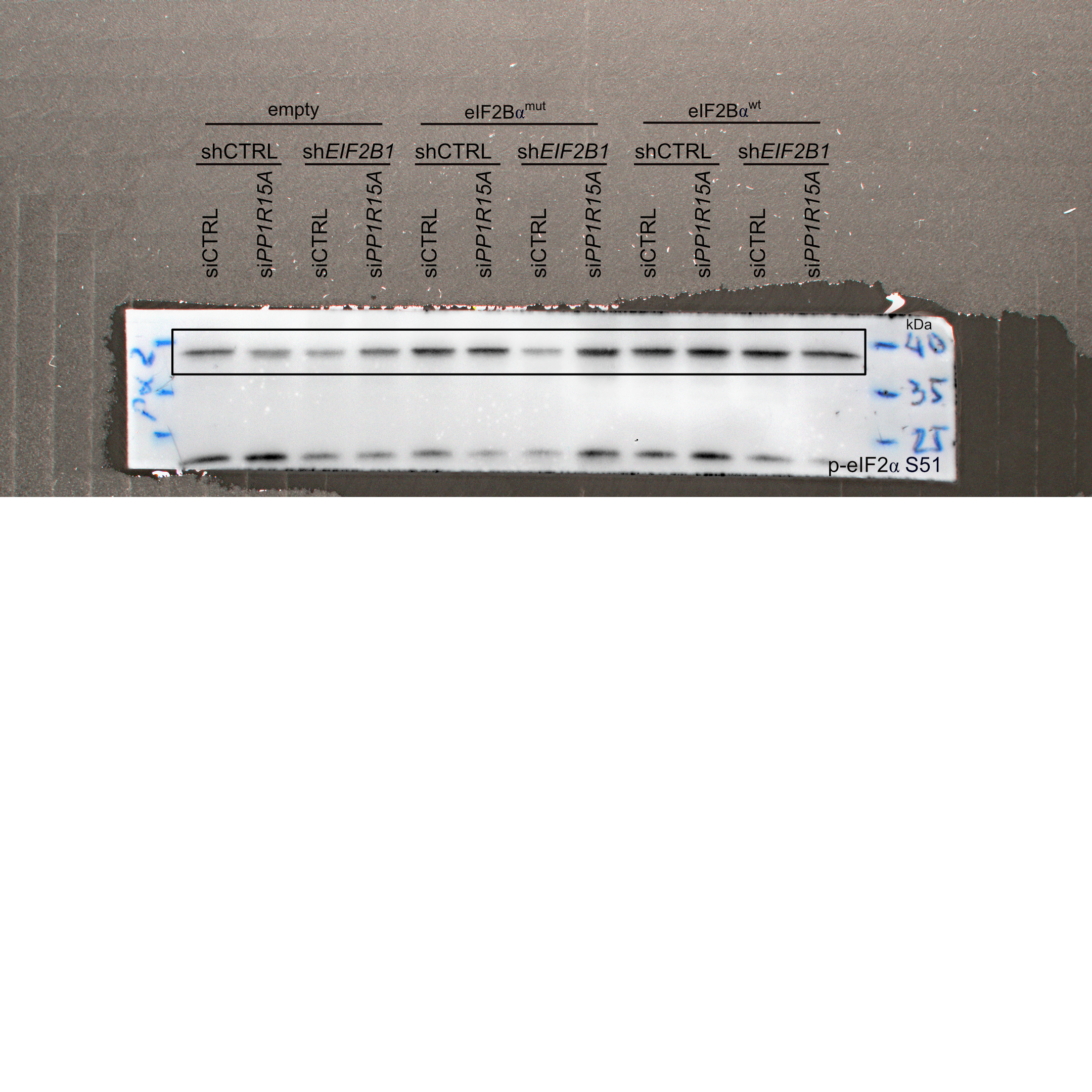

Supplement: Supplementary file 12 — EV Figure Source Data [file 44318_2025_381_MOESM12_ESM.zip › 44318_2025_381_MOESM12_ESM/Figure EV2/EV2F/western p-eIF2a S51.tiff]

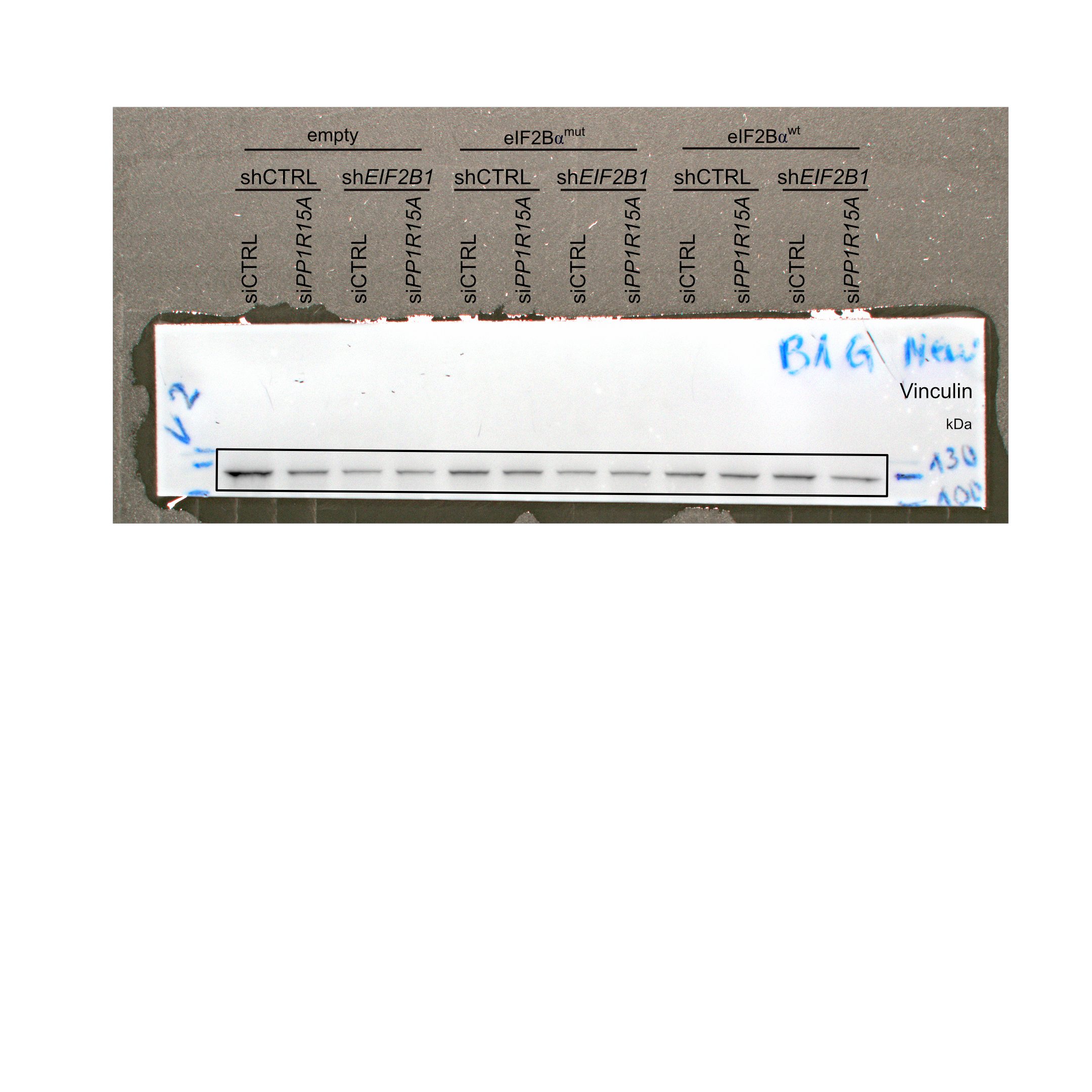

Supplement: Supplementary file 12 — EV Figure Source Data [file 44318_2025_381_MOESM12_ESM.zip › 44318_2025_381_MOESM12_ESM/Figure EV2/EV2F/western vinculin.tiff]

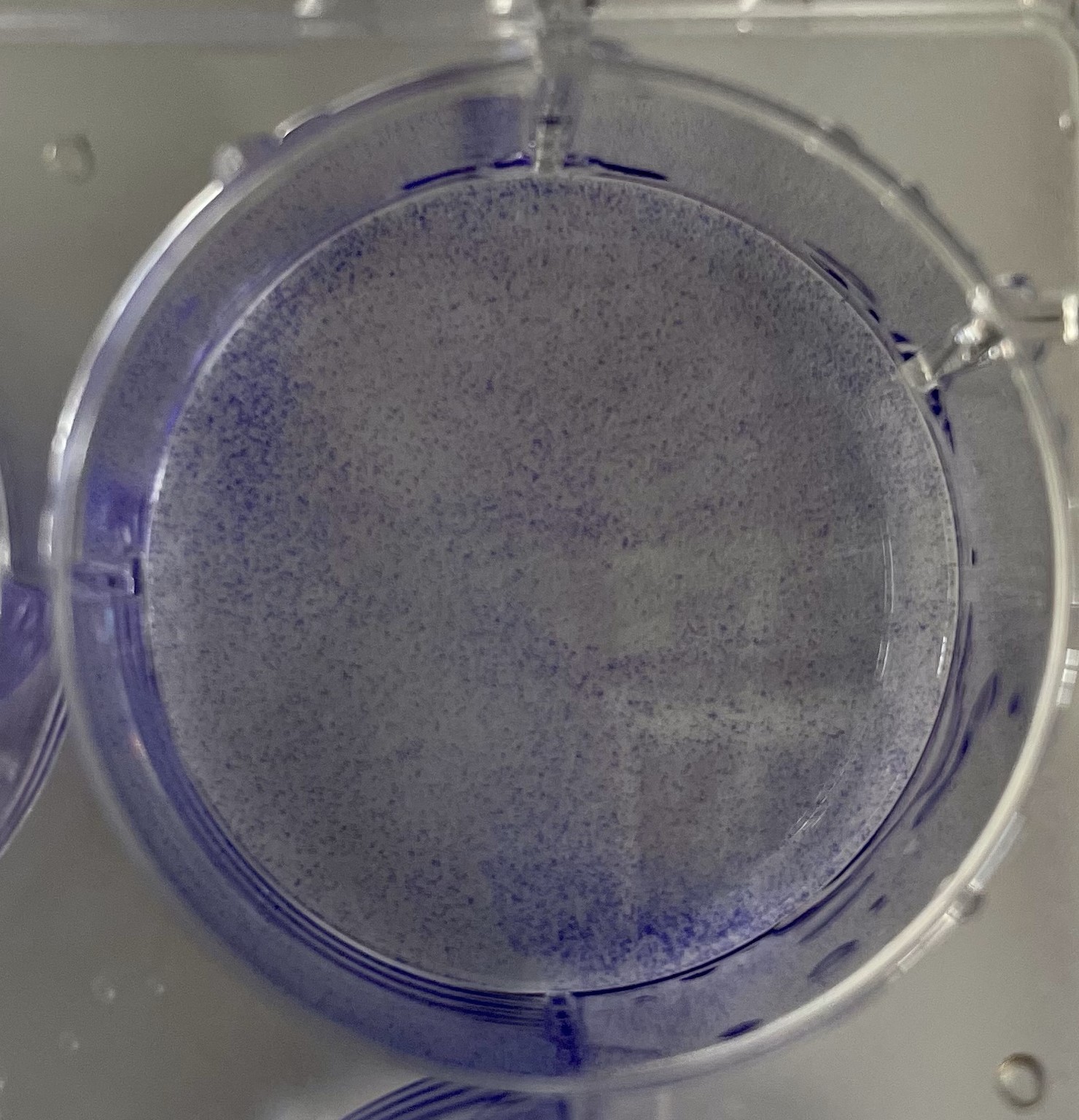

Supplement: Supplementary file 12 — EV Figure Source Data [file 44318_2025_381_MOESM12_ESM.zip › 44318_2025_381_MOESM12_ESM/Figure EV2/EV2G/eIF2Bamut shCTRL siCTRL.jpg]

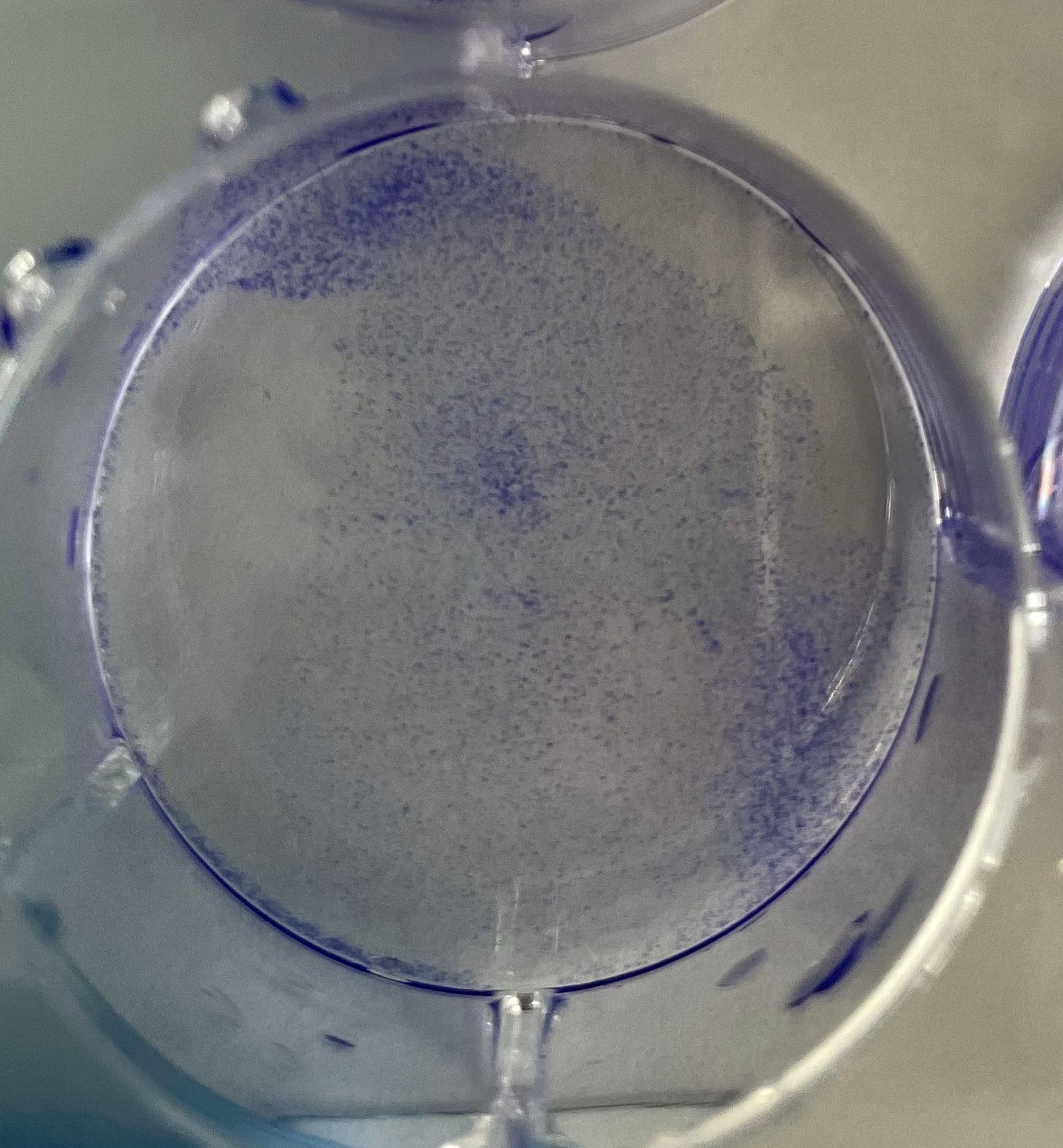

Supplement: Supplementary file 12 — EV Figure Source Data [file 44318_2025_381_MOESM12_ESM.zip › 44318_2025_381_MOESM12_ESM/Figure EV2/EV2G/eIF2Bamut shCTRL siPPP1R15A.jpg]

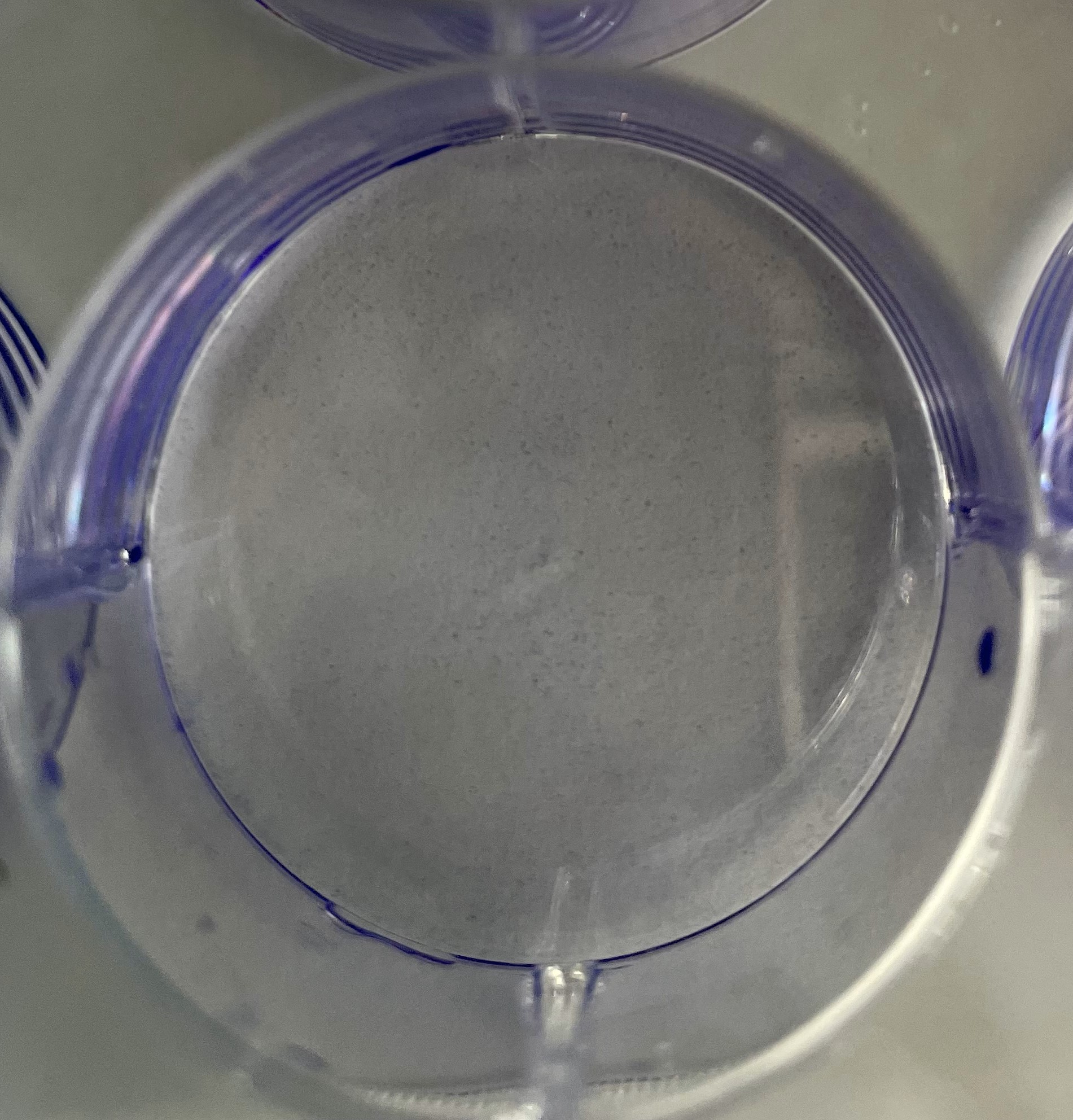

Supplement: Supplementary file 12 — EV Figure Source Data [file 44318_2025_381_MOESM12_ESM.zip › 44318_2025_381_MOESM12_ESM/Figure EV2/EV2G/eIF2Bamut shEIF2B1 siCTRL.jpg]

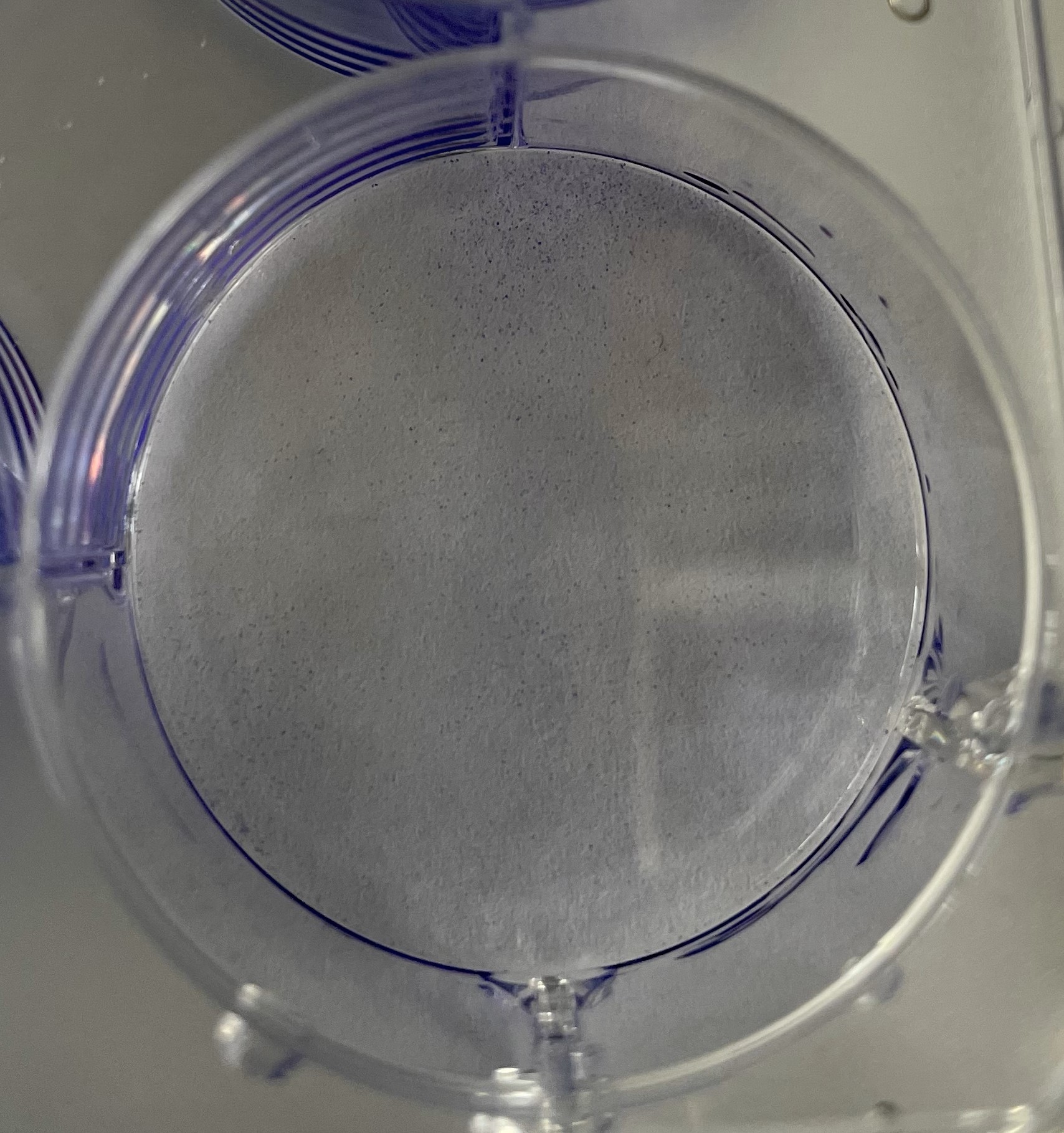

Supplement: Supplementary file 12 — EV Figure Source Data [file 44318_2025_381_MOESM12_ESM.zip › 44318_2025_381_MOESM12_ESM/Figure EV2/EV2G/eIF2Bamut shEIF2B1 siPPP1R15A.jpg]

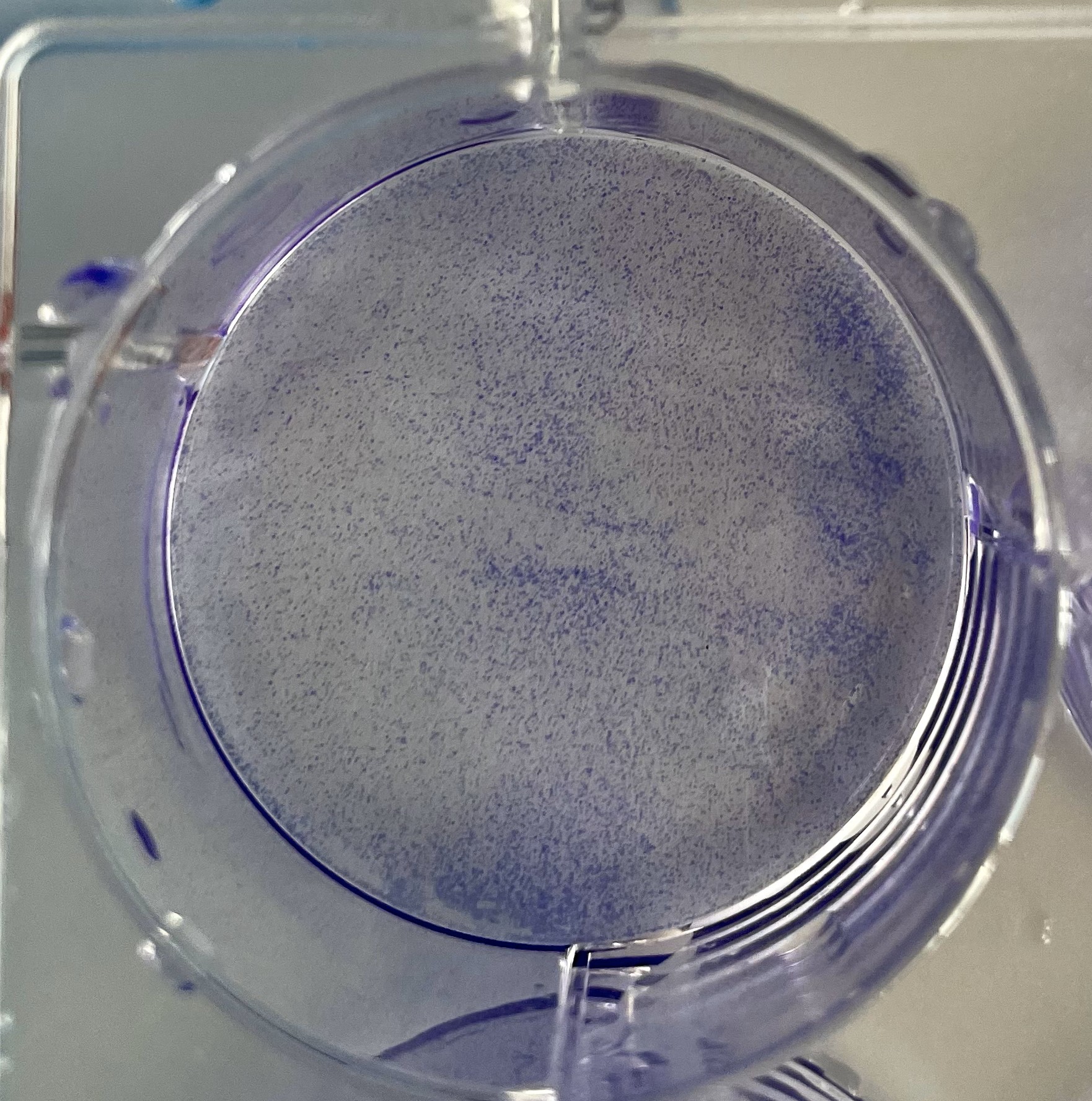

Supplement: Supplementary file 12 — EV Figure Source Data [file 44318_2025_381_MOESM12_ESM.zip › 44318_2025_381_MOESM12_ESM/Figure EV2/EV2G/eIF2Bawt shCTRL siCTRL.jpg]

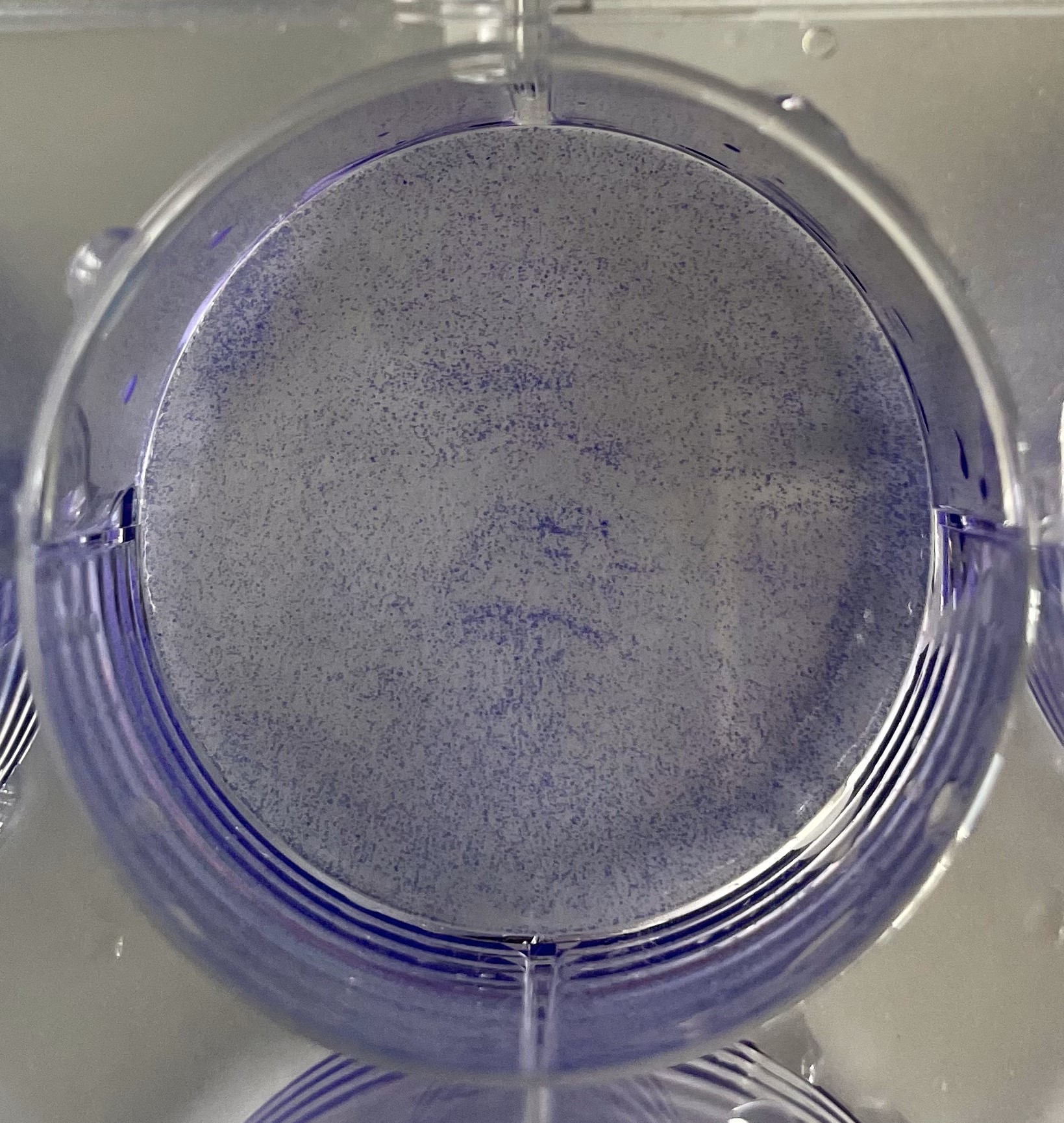

Supplement: Supplementary file 12 — EV Figure Source Data [file 44318_2025_381_MOESM12_ESM.zip › 44318_2025_381_MOESM12_ESM/Figure EV2/EV2G/eIF2Bawt shCTRL siPPP1R15A.jpg]

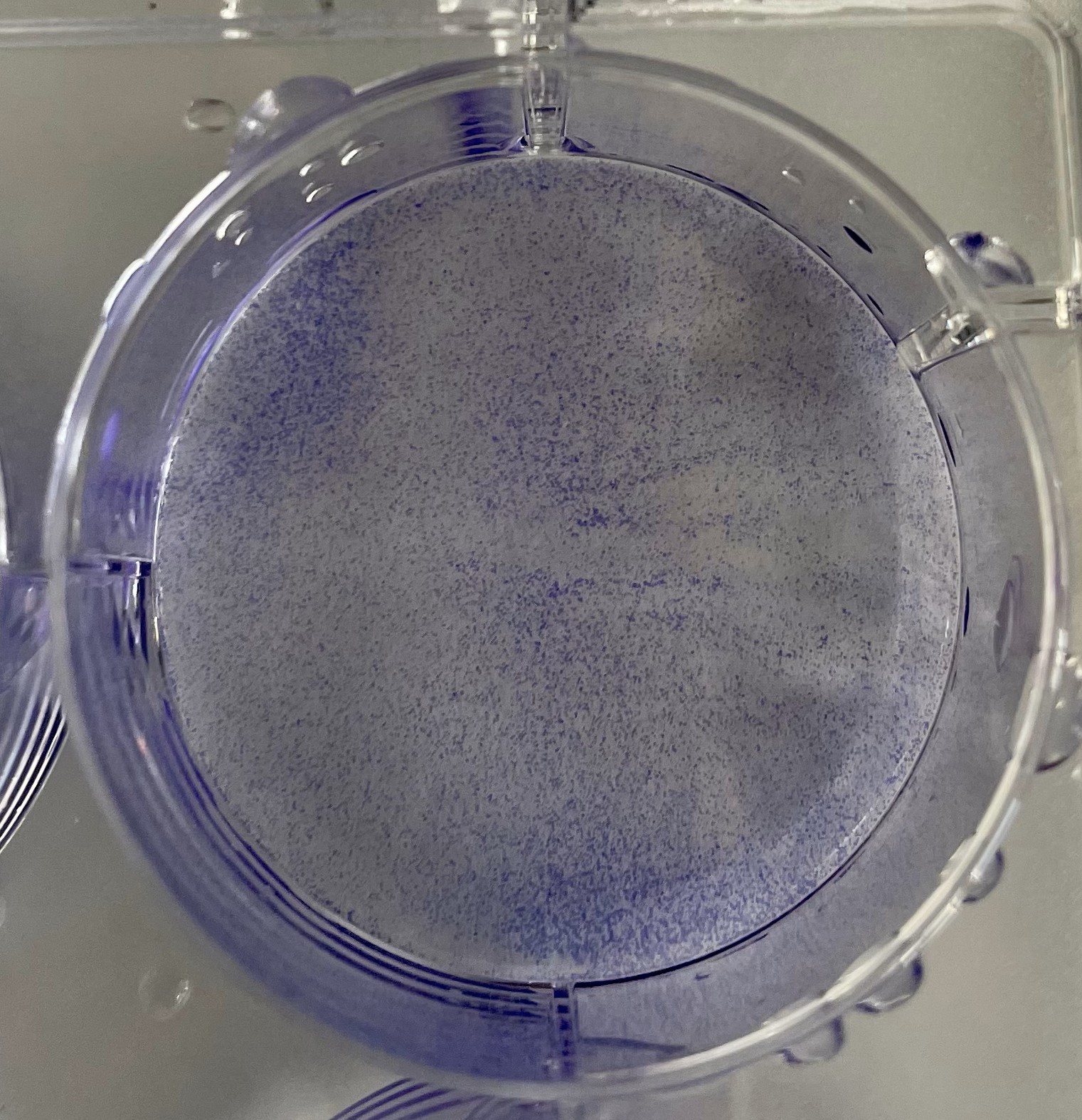

Supplement: Supplementary file 12 — EV Figure Source Data [file 44318_2025_381_MOESM12_ESM.zip › 44318_2025_381_MOESM12_ESM/Figure EV2/EV2G/eIF2Bawt shEIF2B1 siCTRL.jpg]

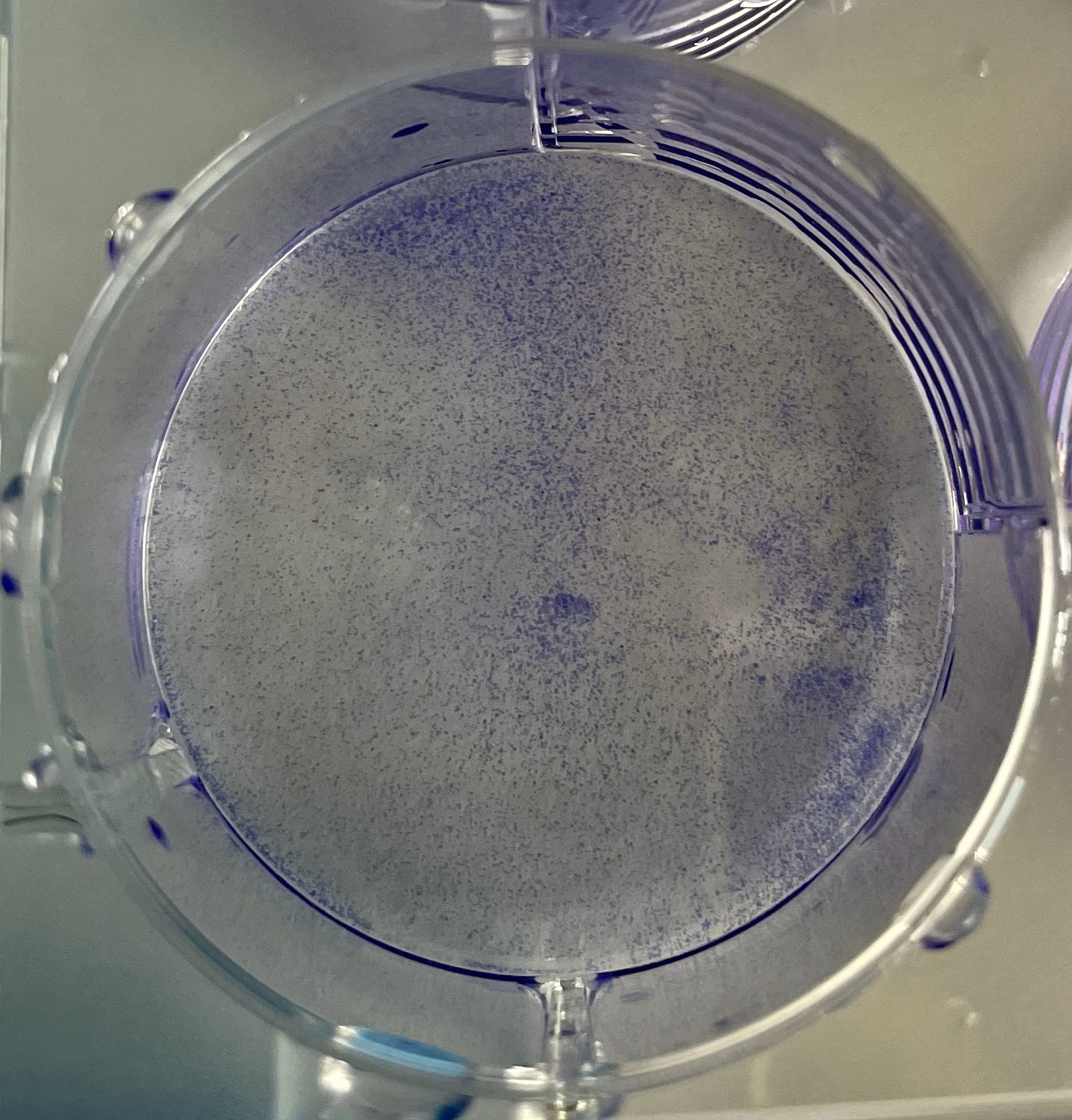

Supplement: Supplementary file 12 — EV Figure Source Data [file 44318_2025_381_MOESM12_ESM.zip › 44318_2025_381_MOESM12_ESM/Figure EV2/EV2G/eIF2Bawt shEIF2B1 siPPP1R15A.jpg]

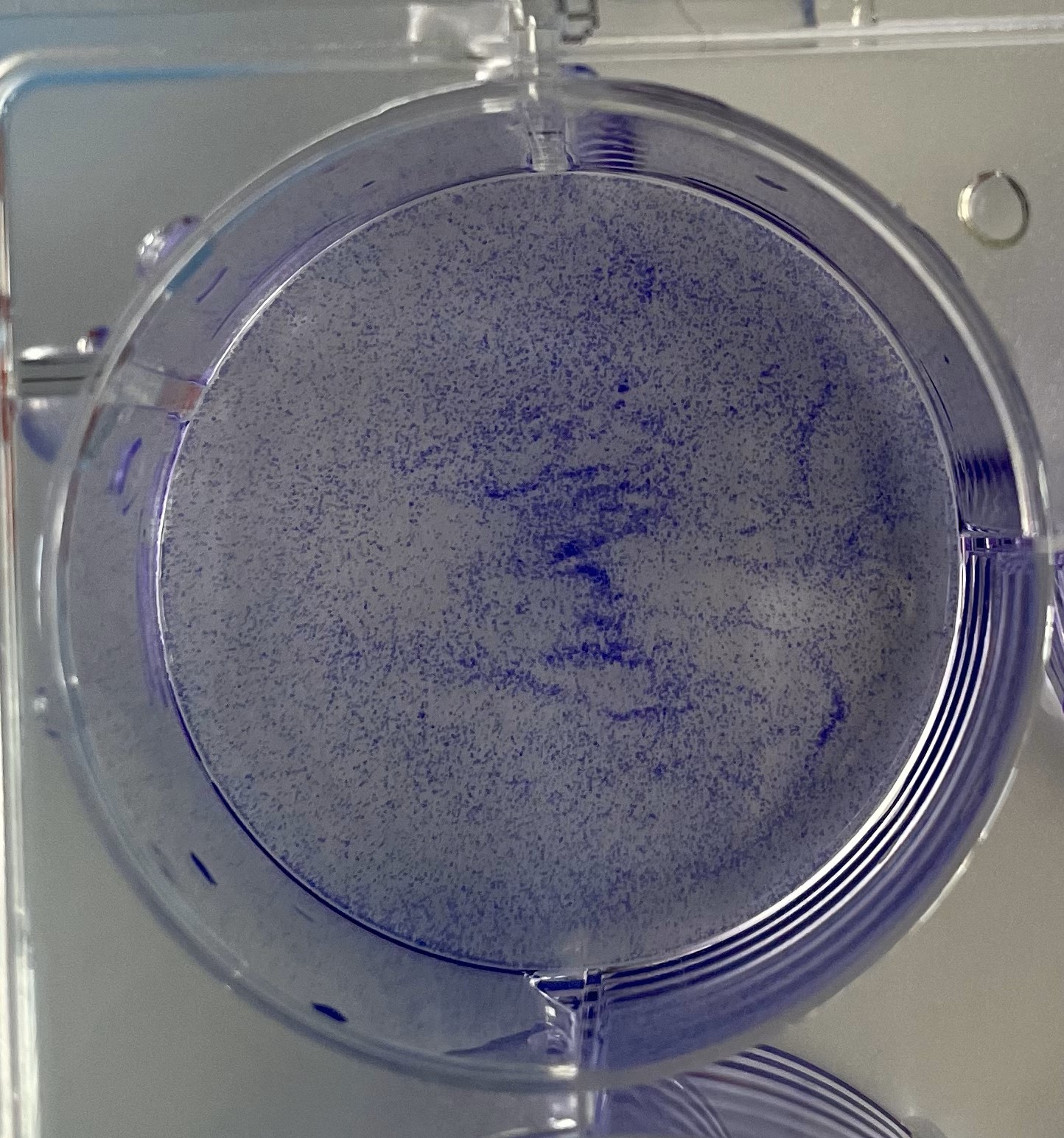

Supplement: Supplementary file 12 — EV Figure Source Data [file 44318_2025_381_MOESM12_ESM.zip › 44318_2025_381_MOESM12_ESM/Figure EV2/EV2G/empty shCTRL siCTRL.jpg]

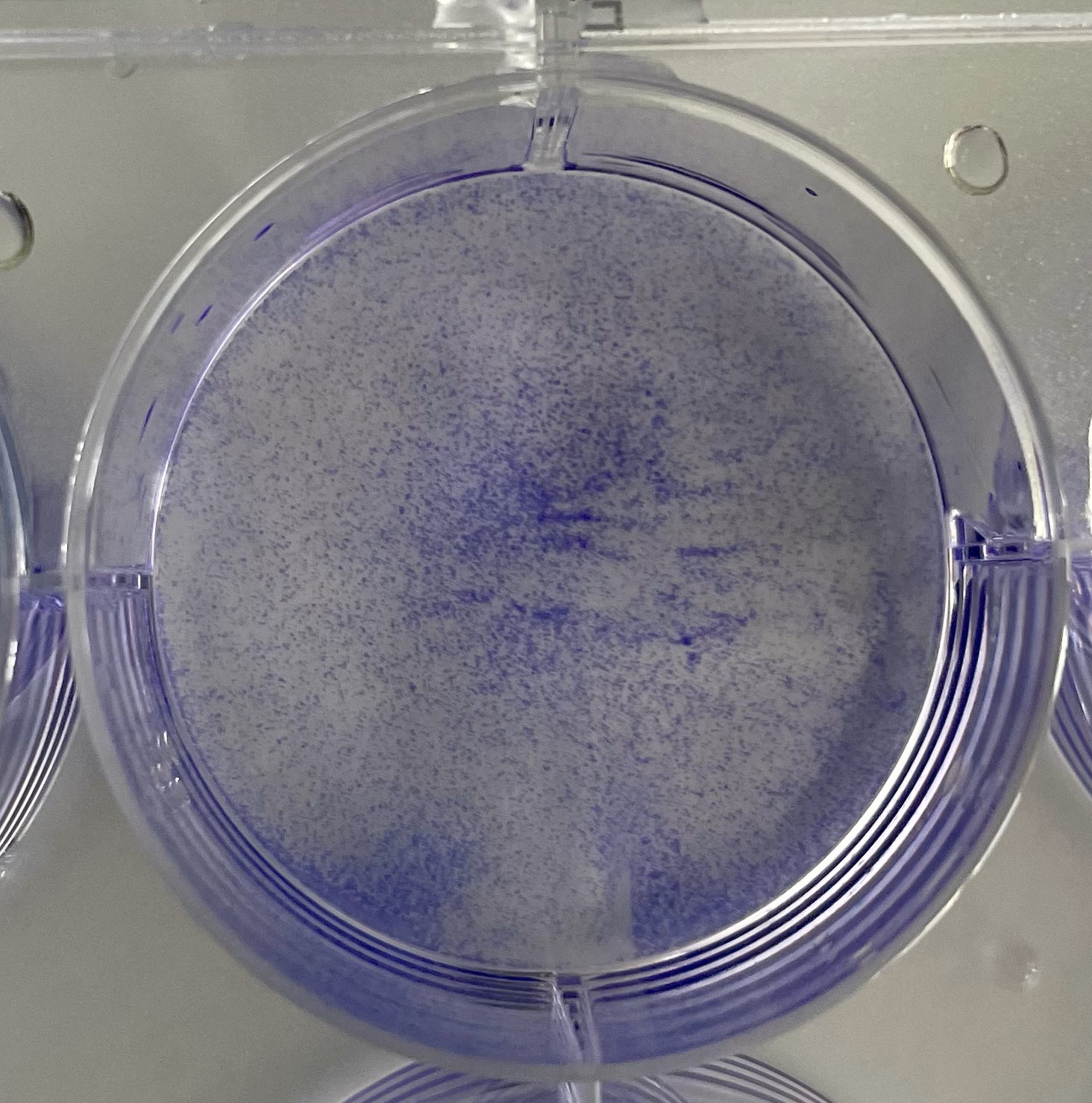

Supplement: Supplementary file 12 — EV Figure Source Data [file 44318_2025_381_MOESM12_ESM.zip › 44318_2025_381_MOESM12_ESM/Figure EV2/EV2G/empty shCTRL siPPP1R15A.jpg]

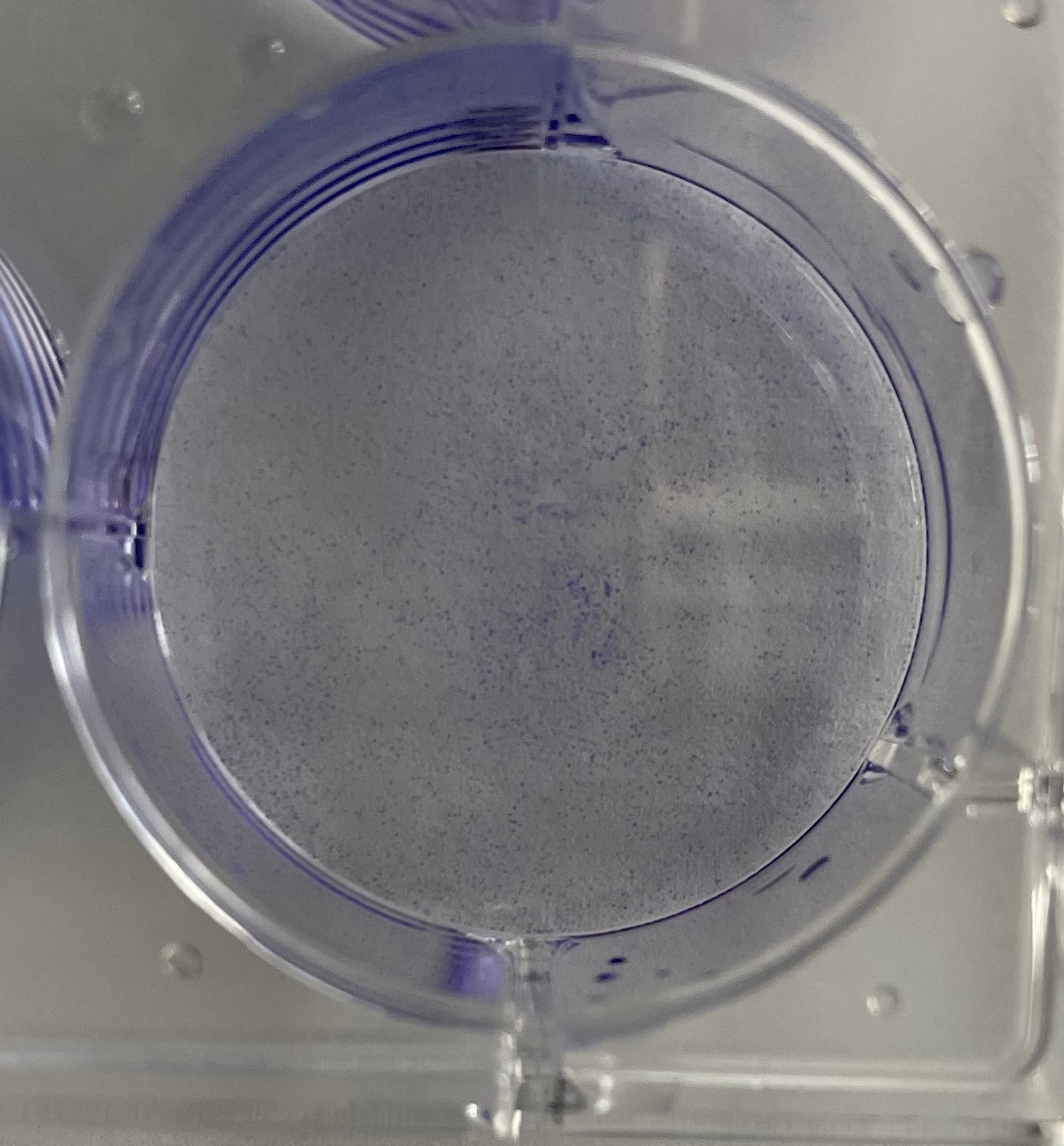

Supplement: Supplementary file 12 — EV Figure Source Data [file 44318_2025_381_MOESM12_ESM.zip › 44318_2025_381_MOESM12_ESM/Figure EV2/EV2G/empty shEIF2B1 siCTRL.jpg]

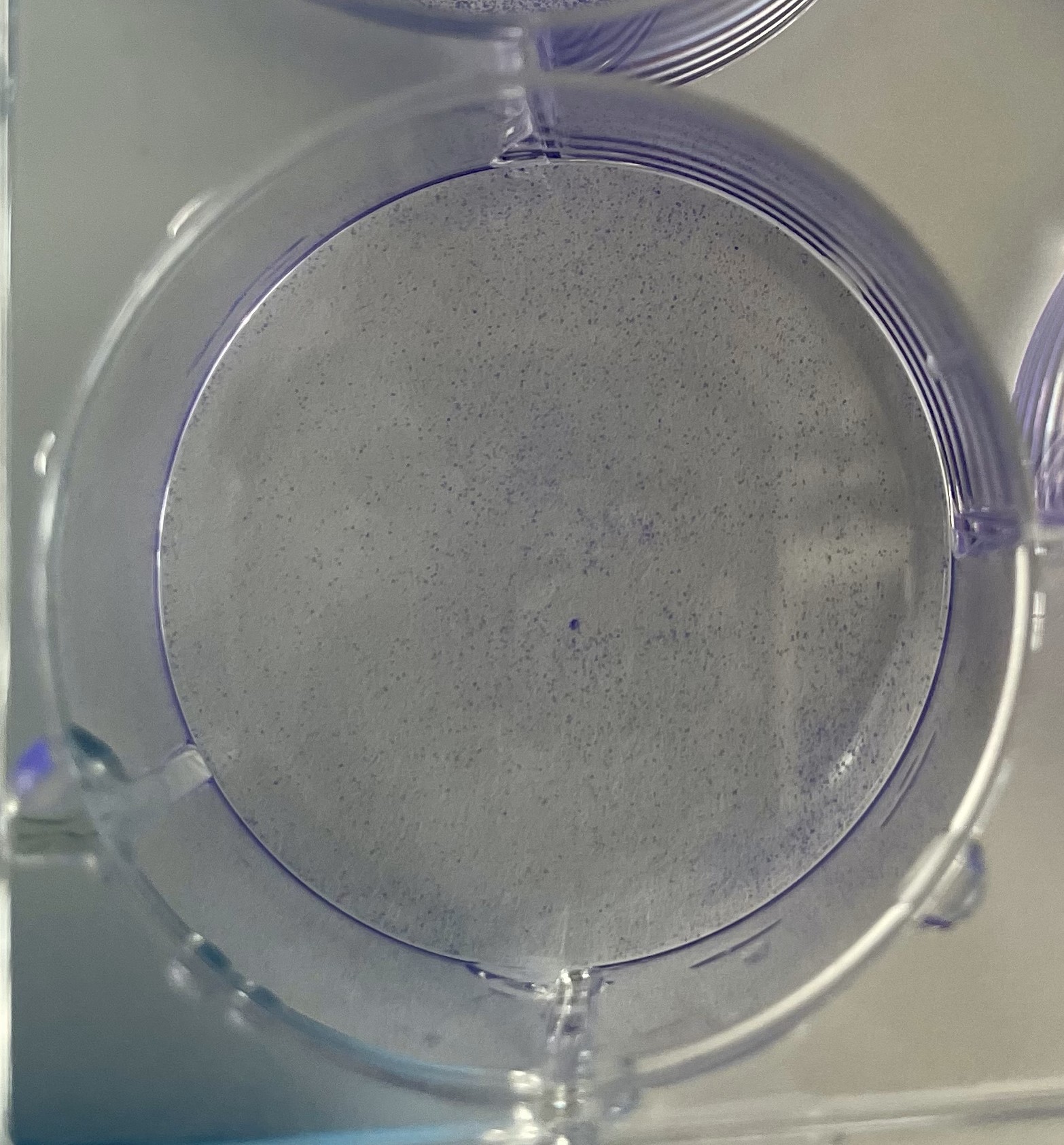

Supplement: Supplementary file 12 — EV Figure Source Data [file 44318_2025_381_MOESM12_ESM.zip › 44318_2025_381_MOESM12_ESM/Figure EV2/EV2G/empty shEIF2B1 siPPP1R15A.jpg]

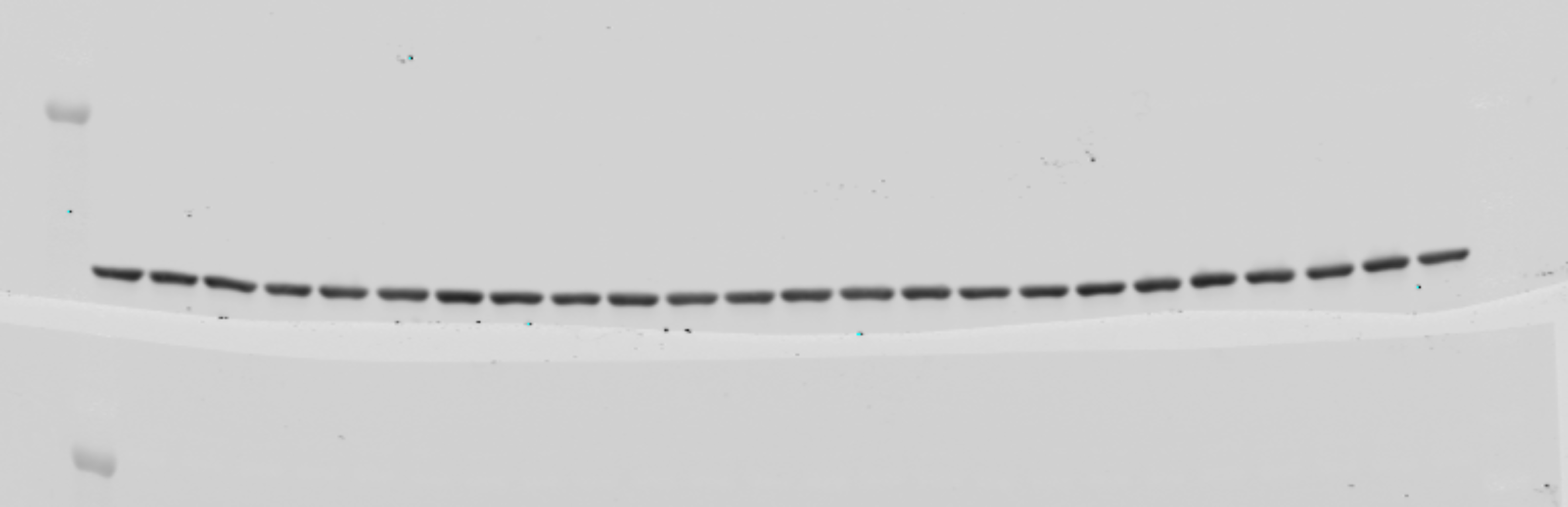

Supplement: Supplementary file 12 — EV Figure Source Data [file 44318_2025_381_MOESM12_ESM.zip › 44318_2025_381_MOESM12_ESM/Figure EV4/EV4A/single WBs/0017449_03_Actin.tif]

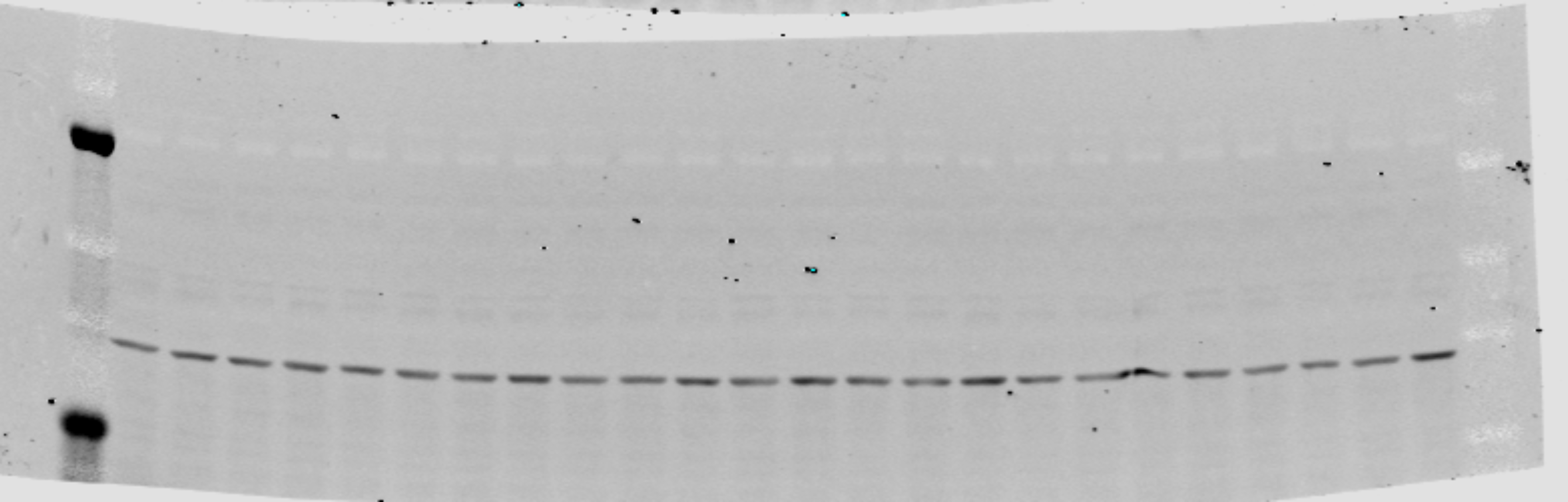

Supplement: Supplementary file 12 — EV Figure Source Data [file 44318_2025_381_MOESM12_ESM.zip › 44318_2025_381_MOESM12_ESM/Figure EV4/EV4A/single WBs/0017449_04_GAPDH.tif]

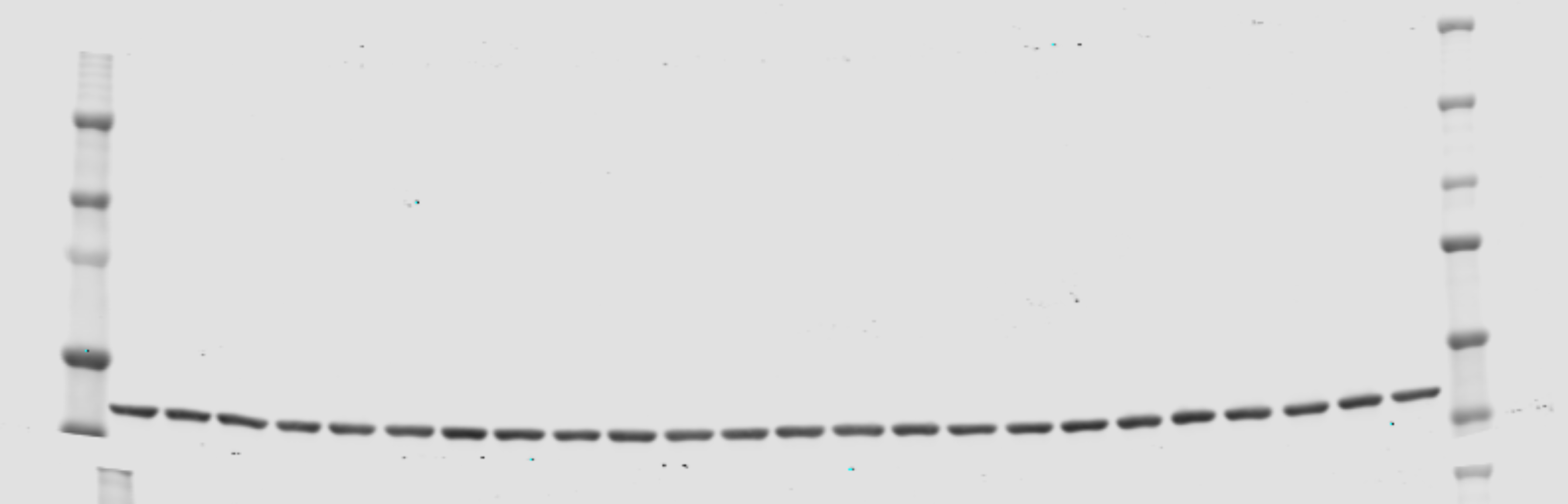

Supplement: Supplementary file 12 — EV Figure Source Data [file 44318_2025_381_MOESM12_ESM.zip › 44318_2025_381_MOESM12_ESM/Figure EV4/EV4A/single WBs/0017449_05_Actin.tif]

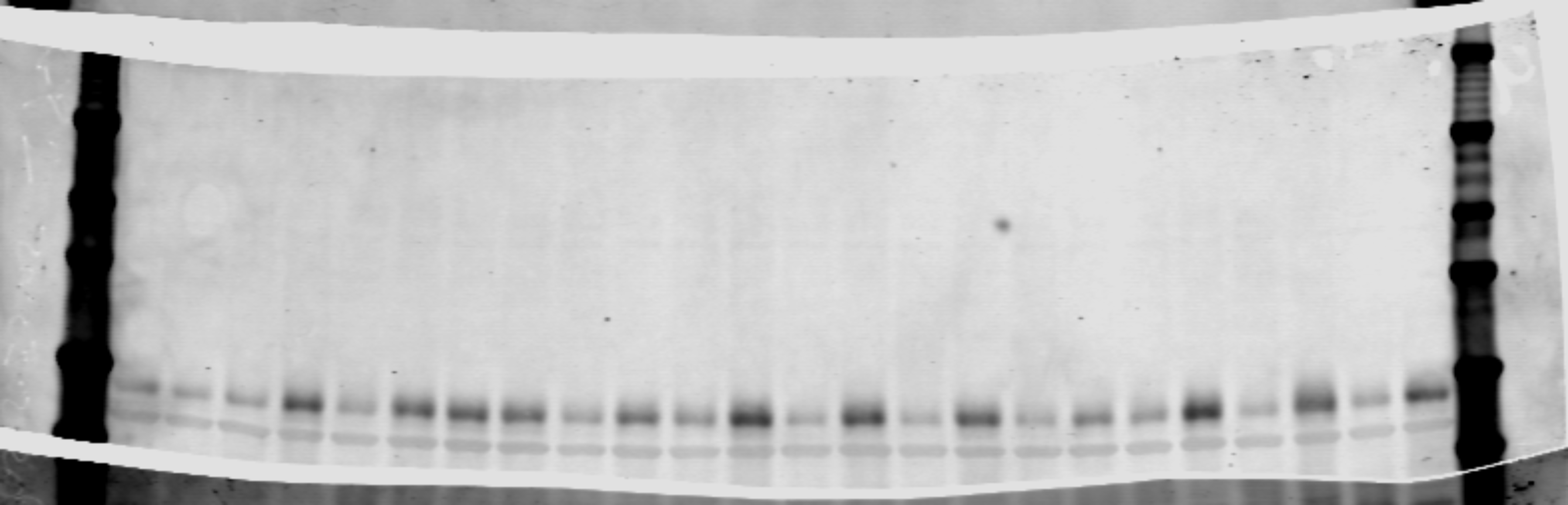

Supplement: Supplementary file 12 — EV Figure Source Data [file 44318_2025_381_MOESM12_ESM.zip › 44318_2025_381_MOESM12_ESM/Figure EV4/EV4A/single WBs/0017454_04_ATF4.tif]

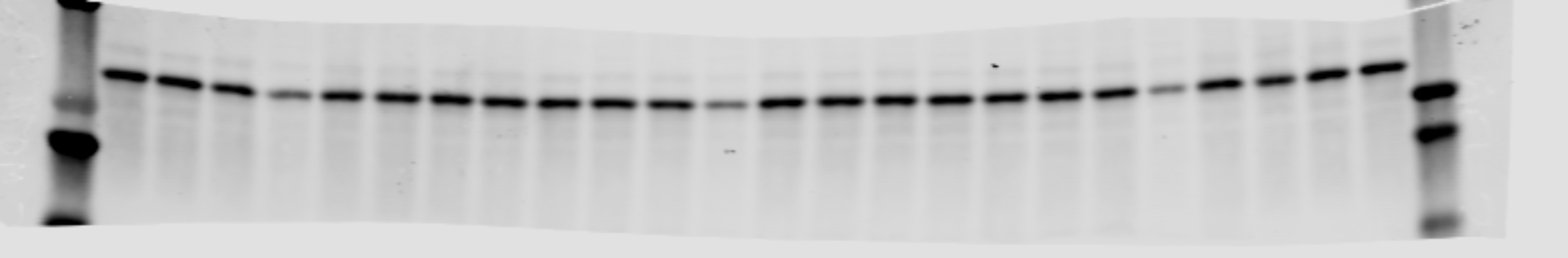

Supplement: Supplementary file 12 — EV Figure Source Data [file 44318_2025_381_MOESM12_ESM.zip › 44318_2025_381_MOESM12_ESM/Figure EV4/EV4A/single WBs/0017454_05_2Balpha.tif]

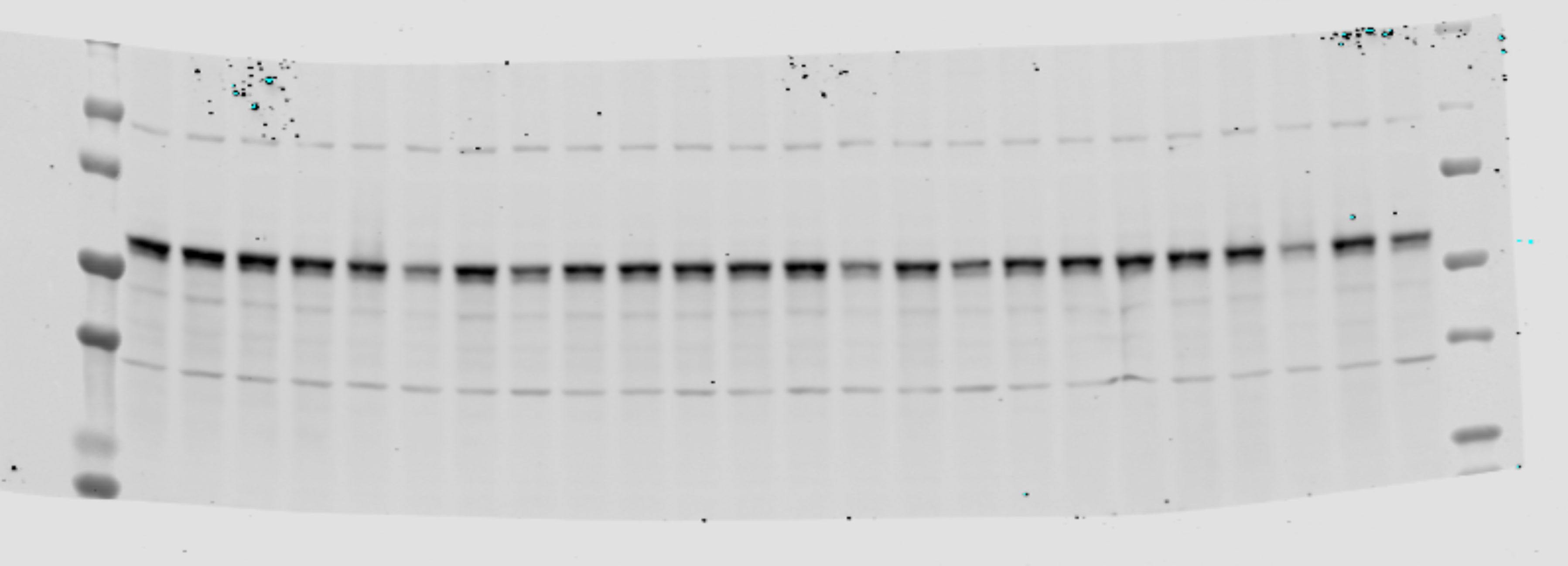

Supplement: Supplementary file 12 — EV Figure Source Data [file 44318_2025_381_MOESM12_ESM.zip › 44318_2025_381_MOESM12_ESM/Figure EV4/EV4A/single WBs/0017454_06_2Bdelta.tif]

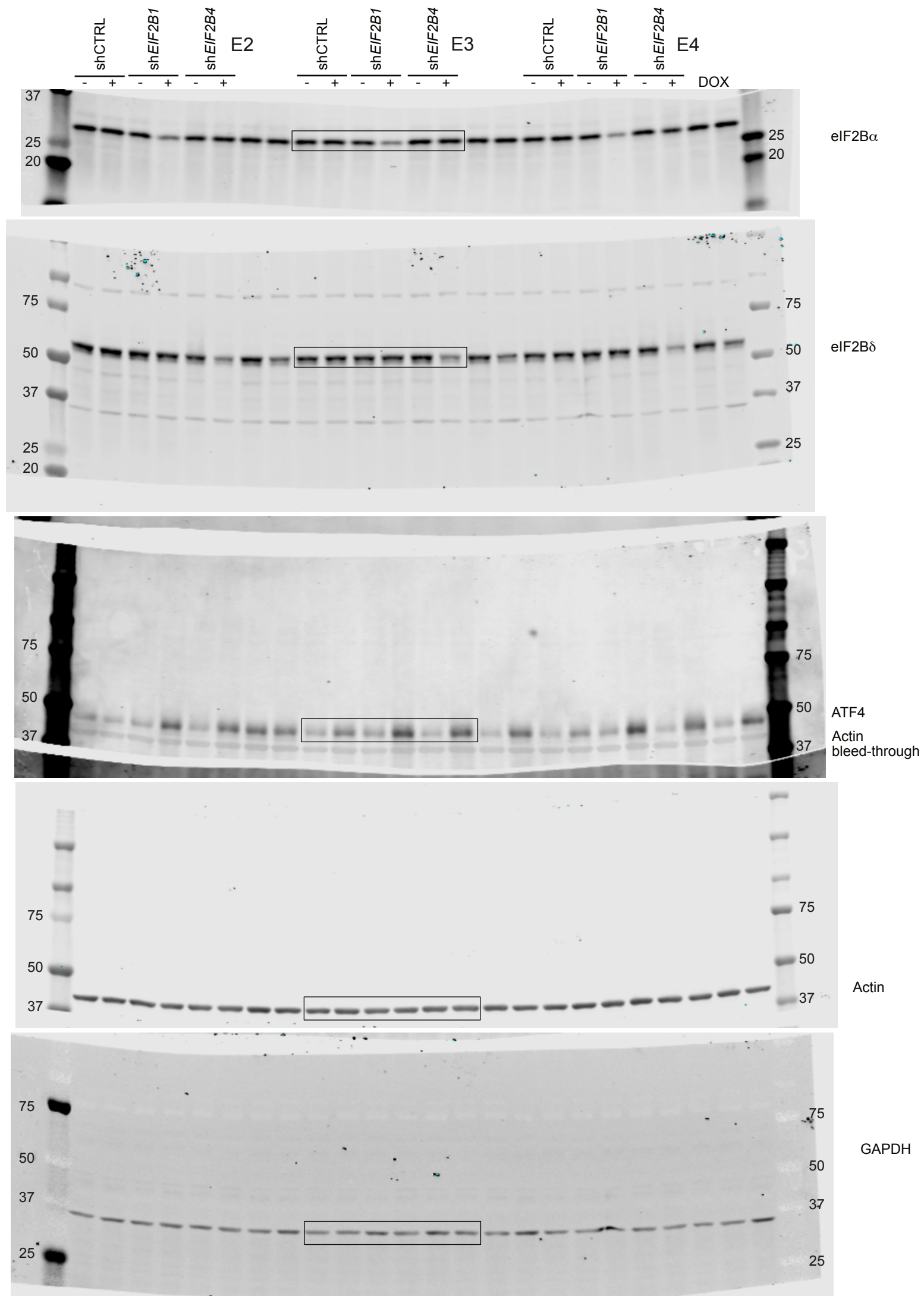

Supplement: Supplementary file 12 — EV Figure Source Data [file 44318_2025_381_MOESM12_ESM.zip › 44318_2025_381_MOESM12_ESM/Figure EV4/EV4A/single WBs/EV4A_Uncropped_blots.pdf]

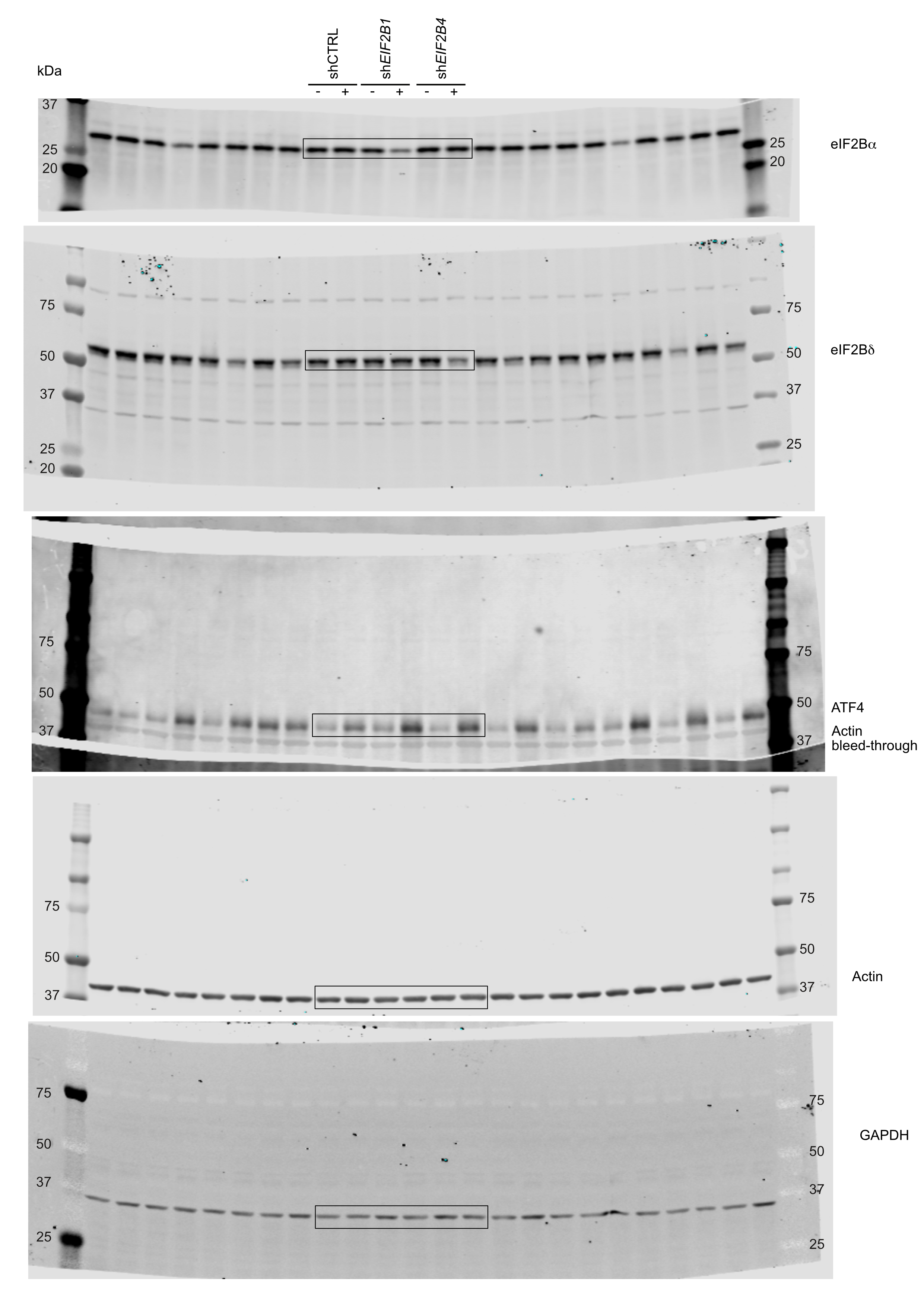

Supplement: Supplementary file 12 — EV Figure Source Data [file 44318_2025_381_MOESM12_ESM.zip › 44318_2025_381_MOESM12_ESM/Figure EV4/EV4A/western_EV4A.tiff]

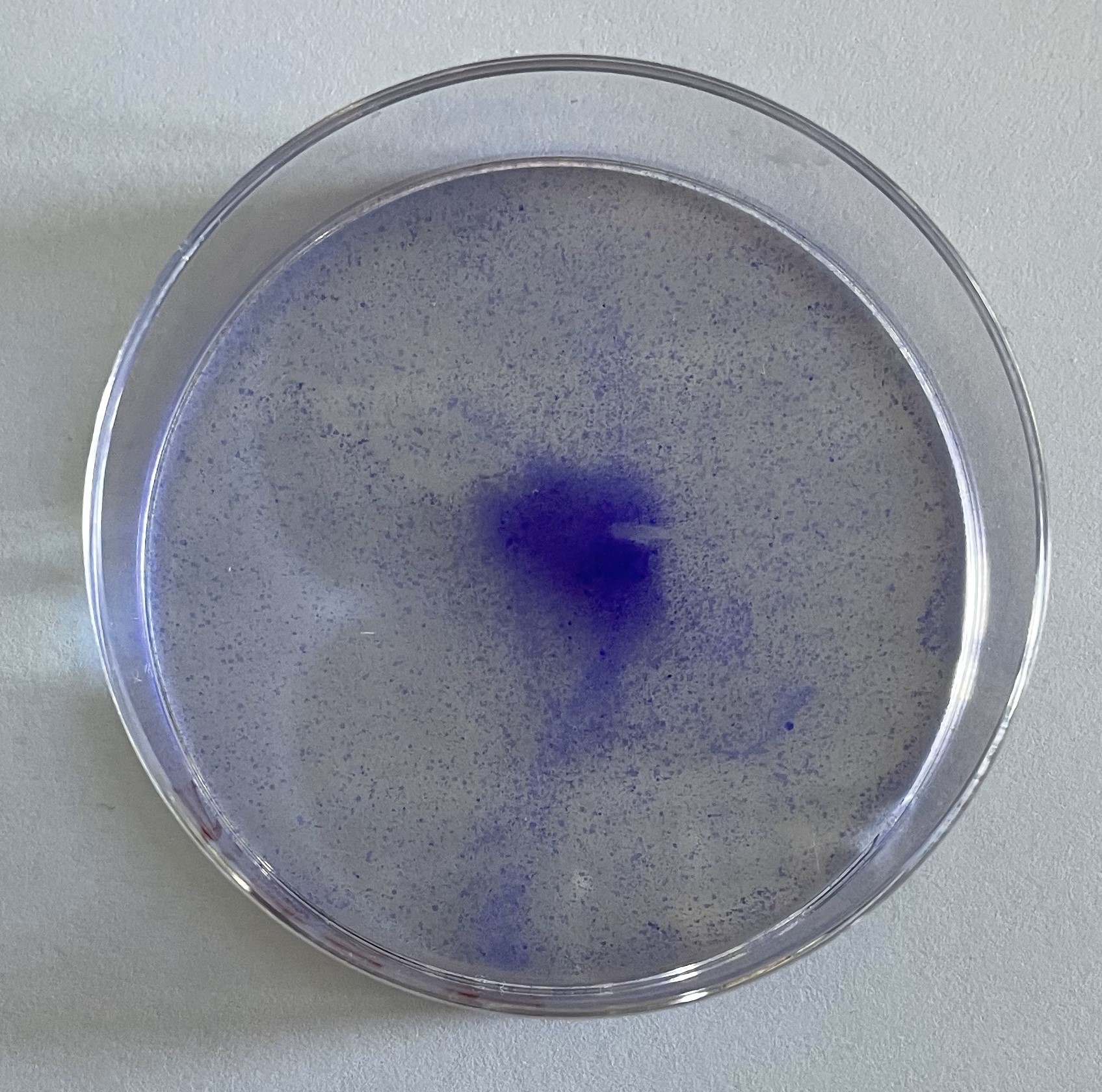

Supplement: Supplementary file 12 — EV Figure Source Data [file 44318_2025_381_MOESM12_ESM.zip › 44318_2025_381_MOESM12_ESM/Figure EV4/EV4B/shEIF2B1 +DOX.jpg]

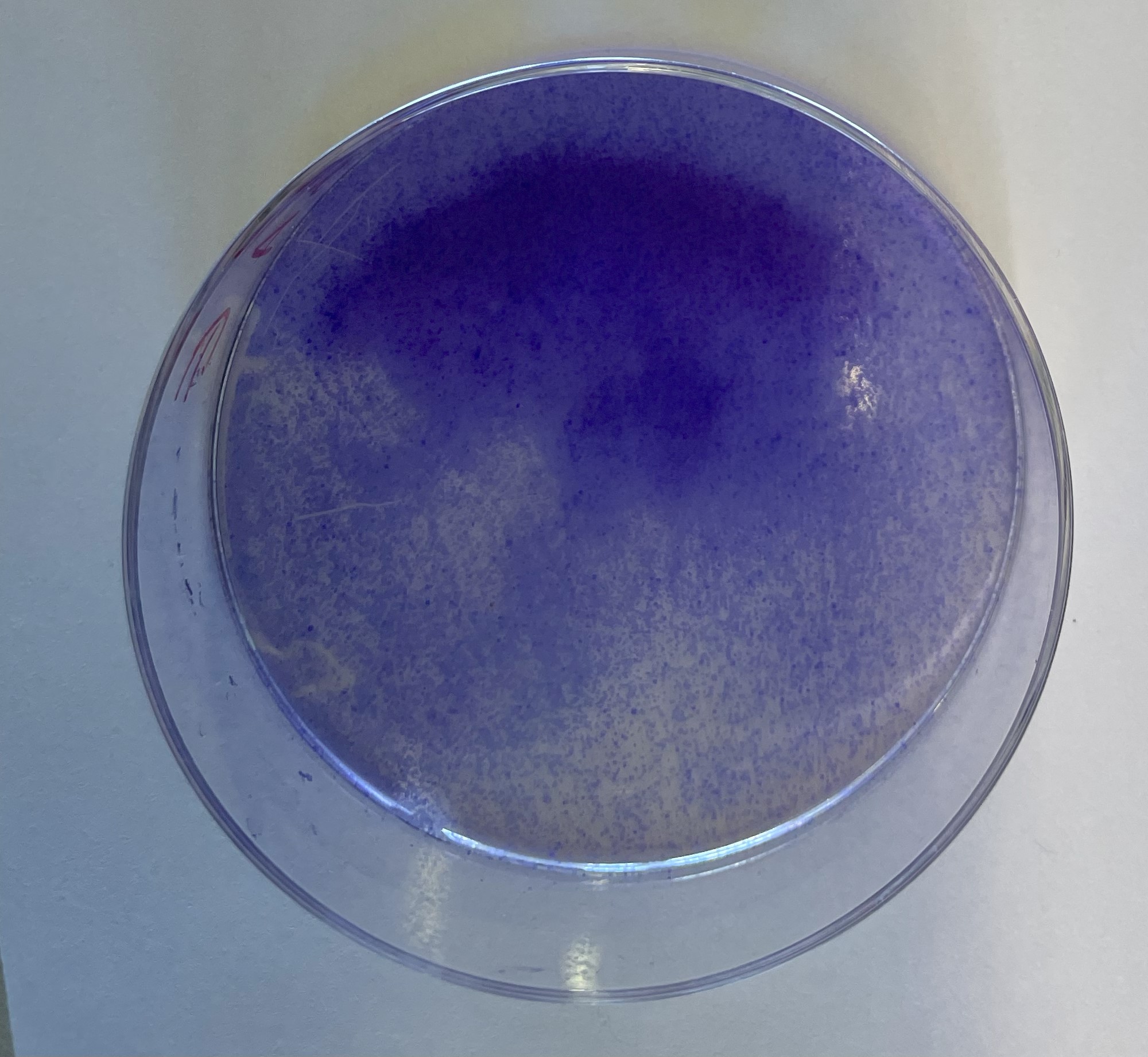

Supplement: Supplementary file 12 — EV Figure Source Data [file 44318_2025_381_MOESM12_ESM.zip › 44318_2025_381_MOESM12_ESM/Figure EV4/EV4B/shEIF2B1 -DOX.jpg]

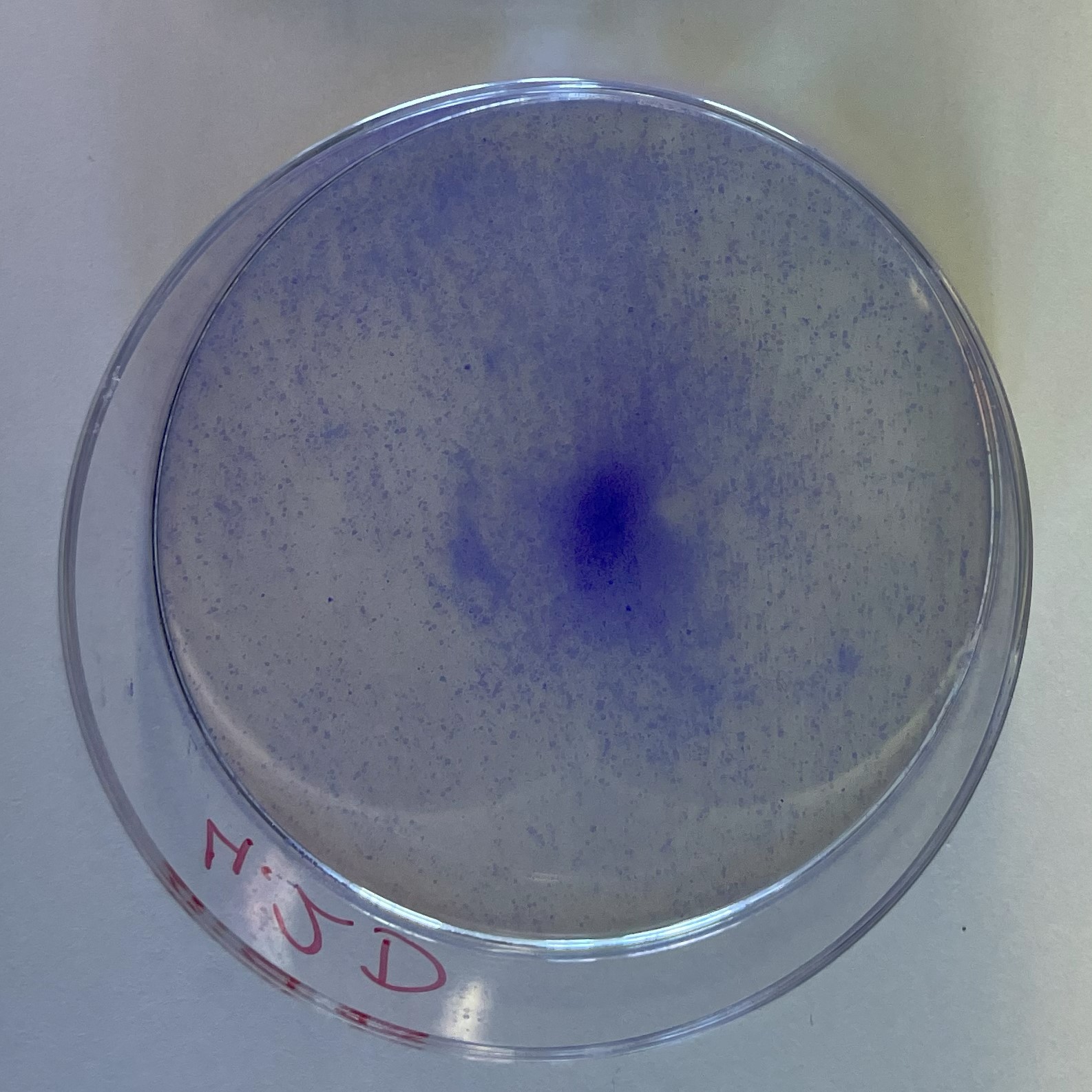

Supplement: Supplementary file 12 — EV Figure Source Data [file 44318_2025_381_MOESM12_ESM.zip › 44318_2025_381_MOESM12_ESM/Figure EV4/EV4B/shEIF2B4 +DOX.jpg]

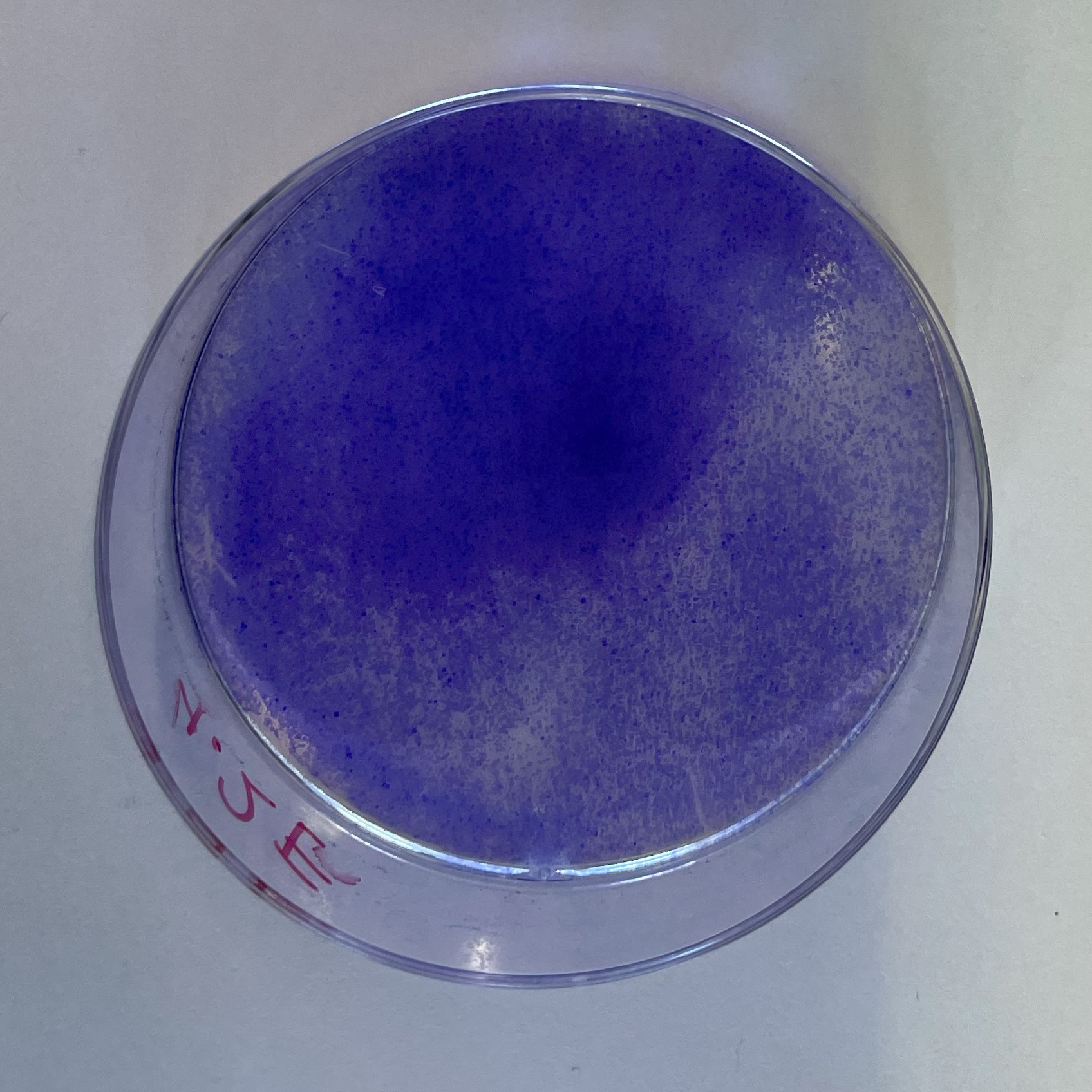

Supplement: Supplementary file 12 — EV Figure Source Data [file 44318_2025_381_MOESM12_ESM.zip › 44318_2025_381_MOESM12_ESM/Figure EV4/EV4B/shEIF2B4 -DOX.jpg]

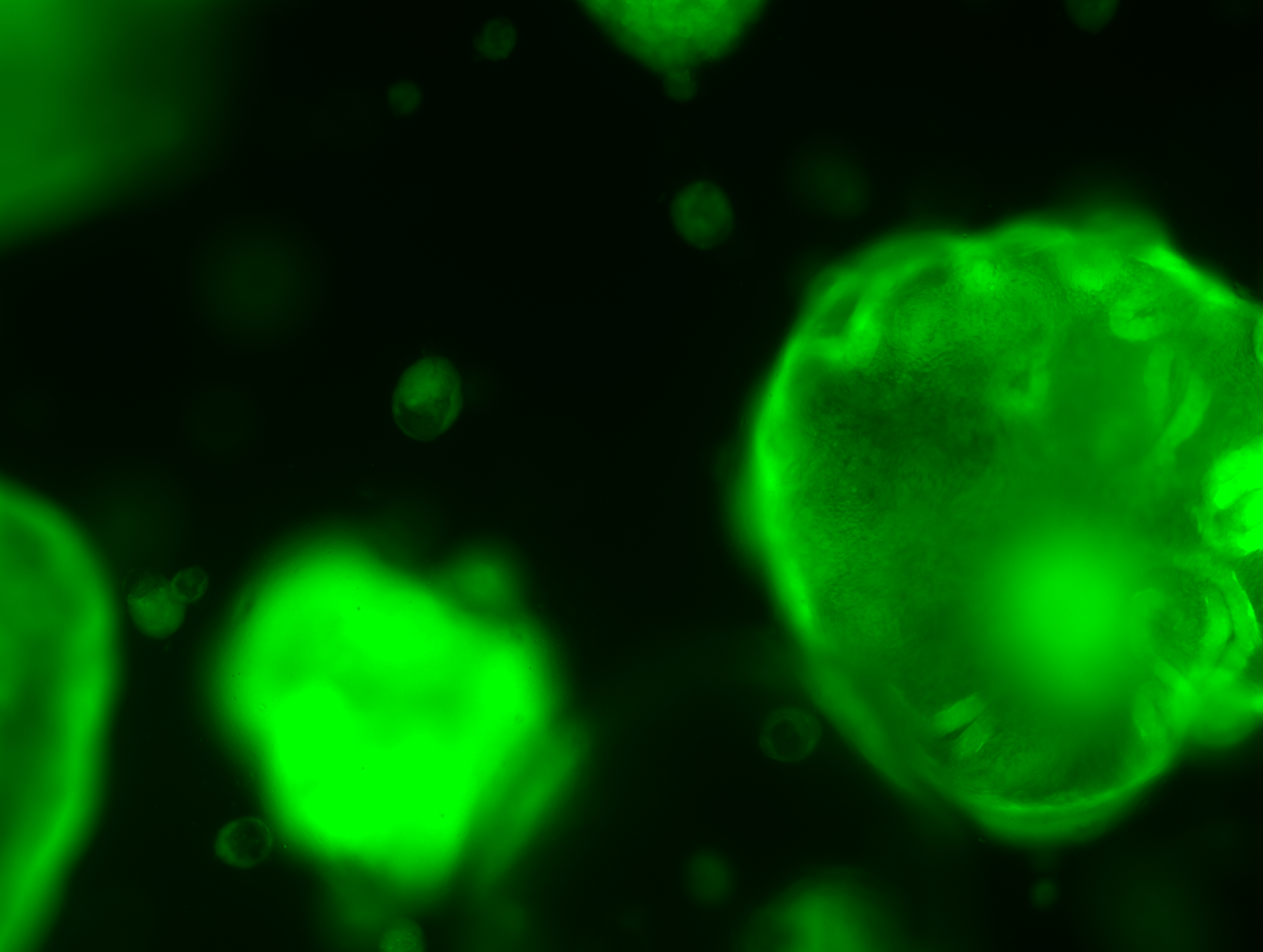

Supplement: Supplementary file 12 — EV Figure Source Data [file 44318_2025_381_MOESM12_ESM.zip › 44318_2025_381_MOESM12_ESM/Figure EV5/EV5A/AK_shCTRL.png]

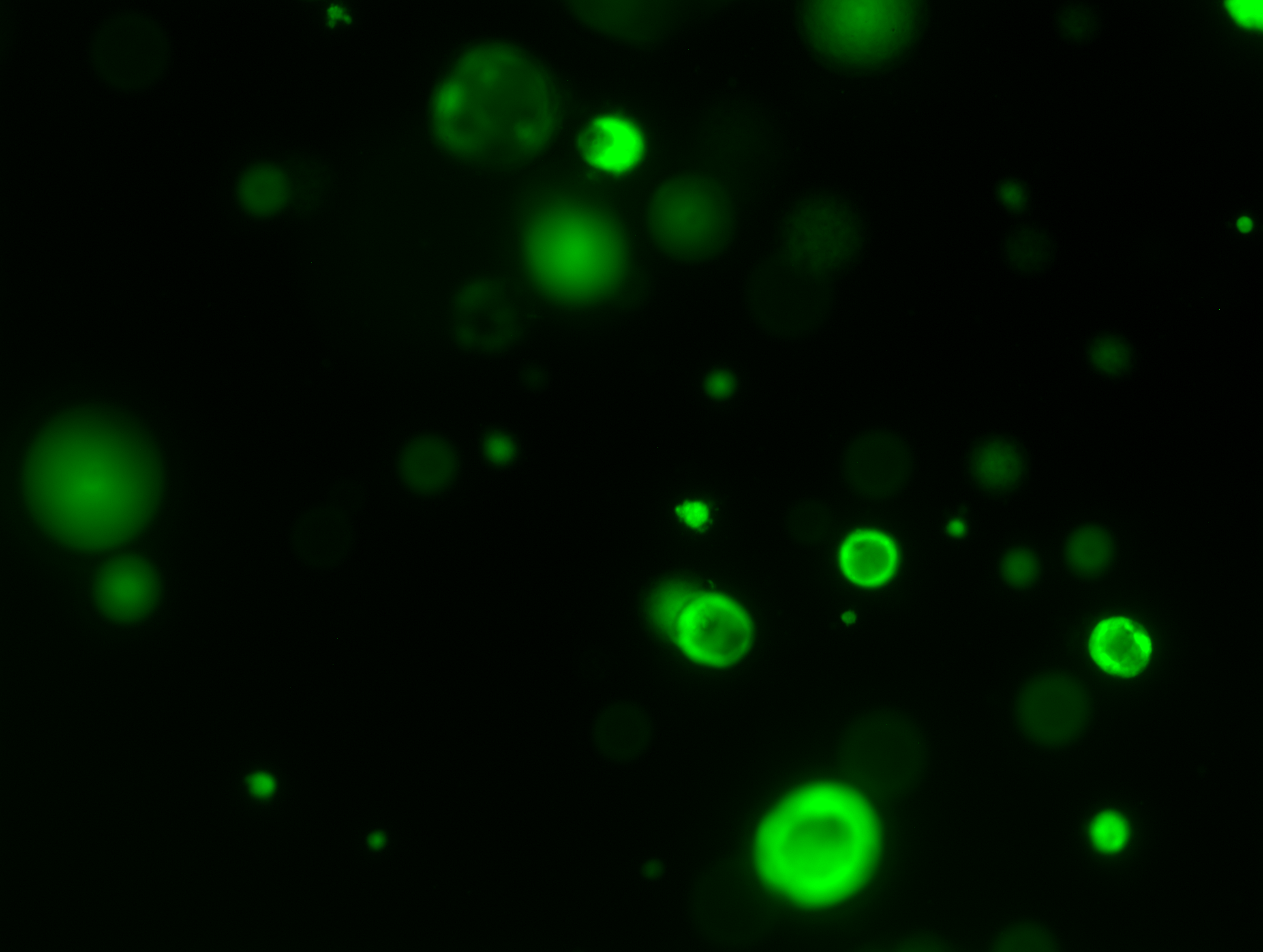

Supplement: Supplementary file 12 — EV Figure Source Data [file 44318_2025_381_MOESM12_ESM.zip › 44318_2025_381_MOESM12_ESM/Figure EV5/EV5A/AK_shEif2b1-2.png]

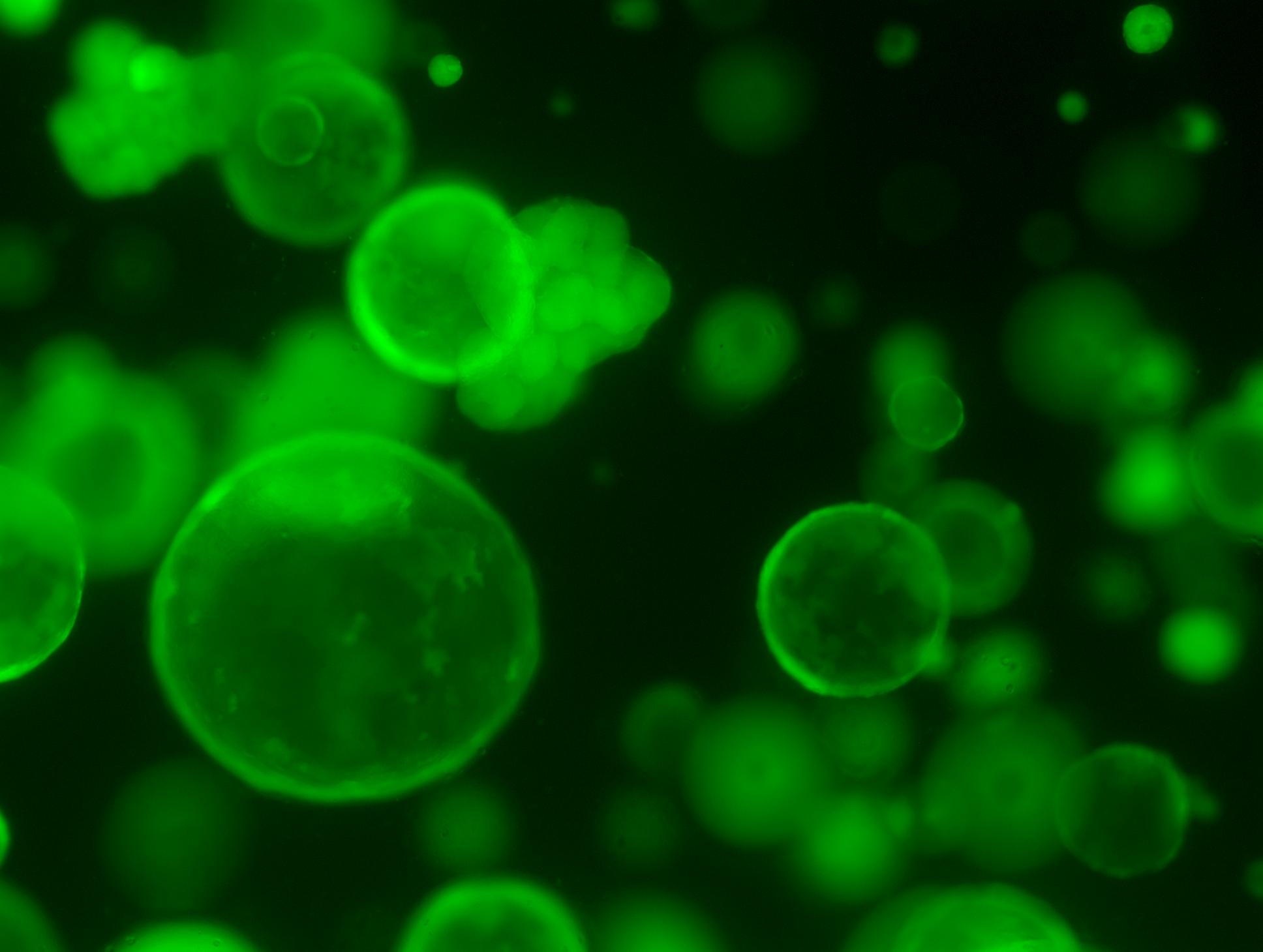

Supplement: Supplementary file 12 — EV Figure Source Data [file 44318_2025_381_MOESM12_ESM.zip › 44318_2025_381_MOESM12_ESM/Figure EV5/EV5A/A_shCTRL.png]

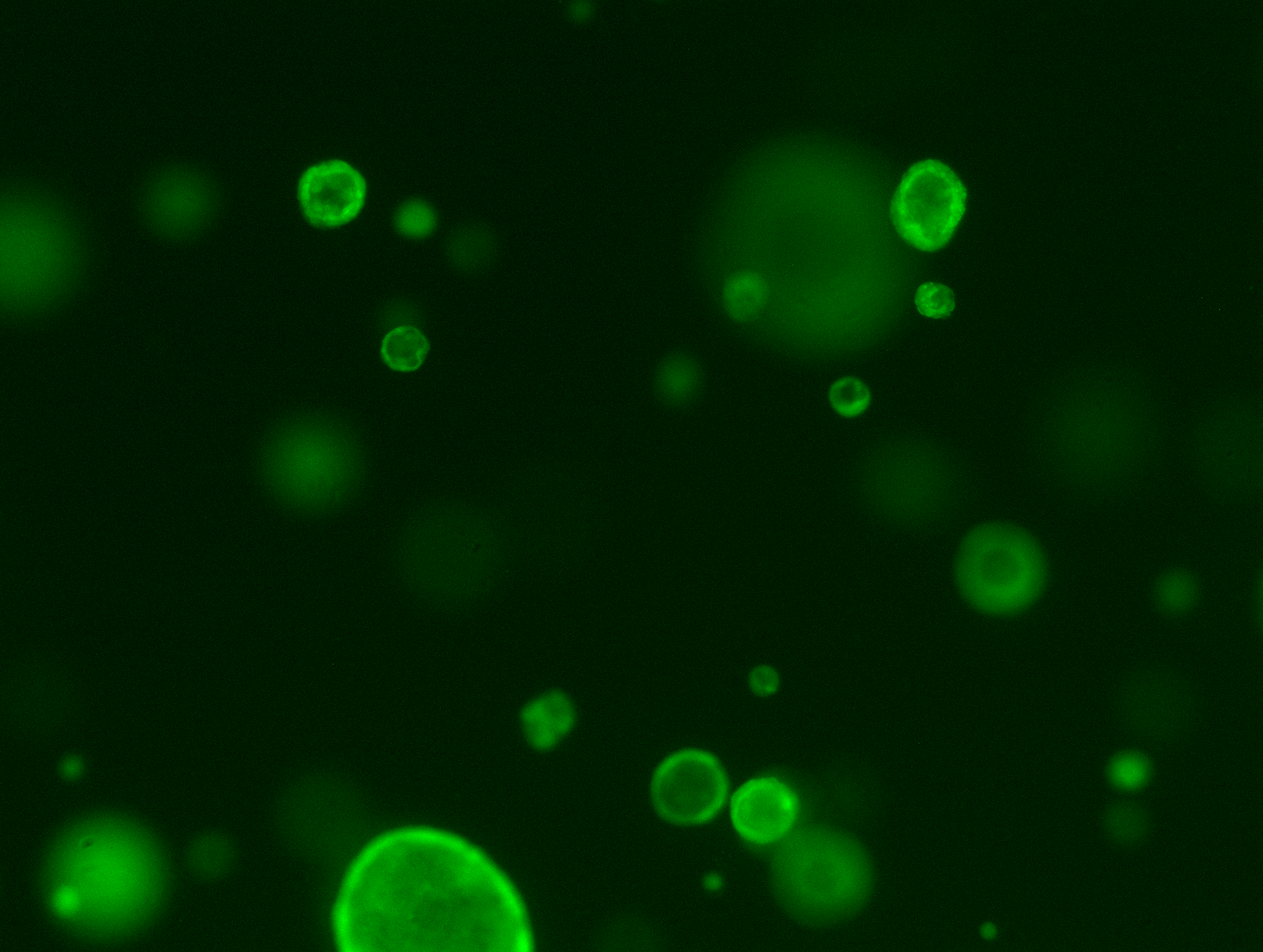

Supplement: Supplementary file 12 — EV Figure Source Data [file 44318_2025_381_MOESM12_ESM.zip › 44318_2025_381_MOESM12_ESM/Figure EV5/EV5A/A_shEif2b1-2.png]

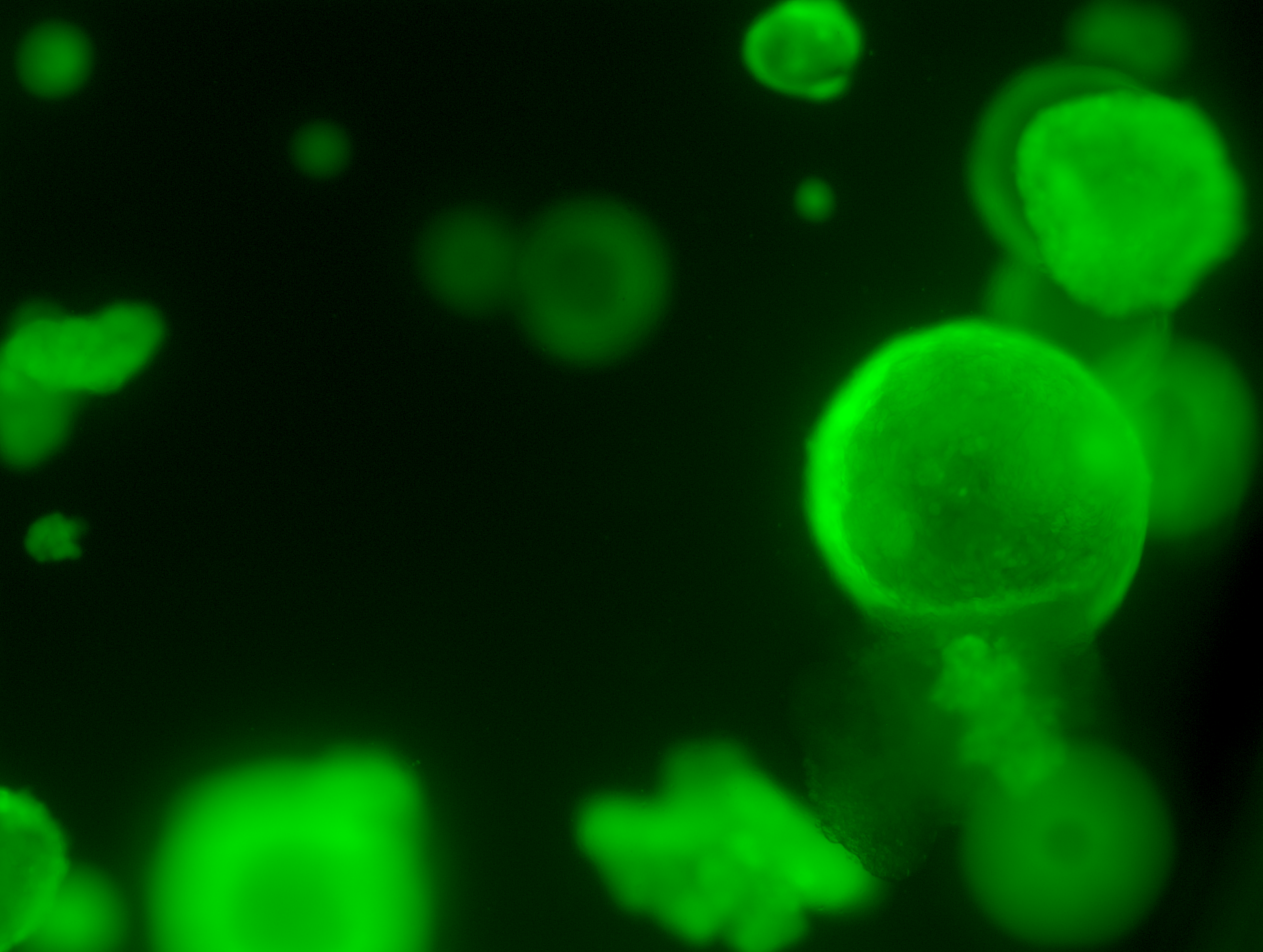

Supplement: Supplementary file 12 — EV Figure Source Data [file 44318_2025_381_MOESM12_ESM.zip › 44318_2025_381_MOESM12_ESM/Figure EV5/EV5A/LAKTP_shCTRL.png]

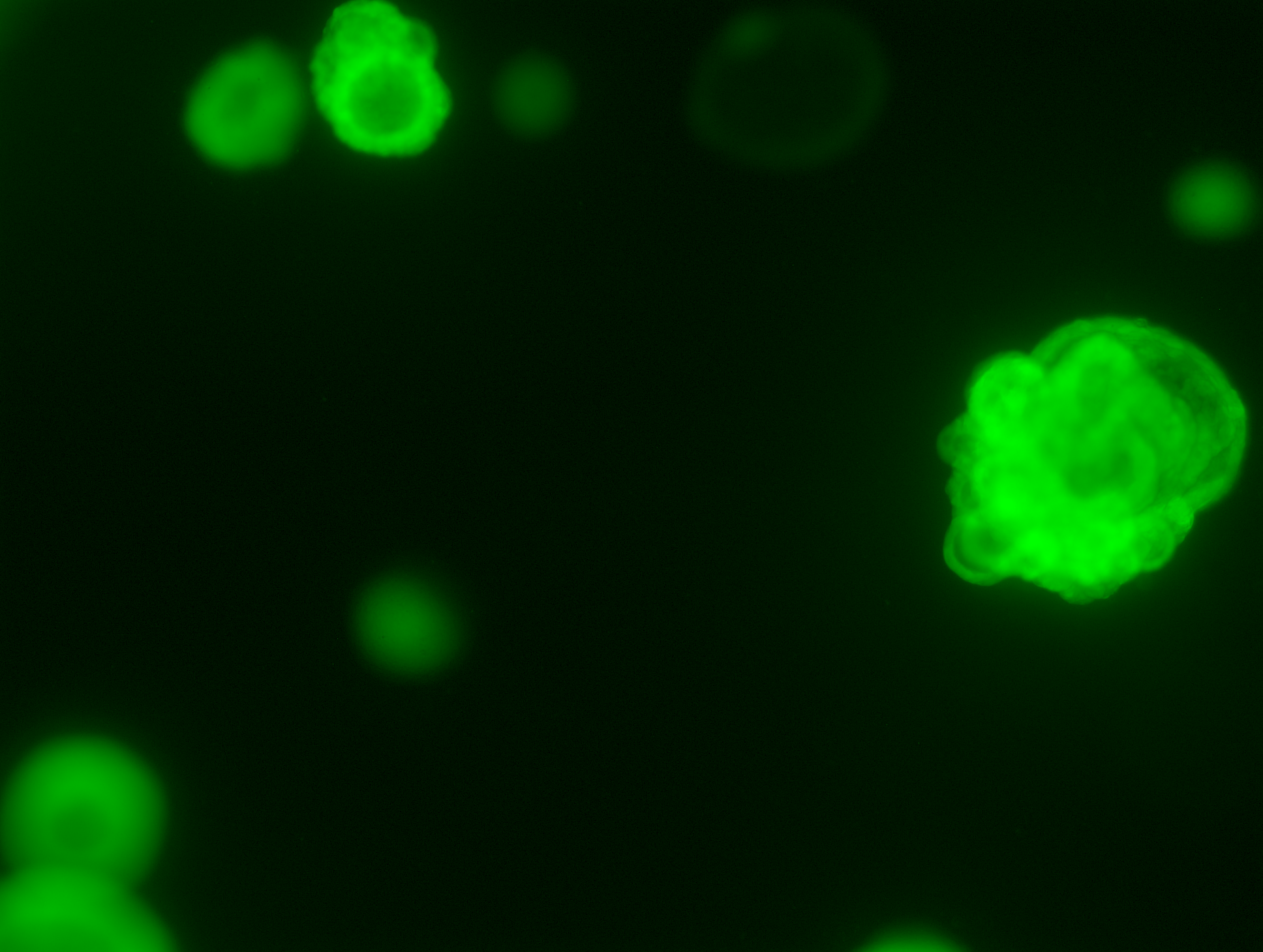

Supplement: Supplementary file 12 — EV Figure Source Data [file 44318_2025_381_MOESM12_ESM.zip › 44318_2025_381_MOESM12_ESM/Figure EV5/EV5A/LAKTP_shEif2b1-2.png]

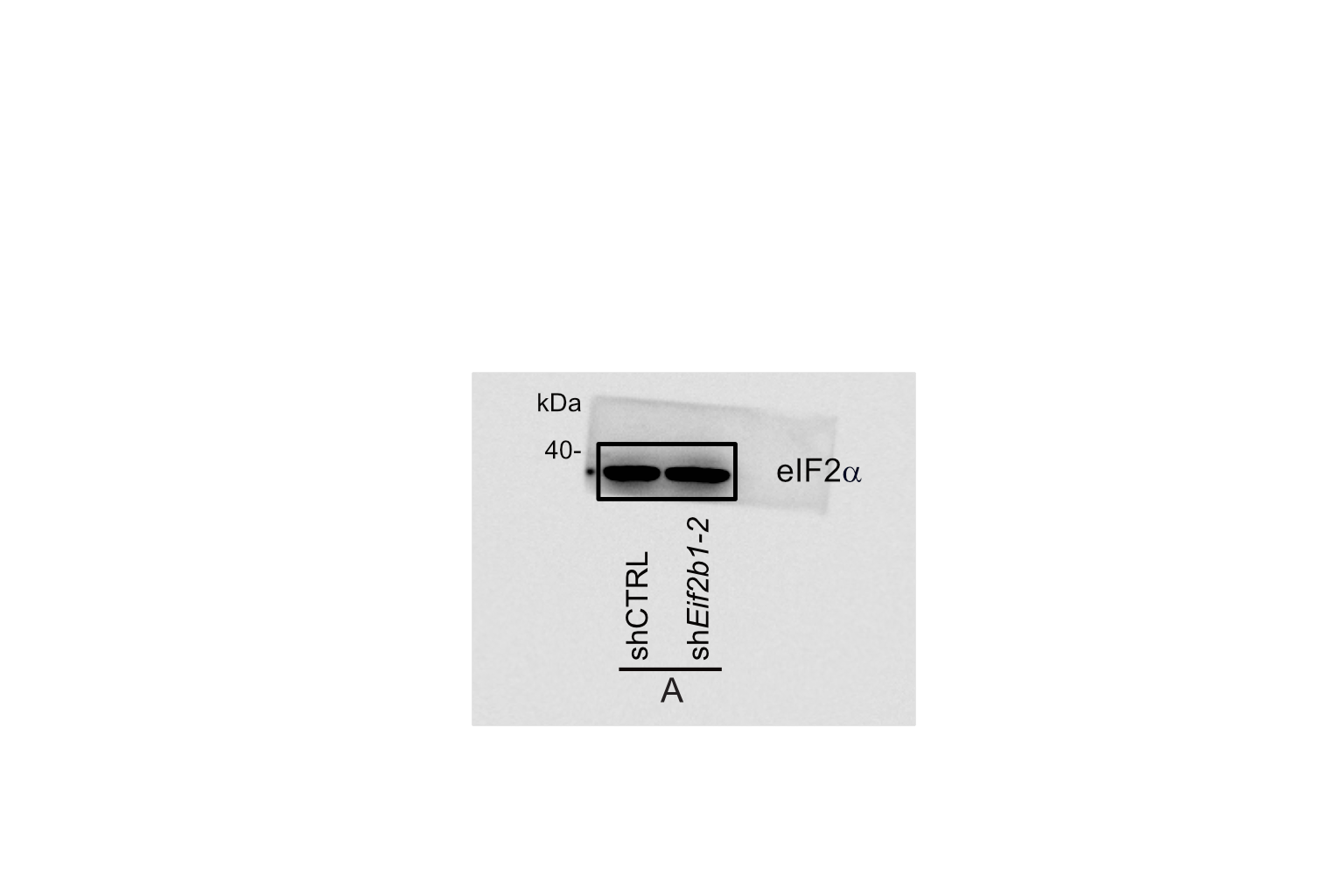

Supplement: Supplementary file 12 — EV Figure Source Data [file 44318_2025_381_MOESM12_ESM.zip › 44318_2025_381_MOESM12_ESM/Figure EV5/EV5C/A/western eIF2a_A.tif]

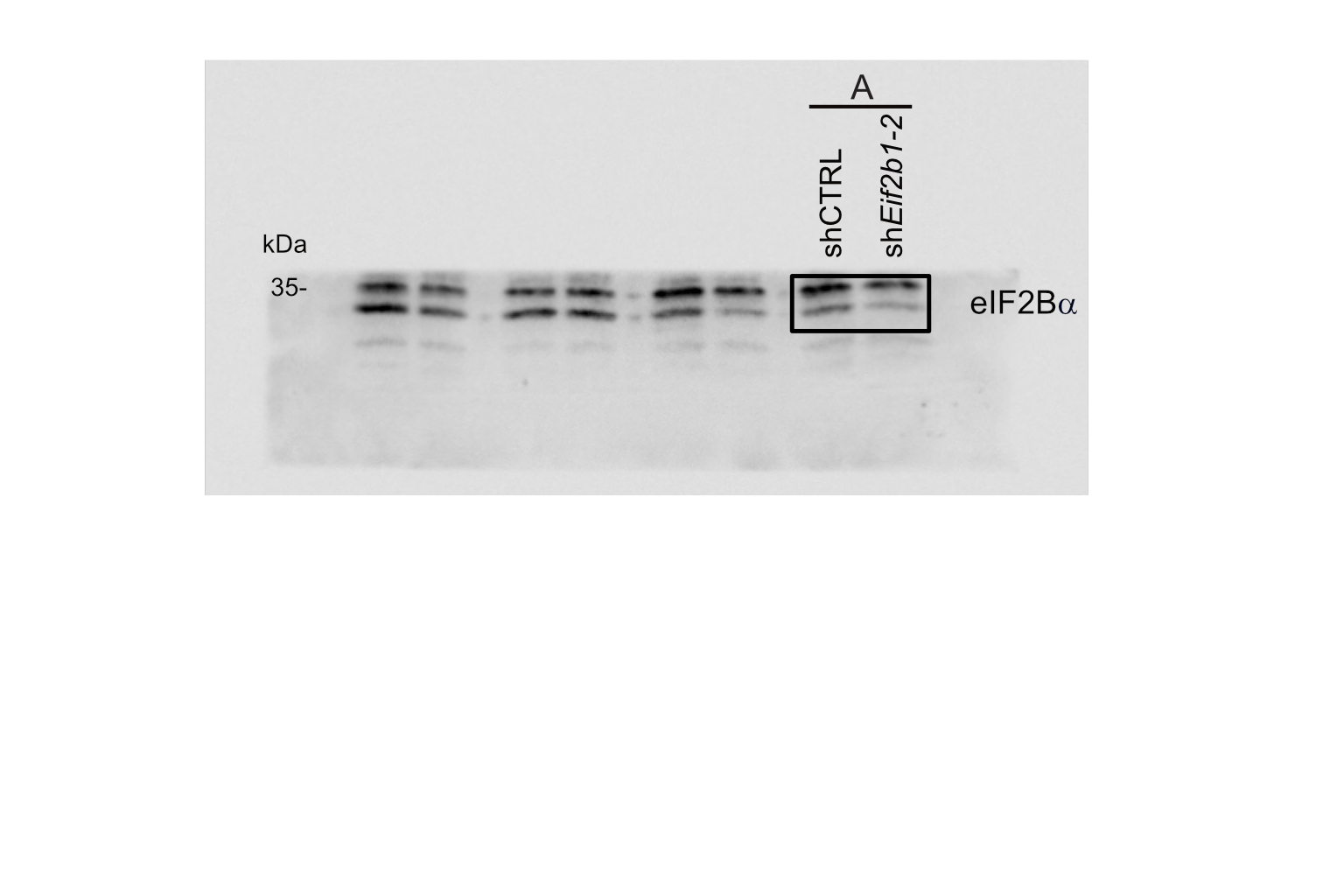

Supplement: Supplementary file 12 — EV Figure Source Data [file 44318_2025_381_MOESM12_ESM.zip › 44318_2025_381_MOESM12_ESM/Figure EV5/EV5C/A/western eIF2Ba_A.tif]

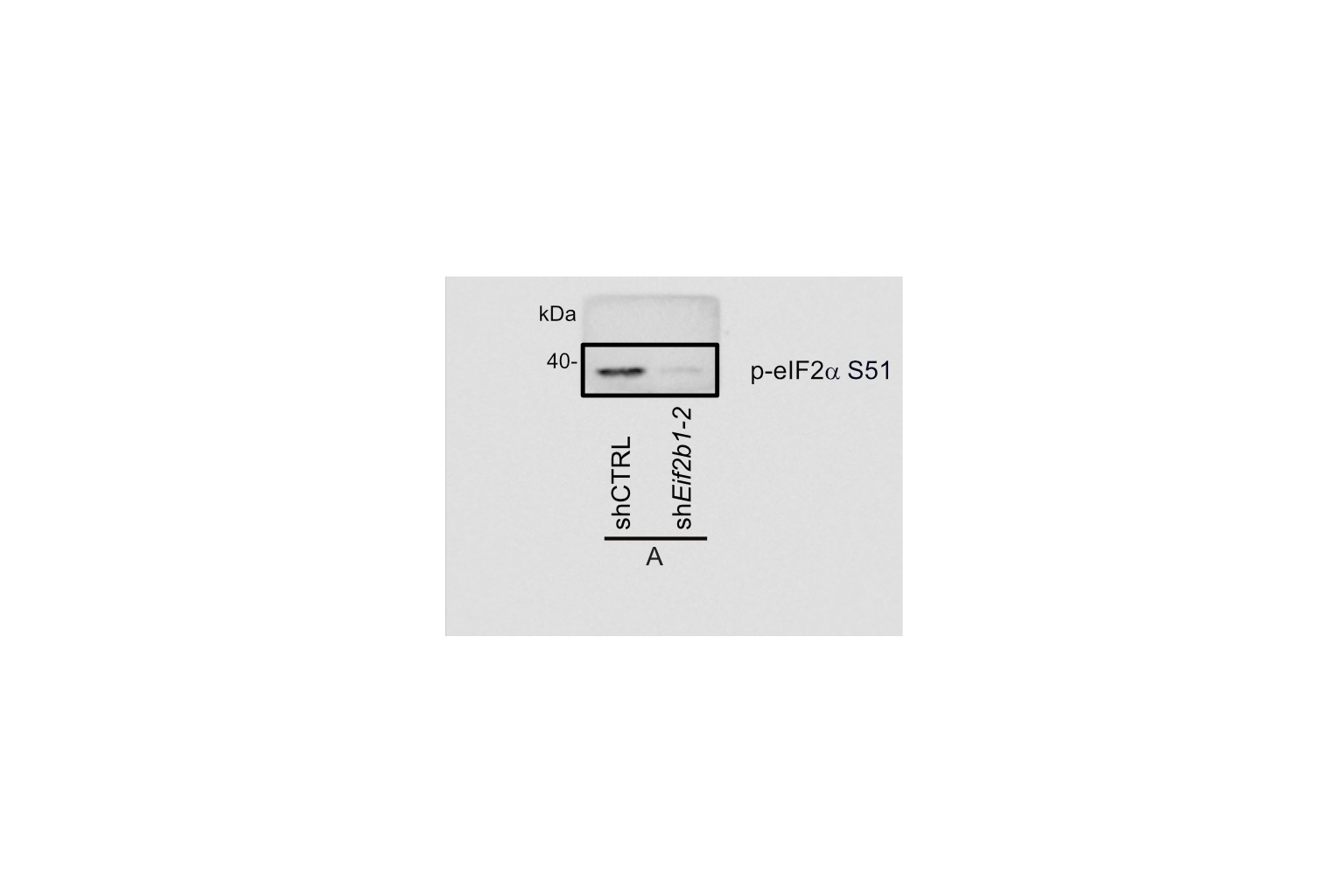

Supplement: Supplementary file 12 — EV Figure Source Data [file 44318_2025_381_MOESM12_ESM.zip › 44318_2025_381_MOESM12_ESM/Figure EV5/EV5C/A/western p-eIF2a S51_A.tif]

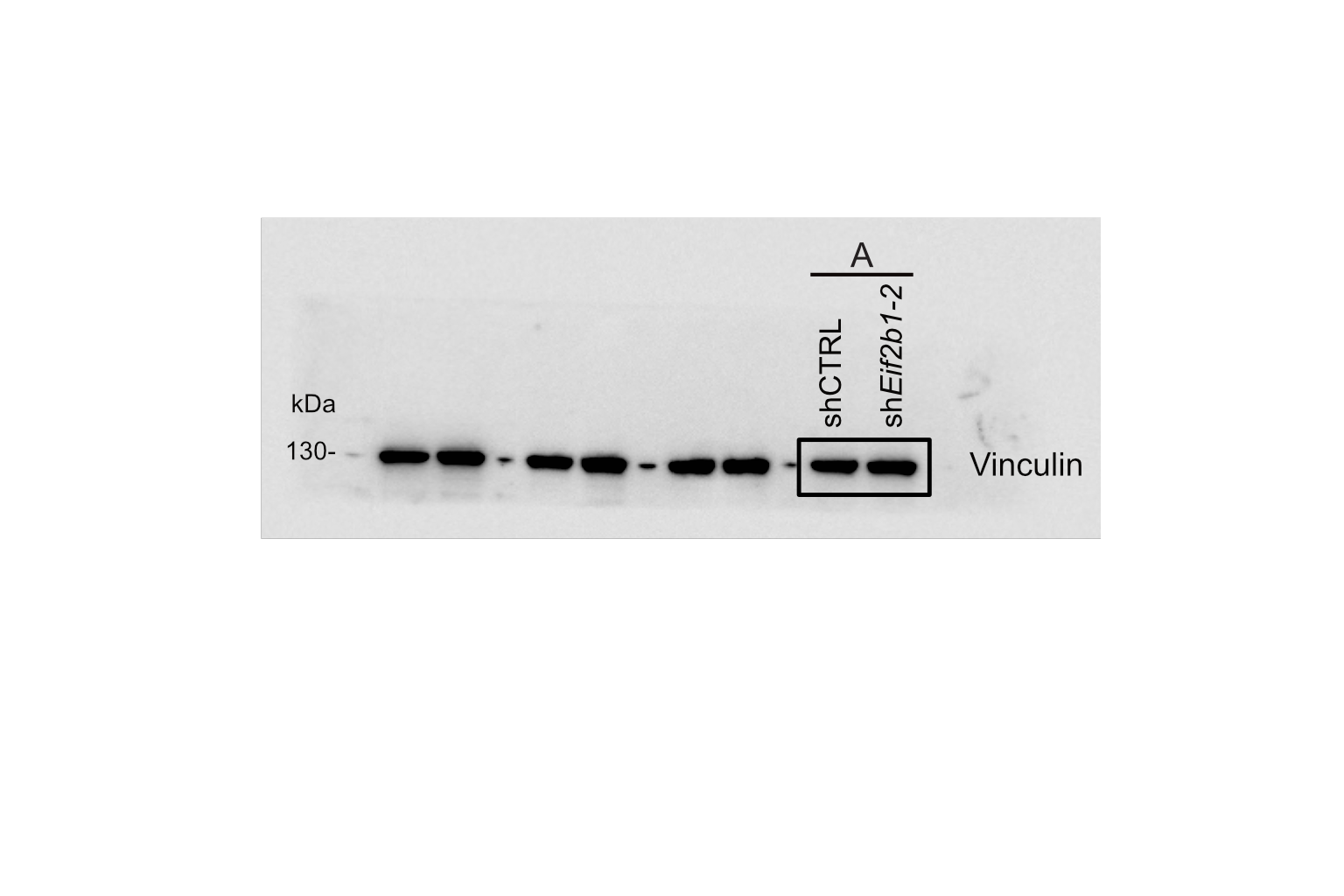

Supplement: Supplementary file 12 — EV Figure Source Data [file 44318_2025_381_MOESM12_ESM.zip › 44318_2025_381_MOESM12_ESM/Figure EV5/EV5C/A/western vinculin_A.tif]

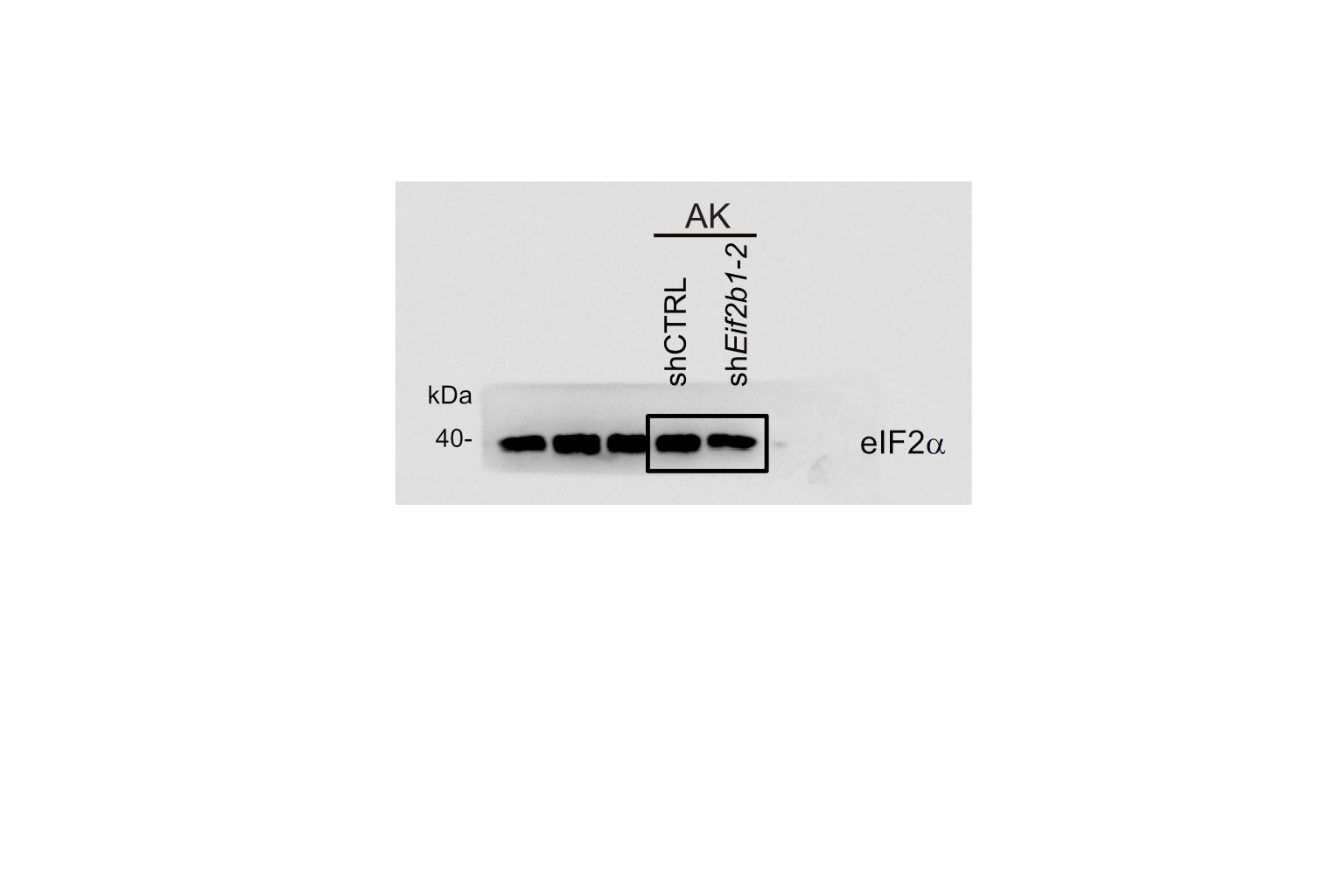

Supplement: Supplementary file 12 — EV Figure Source Data [file 44318_2025_381_MOESM12_ESM.zip › 44318_2025_381_MOESM12_ESM/Figure EV5/EV5C/AK/western eIF2a_AK.tif]

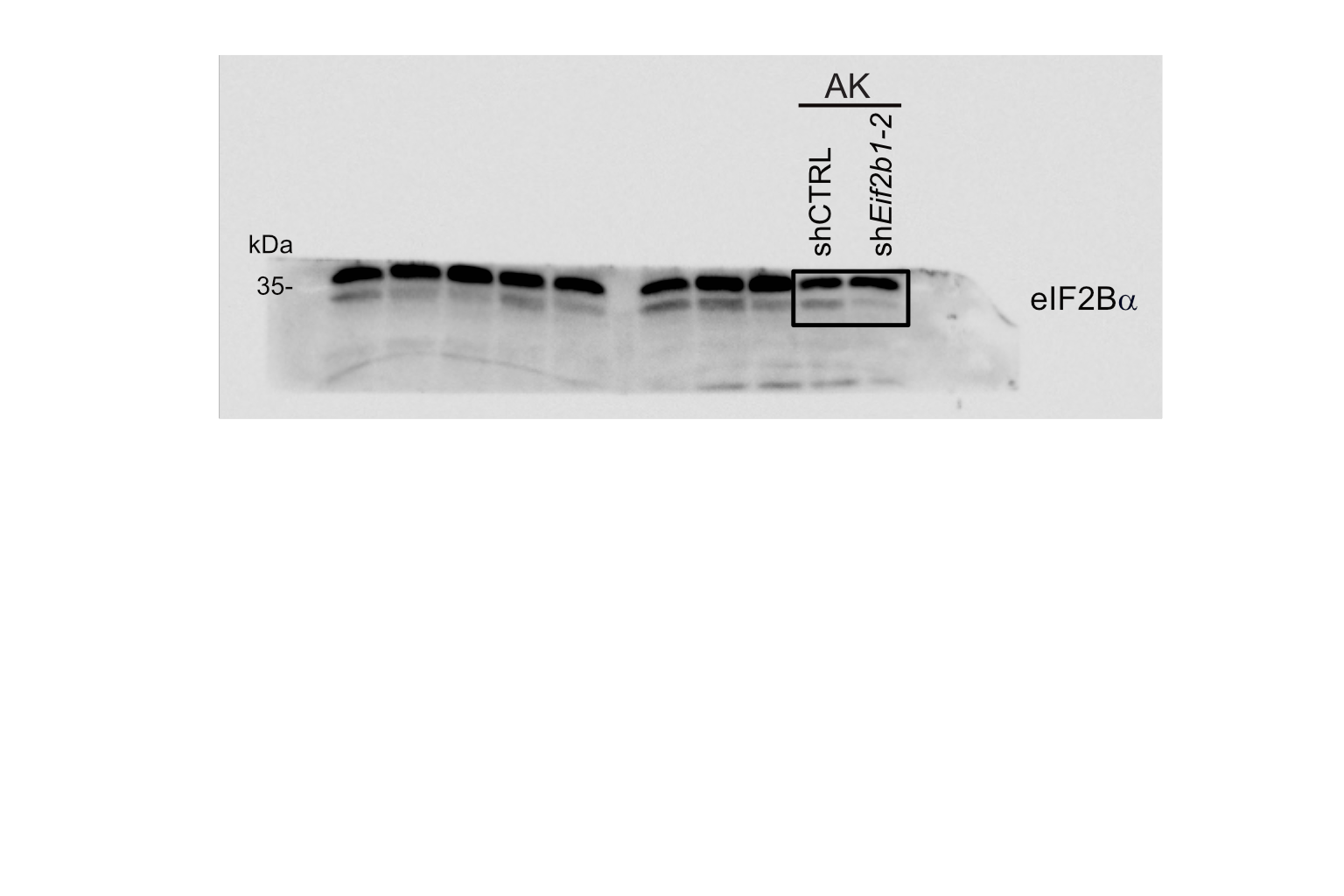

Supplement: Supplementary file 12 — EV Figure Source Data [file 44318_2025_381_MOESM12_ESM.zip › 44318_2025_381_MOESM12_ESM/Figure EV5/EV5C/AK/western eIF2Ba_AK.tif]

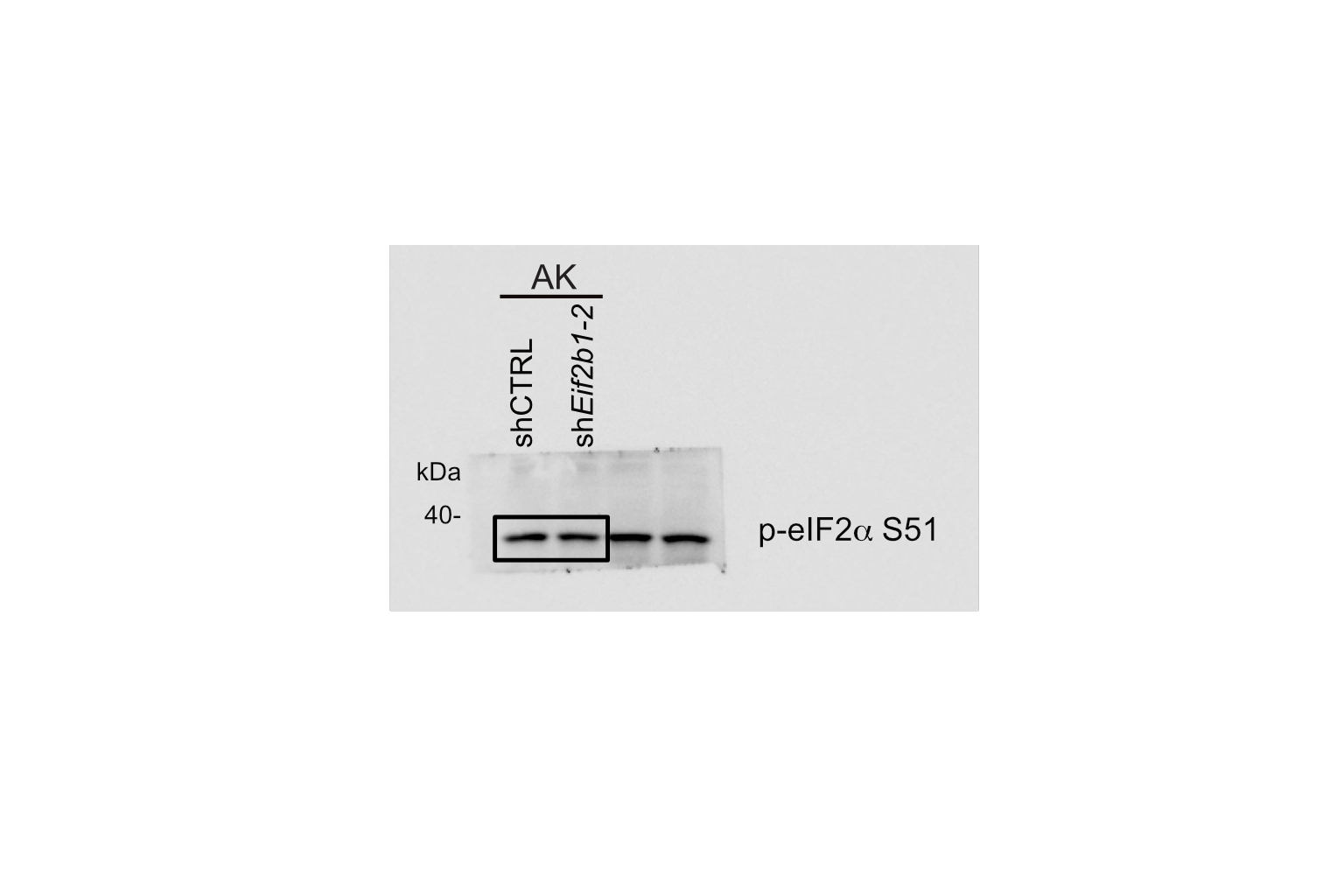

Supplement: Supplementary file 12 — EV Figure Source Data [file 44318_2025_381_MOESM12_ESM.zip › 44318_2025_381_MOESM12_ESM/Figure EV5/EV5C/AK/western p-eIF2a S51_AK.tif]

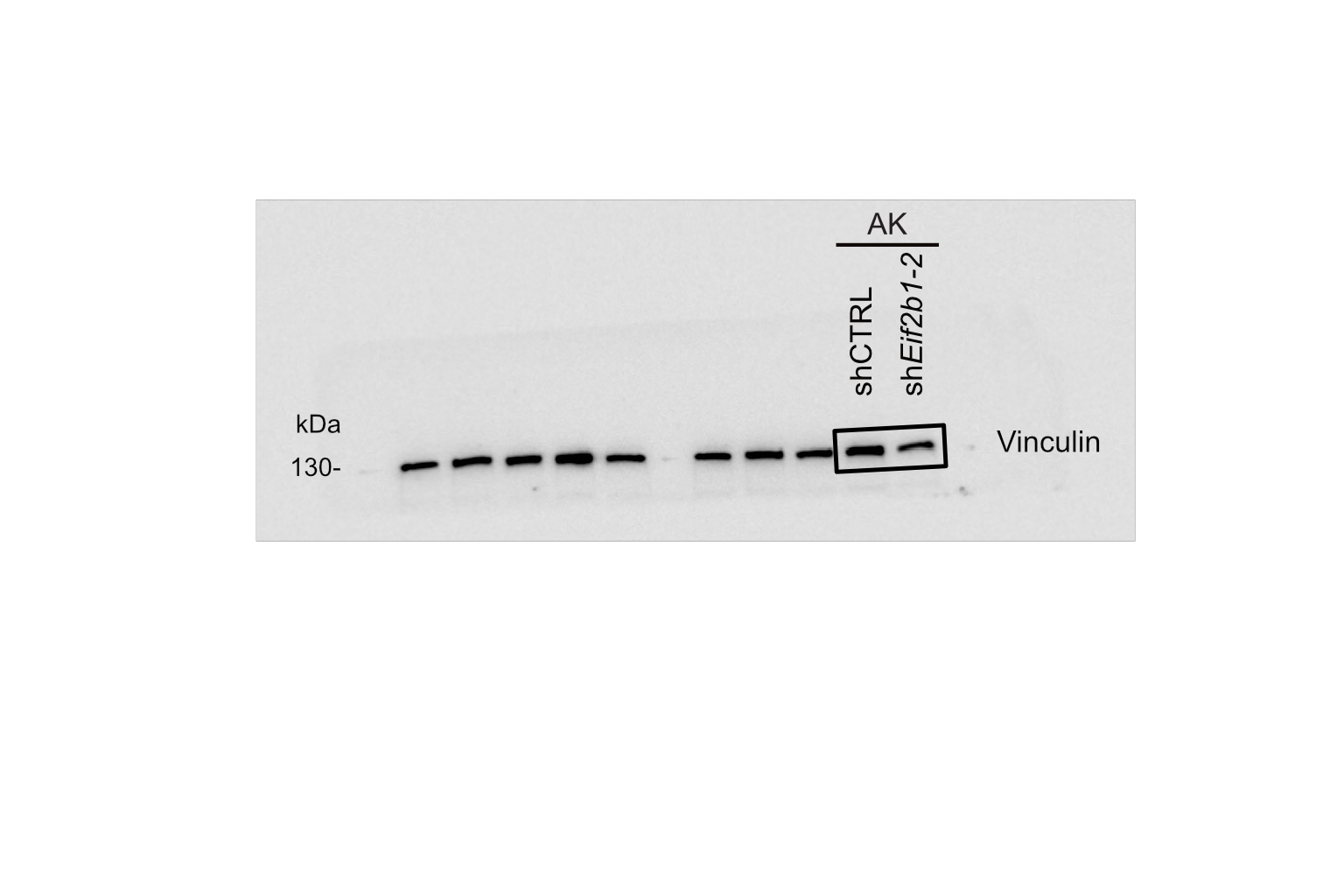

Supplement: Supplementary file 12 — EV Figure Source Data [file 44318_2025_381_MOESM12_ESM.zip › 44318_2025_381_MOESM12_ESM/Figure EV5/EV5C/AK/western vinculin_AK.tif]

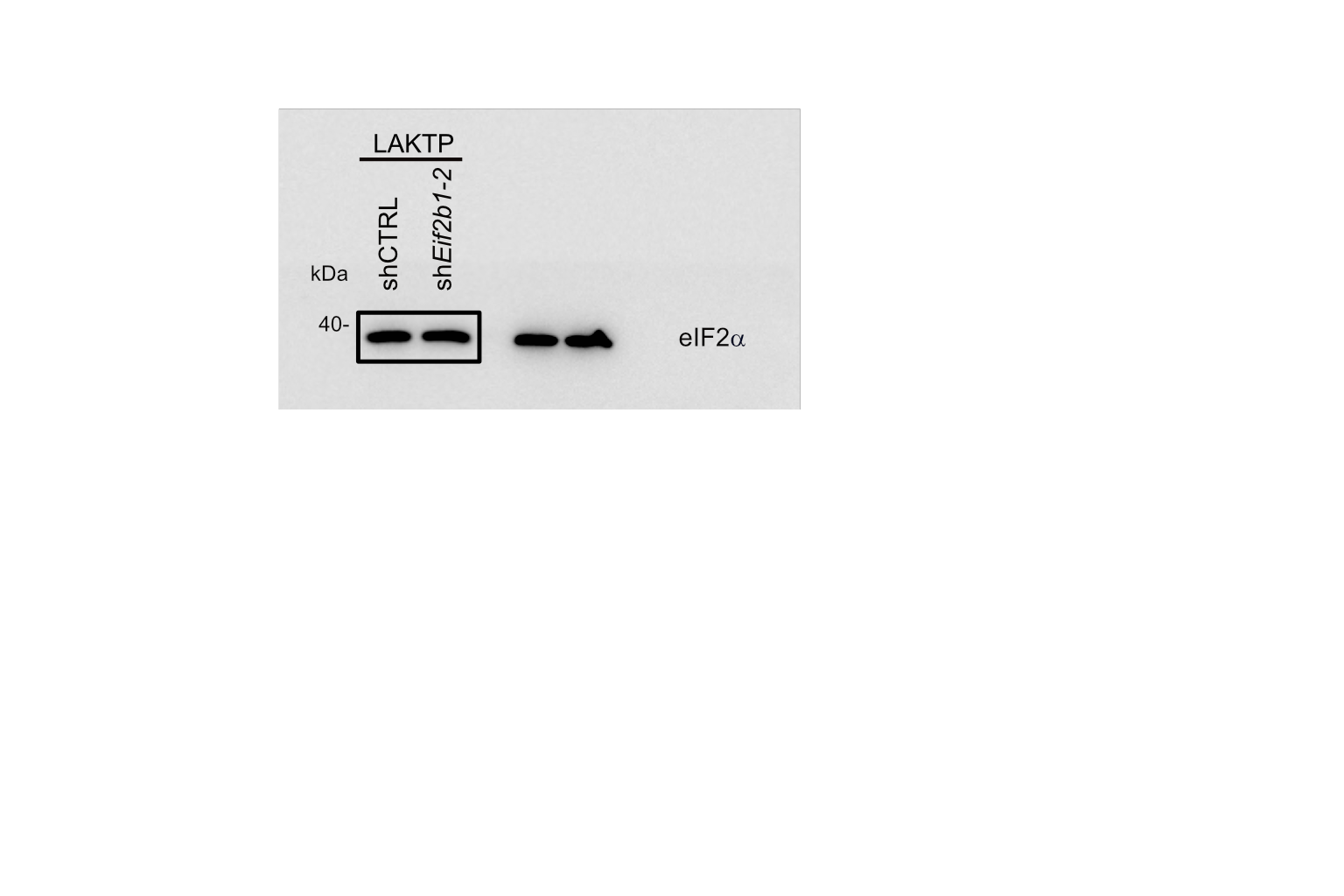

Supplement: Supplementary file 12 — EV Figure Source Data [file 44318_2025_381_MOESM12_ESM.zip › 44318_2025_381_MOESM12_ESM/Figure EV5/EV5C/LAKTP/western eIF2a_LAKTP.tif]

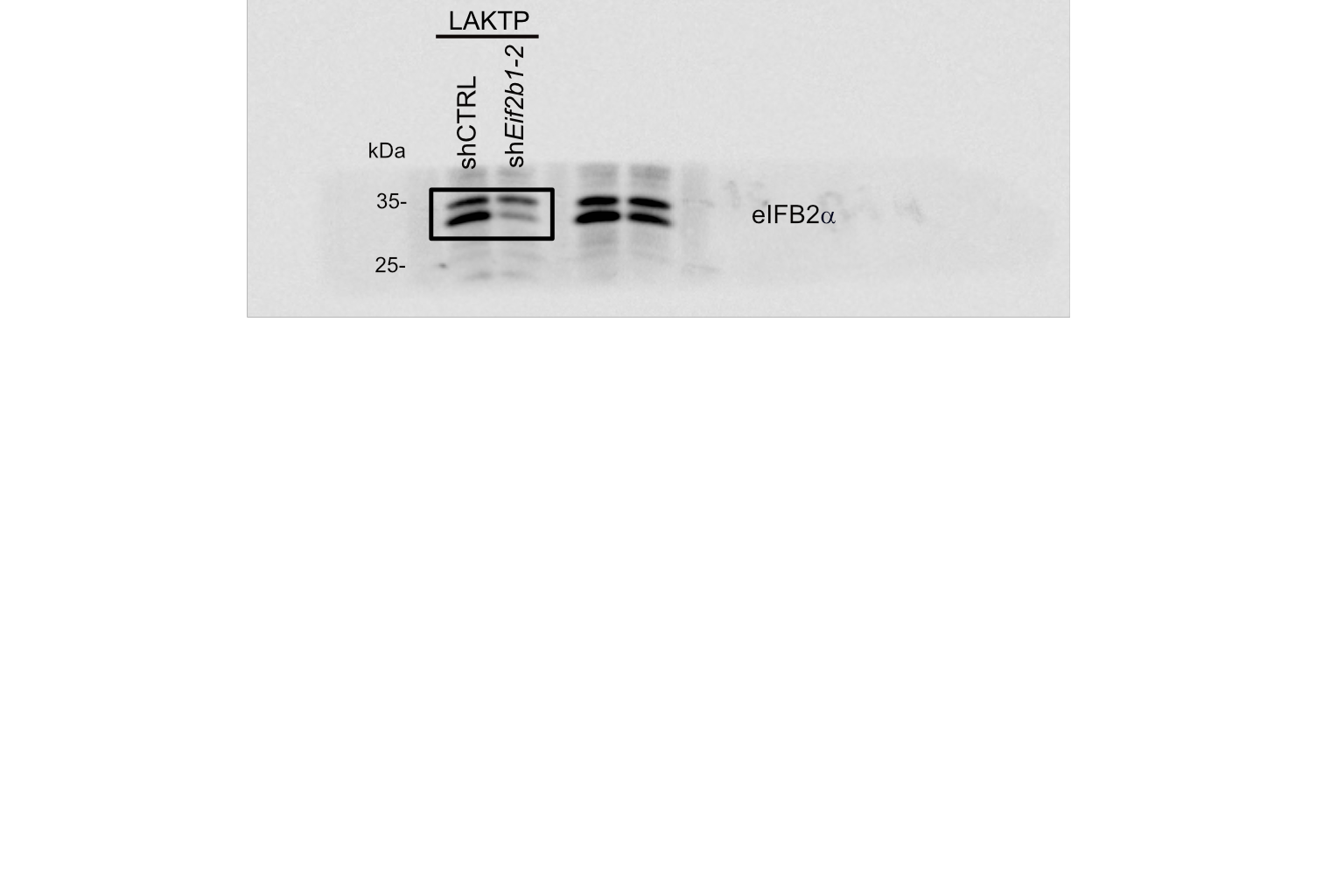

Supplement: Supplementary file 12 — EV Figure Source Data [file 44318_2025_381_MOESM12_ESM.zip › 44318_2025_381_MOESM12_ESM/Figure EV5/EV5C/LAKTP/western eIF2Ba_LAKTP.tif]

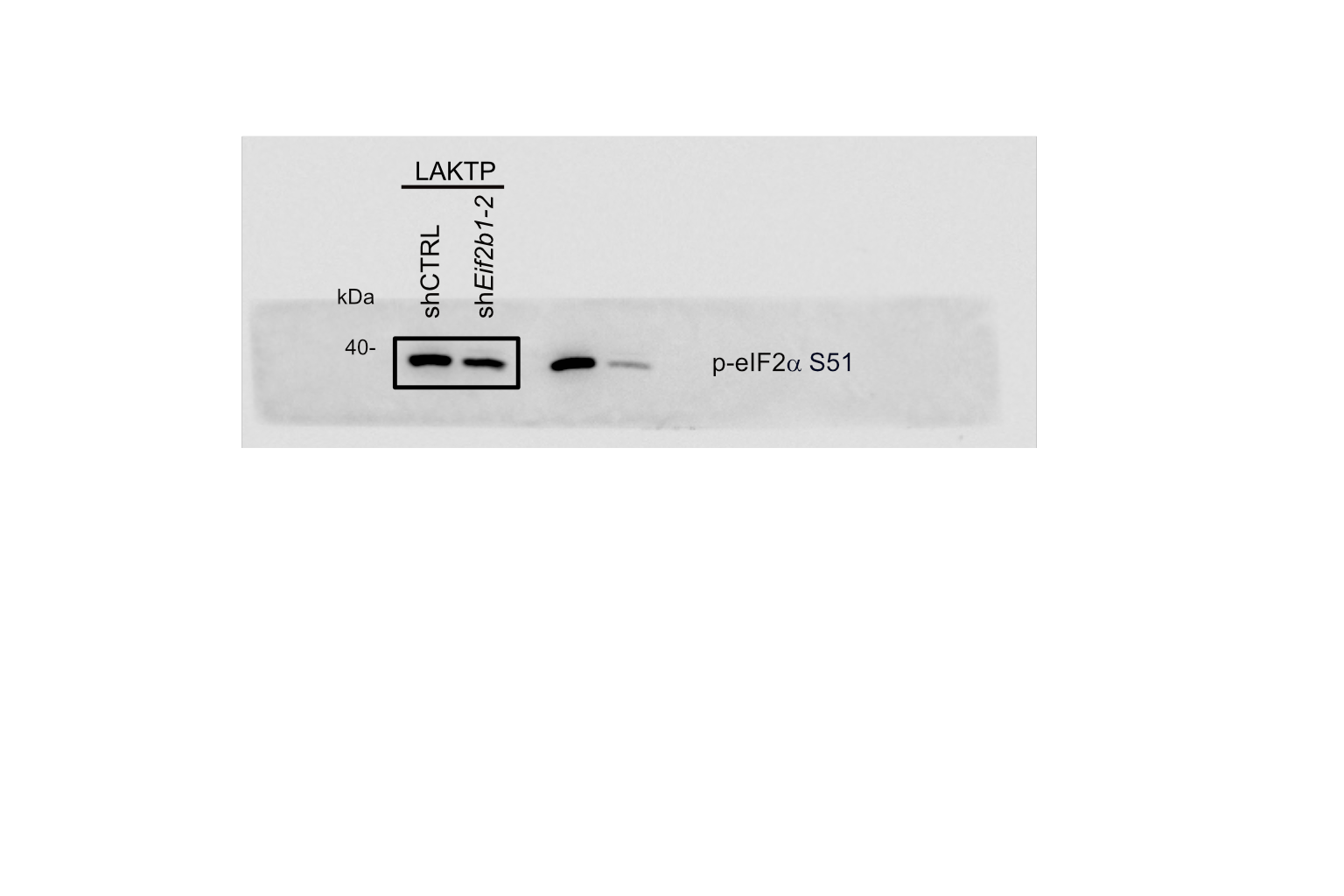

Supplement: Supplementary file 12 — EV Figure Source Data [file 44318_2025_381_MOESM12_ESM.zip › 44318_2025_381_MOESM12_ESM/Figure EV5/EV5C/LAKTP/western p-eIF2a S51_LAKTP.tif]

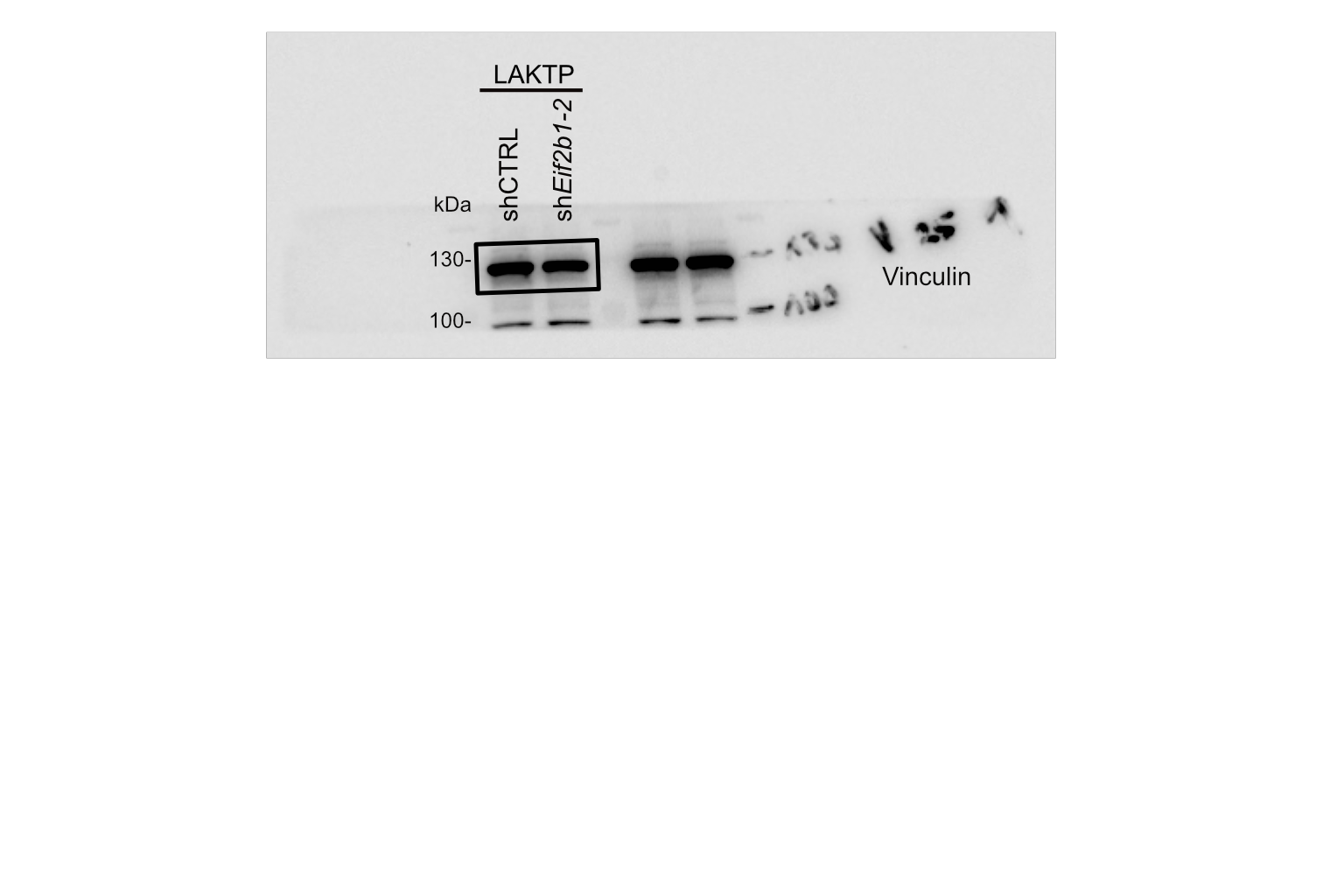

Supplement: Supplementary file 12 — EV Figure Source Data [file 44318_2025_381_MOESM12_ESM.zip › 44318_2025_381_MOESM12_ESM/Figure EV5/EV5C/LAKTP/western vinculin_LAKTP.tif]

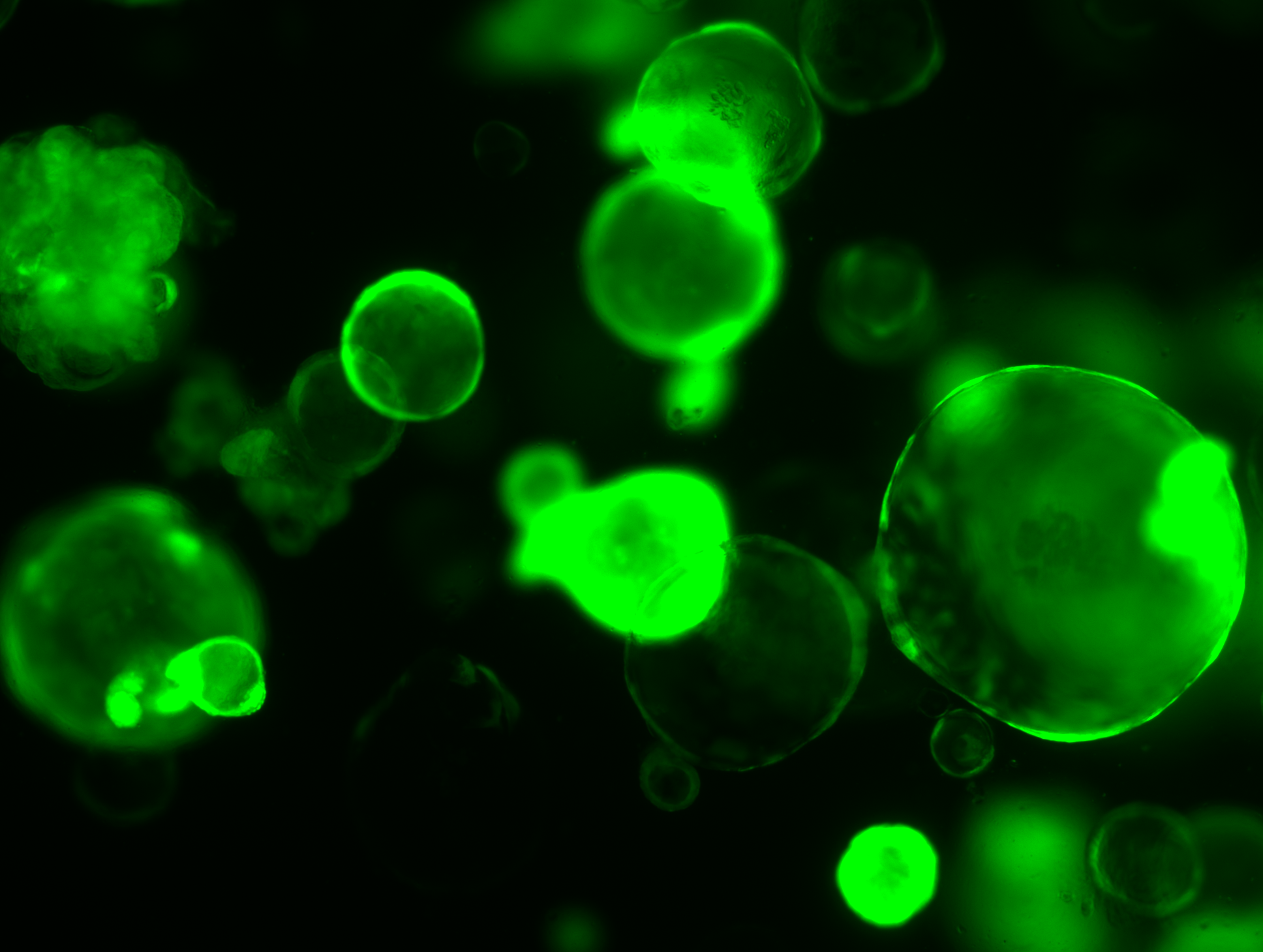

Supplement: Supplementary file 12 — EV Figure Source Data [file 44318_2025_381_MOESM12_ESM.zip › 44318_2025_381_MOESM12_ESM/Figure EV5/EV5E/FAP_shCTRL.tif]

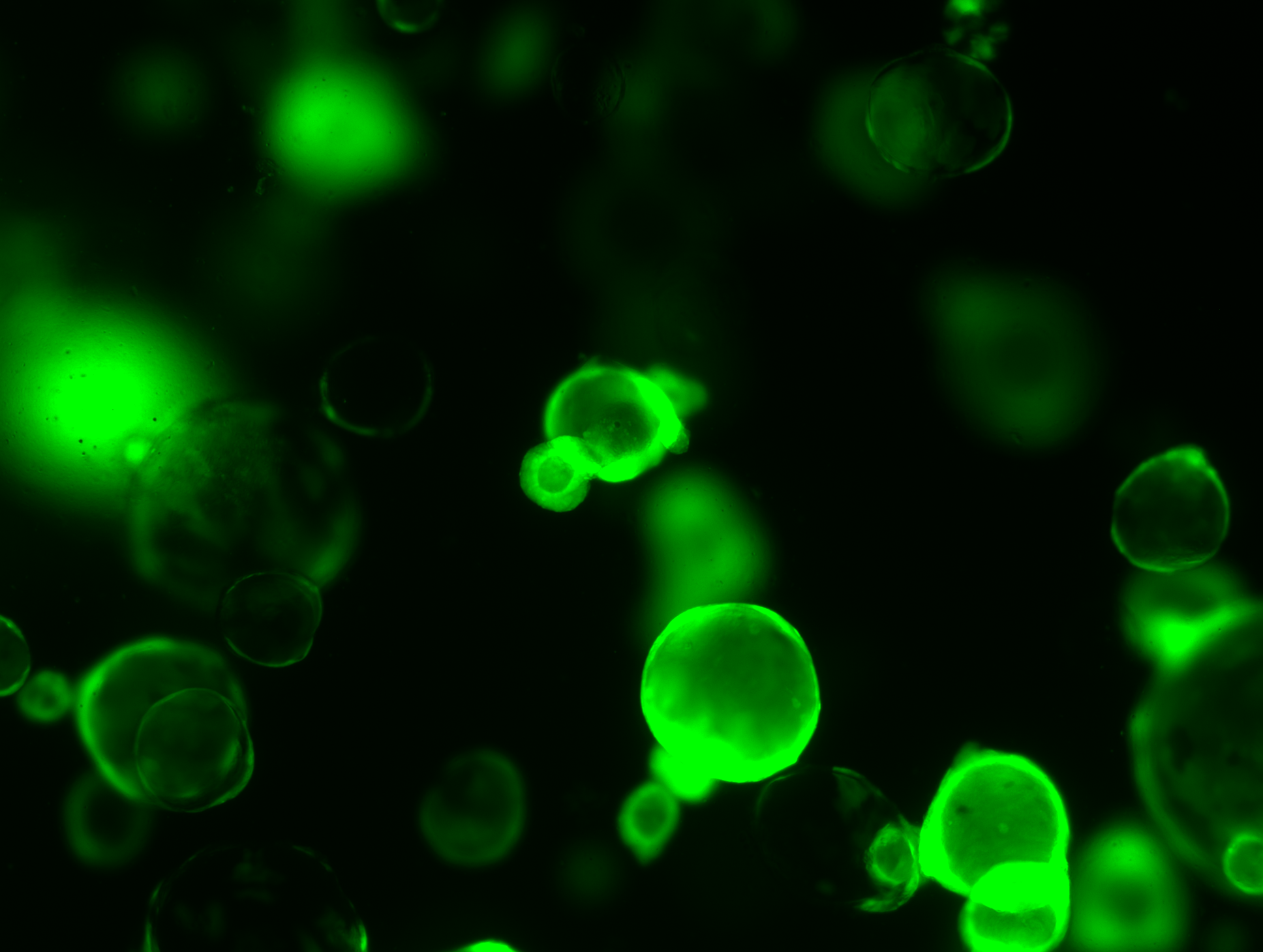

Supplement: Supplementary file 12 — EV Figure Source Data [file 44318_2025_381_MOESM12_ESM.zip › 44318_2025_381_MOESM12_ESM/Figure EV5/EV5E/FAP_shEIF2B1-2.tif]

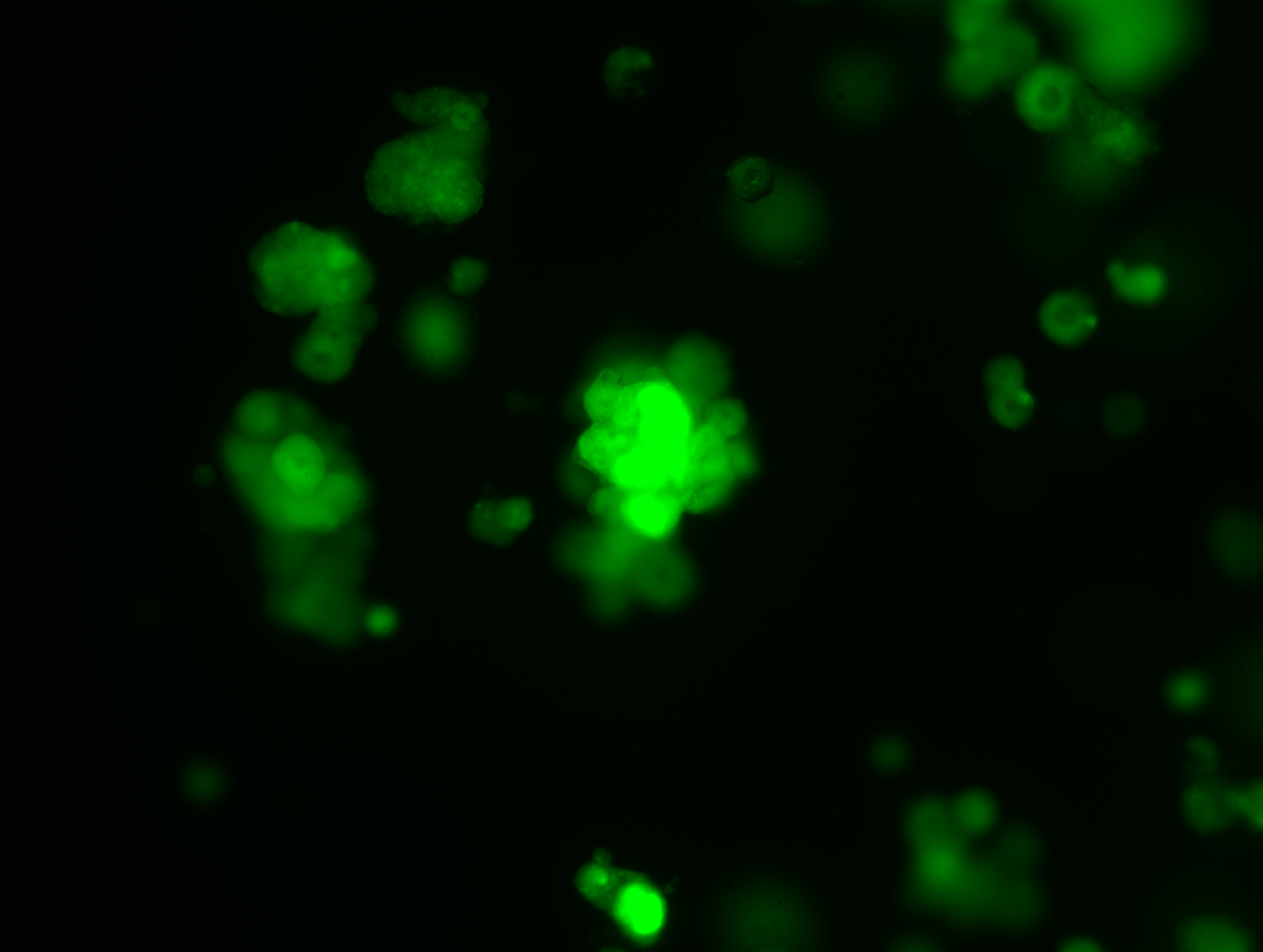

Supplement: Supplementary file 12 — EV Figure Source Data [file 44318_2025_381_MOESM12_ESM.zip › 44318_2025_381_MOESM12_ESM/Figure EV5/EV5E/HD-3_shCTRL.tif]

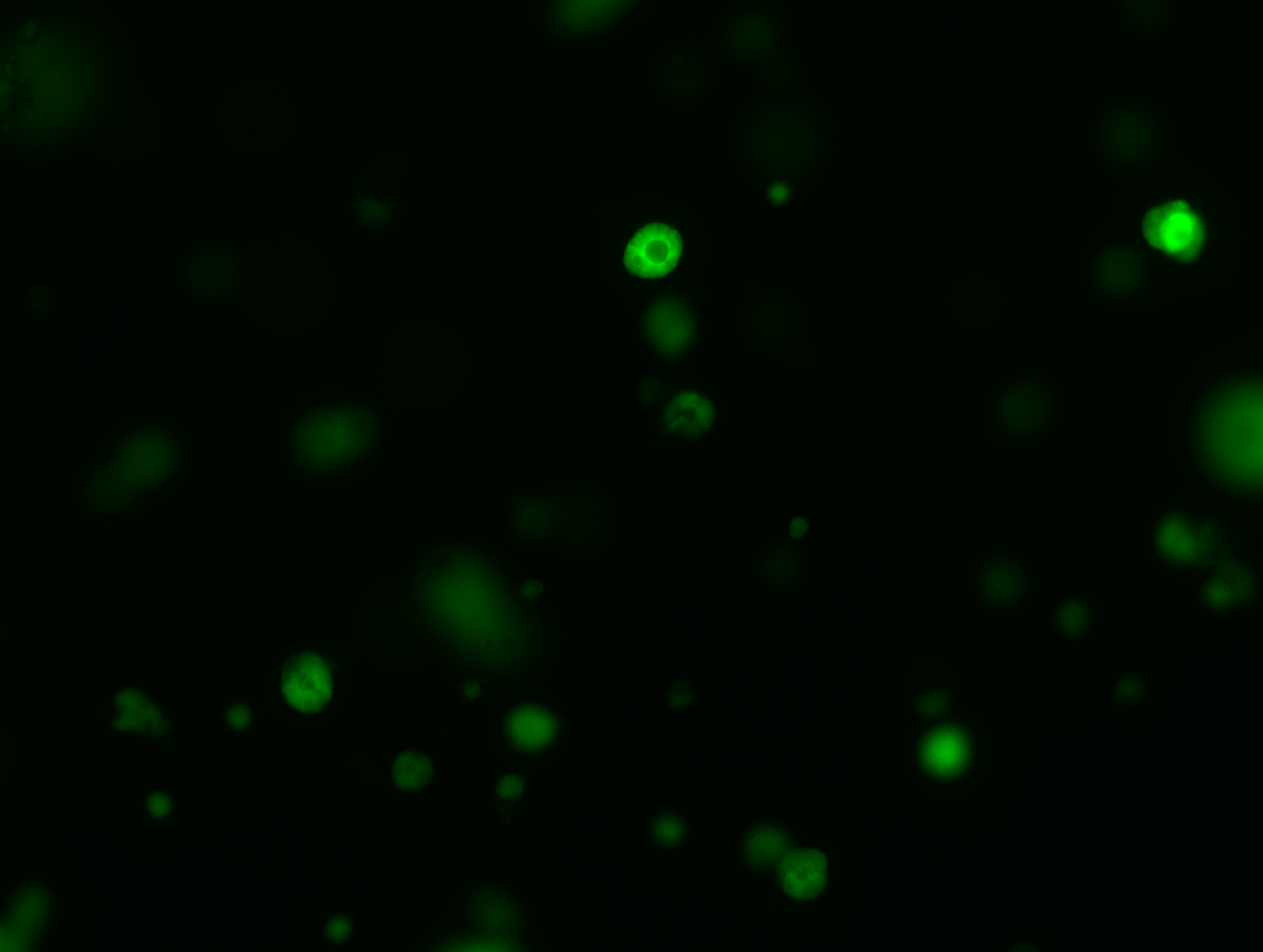

Supplement: Supplementary file 12 — EV Figure Source Data [file 44318_2025_381_MOESM12_ESM.zip › 44318_2025_381_MOESM12_ESM/Figure EV5/EV5E/HD-3_shEIF2B1-2.tif]

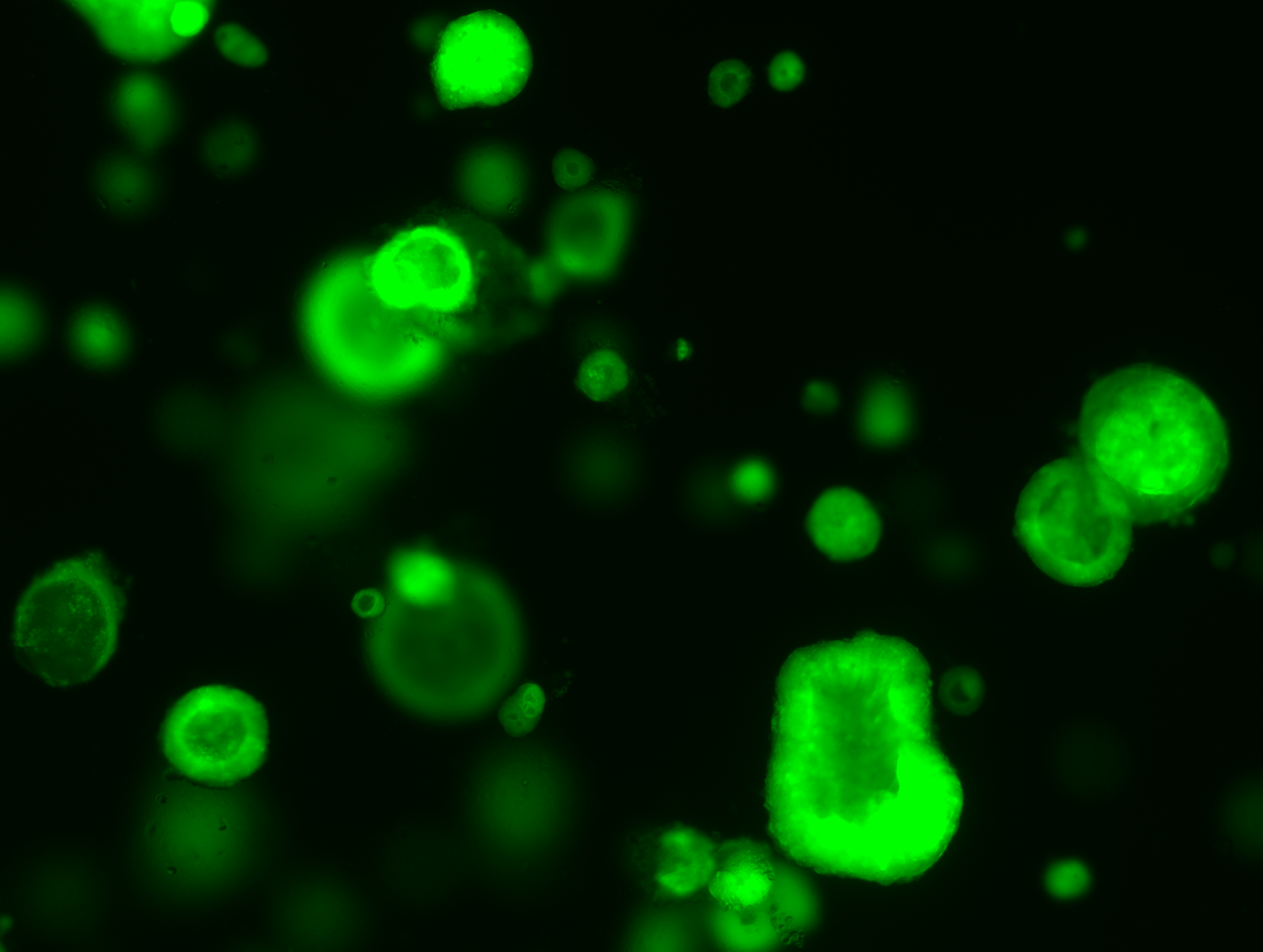

Supplement: Supplementary file 12 — EV Figure Source Data [file 44318_2025_381_MOESM12_ESM.zip › 44318_2025_381_MOESM12_ESM/Figure EV5/EV5E/T4_shCTRL.tif]

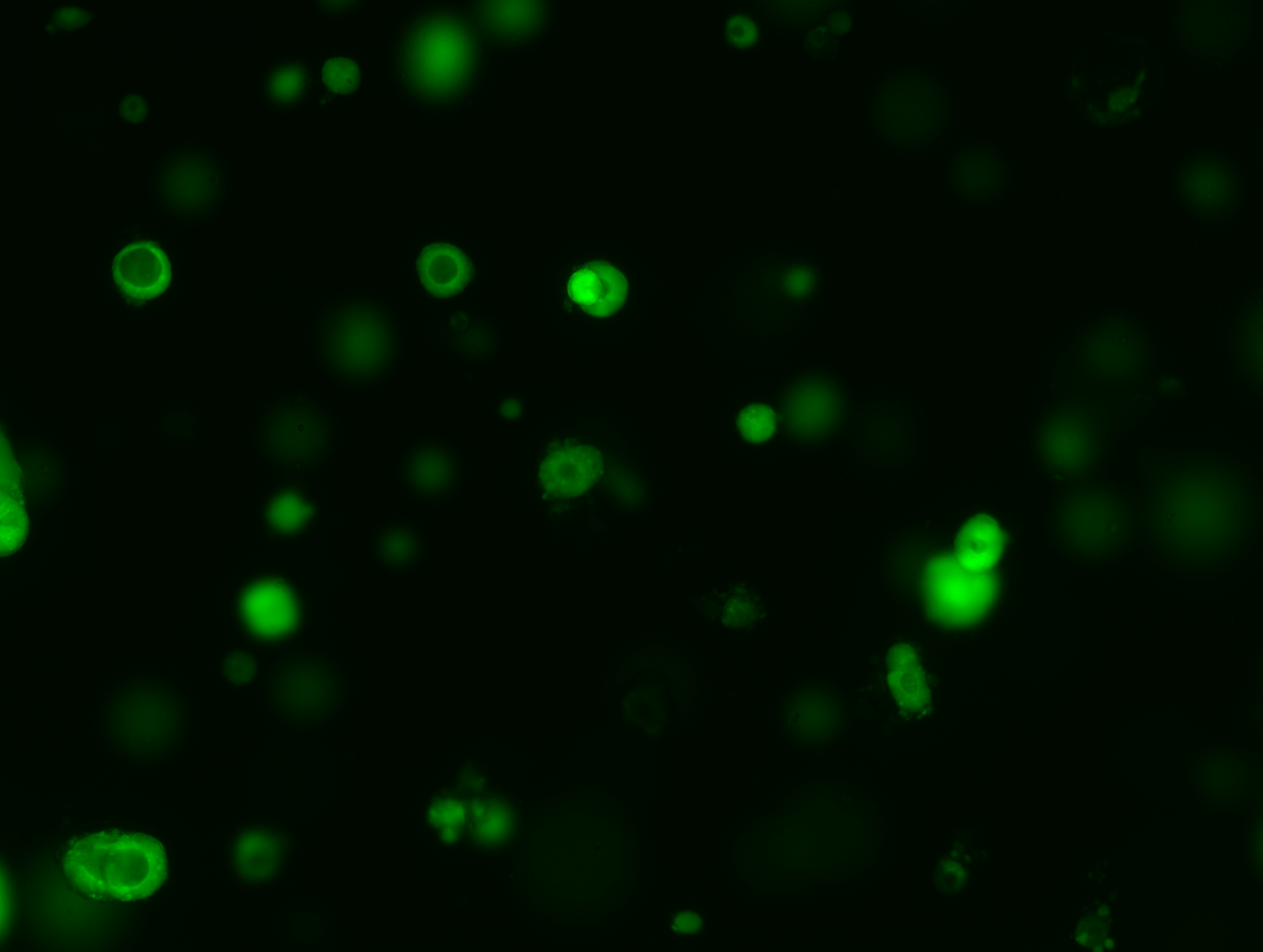

Supplement: Supplementary file 12 — EV Figure Source Data [file 44318_2025_381_MOESM12_ESM.zip › 44318_2025_381_MOESM12_ESM/Figure EV5/EV5E/T4_shEIF2B1-2.tif]

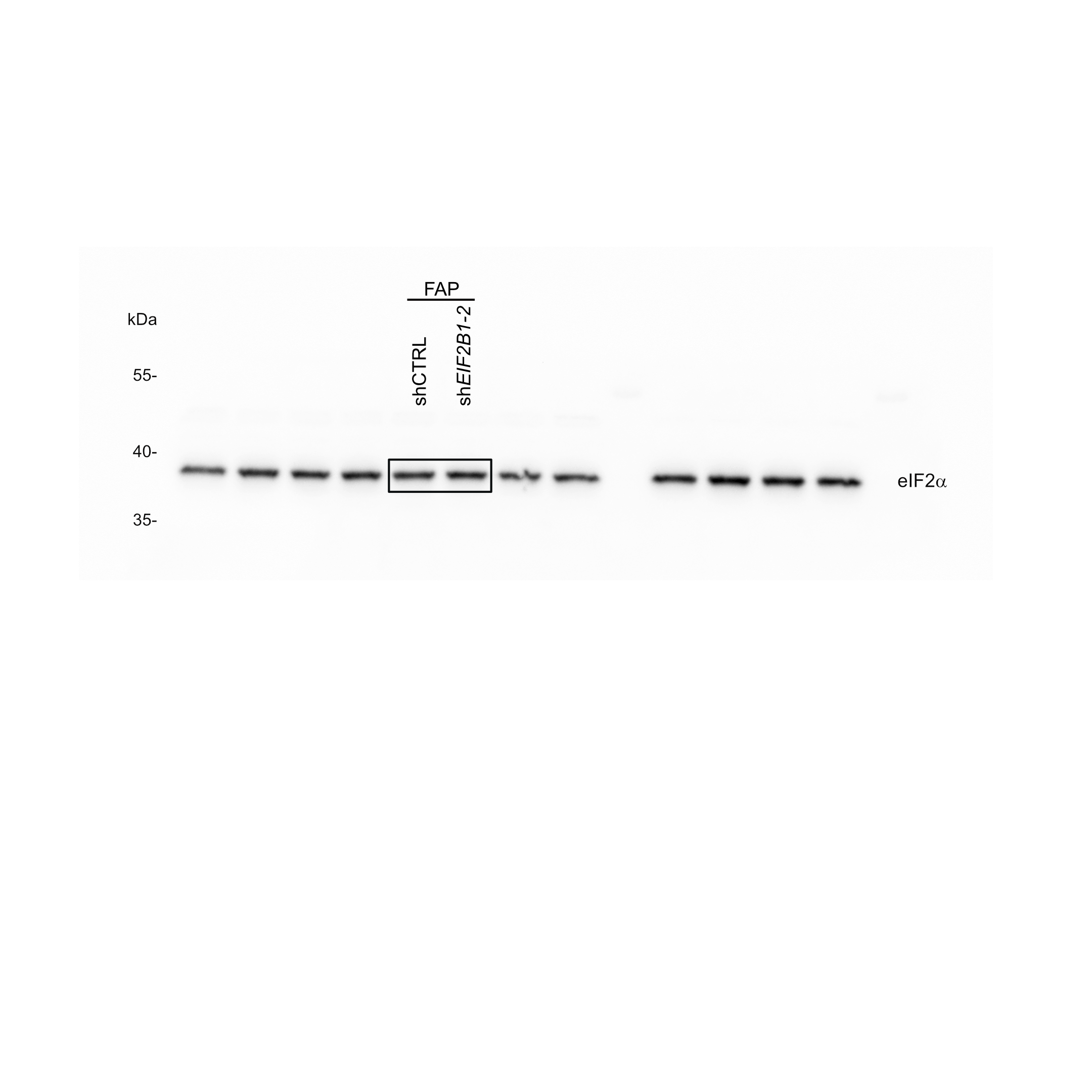

Supplement: Supplementary file 12 — EV Figure Source Data [file 44318_2025_381_MOESM12_ESM.zip › 44318_2025_381_MOESM12_ESM/Figure EV5/EV5G/western eIF2a FAP.tiff]

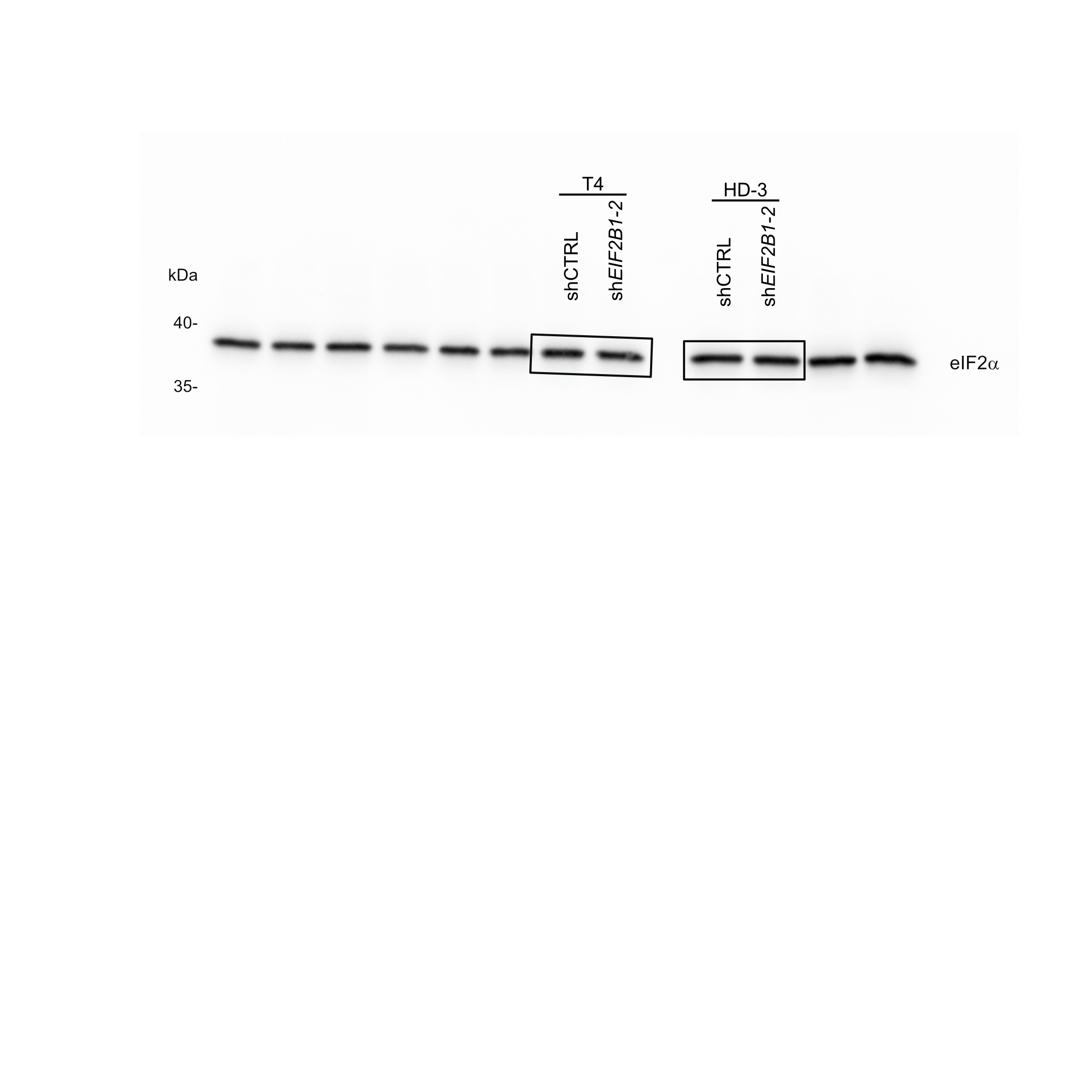

Supplement: Supplementary file 12 — EV Figure Source Data [file 44318_2025_381_MOESM12_ESM.zip › 44318_2025_381_MOESM12_ESM/Figure EV5/EV5G/western eIF2a HD-3,T4.tiff]

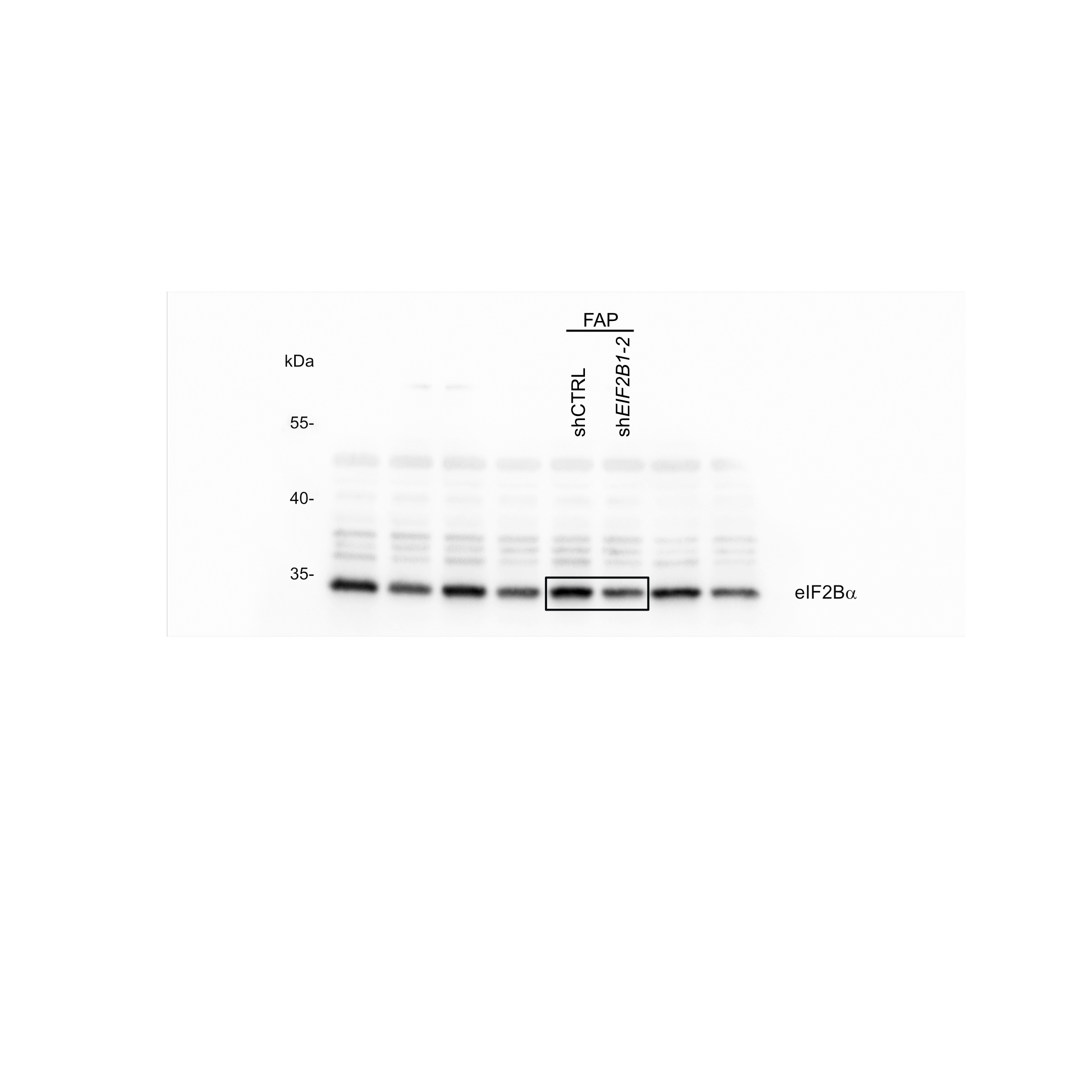

Supplement: Supplementary file 12 — EV Figure Source Data [file 44318_2025_381_MOESM12_ESM.zip › 44318_2025_381_MOESM12_ESM/Figure EV5/EV5G/western eIF2Ba FAP.tiff]

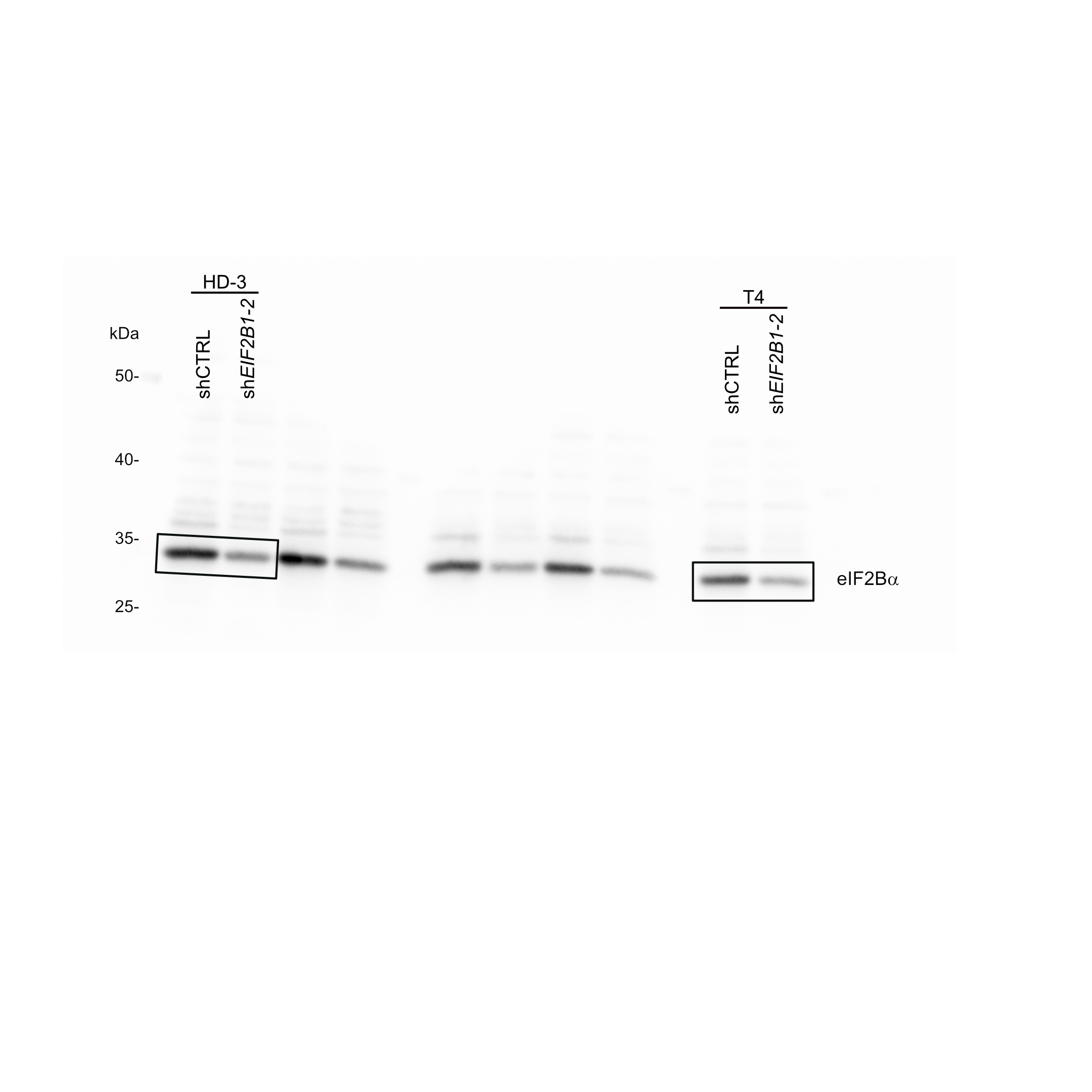

Supplement: Supplementary file 12 — EV Figure Source Data [file 44318_2025_381_MOESM12_ESM.zip › 44318_2025_381_MOESM12_ESM/Figure EV5/EV5G/western eIF2Ba HD-3,T4.Tif]

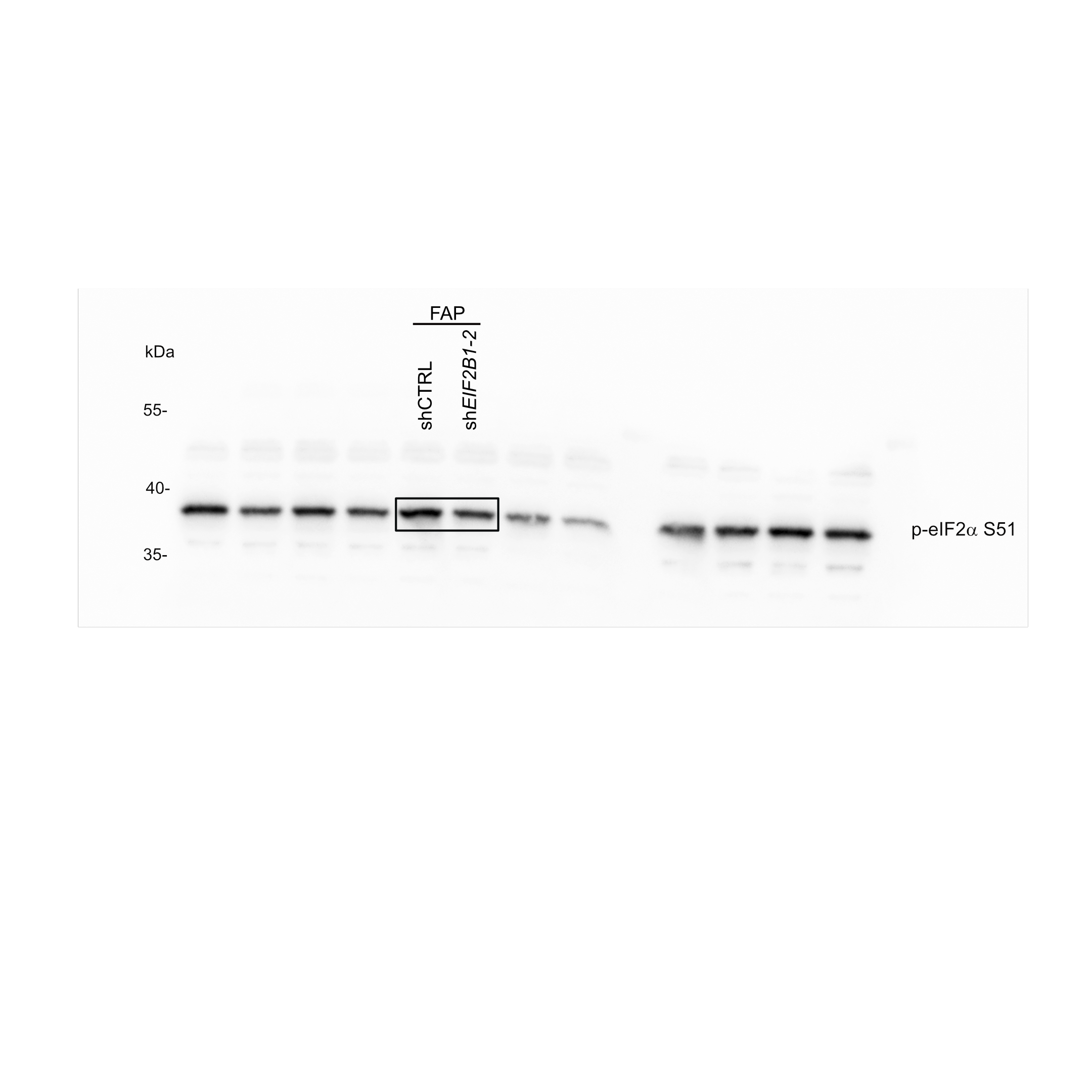

Supplement: Supplementary file 12 — EV Figure Source Data [file 44318_2025_381_MOESM12_ESM.zip › 44318_2025_381_MOESM12_ESM/Figure EV5/EV5G/western p-eIF2a S51 FAP.tiff]

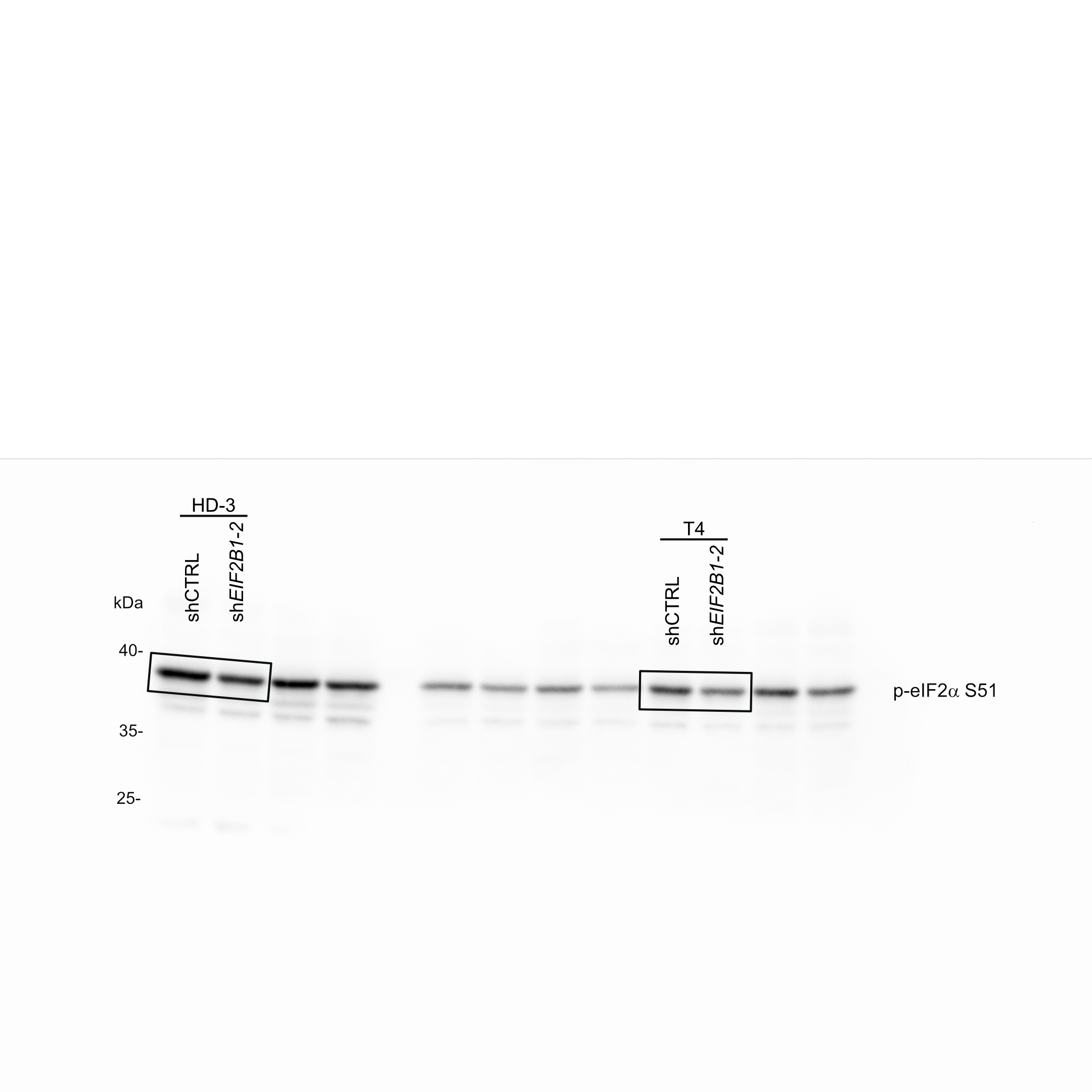

Supplement: Supplementary file 12 — EV Figure Source Data [file 44318_2025_381_MOESM12_ESM.zip › 44318_2025_381_MOESM12_ESM/Figure EV5/EV5G/western p-eIF2a S51 HD-3,T4.Tif]

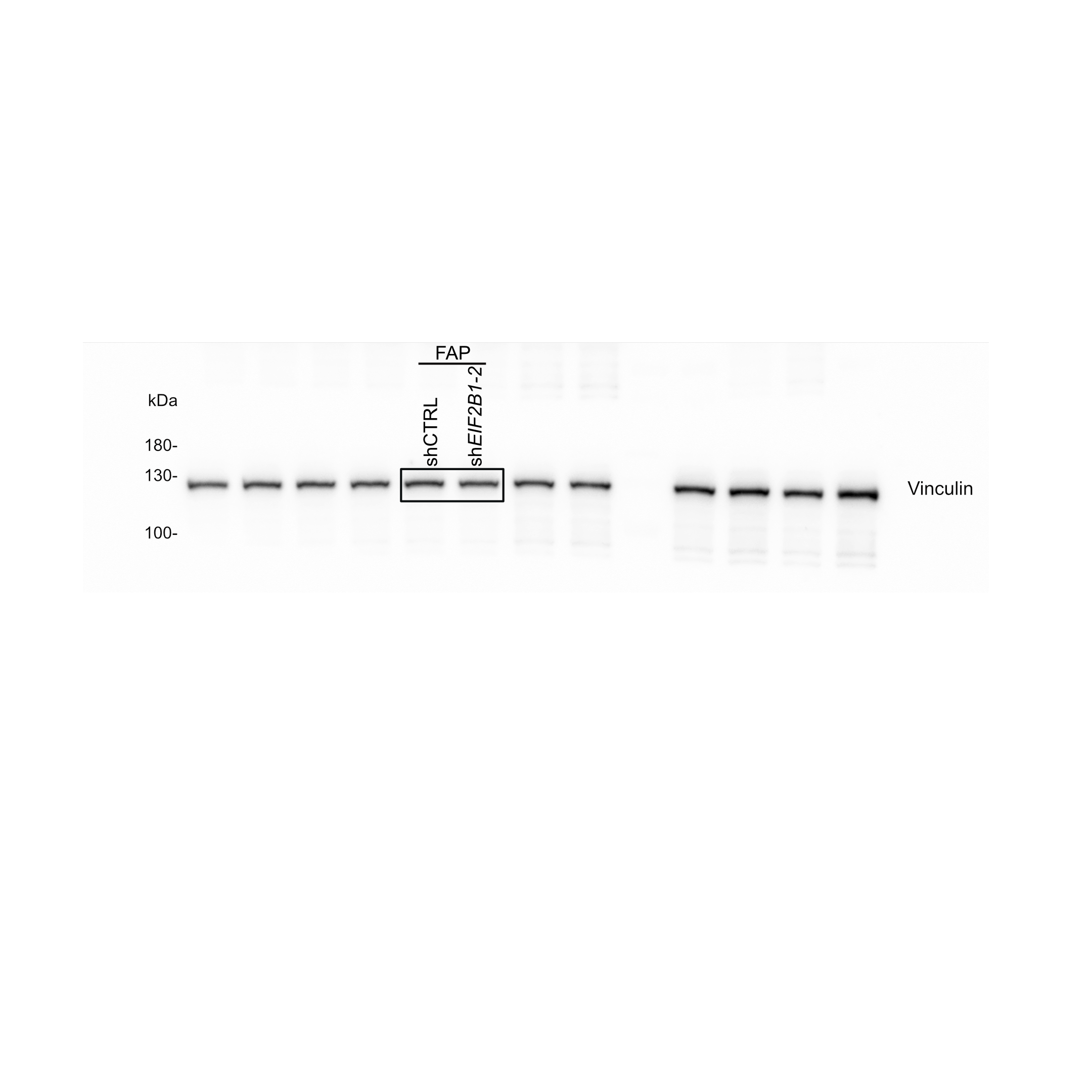

Supplement: Supplementary file 12 — EV Figure Source Data [file 44318_2025_381_MOESM12_ESM.zip › 44318_2025_381_MOESM12_ESM/Figure EV5/EV5G/western vinculin FAP.tiff]

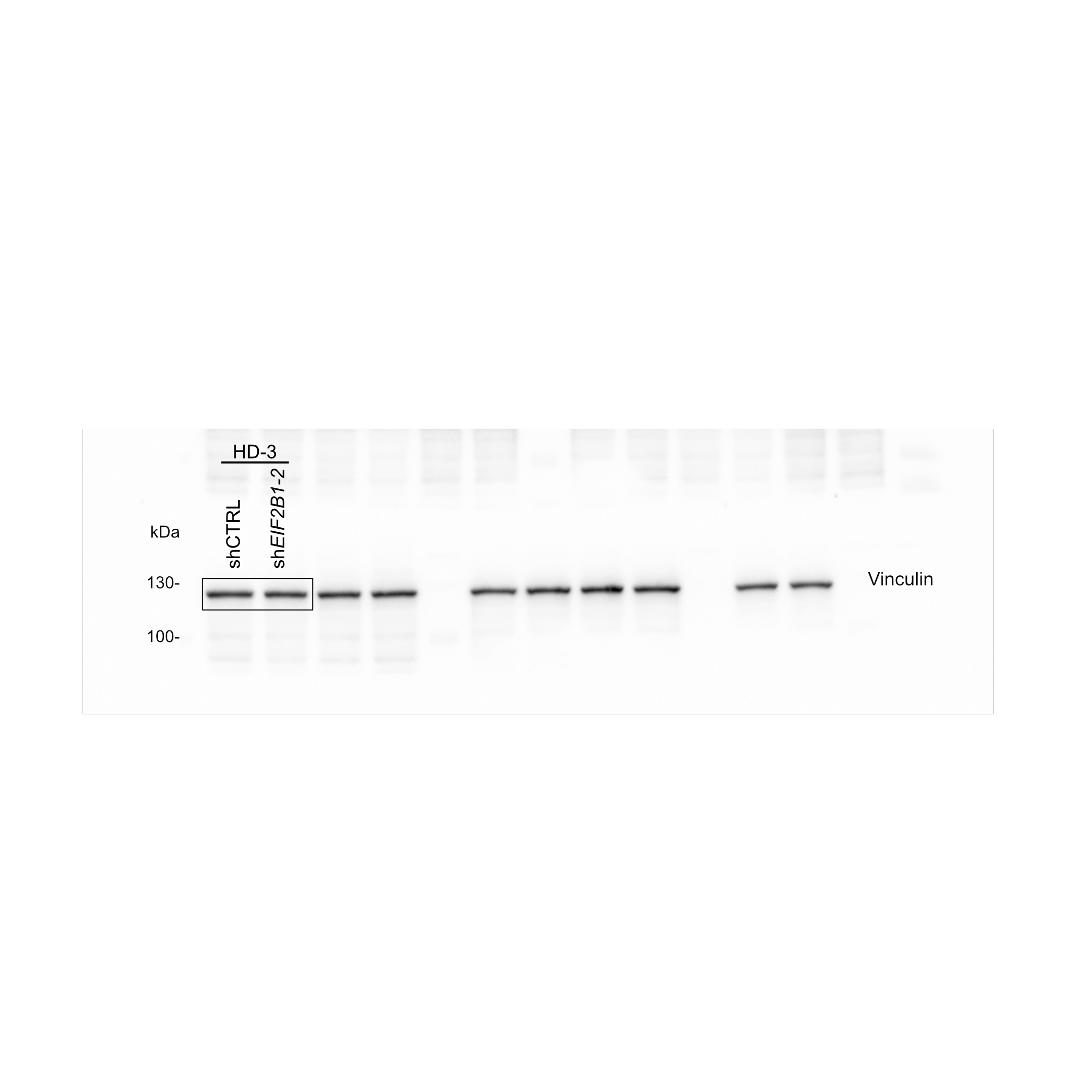

Supplement: Supplementary file 12 — EV Figure Source Data [file 44318_2025_381_MOESM12_ESM.zip › 44318_2025_381_MOESM12_ESM/Figure EV5/EV5G/western vinculin HD-3.tiff]

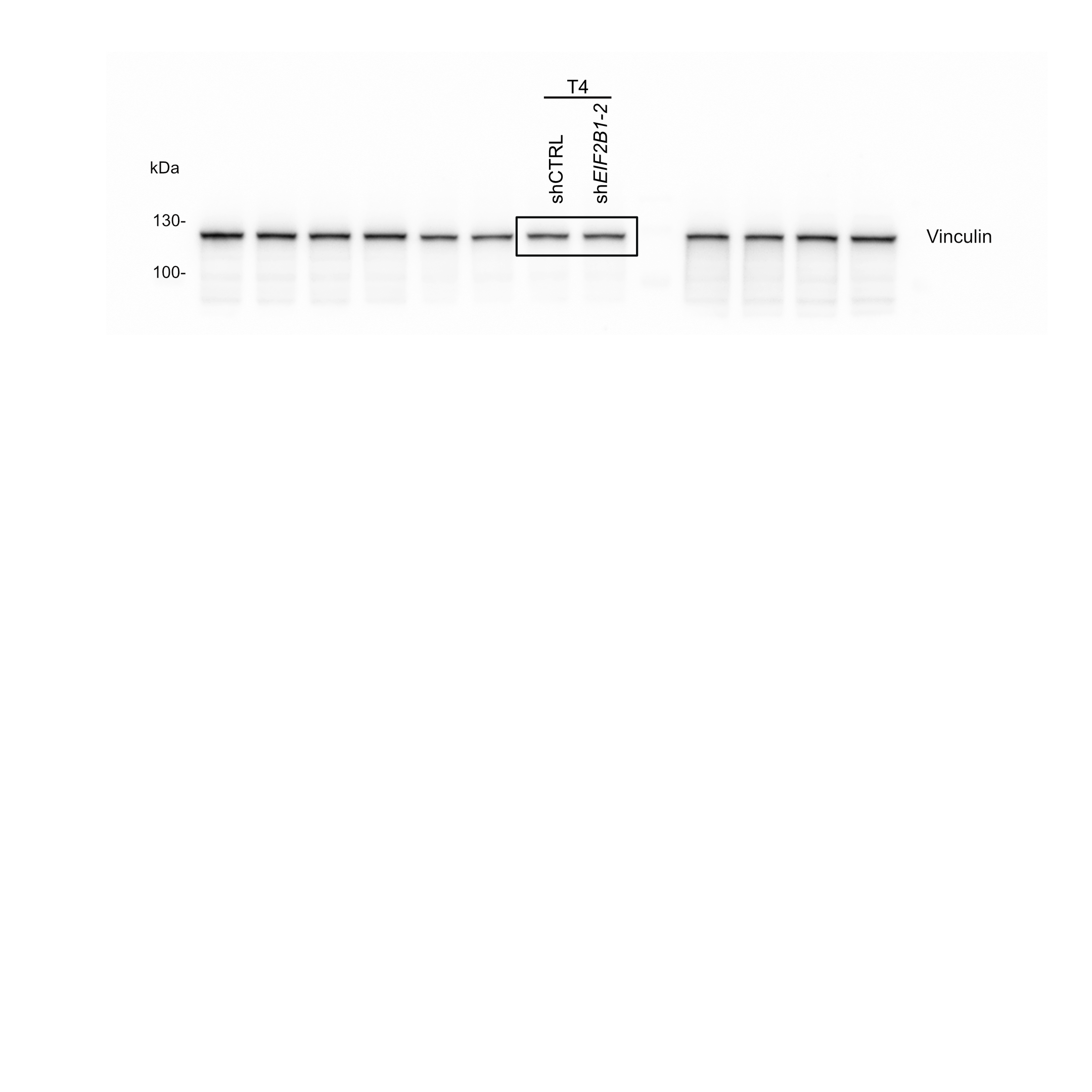

Supplement: Supplementary file 12 — EV Figure Source Data [file 44318_2025_381_MOESM12_ESM.zip › 44318_2025_381_MOESM12_ESM/Figure EV5/EV5G/western vinculin T4.tiff]
