# Supplementary material for: Maintenance of p-eIF2α levels by the eIF2B complex is vital for colorectal cancer
Source: EMBO J. 2025 Feb 27;44(7):2075–105. doi: 10.1038/s44318-025-00381-9 (PMC11962125; doi:10.1038/s44318-025-00381-9)
Supplement: Supplementary file 13 — Expanded View Figures [file 44318_2025_381_MOESM13_ESM.pdf]

Expanded View Figures

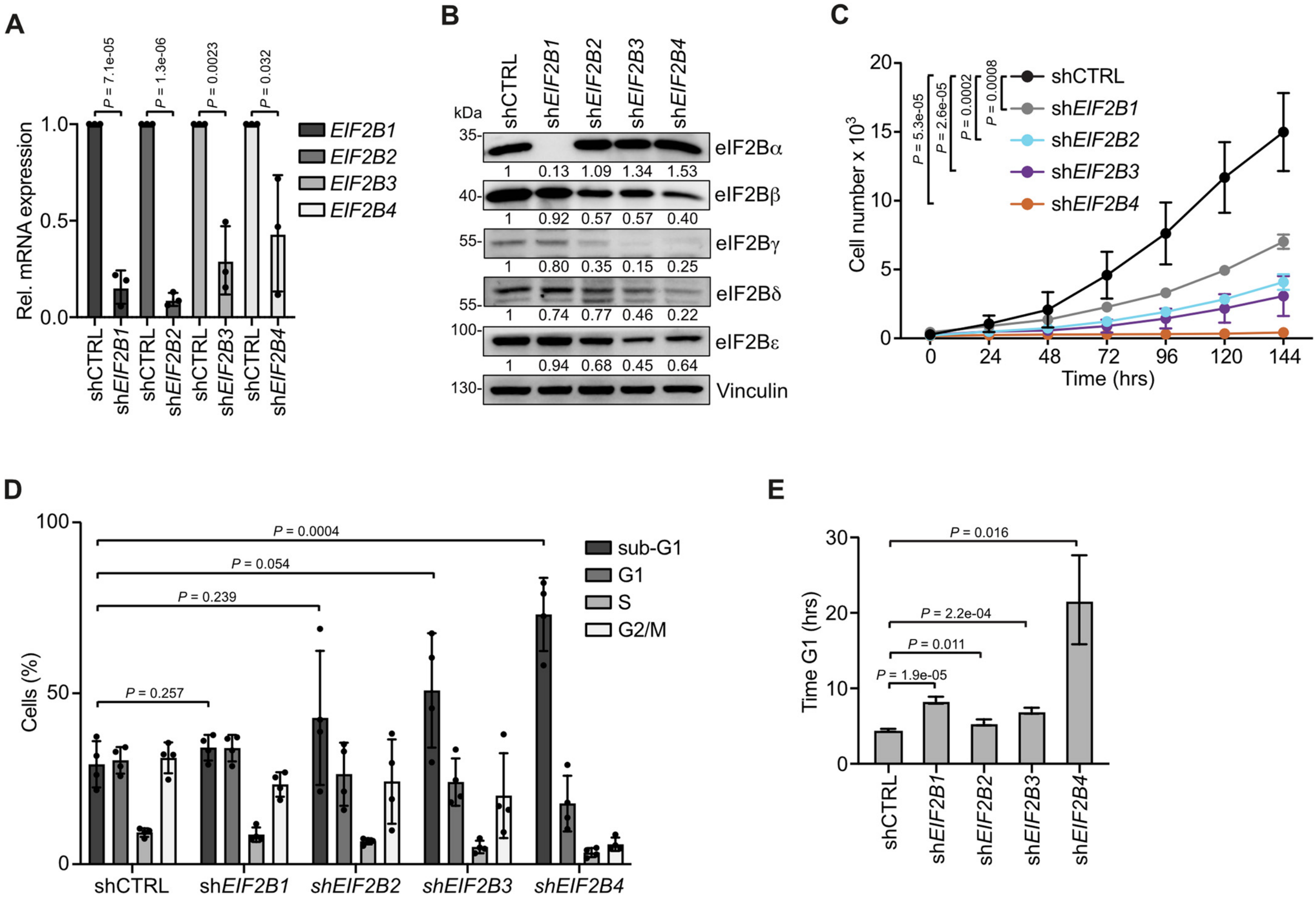

Figure EV1. DLD1 cells show reduced viability upon eIF2B subunit depletion.

(**A**) mRNA expression of indicated genes in SW480 cells transduced with shCTRL or shRNAs against *EIF2B1-4*. Data show mean ± s.d. ($n = 3$ biological replicates); Student's *t* test. (**B**) Western blot of indicated proteins in DLD1 cells transduced with shCTRL or shRNAs against *EIF2B1-4*, representative of three biological replicates with similar results. Levels of the respective eIF2B subunits, relative to vinculin, are given below each corresponding panel. (**C**) Growth curve of DLD1 cells transduced as described in (**B**), measured with Operetta screening microscope. Data show mean ± s.d. ($n = 6$ biological replicates); Student's *t* test. (**D**) PI cell cycle FACS analysis of DLD1 cells transduced as described in (**B**). Data show mean ± s.d. ($n = 4$ biological replicates); Student's *t* test. (**E**) Length of G1 cell cycle phase of DLD1 cells transduced as described in (**B**), calculated with data acquired from growth curve in (**C**) and PI cell cycle FACS in (**D**). Data show mean ± s.e.m.; Student's *t* test. Source data are available online for this figure.

 © The Author(s)

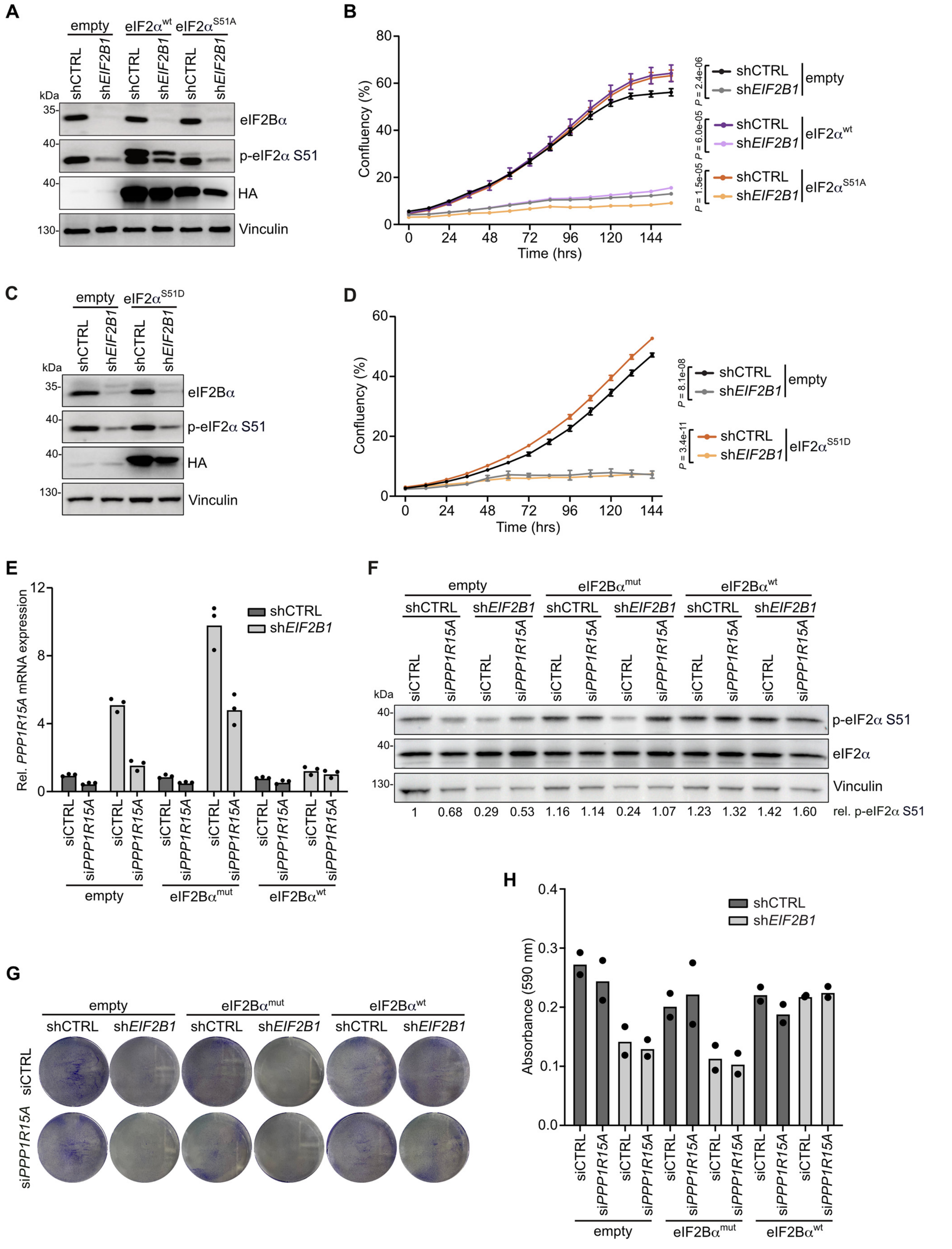
A
empty
eIF2α wt
eIF2α S51A
shCTRL
shEIF2B1
kDa
35-
40-
40-
130-
eIF2Bα
p-eIF2α S51
HA
Vinculin
B
Confluency (%)
Time (hrs)
0
24
48
72
96
120
144
80
60
40
20
P = 2.4e-06
P = 6.0e-05
P = 1.5e-05
shCTRL
shEIF2B1
empty
eIF2α wt
eIF2α S51A
C
empty
eIF2α S51D
shCTRL
shEIF2B1
kDa
35-
40-
40-
130-
eIF2Bα
p-eIF2α S51
HA
Vinculin
D
Confluency (%)
Time (hrs)
P = 8.1e-08
P = 3.4e-11
shCTRL
shEIF2B1
empty
eIF2α S51D
E
Rel. PPP1R15A mRNA expression
12
8
4
0
shCTRL
shEIF2B1
siCTRL
siPPP1R15A
empty
eIF2Bα mut
eIF2Bα wt
F
empty
eIF2Bα mut
eIF2Bα wt
shCTRL
shEIF2B1
siCTRL
siPPP1R15A
kDa
40-
40-
130-
p-eIF2α S51
eIF2α
Vinculin
1
0.68
0.29
0.53
1.16
1.14
0.24
1.07
1.23
1.32
1.42
1.60
rel. p-eIF2α S51
G
empty
eIF2Bα mut
eIF2Bα wt
shCTRL
shEIF2B1
siCTRL
siPPP1R15A
H
Absorbance (590 nm)
0.4
0.3
0.2
0.1
0.0
shCTRL
shEIF2B1
siCTRL
siPPP1R15A
empty
eIF2Bα mut
eIF2Bα wt

Figure EV2. Restoration of eIF2α phosphorylation does not rescue the viability defect upon eIF2Bα modulation.

(**A**) Western blot of indicated proteins in shCTRL- or sh*EIF2B1*-transduced SW480 cells stably overexpressing eIF2α WT (eIF2α^{wt}), eIF2α S51A mutant (eIF2α^{S51A}) construct, or without any overexpression (empty). The western blot is representative of three biological replicates with similar results. (**B**) Growth curve of SW480 cells transduced as described in (**A**), measured with Incucyte® live-cell imaging system. Data show mean ± s.d. ($n = 4$ biological replicates); Student's *t* test. (**C**) Western blot of indicated proteins in shCTRL- or sh*EIF2B1*-transduced SW480 cells stably overexpressing eIF2α S51D mutant (eIF2α^{S51D}) construct, or without any overexpression (empty). The western blot is representative of three biological replicates with similar results. (**D**) Growth curve of SW480 cells transduced as described in (**C**), measured with Incucyte® live-cell imaging system. Data show mean ± s.d. ($n = 4$ biological replicates); Student's *t* test. (**E**) mRNA expression of *PPP1R15A* in shCTRL- or sh*EIF2B1*-transduced SW480 cells stably overexpressing eIF2Bα mutant (eIF2Bα^{mut}), eIF2Bα WT (eIF2Bα^{wt}) construct, or without any overexpression (empty), transfected with siCTRL or si*PPP1R15A* for 72 hr. Data show mean of technical triplicates of one representative experiment ($n = 2$ biological replicates). (**F**) Western blot of indicated proteins in SW480 cells transduced and transfected as described in (**E**). The western blot is representative of two biological replicates with similar results. Levels of p-eIF2α S51, relative to total eIF2α, are given below. (**G**) Crystal violet staining of SW480 cells transduced and transfected as described in (**E**). Staining was done 7 days after seeding. Pictures are representative of two biological replicates with similar results. (**H**) Quantification of crystal violet staining described in (**G**). Data show mean ($n = 2$ biological replicates). Source data are available online for this figure.

 © The Author(s)

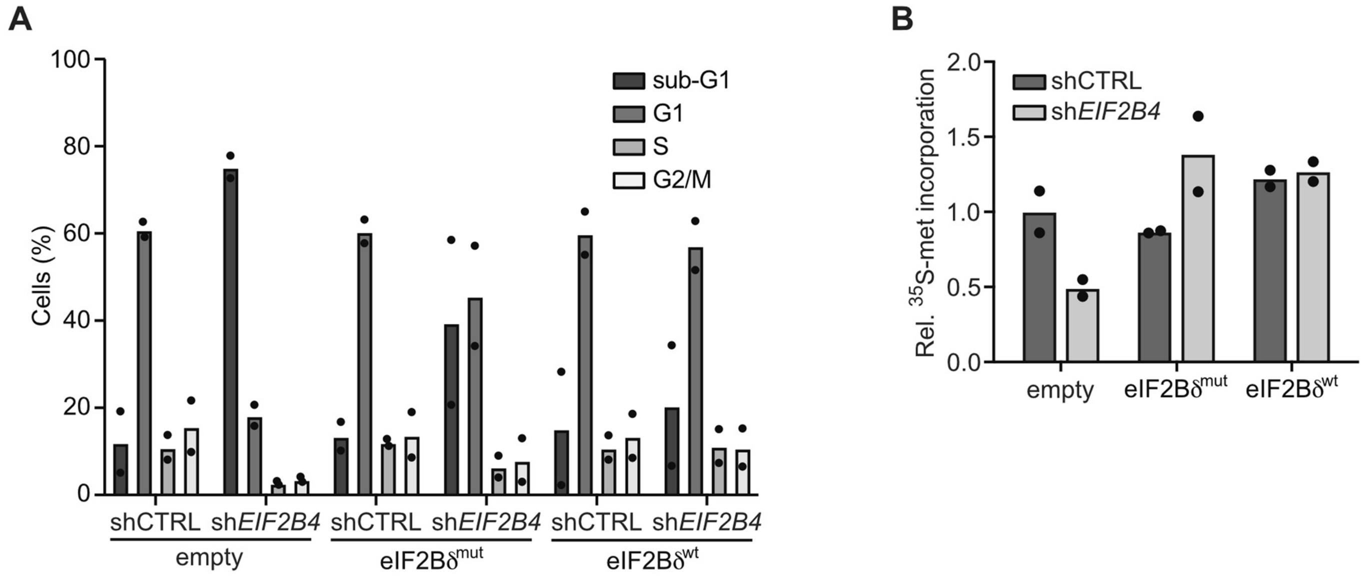

Figure EV3. eIF2Bδ^{mut} expression rescues protein synthesis rates but not the viability defect upon eIF2Bδ depletion.

(**A**) PI cell cycle FACS analysis of shCTRL- or sh*EIF2B4*-transduced SW480 cells stably overexpressing eIF2Bδ mutant (eIF2Bδ^{mut}), eIF2Bδ WT (eIF2Bδ^{wt}) construct, or without any overexpression (empty). Data show mean ($n = 2$ biological replicates). (**B**) Relative ^{35}S-methionine incorporation of SW480 cells transduced as described in (**A**). Data show mean ($n = 2$ biological replicates). Source data are available online for this figure.

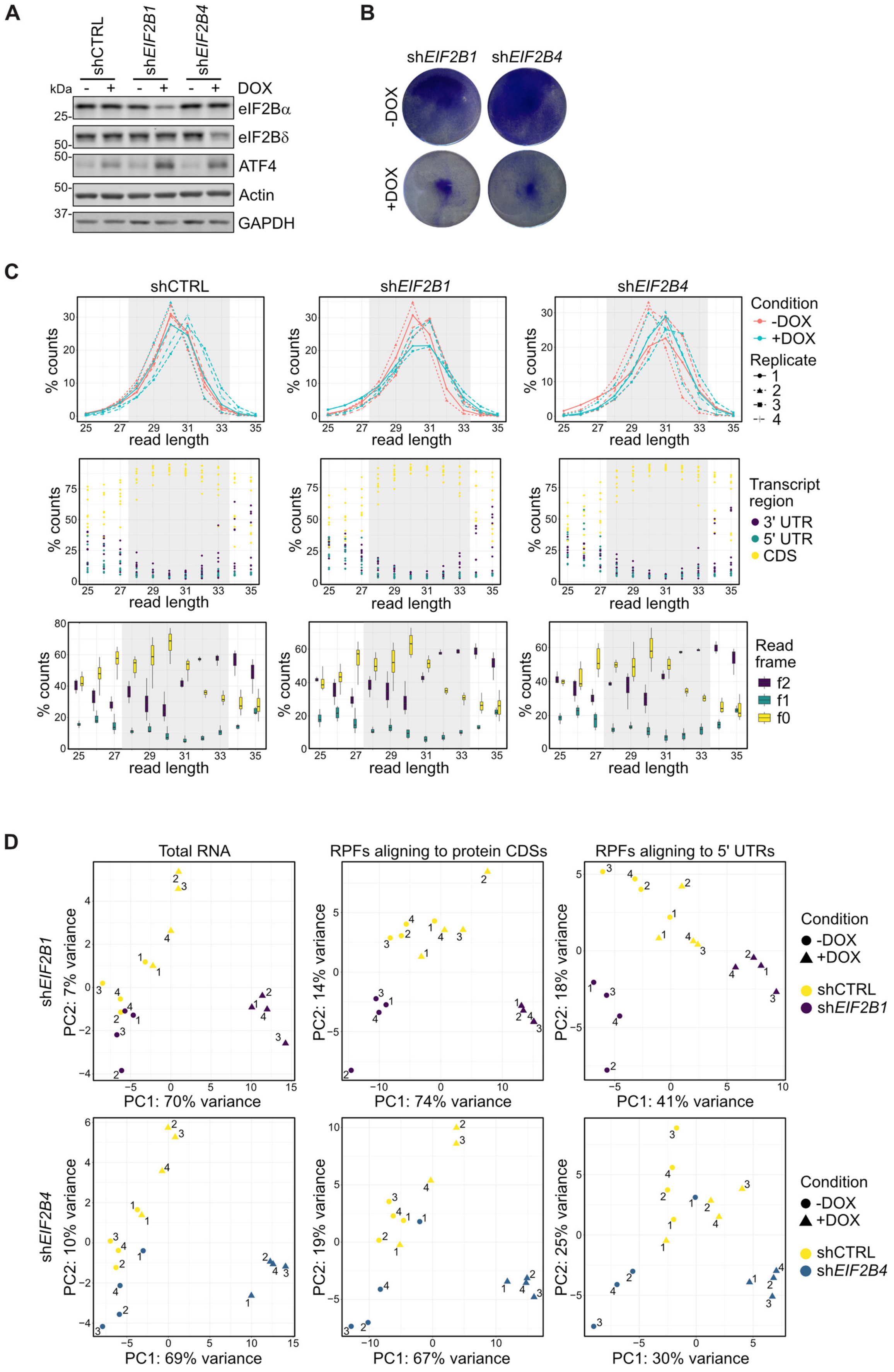
A
shCTRL
shEIF2B1
shEIF2B4
kDa
DOX
eIF2Bα
eIF2Bδ
ATF4
Actin
GAPDH
B
shEIF2B1 shEIF2B4
-DOX
+DOX
C
shCTRL
shEIF2B1
shEIF2B4
% counts
read length
Condition
-DOX
+DOX
Replicate
Transcript region
3' UTR
5' UTR
CDS
Read frame
f2
f1
f0
D
Total RNA
RPFs aligning to protein CDSs
RPFs aligning to 5' UTRs
shEIF2B1
shEIF2B4
PC2: 7% variance
PC1: 70% variance
PC2: 14% variance
PC1: 74% variance
PC2: 18% variance
PC1: 41% variance
PC2: 10% variance
PC1: 69% variance
PC2: 19% variance
PC1: 67% variance
PC2: 25% variance
PC1: 30% variance
Condition
-DOX
+DOX
shCTRL
shEIF2B1
shEIF2B4

 © The Author(s)

Figure EV4. Ribo-seq quality controls (QC).

(**A**) Western blot of indicated proteins in SW480 cells transduced with doxycycline-inducible shCTRL, sh*EIF2B1* or sh*EIF2B4* (5 days of doxycycline treatment (+DOX), DMSO as control (-DOX)), representative of four biological replicates with similar results. GAPDH is probed on the same membrane as eIF2Bδ, and is used as its loading control; Actin is probed on the same membrane as ATF4 and eIF2Bα, and is used as loading control for these two targets. (**B**) Crystal violet staining of SW480 cells transduced with doxycyline-inducible sh*EIF2B1* or sh*EIF2B4* after 7 days of DOX treatment (+DOX, EtOH as control (-DOX)). The experiment was performed once. (**C**) Ribo-seq QC plots; the gray shaded area in all graphs indicates reads of length 28–33 nt, representing the extracted reads for downstream analyses. Columns show results from cells transduced with respective doxycyline-inducible shRNA. The first row shows distribution of ribosome-protected fragments (RPFs) reads for -DOX and +DOX samples, with four replicates plotted independently. The second row shows the transcript region where the RPF reads align to; each dot represents an individual sample. The third row shows the read frame respective to the known codon position within the RPFs. Box plot summary statistics: boxes' lower and upper hinges represent first and third quantiles, lines represent the median, whiskers extend until last data point to a maximum of 1.5* inter-quantile range. (**D**) PCA plots for total cytoplasmic RNA sequencing (first column), RPFs aligning to protein-coding sequences (second column), or RPFs aligning to the 5′ UTRs (third column). Data from each shRNA against either of the two subunits is plotted with the data for the shCTRL (yellow in all plots). Variance is displayed on each axis; size of axes is not variance-scaled. Source data are available online for this figure.

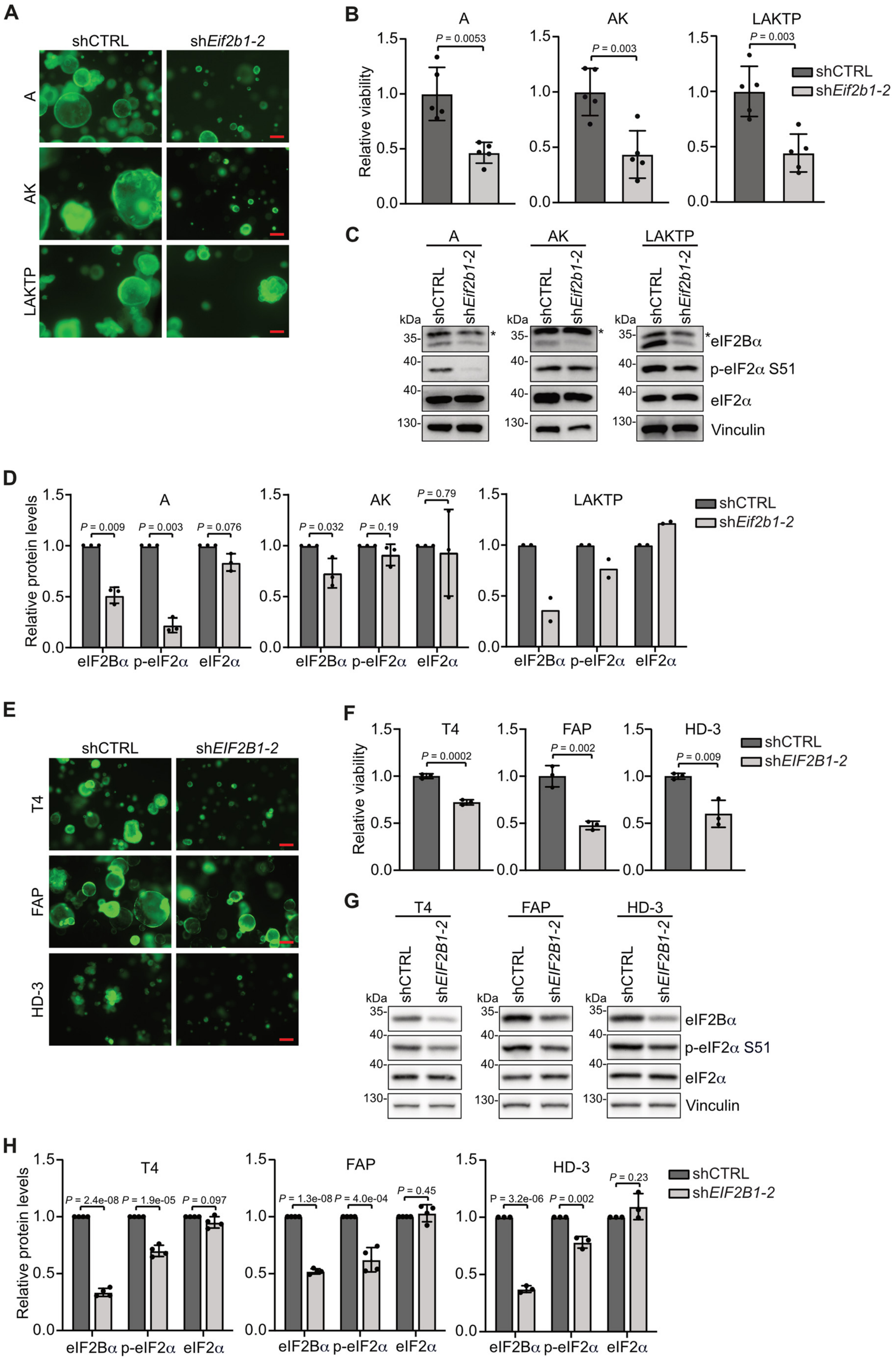
A
shCTRL
shEif2b1-2
A
AK
LAKTP
B
Relative viability
A
AK
LAKTP
P = 0.0053
P = 0.003
P = 0.003
shCTRL
shEif2b1-2
C
A
AK
LAKTP
shCTRL
shEif2b1-2
kDa
eIF2Bα
p-eIF2α S51
eIF2α
Vinculin
D
Relative protein levels
A
AK
LAKTP
P = 0.009
P = 0.003
P = 0.076
P = 0.032
P = 0.19
P = 0.79
eIF2Bα p-eIF2α eIF2α
shCTRL
shEif2b1-2
E
shCTRL
shEIF2B1-2
T4
FAP
HD-3
F
Relative viability
T4
FAP
HD-3
P = 0.0002
P = 0.002
P = 0.009
shCTRL
shEIF2B1-2
G
T4
FAP
HD-3
shCTRL
shEIF2B1-2
kDa
eIF2Bα
p-eIF2α S51
eIF2α
Vinculin
H
Relative protein levels
T4
FAP
HD-3
P = 2.4e-08
P = 1.9e-05
P = 0.097
P = 1.3e-08
P = 4.0e-04
P = 0.45
P = 3.2e-06
P = 0.002
P = 0.23
eIF2Bα p-eIF2α eIF2α
shCTRL
shEIF2B1-2

 © The Author(s)

Figure EV5. Depletion of eIF2Bα by a second independent shRNA reduces viability of murine and human tumor organoids.

(**A**) Pictures of murine A, AK and LAKTP intestinal organoids transduced with doxycycline-inducible shCTRL or sh*Eif2b1-2* (7 days of doxycycline treatment), representative of five biological replicates with similar results. Green signal (GFP) indicates shRNA induction. Scale bar = 200 µm. (**B**) Relative viability of A, AK and LAKTP organoids transduced and treated as described in (**A**). Data show mean ± s.d. ($n = 5$ biological replicates); Student's *t* test. (**C**) Western blot of indicated proteins in A, AK and LAKTP organoids transduced as described in (A), representative of two or three biological replicates with similar results (96 h of doxycycline treatment); *unspecific bands. (**D**) Quantification of eIF2Bα, p-eIF2α S51 and total eIF2α levels, normalized to vinculin, of western blots described in (**C**). Data show mean ± s.d. ($n = 2$ or 3 biological replicates); Student's *t* test. (**E**) Pictures of human T4, FAP, HD-3 PDOs transduced with doxycycline-inducible shCTRL or sh*EIF2B1-2* (7 days of doxycycline treatment), representative of three biological replicates with similar results. Green signal (GFP) indicates shRNA induction. Scale bar = 200 µm. (**F**) Relative viability of T4, FAP, HD-3 PDOs transduced and treated as described in (**E**). Data show mean ± s.d. ($n = 3$ biological replicates); Student's *t* test. (**G**) Western blot of indicated proteins in T4, FAP, HD-3 PDOs transduced as described in (**E**), representative of three or four biological replicates with similar results (96 h of doxycycline treatment). (**H**) Quantification of eIF2Bα, p-eIF2α S51 and total eIF2α levels, normalized to vinculin, of western blots described in (**G**). Data show mean ± s.d. ($n = 3$ or 4 biological replicates); Student's *t* test. Source data are available online for this figure.

© The Author(s)
